# Cobalt-catalysed reductive C–H alkylation of indoles using carboxylic acids and molecular hydrogen†
†Electronic supplementary information (ESI) available: General information concerning experimental procedures, additional tables, figures, schemes, characterization data and NMR spectra of the isolated compounds are available. See DOI: 10.1039/c7sc02117h



**DOI:** 10.1039/c7sc02117h

**Published:** 2017-07-26

**Authors:** Jose R. Cabrero-Antonino, Rosa Adam, Kathrin Junge, Matthias Beller

**Affiliations:** a Leibniz-Institut für Katalyse e.V. an der Universität Rostock , Albert-Einstein-Straße 29a , 18059 Rostock , Germany . Email: matthias.beller@catalysis.de

## Abstract

The direct CH-alkylation of indoles using carboxylic acids is presented for the first time. The catalytic system based on the combination of Co(acac)_3_ and 1,1,1-tris(diphenylphosphinomethyl)-ethane (Triphos, **L1**), in the presence of Al(OTf)_3_ as co-catalyst, is able to perform the reductive alkylation of 2-methyl-1*H*-indole with a wide range of carboxylic acids. The utility of the protocol was further demonstrated through the C3 alkylation of several substituted indole derivatives using acetic, phenylacetic or diphenylacetic acids. In addition, a careful selection of the reaction conditions allowed to perform the selective C3 alkenylation of some indole derivatives. Moreover, the alkenylation of C2 position of 3-methyl-1*H*-indole was also possible. Control experiments indicate that the aldehyde, *in situ* formed from the carboxylic acid hydrogenation, plays a central role in the overall process. This new protocol enables the direct functionalization of indoles with readily available and stable carboxylic acids using a non-precious metal based catalyst and hydrogen as reductant.

## Abstract 2

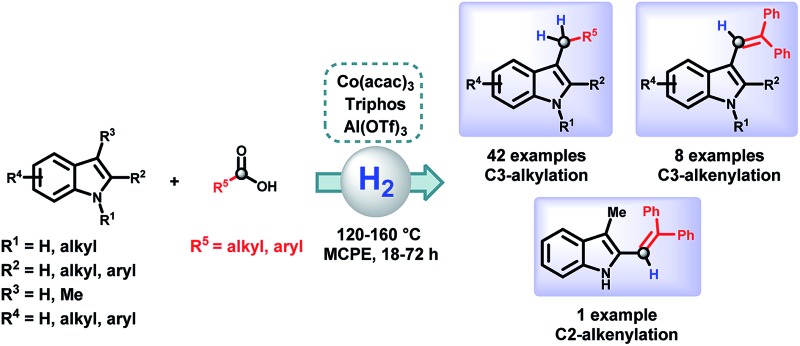
The first direct CH-alkylation of indoles using carboxylic acids and hydrogen is presented. A non-noble metal based catalytic system, [Co(acac)_3_/Triphos/Al(OTf)_3_], efficiently catalyses the alkylation of a variety of indoles with a wide range of carboxylic acids.

## Introduction

**Fig. 1:**
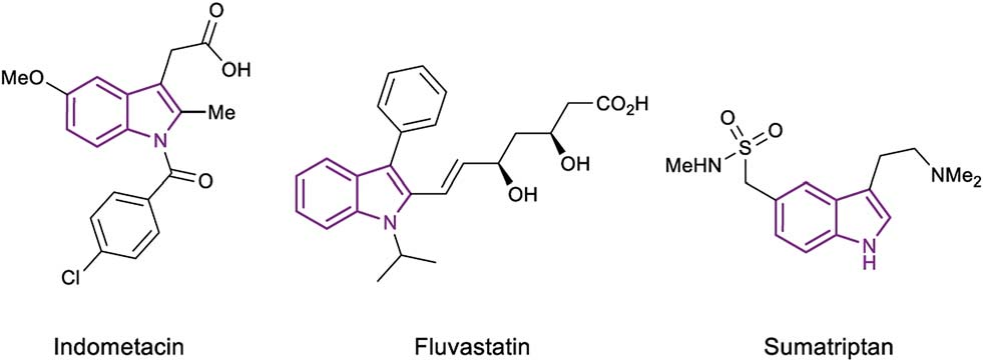
Examples of indole-based marketed drugs.

Indoles are privileged scaffolds, acting as building blocks in multiple natural products.^1^ In fact, the essential amino acid tryptophan and the neurotransmitter serotonin contain the indole moiety in their structure. Inspired by nature, chemists have designed a myriad of indole based compounds with biological activities for its use as drugs or agrochemicals.^2^ For example, indometacin, sumatriptan or fluvastatin are currently marketed as drugs for the treatment of inflammation, migraine or hipercholesterolemia, respectively (Fig. 1). Hence, the development of new methodologies for the derivatization of this class of heterocycles continues to be an important topic in organic synthesis and catalysis.

Traditionally, the cyclisation of pre-functionalized benzoid precursors is the method of choice for the synthesis of substituted indoles.^3^ Since the development of the useful Fisher indole synthesis in the 19^th^ century,^4^ many other catalytic and non-catalytic cyclisation protocols have been described along the years.^5^ In contrast, the direct functionalization of indole has emerged more recently as a preferred methodology as it is more practical and step economical.^6^ Among the several approaches to the direct substitution of indoles, transition metal catalysed CH activations are attractive.^5n,7^ While positions C2 and C3 are the most activated ones, direct C3 alkylations are still limited. Therefore, allylic alkylations,^8^ Baylis–Hillman^9^ and more frequently Friedel–Crafts type reactions^10^ have been especially useful to achieve this transformation. Intimately related with Friedel–Crafts functionalizations are reductive alkylations. Thus, reactions at indole C3 position have been successfully performed employing aldehydes or ketones^11^ in the presence of silanes,^12^ hydrogen^13^ or other reductants.^14^ Despite being a practical transformation, the use of carbonyl compounds limits its applicability as they are sometimes not easily available or undergo unwanted side-reactions, *e.g.*, aldol condensations. Hence, the use of more stable carboxylic acids can be more convenient. However, to the best of our knowledge, this strategy has not been explored and only an example of a related indole methylation using CO_2_ was described by our group in 2014.^15^

Selective hydrogenation of carboxylic acids is a challenging transformation of interest for organic synthesis and catalysis. The first homogeneously catalysed examples of this reaction employed a ruthenium^16^ or an iridium^17^ based complex. Nowadays, the substitution of precious metals by earth abundant non-noble metals, less toxic and expensive, is an exciting goal in catalysis.^18^ In this direction, many examples of homogeneous hydrogenation catalysts based on cobalt have been reported recently.^19,20^ In 2015, the groups of de Bruin and Elsevier described the first base metal catalyst able to hydrogenate carboxylic acids using a system composed by [Co(BF_4_)_2_·6H_2_O/Triphos (**L1**)].^21^ Later on, our group reported the CO_2_ hydrogenation to methanol using a modified related catalyst [Co(acac)_3_/Triphos (**L1**)/HNTf_2_].^22^ Inspired by these precedents, we envisaged the development of a methodology for the alkylation of indole directly using carboxylic acids with a cobalt based catalytic system. Here we describe the first general reductive alkylation of indole C3 position with a variety of carboxylic acids).

## Results and discussion

As a starting point of this project, we selected the reductive C–H alkylation of 2-methyl-1*H*-indole **1a** with acetic acid **2a** (4 eq. respect to **1a**) as benchmark reaction (Table 1). Notably, functionalization of 2-substituted indoles is an interesting task for medicinal chemistry due to increased metabolic stability of the resulting products. Inspired by the previously known catalytic systems *vide supra*, Co(BF_4_)_2_·6H_2_O and Co(acac)_3_ (4 mol%) in combination with 1,1,1-tris(diphenylphosphinomethyl)-ethane, so-called Triphos (ligand **L1**, 2 eq. to Co), were tested under 60 bar of H_2_, at 160 °C, using THF as solvent during 18 h (Table 1, entries 1 and 2). Unfortunately, no activity was observed. Previous works involving carboxylic acid derivatives hydrogenation catalysed by a Ru/Triphos system showed the crucial effect of an acid additive for the catalytic activity.^16a,23^ Usually this additive provides a weakly coordinating counter anion, able to stabilize the active complex, as well as the optimal reaction medium p*K*_a_ for the hydrogenation events to take place. Recently, we showed a similar effect in the Co/Triphos catalysed CO_2_ hydrogenation to methanol, where the presence of HNTf_2_ is required to form the catalytic active species.^22^ Hence, we decided to explore the reductive alkylation of indole **1a** with the Co/Triphos system in the presence of an external acid additive.

**Table 1:** Cobalt-catalysed reductive C–H alkylation of 2-methyl-1*H*-indole (**1a**) with acetic acid (**2a**) and molecular hydrogen: initial screening of the reaction conditions

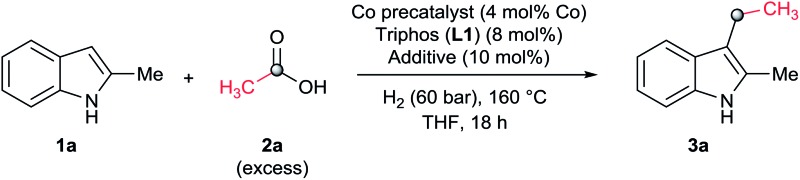					
Entry^*a*^	**2a** (eq.)	[Co]	Additive	Conv.^*b*^ (%)	**3a** ^*b*^(%)
1	4	Co(BF_4_)_2_·6H_2_O	—	15	—
2	4	Co(acac)_3_	—	—	—
3	4	Co(acac)_3_	HNTf_2_	89	42
4	4	Co(acac)_3_	Al(OTf)_3_	>99	52
5	2	Co(acac)_3_	Al(OTf)_3_	>99	69
6^*c*^	2	Co(acac)_3_	Al(OTf)_3_	74	53
7	1.5	Co(acac)_3_	Al(OTf)_3_	89	62
8^*d*^	4	Co(acac)_3_	Al(OTf)_3_	>99	44
9^*d*^	3	Co(acac)_3_	Al(OTf)_3_	91	48
10^*e*^	2	Co(acac)_3_	Al(OTf)_3_	>99	68
11^*e*^	1.75	Co(acac)_3_	Al(OTf)_3_	>99	68
12^*e*^	1.5	Co(acac)_3_	Al(OTf)_3_	94	67
**13** ^*f*^	**1.75**	**Co(acac)**_**3**_	**Al(OTf)**_**3**_	**>99**	**70**
14^*f*^	1.5	Co(acac)_3_	Al(OTf)_3_	90	63
15^*g*^	1.75	Co(acac)_3_	Al(OTf)_3_	94	67
16^*h*^	1.75	Co(acac)_3_	Al(OTf)_3_	35	—

^*a*^Standard reaction conditions: 2-methyl-1*H*-indole **1a** (67.0 mg, 0.5 mmol), Co precatalyst (0.02 mmol, 4 mol%), Triphos **L1** (25.0 mg, 0.04 mmol, 8 mol%, 2 eq. to Co), additive (0.05 mmol, 10 mol%, 2.5 eq. to Co), THF (2 mL), acetic acid **2a** (0.75–1.25 mmol, 1.5–2.5 eq.) and H_2_ (60 bar) at 160 °C.

^*b*^Conversions of **1a** and yields of product **3a** were calculated by GC using hexadecane as internal standard.

^*c*^Run with 2 mol% of Co(acac)_3_, 4 mol% of ligand **L1** (2 eq. to Co) and 5 mol% of Al(OTf)_3_ (2.5 eq. to Co).

^*d*^Run at 140 °C.

^*e*^Run at 40 bar of H_2_.

^*f*^Run at 30 bar of H_2_.

^*g*^Run at 15 bar of H_2_.

^*h*^Run without ligand **L1**.

Gratifyingly, moderate yields of 3-ethyl-2-methyl-1*H*-indole **3a** were detected when catalytic amounts of HNTf_2_ or Al(OTf)_3_ (10 mol%, 2.5 eq. to Co) were added to the [Co(acac)_3_/Triphos (**L1**)] mixture (42–52%, Table 1, entries 3 and 4, respectively). Surprisingly, no traces of *N*-alkylated products or bis(indole) derivatives were observed in the reaction mixtures by GC analysis.

Next, a more detailed investigation regarding the effect of alkylating agent **2a** amount, catalyst loading, pressure and temperature on the catalytic activity was carried out (Table 1, entries 5–15). In general, high conversions of **1a** were detected, although only moderate yields of the desired C3-alkylated product **3a** were obtained, indicating some degradation of the indoles. Nevertheless, the yield of the desired C3-alkylated indole derivative **3a** could be improved to 70% using 1.75 eq. of acetic acid **2a** under 30 bar of hydrogen and 160 °C (Table 1, entry 13). Milder reaction temperatures or lower catalysts loadings and hydrogen pressures did not afford better yields of **3a** (Table 1, entries 6, 8, 9 and 15). In the absence of ligand **L1**, no desired product was detected and only some degradation of **1a** was observed (Table 1, entry 16).

**Fig. 2:**
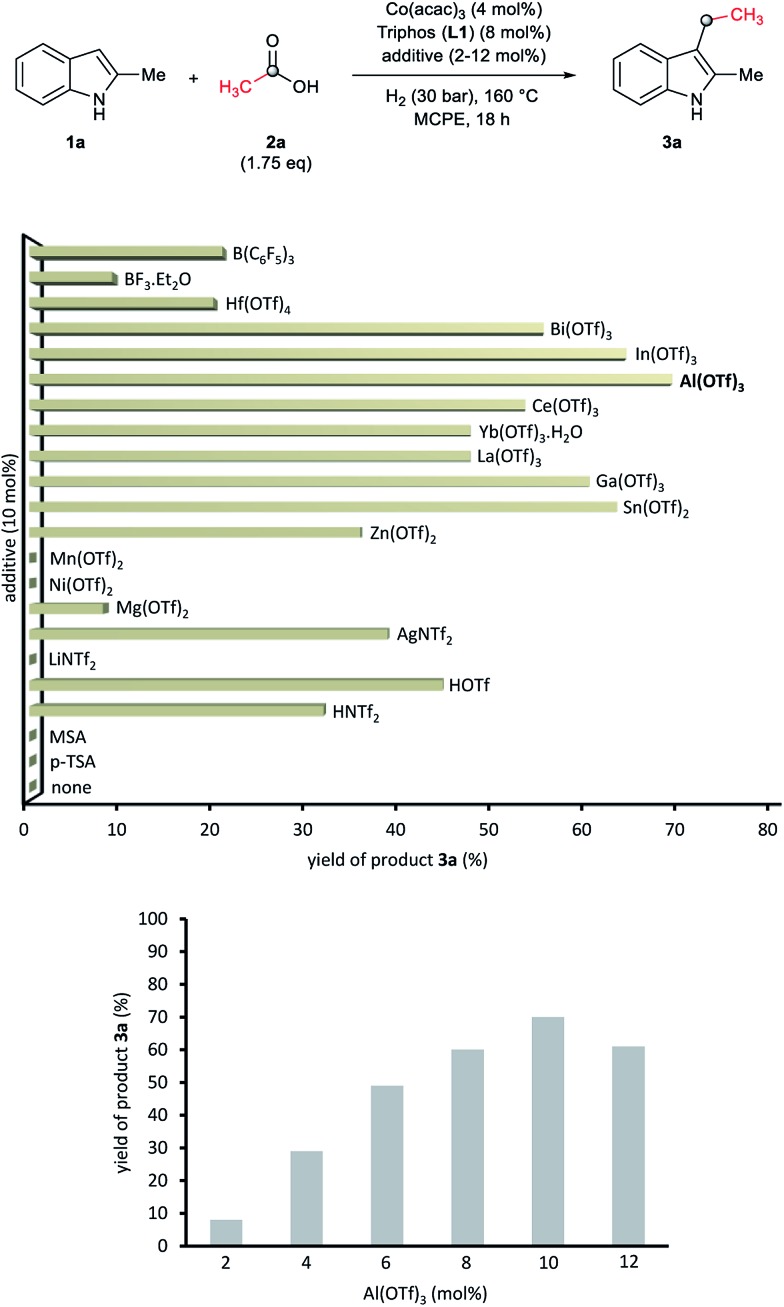
Up: Testing of additives in the cobalt-catalysed reductive C–H alkylation of 2-methyl-1*H*-indole **1a** with acetic acid **2a** and molecular hydrogen. Bottom: Influence of the Al(OTf)_3_ loading in the formation of product **3a** from **1a** and **2a**. Standard reaction conditions: 2-methyl-1*H*-indole **1a** (67.0 mg, 0.5 mmol), Co(acac)_3_ (7.2 mg, 0.02 mmol, 4 mol%), Triphos **L1** (25.0 mg, 0.04 mmol, 8 mol%, 2 eq. to Co), additive (2–12 mol%, 0.5–3 eq. to Co), THF (2 mL), acetic acid **2a** (50.3 μL, 0.875 mmol, 1.75 eq.) and H_2_ (30 bar) at 160 °C. The yields of product **3a** were calculated by GC using hexadecane as internal standard.

At this point we became interested in studying the influence of the acid co-catalyst. Fig. 2 (up) shows that, among the different Brönsted and Lewis acid additives tested for the CH-alkylation of the indole **1a**, aluminium(III) trifluoromethanesulfonate [Al(OTf)_3_] afforded the best yield of **3a** (70%). Other Lewis acid additives such as In(OTf)_3_, Ga(OTf)_3_ or Sn(OTf)_2_ also promoted the desired transformation and gave yields of alkylated product **3a** above 60%. In order to improve the model reaction further on, we varied the relative Al(OTf)_3_ amount (with respect to the cobalt precatalyst). As shown in Fig. 2 (bottom) 10 mol% of Al(OTf)_3_ (2.5 eq. to Co) gave the optimal yield (70% of **3a**).

Next, the influence of the solvent was investigated in detail (see ESI, Table S1†). The presence of water (10% v/v respect to THF) was detrimental for the activity of the catalytic system, affording very poor yields of product **3a** (7%, Table S1,† entry 2). In general, other ether-type solvents gave similar results compared to THF (Table S1,† entries 2–8), being methyl cyclopentyl ether (MCPE) the one that gave the best result (Table S1,† entry 6, 83% yield **3a**). Also in toluene a good yield of the desired indole **3a** was obtained (76%, Table S1,† entry 9). In contrast, the catalyst was totally inactive when DMF was used (Table S1,† entry 10).

To our delight, the employment of MCPE allowed to use a lower catalyst loading (2 mol% of cobalt) with excellent results (Table 2, entry 5, 89% yield of **3a**). Blank experiments revealed that the presence of all the three components of the catalytic system, [Co(acac)_3_/Triphos (**L1**)/Al(OTf)_3_], are required for the reductive alkylation of indole (Table 2, entries 6–10). In addition, these experiments showed that the partial degradation of **1a** can be attributed mainly to the presence of the acid additive (Table 2, entries 7 and 10).

**Table 2:** Cobalt-catalysed reductive C–H alkylation of 2-methyl-1*H*-indole (**1a**) with acetic acid (**2a**) and molecular hydrogen: optimization of the reaction conditions using MCPE as solvent

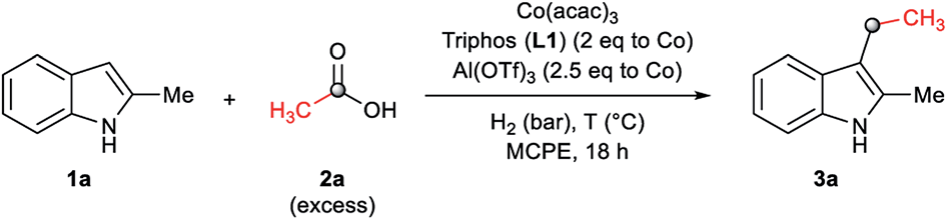						
Entry^*a*^	*T* (°C)	H_2_ (bar)	**2a** (eq.)	[Co] (mol%)	Conv.^*b*^ (%)	**3a** ^*b*^ (%)
1	160	30	1.75	4	>99	83
2	160	30	1.5	4	>99	79
3	160	30	1.25	4	86	62
4	160	30	2.5	2	>99	85
**5**	**160**	**30**	**1.75**	**2**	**>99**	**89**
6^*c*^	160	30	1.75	2	24	—
7^*d*^	160	30	1.75	2	57	—
8^*e*^	160	30	1.75	2	14	—
9^*c*^	160	30	1.75	—	12	—
10^*d*^	160	30	1.75	—	54	—
11^*f*^	160	30	1.75	2	64	42
12^*g*^	160	30	1.75	2	47	—
13	160	30	1.5	2	87	76
14^*c*^	160	30	2.5	1	47	31
15^*d*^	160	30	1.75	1	40	23
16	160	15	1.75	2	56	27
17^*h*^	160	—	1.75	2	15	—
18	140	30	1.75	4	96	68
19	140	30	2.5	4	96	70
20	140	60	1.75	2	69	41
21	140	60	2.5	2	83	49
22^*i*^	160	30	1.75	2	70	64

^*a*^Standard reaction conditions: 2-methyl-1*H*-indole **1a** (67.0 mg, 0.5 mmol), Co(acac)_3_ (1–4 mol%), Triphos **L1** (2–8 mol%, 2 eq. to Co), Al(OTf)_3_ (2.5–10 mol%, 2.5 eq. to Co), MCPE (2 mL), acetic acid **2a** (0.75–1.25 mmol, 1.5–2.5 eq.) and H_2_ (15–60 bar) at 140–160 °C.

^*b*^Conversion of **1a** and yield of product **3a** were calculated by GC using hexadecane as internal standard.

^*c*^Run without ligand **L1** and Al(OTf)_3_.

^*d*^Run without ligand **L1**.

^*e*^Run without Al(OTf)_3_.

^*f*^Run with 3 mol% of ligand **L1** (1.5 eq. to Co).

^*g*^Run with 2 mol% of ligand **L1** (1 eq. to Co).

^*h*^Run with 30 bar of N_2_.

^*i*^Run at 6 h.

Notably, the relative amounts of ligand **L1** with respect to the cobalt precursor had a significant influence on the product yield. When the reaction was conducted using 1.5 and 1 equivalents of ligand, moderate yields (42%) of **3a** or no product were detected, respectively (Table 2, entries 11 and 12), showing that 2 eq. of Triphos (**L1**) are the minimum required to perform the reaction efficiently.

Next, the catalytic activity of different metal pre-catalysts was evaluated under the optimized reaction conditions (30 bar of H_2_, 160 °C, 1.75 eq. of **2a**, 2 mol% of cobalt and 18 h; see Table 1 and ESI, Fig. S1†). Among the different Co(iii), Co(ii), Ru(iii), Mn(iii), Fe(iii) and Cu(ii) acetylacetonate complexes tested, Co(acac)_3_ was the one that gave the best yields of the desired alkylated indole **3a** (89%, see ESI, Fig. S1†). In addition, Co(acac)_2_·H_2_O afforded a slightly lower yield (78% yield of **3a**, Fig. S1†), while other related metal salts were not active. These results indicate that cobalt is unique for this reaction.

Among the different cobalt-based precatalysts containing counteranions such as [BF_4_^–^], [ClO_4_^–^], [OAc^–^], [F^–^], [NO_3_^–^] and [SO_4_^2–^] (see ESI, Fig. S1†) tested for the alkylation of indole **1a**, only Co(BF_4_)_2_·6H_2_O and Co(SO_4_)·7H_2_O promoted the formation of the product **3a**, albeit in lower yields (21% and 8% yield of **3a**, respectively, Fig. S1†).

With regard to the ligand, we compared the activity of Triphos **L1** with several multidentate (**L2–L11**) and one monodentate ligands (**L11**) (see ESI, Scheme S1†). All the other tested ligands showed lower activities than Triphos (**L1**), and only the tridentate ligands **L2** and **L3** or the tetradentate **L5** afforded 3-ethyl-2-methyl-1*H*-indole **3a**, though in low yields (18%, 17% and 3%, respectively, Scheme S1†).

At this point, we decided to explore the general applicability of the cobalt-based system in the C3-alkylation of 2-methyl-1*H*-indole **1a** using a wide range of carboxylic acids (Table 3). In some cases, higher catalyst loadings (up to 4 mol%) or excess of the alkylating agent (up to 2.5 eq.) were needed in order to achieve a total conversion of indole **1a**.

**Table 3:** [Co/Triphos (**L1**)]-catalysed reductive C(3)–H alkylation of 2-methyl-1*H*-indole (**1a**) using different carboxylic acids and molecular hydrogen

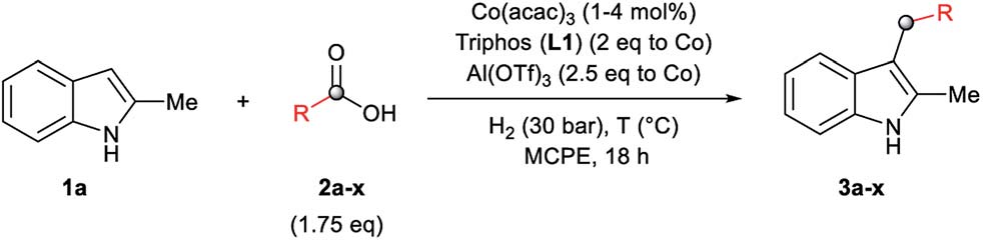				
Entry^*a*^	*T* (°C)	[Co] (mol%)	Product **3**	Yield^*b*^ (%)
1	160	2	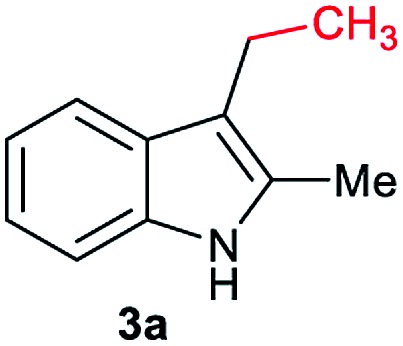	[80]
2	140	2	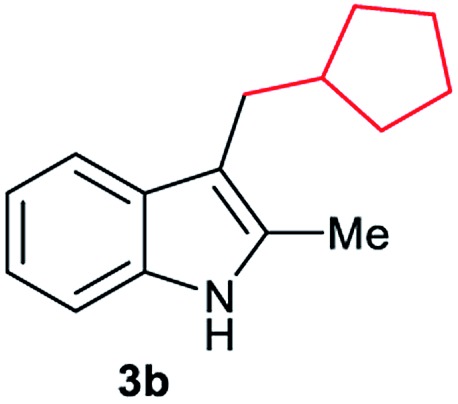	[80]
3	160	2	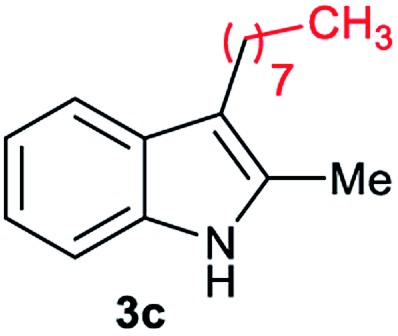	[67]
4	160	2	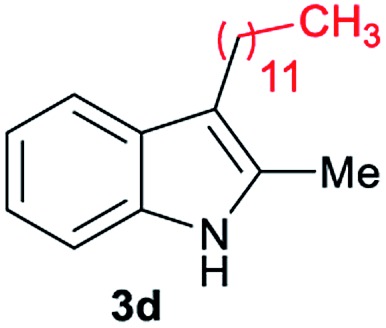	[74]
5^*c*^	140	2	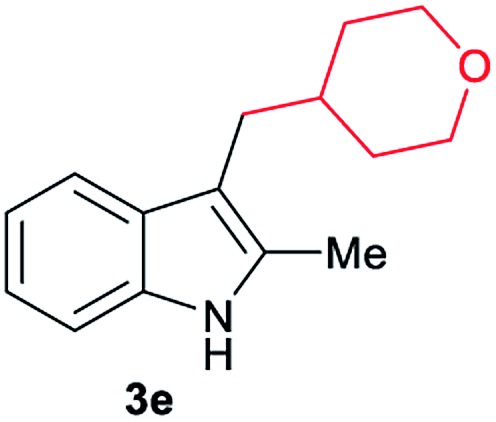	[59]
6	160	2	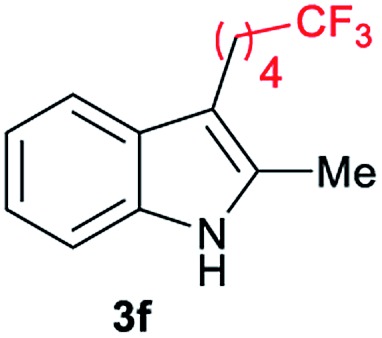	[74]
7^*c*^	160	2	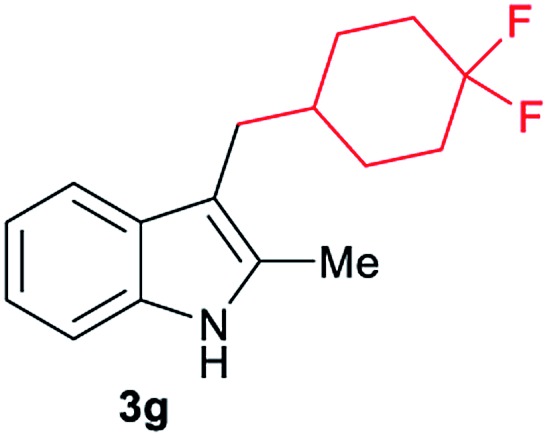	[70]
8	140	4	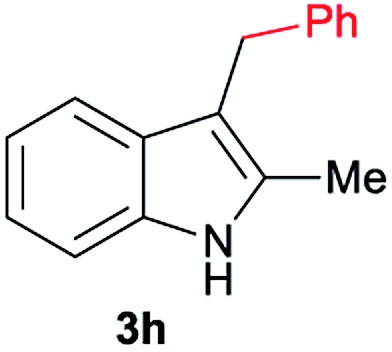	[75]
9^*c*^	160	4	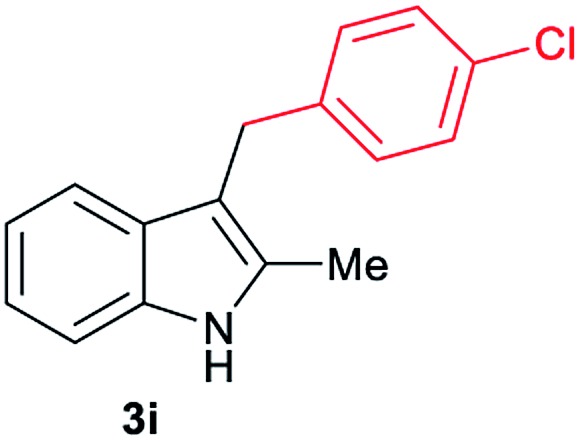	[64]
10	140	2	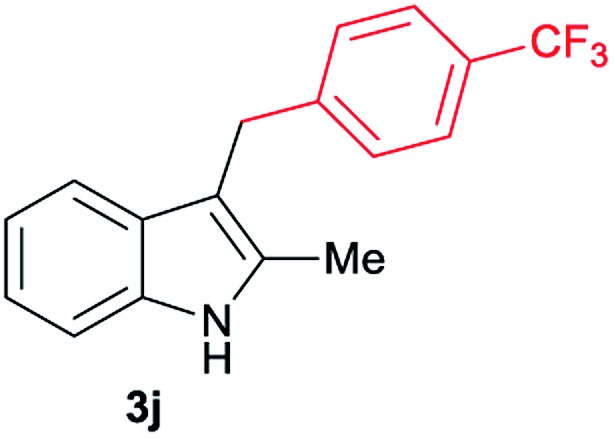	[49]
11^*c*^	160	3	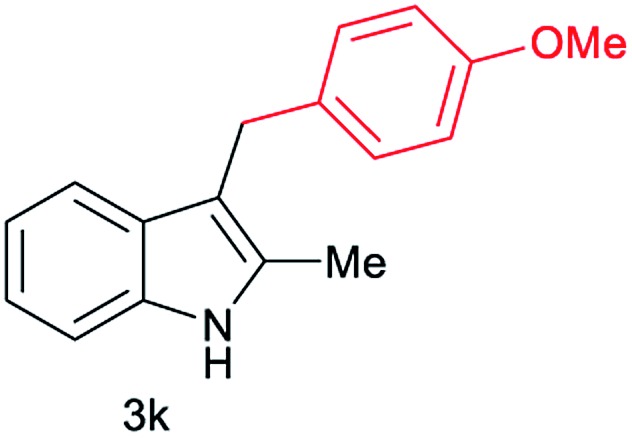	[46]
12	160	3	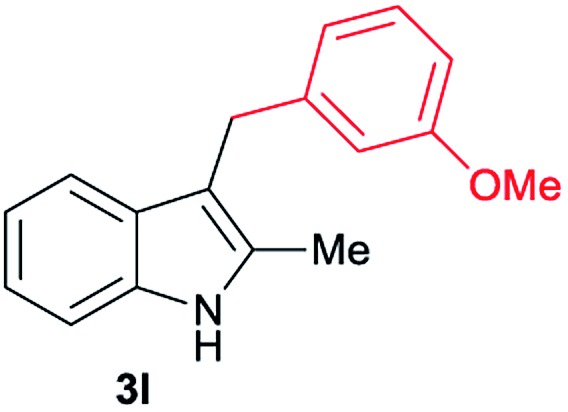	[68]
13^*d*^	140	2	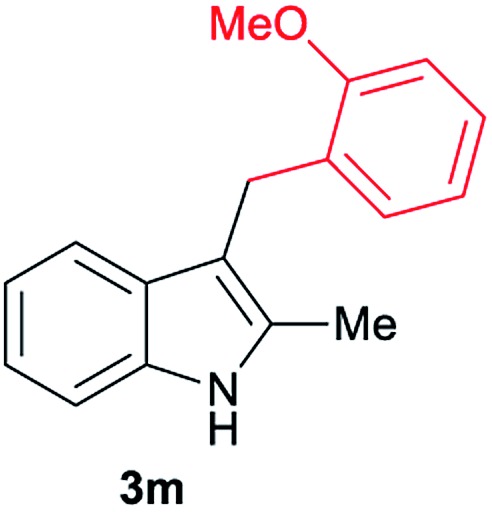	[47]
14	140	1	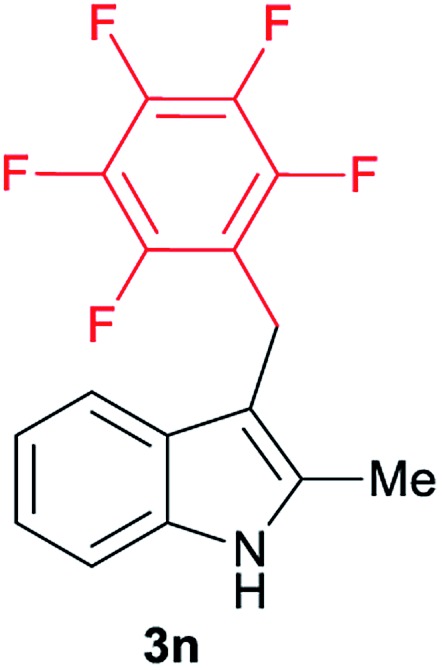	[35]
15	140	2	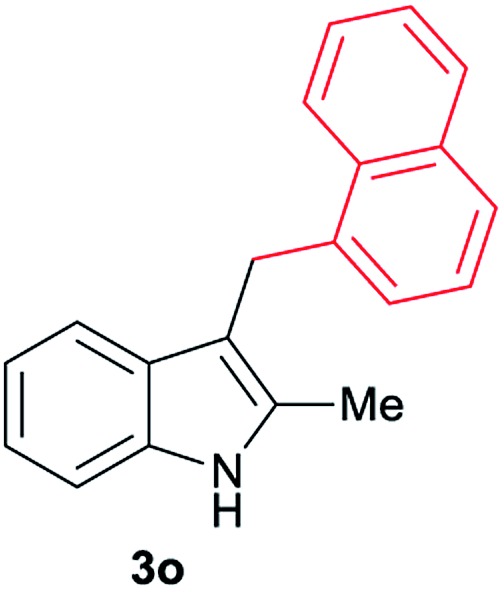	[73]
16	140	2	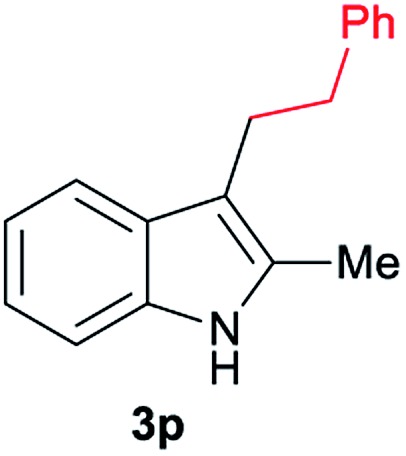	[78]
17	160	3	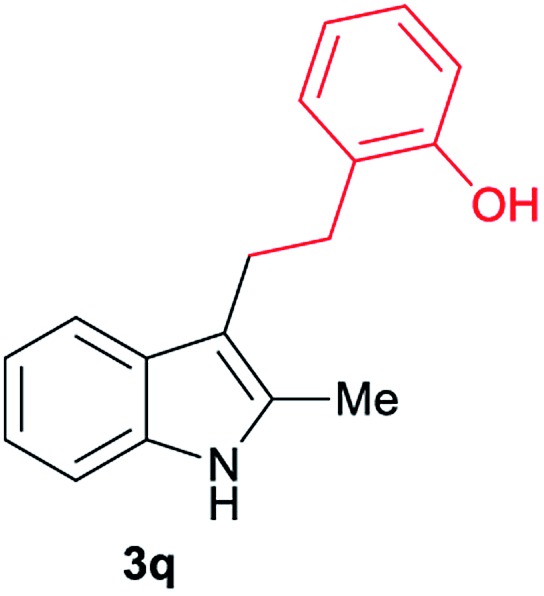	[70]
18	140	2	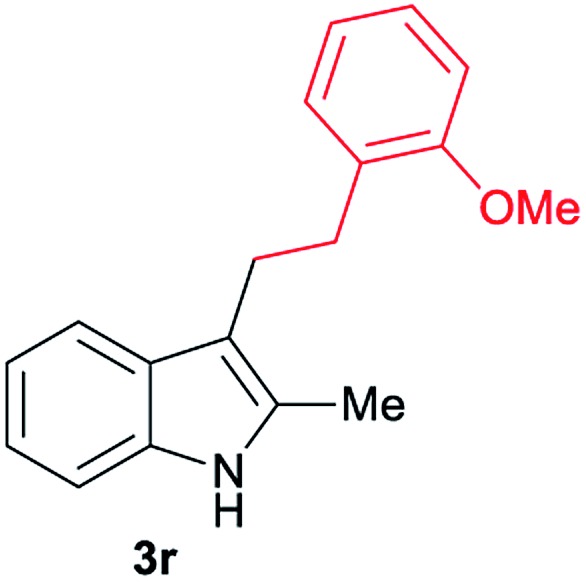	[55]
19	160	3	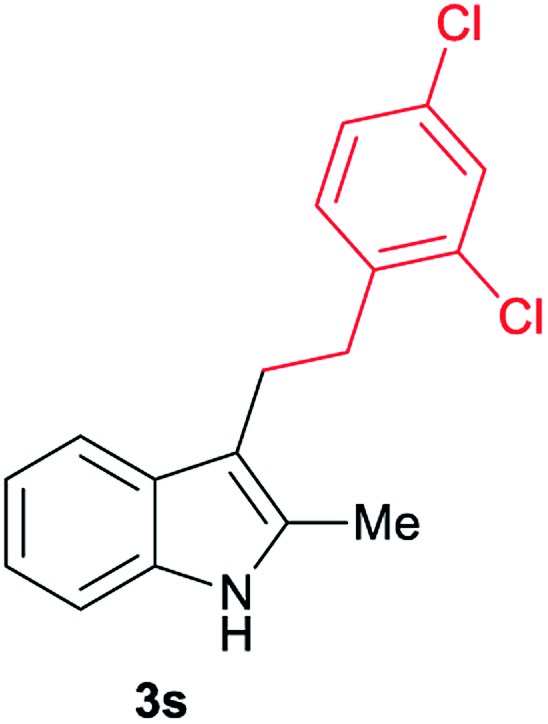	[72]
20	160	2	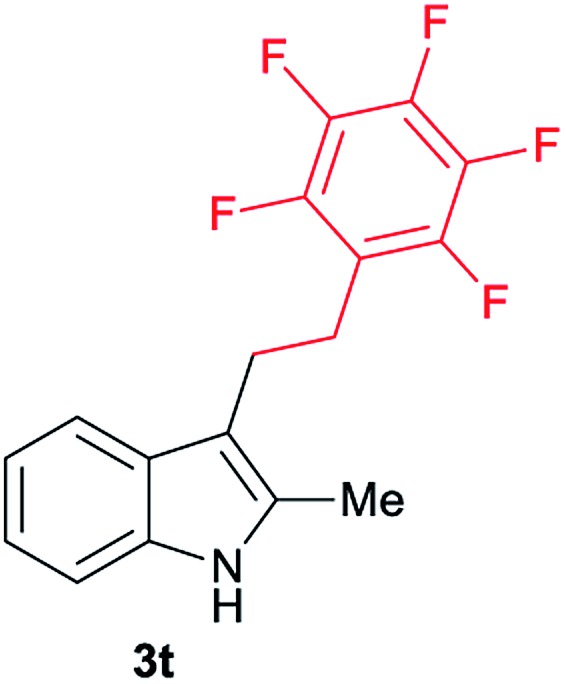	[42]
21	140	2	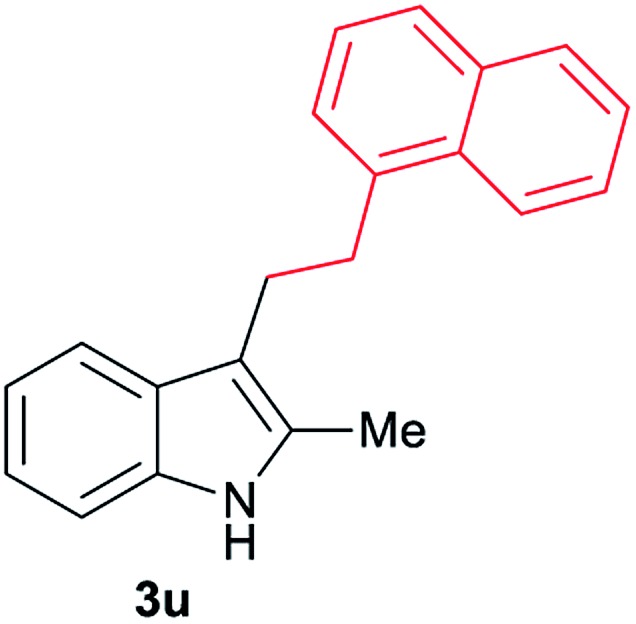	[64]
22	140	2	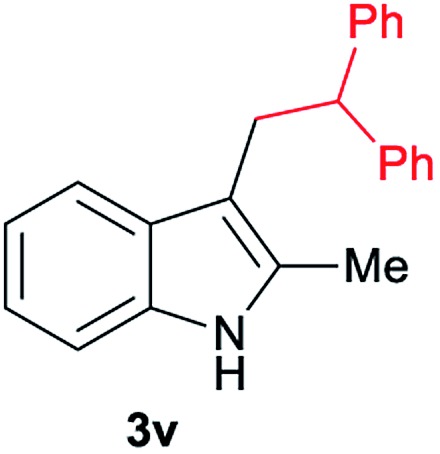	[70]
23	140	2	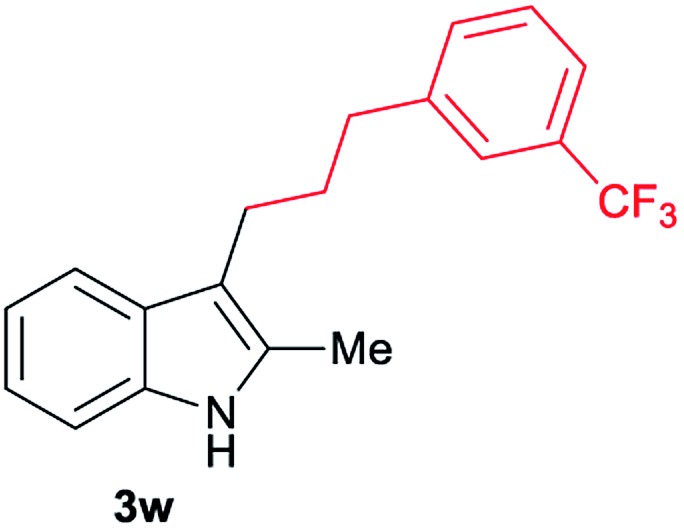	[72]
24	140	2	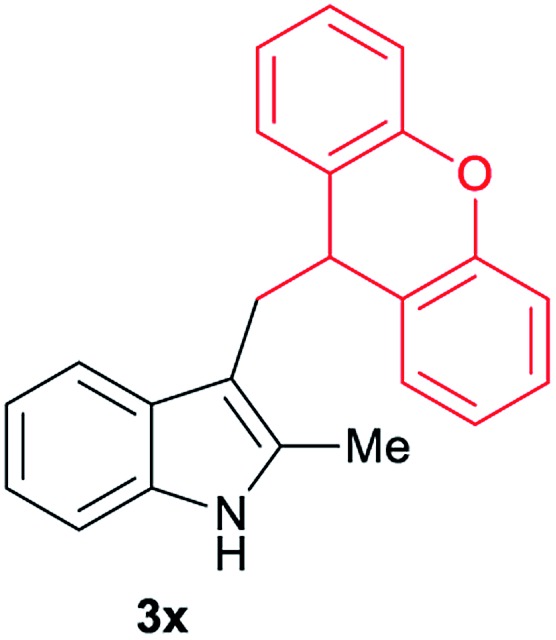	[55]

^*a*^Standard reaction conditions: 2-methyl-1*H*-indole **1a** (67.0 mg, 0.5 mmol), Co(acac)_3_ (1–4 mol%), Triphos **L1** (2–8 mol%, 2 eq. to Co), Al(OTf)_3_ (2.5–10 mol%, 2.5 eq. to Co), MCPE (2 mL), carboxylic acid (0.875 mmol, 1.75 eq.) and H_2_ (30 bar) at 140–160 °C. The selectivity to the desired product was >95% in all cases. In some examples degradation problems were observed.

^*b*^Yield of isolated product after column chromatography on silica.

^*c*^Run with (1.75 mmol, 2.5 eq.) of carboxylic acid.

^*d*^Run at 5 h.

Aliphatic carboxylic acids **2a–g**, including examples containing heteroatoms such as oxygen-(**2e**) or fluorine-(**2f** and **2g**), afforded the corresponding C3-alkylated indoles **3a–g** in good to very good isolated yields (59–80%, Table 3, entries 1–7). Moreover, benzoic acid **2h** and different *o*-, *m*- and *p*-substituted benzoic acids containing chloro (**2i**), trifluoromethyl (**2j**) or methoxy groups (**2k–m**), could also be used as alkylating agents giving good isolated yields of the C3-benzylated indole derivatives **3h–m** (46–75%, Table 3, entries 8–13). Gratifyingly, perfluorinated benzoic acid **2n** and naphthoic acid **2o** also gave the desired functionalized indole derivatives **3n** and **3o** in moderate to good yields after isolation (36 and 73%, Table 3, entry 14 and 15, respectively). In addition, more sensitive phenylacetic acid **2p**, naphthaleneacetic acid **2u** as well as 2-hydroxy-(**2q**), 2-methoxy-(**2r**), 2,4-dichloro-(**2s**) and perfluoro-(**2t**) substituted phenylacetic acids were successfully employed as alkylating agents of indole **1a** (42–78%, Table 3, entries 16–21). In general, no clearly correlation between the reactivity and the electronic character of the substituent attached to the benzene ring of the carboxylic acid could be observed. Finally, diphenylacetic acid **2v**, 3-(3-(trifluoromethyl)phenyl)propanoic acid **2w** and 9-xanthene carboxylic acid **2x**, as an example of (hetero)aromatic acid, exhibited good activity (55–70%, Table 3, entries 22–24). These examples illustrate the potential of the base metal catalysed methodology as a practical tool for introducing molecular diversity in the indole core using readily available carboxylic acids as alkylating agents.

**Scheme 1:**
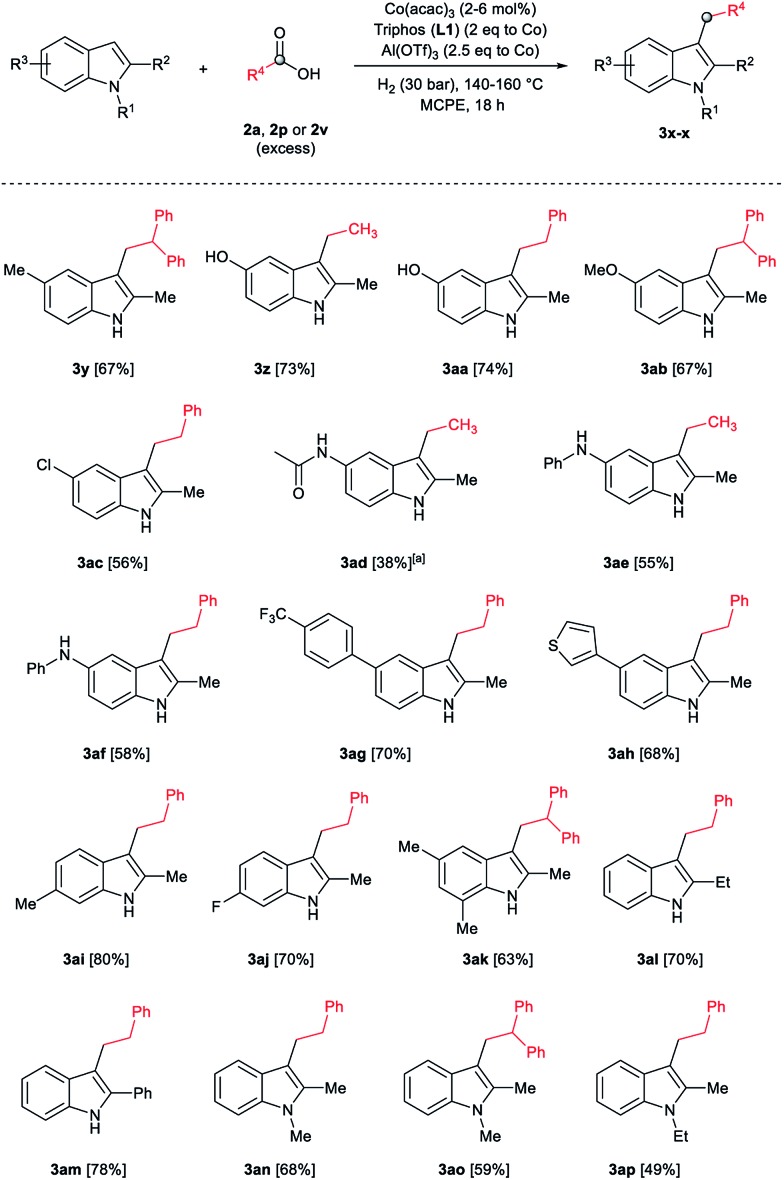
Standard reaction conditions: indole (0.5 mmol), Co(acac)_3_ (1–4 mol%), Triphos **L1** (2–8 mol%, 2 eq. to Co), Al(OTf)_3_ (2.5–10 mol%, 2.5 eq. to Co), MCPE (2 mL), carboxylic acid (0.875–1.75 mmol, 1.75–3.5 eq.) and H_2_ (30 bar) at 140–160 °C during 18–60 h. Yield of isolated product after column chromatography on silica are given. The selectivity to the desired product, calculated by GC-MS, was >90% in all cases. In some examples degradation problems were observed. Specific reaction conditions for substrate **3y** and **3ak**: Co(acac)_3_ (4 mol%), carboxylic acid (0.875 mmol, 1.75 eq.), 160 °C, 18 h; for substrate **3z**: Co(acac)_3_ (2 mol%), carboxylic acid (0.875 mmol, 1.75 eq.), 160 °C, 18 h; for substrates **3aa** and **3ai**: Co(acac)_3_ (2 mol%), carboxylic acid (0.875 mmol, 1.75 eq.), 140 °C, 18 h; for substrate **3ab**: Co(acac)_3_ (3 mol%), carboxylic acid (0.875 mmol, 1.75 eq.), 160 °C, 48 h; for substrate **3ac**: Co(acac)_3_ (6 mol%), carboxylic acid (1.5 mmol, 3.5 eq.), 160 °C, 18 h; for substrates **3ad–ae**: Co(acac)_3_ (4 mol%), carboxylic acid (1.5 mmol, 3 eq.), 160 °C, 18 h; for substrate **3af**: Co(acac)_3_ (3 mol%), carboxylic acid (0.875 mmol, 1.75 eq.), 160 °C, 18 h; for substrates **3ag–ah** and **3aj**: Co(acac)_3_ (5 mol%), carboxylic acid (1.25 mmol, 2.5 eq.), 160 °C, 18 h; for substrate **3al**: Co(acac)_3_ (2 mol%), carboxylic acid (1.25 mmol, 2.5 eq.), 160 °C, 18 h; for substrate **3am**: Co(acac)_3_ (6 mol%), carboxylic acid (1.25 mmol, 2.5 eq.), 160 °C, 18 h; for substrate **3an**: Co(acac)_3_ (3 mol%), carboxylic acid (1.25 mmol, 2.5 eq.), 160 °C, 18 h; for substrate **3ao**: Co(acac)_3_ (6 mol%), carboxylic acid (0.875 mmol, 1.75 eq.), H_2_ (60 bar), 160 °C, 60 h; for substrate **3ap**: Co(acac)_3_ (6 mol%), carboxylic acid (1.25 mmol, 2.5 eq.), 160 °C, 18 h. [a] 2-Methyl-1*H*-indol-5-amine was used as starting material.

After showing the reductive alkylation using different carboxylic acids, we investigated the reaction of several substituted indole derivatives (Scheme 1). Using the optimized conditions, a variety of indoles were reacted with acetic acid **2a**, phenylacetic acid **2p** or diphenylacetic acid **2v**. In general, for the same indole, **2p** was the most reactive carboxylic acid followed by **2v** and **2a**, as the less reactive one. As shown in Scheme 1, several 5-substituted 2-methyl-1*H*-indole derivatives – some of them previously synthetized by us (see ESI† for experimental details) – containing methyl, hydroxy, methoxy, chloro, amino, phenylamino, trifluoromethyl and thiophenyl groups were well tolerated. The desired C3-alkylated products **3y-ah** were obtained in moderate to good yields after isolation (38–74%, Scheme 1). Interestingly, in the case of 5-amino-2-methyl-1*H*-indole reacting with acetic acid, the main product was the one corresponding to the simultaneous C3 alkylation and amidation, being possible to isolate the amide **3ad** in moderate yields (38%, Scheme 1). This result indicates that the [Co(acac)_3_/Triphos (**L1**)/Al(OTf)_3_] catalysts exhibits selectivity towards the hydrogenation of a carboxylic acid in the presence of an amide. Moreover, 6-substituted as well as 5,7-substituted indole substrates were also successfully alkylated giving products **3ai–ak** in 63–80% isolated yields. At this point it was interesting to explore the tolerance of our protocol towards different alkyl and aryl substituents in the C2 position as well as in the nitrogen atom of the indole. To our delight, the reductive alkylation of this indoles class was successfully achieved under different reaction conditions using phenylacetic acid **2p** and diphenylacetic acid **2v** as alkylating agents. Thus, the desired C3-alkylated products **3al–ap** could be isolated in 49–70% yields (Scheme 1).

As expected, in case of 2,3-dimethyl-1*H*-indole **S1** (Scheme S2,† eqn (A)), no products were detected after its reaction with acetic acid **2a**, discarding a possible *N*-alkylation catalysed by our system. In agreement with this observation, for 3-methyl-1*H*-indole **1b** where the C3 position is blocked, only very low amounts of C2-ethylated product **S3** (<2%) were detected (Scheme S2,† eqn (B)), confirming the lower nucleophilicity of C2 position in comparison with C3. Finally, when simple indole **1c** or *N*-methyl derivative **1d** were used, low conversions (<20%) and poor yields of the desired C3-ethylated products **S3** and **S5** were observed (10 and 8%, respectively, Scheme S2,† eqn (C) and (D)). Interestingly, in these two cases small amounts of bis(indole) compounds **S4** and **S6** (<2 and <5%, respectively, Scheme S2,† eqn (C) and (D)) were detected.

**Scheme 2:**
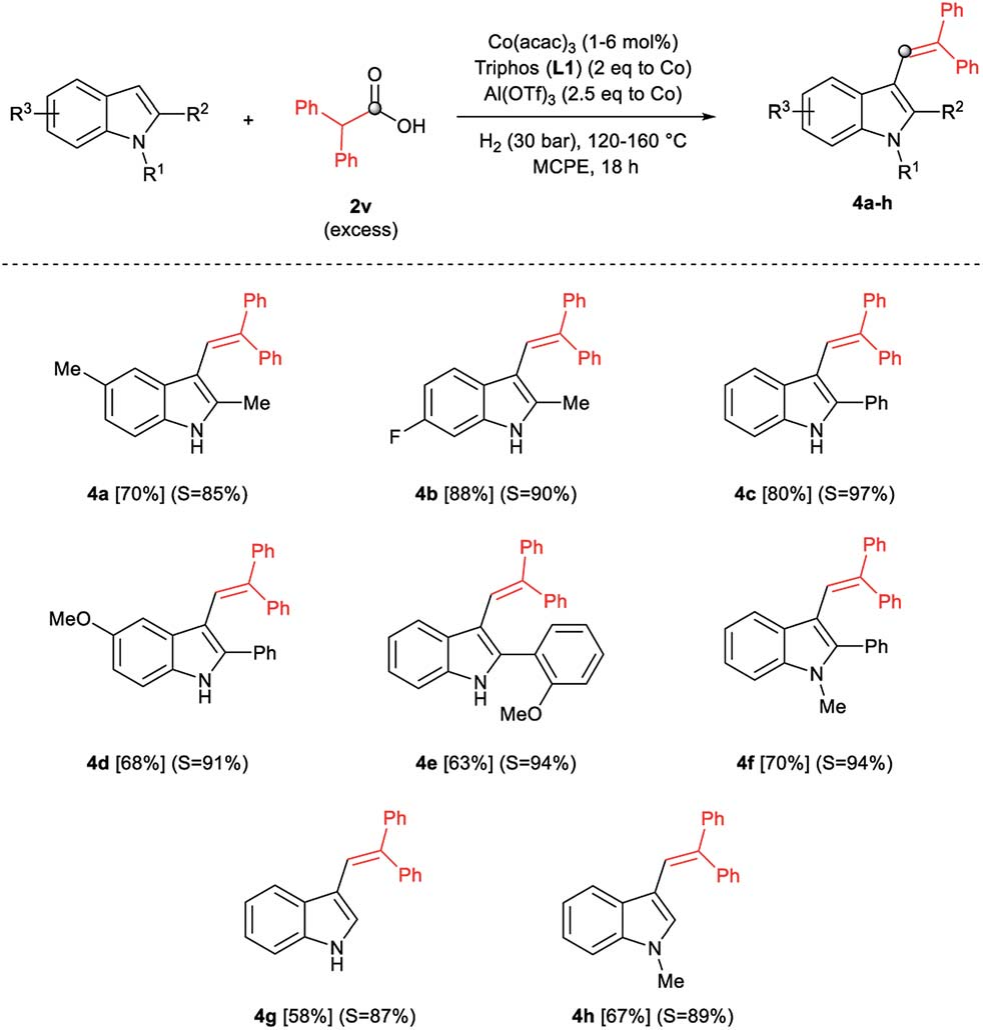
Cobalt-catalysed reductive selective C(3)–H diphenylvinylation of different indoles using diphenylacetic acid **2v** and molecular hydrogen. Standard reaction conditions: indole (0.5 mmol), Co(acac)_3_ (1–6 mol%), Triphos **L1** (2–12 mol%, 2 eq. to Co), Al(OTf)_3_ (2.5–15 mol%, 2.5 eq. to Co), MCPE (2 mL), diphenylacetic acid **2v** (1.25–1.75 mmol, 2.5–3.5 eq.) and H_2_ (30 bar) at 120–160 °C over 18 h. Yield of isolated products after column chromatography on silica are given between brackets. Between parentheses is shown the selectivity to the desired alkene *vs.* alkane by-product, calculated by GC-MS. In some examples degradation problems were observed. Specific reaction conditions for substrate **4a**: Co(acac)_3_ (2 mol%), carboxylic acid (1.75 mmol, 3.5 eq.), 120 °C; for substrate **4b**: Co(acac)_3_ (2 mol%), carboxylic acid (1.75 mmol, 3.5 eq.), 130 °C; for substrates **4c–e**: Co(acac)_3_ (4 mol%), carboxylic acid (1.25 mmol, 2.5 eq.), 160 °C; for substrate **4f**: Co(acac)_3_ (6 mol%), carboxylic acid (1.75 mmol, 3.5 eq.), 160 °C; for substrate **4g**: Co(acac)_3_ (1 mol%), carboxylic acid (2.25 mmol, 4.5 eq.), 140 °C; for substrate **4h**: Co(acac)_3_ (4 mol%), carboxylic acid (1.75 mmol, 3.5 eq.), 120 °C.

During the development of the alkylation reactions of different indoles using diphenylacetic acid **2v** (see Scheme 1), we observed alkenylation of selected substrates as a side reaction.^24^ A fine control of the reaction parameters (temperature, pressure of hydrogen, catalyst loading, amount of alkylating agent and reaction time) allowed us to selectively stop the reaction at the alkene stage (Scheme 2). For example, 5-methyl- and 6-fluoro-2-methyl-1*H*-indole derivatives were selectively alkenylated (selectivity alkene *vs.* alkane >85%) affording the desired tri-substituted alkenes **4a** and **4b** in very good isolated yields (70 and 88%, respectively, Scheme 2). To the best of our knowledge such alkenylations using carboxylic acids have not described, yet.

**Scheme 3:**
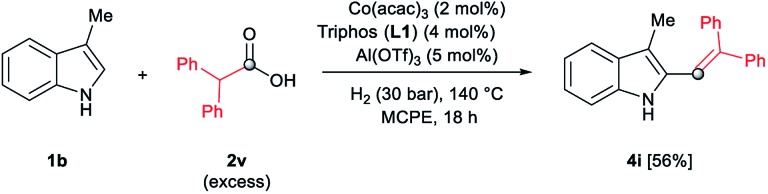
Cobalt-catalysed reductive C(2)–H diphenylvinylation of 3-methyl-1*H*-indole **1b** using diphenylacetic acid **2v** and molecular hydrogen. Standard reaction conditions: 3-methyl-1*H*-indole **1b** (67.0 mg, 0.5 mmol), Co(acac)_3_ (3.6 mg, 0.01 mmol, 2 mol%), Triphos **L1** (12.5 mg, 0.02 mmol, 4 mol%, 2 eq. to Co), Al(OTf)_3_ (11.9 mg, 0.025 mmol, 5 mol%, 2.5 eq. to Co), MCPE (2 mL), diphenylacetic acid **2v** (486.0 mg, 2.25 mmol, 4.5 eq.) and H_2_ (30 bar) at 140 °C over 18 h. Yield of isolated product after column chromatography on silica is given. The selectivity to the alkene product **4i** was 97%.

In addition, different C2-phenyl and/or nitrogen-alkyl substituted indole derivatives gave the corresponding alkene products **4c–f** with high selectivities towards alkene *vs.* alkane (>94%) and good isolated yields (63–80%, Scheme 2). Notably, the simple indole **1c** and the *N*-methyl substituted **1d**, unreactive towards alkylation when acetic acid was used (*vide supra*), were also successfully vinylated in C3 position using diphenylacetic acid **2v**. In these cases only traces of C2-alkenylated products were detected (<2%), observing a full selectivity to C3 *vs.* C2-position. In fact, C3-alkenylated products **4g** and **4h** were isolated in moderate to good yields (58 and 67%, respectively, Scheme 2) with selectivities alkene *vs.* alkane between 87–89%. Finally, despite the lower nucleophicility of the indole C2 position, it was possible to perform the cobalt catalysed C2 alkenylation of 3-methyl-1*H*-indole **1b** by using an excess of diphenylacetic acid **2v** as alkylating source. The desired C2-alkenylated product **4i** could be isolated in 56% (Scheme 3). Hence, this novel non-precious metal based methodology opens the way to the development of further transformations for the C2 and C3-functionalization of indoles.

**Fig. 3:**
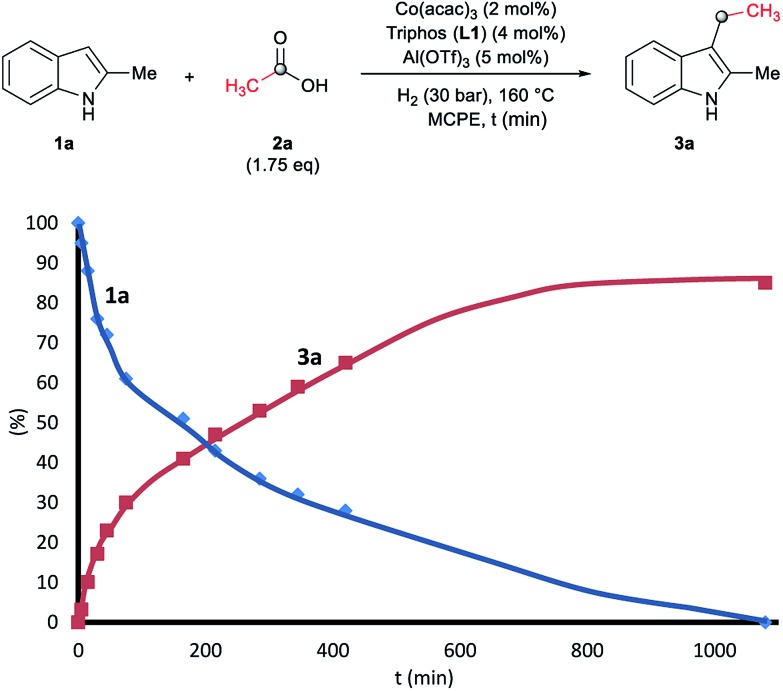
Concentration/time profile for 2-methyl-1*H*-indole **1a** (blue line) and 3-ethyl-2-methyl-1*H*-indole **3a** (red line) in the reductive C–H alkylation of **1a** with acetic acid **2a** at 160 °C and 30 bar of molecular hydrogen. Standard reaction conditions: 2-methyl-1*H*-indole **1a** (3.0 mmol, 402.0 mg), Co(acac)_3_ (21.6 mg, 0.06 mmol, 2 mol%), Triphos **L1** (75.0 mg, 0.12 mmol, 4 mol%, 2 eq. to Co), Al(OTf)_3_ (71.4 mg, 0.15 mmol, 5 mol%, 2.5 eq. to Co), MCPE (12.0 mL), acetic acid **2a** (301.8 μL, 5.25 mmol, 1.75 eq.) and H_2_ (30 bar) at 160 °C. Percentages of **1a** and **3a** were calculated by GC using hexadecane as internal standard.

**Fig. 4:**
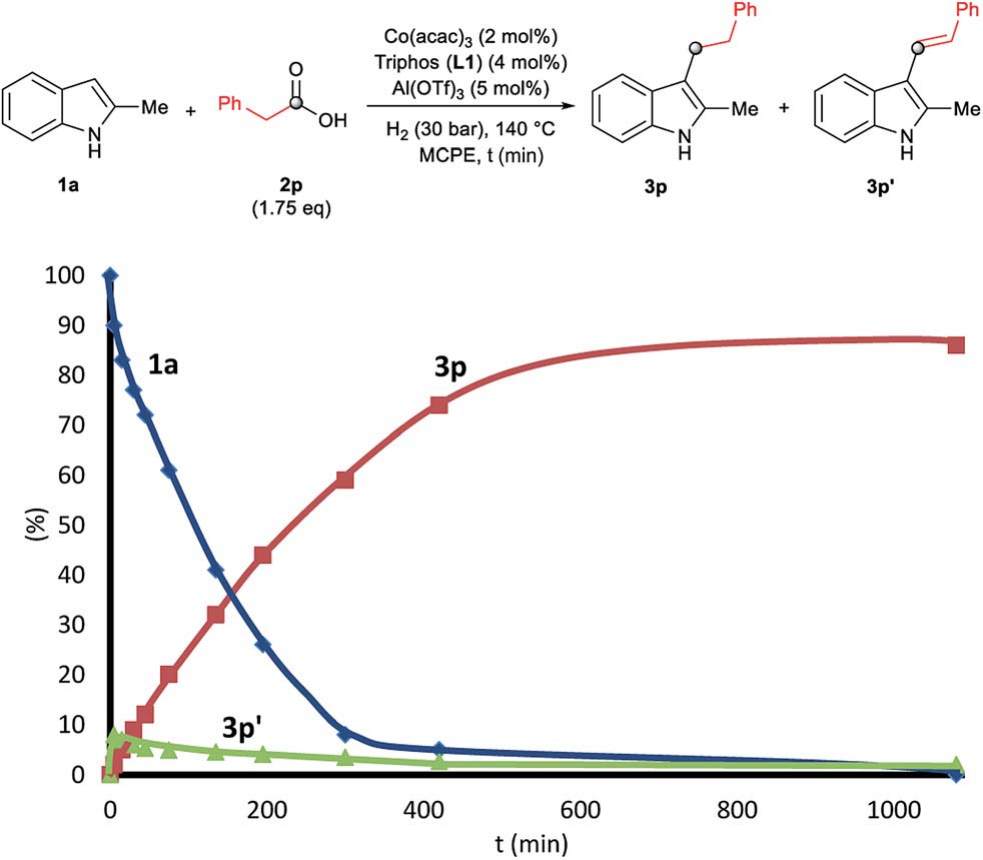
Concentration/time profile for 2-methyl-1*H*-indole **1a** (blue line), 2-methyl-3-phenethyl-1*H*-indole **3p** (red line) and (*E*)-2-methyl-3-styryl-1*H*-indole **3p′** (green line) in the reductive C–H alkylation of **1a** with phenylacetic acid **2p** at 140 °C and 30 bar of molecular hydrogen. Standard reaction conditions: 2-methyl-1*H*-indole **1a** (3.0 mmol, 402.0 mg), Co(acac)_3_ (21.6 mg, 0.06 mmol, 2 mol%), Triphos **L1** (75.0 mg, 0.12 mmol, 4 mol%, 2 eq. to Co), Al(OTf)_3_ (71.4 mg, 0.15 mmol, 5 mol%, 2.5 eq. to Co), MCPE (12.0 mL), phenylacetic acid **2p** (720.0 mg, 5.25 mmol, 1.75 eq.) and H_2_ (30 bar) at 140 °C. Percentages of **1a**, **3p** and **3p′** were calculated by GC using hexadecane as internal standard.

**Scheme 4:**
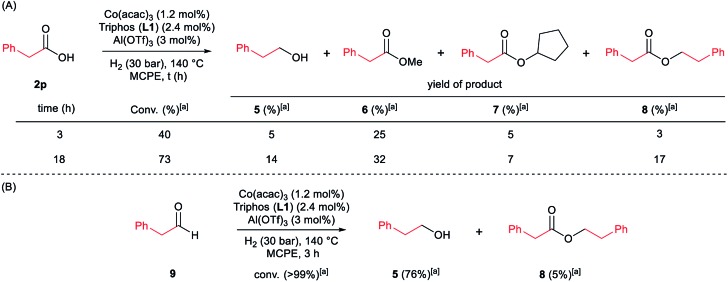
Control experiments in the hydrogenation of phenylacetic acid **2p** (A) or phenylacetaldehyde **9** (B) in the absence of 2-methyl-1*H*-indole **1a**. Standard reaction conditions: phenylacetic acid **2p** or phenylacetaldehyde **9** (0.875 mmol), Co(acac)_3_ (3.6 mg, 0.01 mmol, 1.2 mol%), Triphos **L1** (12.5 mg, 0.02 mmol, 2.4 mol%, 2 eq. to Co), Al(OTf)_3_ (11.9 mg, 0.025 mmol, 3 mol%, 2.5 eq. to Co), MCPE (2 mL) at 140 °C during 3–18 h. [a] Conversion of **2p** and **9** and yield of products **5**, **6**, **7** and **8** were calculated by GC using hexadecane as internal standard.

**Scheme 5:**
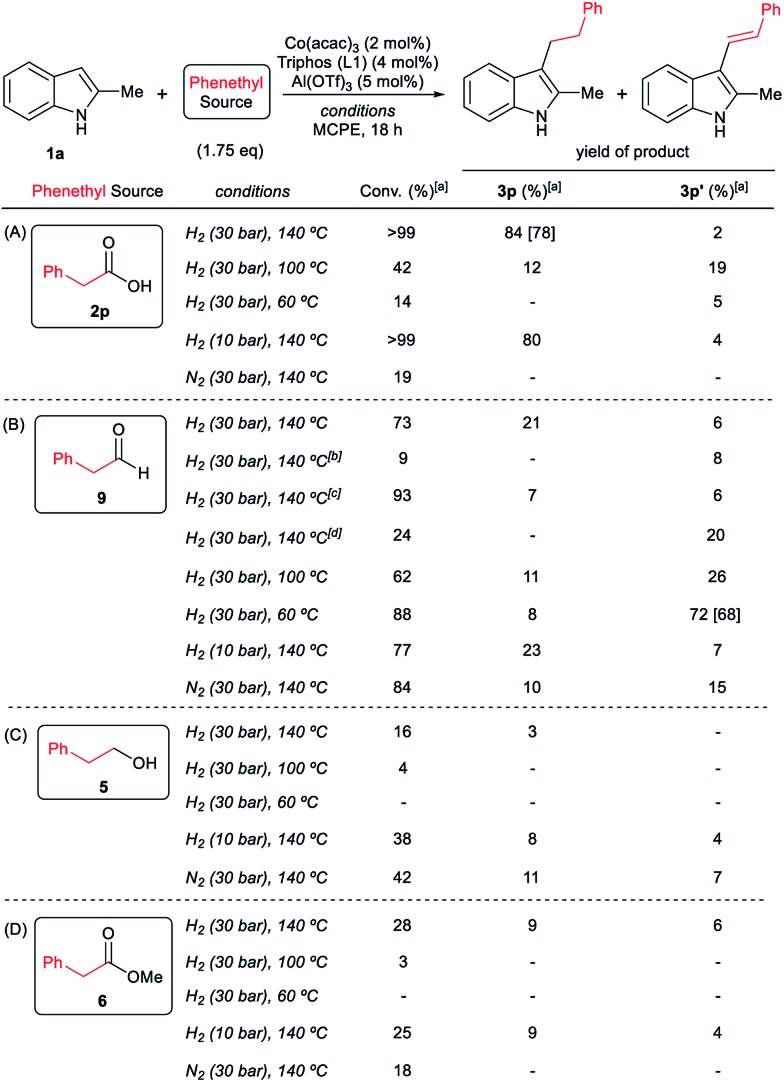
Control experiments in the C–H phenethylation of 2-methyl-1*H*-indole **1a** using phenylacetic acid **2p** (A), phenylacetaldehyde **9** (B), phenethyl alcohol **5** (C) and methyl phenylacetate **6** (D) as alkylating agent. Standard reaction conditions: 2-methyl-1*H*-indole **1a** (67.0 mg, 0.5 mmol), Co(acac)_3_ (3.6 mg, 0.01 mmol, 2 mol%), Triphos **L1** (12.5 mg, 0.02 mmol, 4 mol%, 2 eq. to Co), Al(OTf)_3_ (11.9 mg, 0.025 mmol, 5 mol%, 2.5 eq. to Co), MCPE (2 mL), alkylating agent (0.875 mmol, 1.75 eq.) and H_2_ (10–30 bar) or N_2_ (30 bar) at 60–140 °C during 18 h. [a] Conversion of **1a** and yield of products **3p** and **3p′** were calculated by GC using hexadecane as internal standard. Between brackets is shown the isolated yield of the product after column chromatography on silica. [b] Run without Al(OTf)_3_. [c] Run without Co(acac)_3_ and Triphos **L1**. [d] Run without Co(acac)_3_, Triphos **L1** and Al(OTf)_3_.

**Scheme 6:**
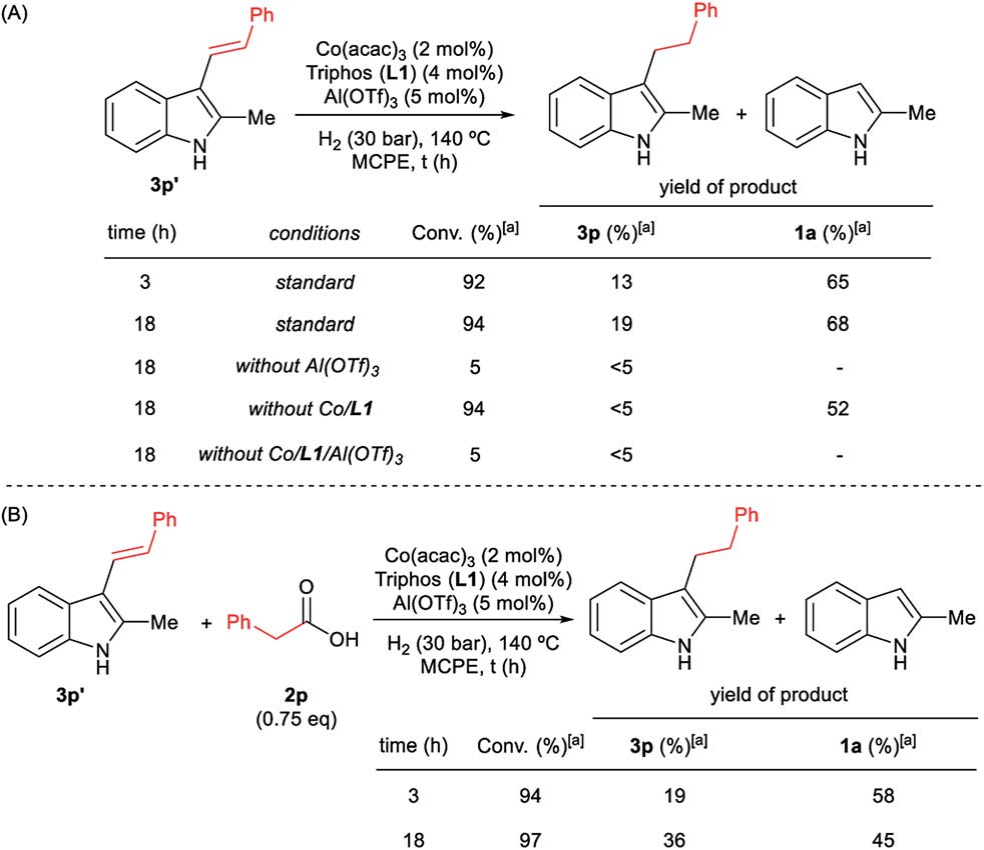
Control experiments in the hydrogenation of (*E*)-2-methyl-3-styryl-1*H*-indole **3p′** in the absence (A) or in the presence (B) of phenylacetic acid **2p**. Standard reaction conditions: (*E*)-2-methyl-3-styryl-1*H*-indole **3p′** (116.7 mg, 0.5 mmol), phenylacetic acid **2p** or not (25.7 mg, 0.375 mmol, 0.75 eq.), Co(acac)_3_ (3.6 mg, 0.01 mmol, 2 mol%), Triphos **L1** (12.5 mg, 0.02 mmol, 4 mol%, 2 eq. to Co), Al(OTf)_3_ (11.9 mg, 0.025 mmol, 5 mol%, 2.5 eq. to Co), MCPE (2 mL) at 140 °C during 3–18 h. [a] Conversion of **3p′** and yield of products **3p** and **1a** were calculated by GC using hexadecane as internal standard.

To gain insight into the mechanism of the cobalt-catalysed reductive alkylation of indoles, some kinetic studies (see Fig. 3 and 4) and control experiments were performed (see Schemes 4–6). Fig. 3 and 4 show the concentration/time profiles for the reductive alkylation of 2-methyl-1*H*-indole **1a** with acetic acid **2a** and phenylacetic acid **2p**, respectively (optimized reaction conditions for each acid). It should be noted that no induction periods were observed in any of the experiments, indicating that the active catalytic species is easily formed. Interestingly, in the case of the reaction with phenylacetic acid **2p** it is possible to detect low amounts of the intermediate (*E*)-alkene **3p′**, observed in slightly higher concentrations at initial reaction times (see Fig. 4). This observation indicates that the hydrogenation of the alkene **3p′** is not the rate determining step of the process.

Next, we carried out the reaction under the optimized conditions for phenylacetic acid **2p** but in the absence of any indole (Scheme 4A). This experiment revealed that our catalytic system is able to hydrogenate the carboxylic acid to the corresponding alcohol in moderate yields (14% of 2-phenylethanol **5** and 17% of 2-phenetyl phenylacetate **8** were detected, Scheme 4A). The experiment at shorter reaction times and using phenylacetaldehyde **9** as starting material, afforded 2-phenylethanol **5** in good yields (76%, Scheme 4B).

At this point it was interesting to compare the reactivity of several possible alkylating agents, all of them being related derivatives of phenylacetic acid **2p**. Thus, reactions of indole **1a** were performed in the presence of the [Co(acac)_3_/Triphos (**L1**)/Al(OTf)_3_] system at 140 °C and 30 bar of H_2_, using phenylacetic acid **2p**, phenylacetaldehyde **9**, phenethyl alcohol **5** and methyl phenylacetate **6** (usually formed from phenylacetic acid and methanol coming from MCPE cleavage) (Scheme 5A–D). Under these conditions, the best alkylating agent was the acid derivative (Scheme 5A), while the ester **6** and the alcohol **5** only afforded only traces of the alkylated product **3p** (Scheme 5C and D, respectively). The aldehyde **9** gave the alkylated indole **3p** in low yield (21%) together with traces of the alkenylated product **3p′** (Scheme 5B). This latter result is especially surprising considering the general use and high reactivity of aldehydes as alkylating agents. At milder reaction temperatures of 100 and 60 °C, a decrease in the catalytic activity was observed for phenylacetic acid **2p**, while phenethyl alcohol **5** and methyl phenylacetate **6** were totally unreactive (Scheme 5A, C and D, respectively). Interestingly, the reaction with phenylacetaldehyde **9** at 100 and 60 °C gave moderate and good yields of the alkenylated product **3p′**, respectively (Scheme 5B). This observation suggests that the aldehyde could be an important reaction intermediate that, when formed slowly from the carboxylic acid at the optimized conditions, is able to afford the alkylated product in good yields. The low yields of **3p** obtained when the reaction was performed starting from the aldehyde at 140 °C, are partially explained by the aldehyde fast hydrogenation to the corresponding alcohol (Scheme 4B) and/or its degradation at this temperature. Notably, when the reaction was performed at low hydrogen pressure or in its total absence, low yields of the alkylated and alkenylated products were observed with phenethyl alcohol **5** and phenylacetaldehyde **9** as alkylating agents (Scheme 5B and C, respectively). This indicates that dehydrogenation pathways could also be partially contributing to the formation of the alkylated product, either by forming the aldehyde from the alcohol or by abstracting hydrogen from water or alcohols in the reaction media.

In addition, it was demonstrated that the presence of the three components of the catalytic system, [Co(acac)_3_/Triphos (**L1**)/Al(OTf)_3_], was required for the hydrogenation of the (*E*)-styryl indole **3p′** to the alkylated product **3p** (Scheme 6A). Surprisingly, when **3p′** was submitted to the optimized reaction conditions, 2-methyl-1*H*-indole **1a** was formed in 68% yield. In the presence of 0.75 eq. of phenylacetic acid **2p**, lower amounts of indole **1a** were detected (Scheme 6B).

Finally, to obtain some information about the catalytic system, the resting state of the reaction mixture after standard conditions was studied by ^31^P NMR (Fig. S2a†). In addition, the same experiment was repeated for several mixtures to see the effect of indole **1a**, acetic acid **2a** and Al(OTf)_3_ in the nature of resting state (Fig. S2b–d†). In all these experiments it was possible to detect signals between 18–32 ppm, corresponding to phosphine ligands coordinated to cobalt,^25^ and similar to the spectra obtained for the [Co(acac)_3_/Triphos (**L1**)/HNTf_2_] system.^22^ Interestingly, when the [Co(acac)_3_/Triphos (**L1**)/Al(OTf)_3_] system was employed, signals in the ranges of 18.7–20.2 ppm and 28.7–31.5 ppm were detected (Fig. S2a–c†). However, in the absence of Al(OTf)_3_ (Fig. S2d†), only signals around 30 ppm were observed, clearly indicating a main role of the additive in the formation of some of the active complexes.

**Fig. 5:**
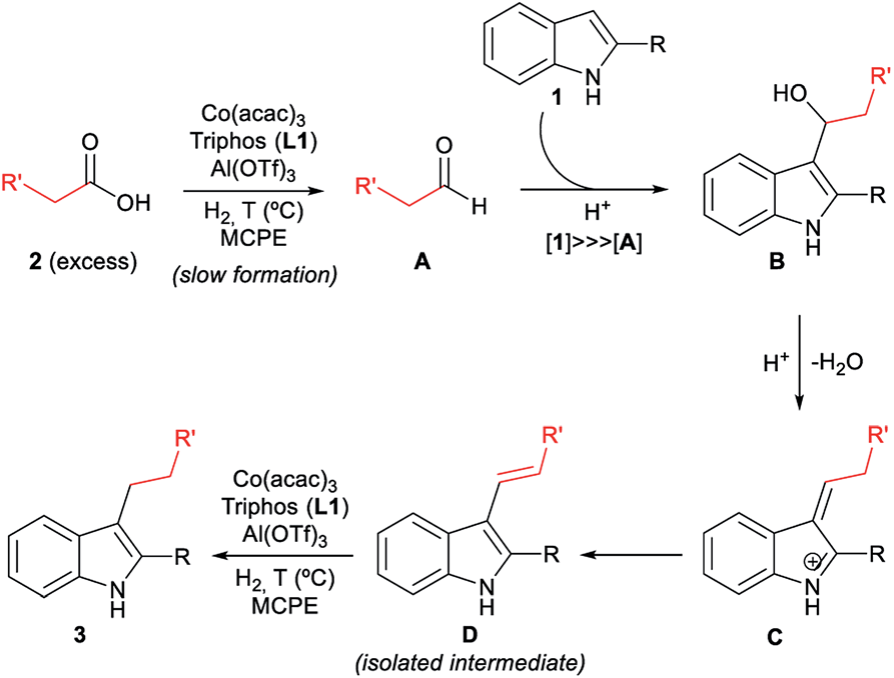
Possible reaction mechanism for the [Co/**L1**/Al(OTf)_3_]-catalysed reductive alkylation of indoles with carboxylic acids (simplified version). The extended version of the overall mechanism containing additional possible minor pathways and secondary transformations is depicted in Scheme S6 (see ESI†).

With all these observations in hand, a plausible mechanism can be proposed for the cobalt catalysed reductive alkylation of indoles (Fig. 5). The major pathway involves in first place the hydrogenation of the carboxylic acid **2** to the corresponding aldehyde **A** or hemiacetal, which would react at the C3 nucleophilic position of indole **1** to afford the alkylated indole **B**. Subsequent dehydration leads to the captured alkene intermediate **D**, that finally gets hydrogenated to afford the alkylated indole. Minor pathways would involve the formation of an ester that can be also hydrogenated to the aldehyde, as well as a dehydrogenation mechanism from the formed alcohol. Scheme S6 (see ESI†) shows the extended version of the reaction mechanism containing additional possible minor pathways and secondary transformations involved in the overall process.

## Conclusions

For the first time, a general reductive C–H alkylation of indoles using carboxylic acids as alkylating agents is presented. This cobalt-catalysed methodology allows functionalization of different indoles at C3 in a straightforward manner. Using the [Co(acac)_3_/Triphos (**L1**)] system in combination with Al(OTf)_3_ as acid co-catalyst a wide range of carboxylic acids can be employed as alkylating agents in the presence of molecular hydrogen. In addition to alkylations, selective alkenylations of some substrates have been successfully performed. Control experiments revealed that the major reaction pathway involves the *in situ* formation of the corresponding aldehyde from the hydrogenation of the carboxylic acid, able to react with the indole. This novel protocol complements previously described reductive alkylations of indoles, mainly employing carbonyl compounds as alkylating agents. Advantageously, the use of carboxylic acids enlarges the potential molecular diversity introduced in the indole scaffold, due to the availability and stability of this class of compounds. Furthermore, the use of a catalyst based on a non-precious metal increases the interest of this transformation.

## Experimental details

### General experimental procedure for the reductive C–H alkylation of 2-methyl-1*H*-indole (**1a**) with acetic acid (**2a**)

A 8 mL glass vial containing a stirring bar was sequentially charged with 2-methyl-1*H*-indole **1a** (67.0 mg, 0.5 mmol), Co(acac)_3_ (3.6 mg, 0.01 mmol, 2 mol%), Triphos **L1** (12.5 mg, 0.02 mmol, 4 mol%, 2 eq. to Co), Al(OTf)_3_ (11.9 mg, 0.025 mmol, 5 mol%, 2.5 eq. to Co), *n*-hexadecane (50.0 mg) as an internal standard, MCPE (2.0 mL) as solvent and acetic acid **2a** (50.3 μL, 0.875 mmol, 1.75 eq.). Afterwards, the reaction vial was capped with a septum equipped with a syringe and set in the alloy plate, which was then placed into a 300 mL autoclave. Once sealed, the autoclave was purged three times with 30 bar of hydrogen, then pressurized to 30 bar and placed into an aluminium block, which was preheated at 160 °C. After 18 h, the autoclave was cooled in an ice bath, and the remaining gas was carefully released. Finally, the reaction mixture was diluted with ethyl acetate and analysed by GC.

### General experimental procedure for the reductive C–H alkylation of indoles with carboxylic acids

A 8 mL glass vial containing a stirring bar was sequentially charged with the indole substrate (0.5 mmol), Co(acac)_3_ (2–6 mol%), Triphos **L1** (4–12 mol%, 2 eq. to Co), Al(OTf)_3_ (5–15 mol%, 2.5 eq. to Co), MCPE (2.0 mL) as solvent and the carboxylic acid (0.875–1.75 mmol, 1.75–3.5 eq.). Afterwards, the reaction vial was capped with a septum equipped with a syringe and set in the alloy plate, which was then placed into a 300 mL autoclave. Once sealed, the autoclave was purged three times with 30–60 bar of hydrogen, then pressurized to 30 bar and placed into an aluminium block, which was preheated at 120–160 °C. After 18–48 h, the autoclave was cooled in an ice bath, and the remaining gas was carefully released. Finally, the reaction mixture was diluted with ethyl acetate and purified by silica gel column chromatography (*n*-heptane/ethyl acetate mixtures) obtaining the desired C3-substituted indole derivatives.
