# Supplementary material for: Cobalt-catalysed reductive C–H alkylation of indoles using carboxylic acids and molecular hydrogen
Source: Chem Sci. 2017 Jul 26;8(9):6439–50. doi: 10.1039/c7sc02117h (PMC5632795; doi:10.1039/c7sc02117h)
Supplement: Supplementary file 1 [file SC-008-C7SC02117H-s001.pdf]

Electronic Supplementary Material (ESI) for Chemical Science.
This journal is © The Royal Society of Chemistry 2017

ELECTRONIC SUPPLEMENTARY INFORMATION

Cobalt-catalyzed Reductive C-H Alkylation of Indoles using Carboxylic Acids and Molecular Hydrogen

Jose R. Cabrero-Antonino,[a] Rosa Adam,[a] Kathrin Junge,[a] and Matthias Beller[a*]

[a]Leibniz Institute für katalyse e. V. an der Universität Rostock,
Albert-Einstein-Straße 29a, 18059 Rostock (Germany);
Phone: +34963877800; Fax: +349638 77809
*Corresponding author: matthias.beller@catalysis.de

1. GENERAL INFORMATION

2. ADDITIONAL EXPERIMENTAL PROCEDURES

3. ADDITIONAL TABLES

Table S1. Study of the solvent effect in the cobalt-catalyzed reductive C-H alkylation of 2-methyl-1*H*-indole **1a** with acetic acid **2a** and molecular hydrogen

4. ADDITIONAL SCHEMES

5. ADDITIONAL FIGURES

6. CHARACTERIZATION DATA OF THE ISOLATED PRODUCTS

7. REFERENCES

8. NMR SPECTRA OF THE ISOLATED PRODUCTS

1. GENERAL INFORMATION

All reagents were obtained commercially from various chemical companies and were used without further purification. Phosphine ligand **L2** was synthetized according previously described methodology.[1] All hydrogenation experiments were carried out in 300 mL autoclave or 100 mL autoclave in the case of kinetic studies (PARR Instrument Company). In order to avoid unspecific reductions, all catalytic reactions were carried out in 8 mL glass vials, which were set in an alloy plate and placed inside the autoclave. In the case of kinetic studies a 100 mL glass inlet was used. GC conversion and yields were determined by GC-FID, HP 6890 with FID detector, column HP530 m x 250 mm x 0.25 μm. Mass spectra were recorded on a GC-MS Agilent 5973 Network equipped with a mass selective detector. All the products were isolated by silica gel column chromatography using as eluent (*n*-heptane / AcOEt) mixtures. ^1H NMR, ^{13}C NMR, ^{19}F NMR and $^{31}P\{^1H\}$ NMR spectra were recorded on a Bruker AV 300 or Bruker AV 400 spectrometer. All chemical shifts (δ) are reported in parts per million (ppm) and coupling constants (*J*) in herzs (Hz). All chemical shifts are reported relative to $CDCl_3$ (deuterated chloroform) peaks (δ 7.26 for ^1H NMR and δ 77.16 for ^{13}C NMR) or CD_3OD (deuterated methanol) peaks (δ 4.87 and 3.31 for ^1H NMR and δ 49.00 for ^{13}C NMR). All measurements were carried out at room temperature unless otherwise stated. HRMS measurements of the all isolated products were performed using the electrospray ionization technique in UPLC (ultra-pressure) equipment.

2. ADDITIONAL EXPERIMENTAL PROCEDURES

General experimental procedure for the reductive C-H alkylation of 2-methyl-1*H*-indole (1a) with acetic acid (2a): A 8 mL glass vial containing a stirring bar was sequentially charged with 2-methyl-1*H*-indole **1a** (67.0 mg, 0.5 mmol), Co(acac)$_3$ (3.6 mg, 0.01 mmol, 2 mol%), Triphos **L1** (12.5 mg, 0.02 mmol, 4 mol%, 2 eq to Co), Al(OTf)$_3$ (11.9 mg, 0.025 mmol, 5 mol%, 2.5 eq to Co), *n*-hexadecane (50.0 mg) as an internal standard, MCPE (2.0 mL) as solvent and acetic acid **2a** (50.3 μL, 0.875 mmol, 1.75 eq). Afterwards, the reaction vial was capped with a septum equipped with a syringe and set in the alloy plate, which was then placed into a 300 mL autoclave. Once sealed, the autoclave was purged three times with 30 bar of hydrogen, then pressurized to 30 bar and placed into an aluminium block, which was preheated at 160 °C. After 18 h, the autoclave was cooled in an ice bath, and the remaining gas was carefully released. Finally, the reaction mixture was diluted with ethyl acetate and analysed by GC.

General experimental procedure for the reductive C-H alkylation of indoles with carboxylic acids: A 8 mL glass vial containing a stirring bar was sequentially charged with the indole substrate (0.5 mmol), Co(acac)$_3$ (2-6 mol%), Triphos **L1** (4-12 mol%, 2 eq to Co), Al(OTf)$_3$ (5-15 mol%, 2.5 eq to Co), MCPE (2.0 mL) as solvent and the carboxylic acid (0.875-1.75 mmol, 1.75-3.5 eq). Afterwards, the reaction vial was capped with a septum equipped with a syringe and set in the alloy plate, which was then placed into a 300 mL autoclave. Once sealed, the autoclave was purged three times with 30-60 bar of hydrogen, then pressurized to 30 bar and placed into an aluminium block, which was preheated at 120-160 °C. After 18-48 h, the autoclave was cooled in an ice bath, and the remaining gas was carefully released. Finally, the reaction mixture was diluted with ethyl acetate and purified by silica gel column chromatography (*n*-heptane / ethyl acetate mixtures) obtaining the desired C3-substituted indole derivatives.

General experimental procedure for the kinetic studies: A 100 mL glass inlet containing a stirring bar was sequentially charged with the 2-methyl-1*H*-indole **1a** (402.0 mg, 3.0 mmol), Co(acac)$_3$ (21.6 mg, 0.06 mmol, 2 mol%), Triphos **L1** (75.0 mg, 0.12 mmol, 4 mol%, 2 eq to Co), Al(OTf)$_3$ (71.4 mg, 0.15 mmol, 5 mol%, 2.5 eq to Co), *n*-hexadecane (250.0 mg) as an internal standard, MCPE (12.0 mL) as solvent and acetic acid **2a** or phenylacetic acid **2p** (5.25 mmol, 1.75 eq). Afterwards, the reaction inlet was then placed into a 100 mL autoclave. Once sealed, the autoclave was purged three times with 30 bar of hydrogen, then pressurized to 30 bar and placed into an aluminium block, which was preheated at 140 or 160 °C. Periodically, aliquots of 200 μL were taken at different times of reaction, diluted with ethyl acetate and analysed by GC.

Experimental procedure for the synthesis of 5-aminophenyl-2-methyl-1*H*-indole (S9) through Buchwald-Hartwig amination[2]: A 50 mL Schlenk containing a stirring bar was sequentially charged under argon with bromobenzene **S7** (268.0 μL, 2.5 mmol), 5-amino-2-methyl-1*H*-indole **S8** (448.8 mg, 3.0 mmol, 1.2 eq), Pd$_2$(dba)$_3$ (22.8 mg, 0.025 mmol, 1 mol%, 2 mol% Pd), XPhos (96.3 mg, 0.2 mmol, 8 mol%), K_2CO_3 (691.3 mg, 5.0 mmol, 2 eq), and dry 1,4-dioxane (15 mL) as solvent. Afterwards, the Schlenk was sealed and set in an oil bath at 100 ºC during 16 h. When the reaction was complete, the crude reaction mixture was allowed to reach room temperature, passed to a funnel extraction, and 20 mL of distillated water were added. The aqueous phase was extracted three times with 20 mL of dichloromethane. The combined organic layers were dried with anhydrous Na_2SO_4 and filtered under gravity. The solvent was removed under reduced pressure. Finally, reaction mixture was purified by silica gel column chromatography (*n*-heptane / ethyl acetate mixtures) obtaining 5-aminophenyl-2-methyl-1*H*-indole **S9** in 56% yield.

General experimental procedure for the synthesis of indole derivatives (S11 and S12) through Suzuki-Miyaura cross coupling reactions[3]: A 250 mL Schlenk containing a stirring bar was sequentially charged under argon with 5-bromo-2-methyl-1*H*-indole **S10** (552.8 mg, 2.5 mmol), $Pd(PPh_3)_4$ (116.0 mg, 0.1 mmol, 4 mol%) and dry toluene (50 mL) as solvent. Then, a solution of boronic acid (3.75 mmol, 1.5 eq) in dry ethanol (15 mL) was added to the reaction followed by saturated 25 mL aqueous $NaHCO_3$. Afterwards, the Schlenk was sealed and set in an oil bath at reflux during 20 h. When the reaction was complete, the crude reaction mixture was allowed to reach room temperature and the solvent was evaporated. Next, 20 mL of distillated water and 30 mL of ethyl acetate were added to the crude and passed to a funnel extraction. The aqueous phase was extracted three times with 30 mL of ethyl acetate. The combined organic layers were dried with anhydrous Na_2SO_4 and filtered under gravity. The solvent was removed under reduced pressure. Finally, reaction mixture was purified by silica gel column chromatography (*n*-heptane / ethyl acetate mixtures) obtaining the corresponding indole derivative **S11** or **S12** in 43% or 70% yield, respectively.

Experimental procedure for the synthesis of (*E*)-2-methyl-3-styryl-1*H*-indole (3p')[4]: A 100 mL Schlenk containing a stirring bar was sequentially charged with 2-methyl-1*H*-indole **1a** (804.0 mg, 6.0 mmol), $FeCl_3 \cdot 6H_2O$ (48.7 mg, 0.18 mmol, 3 mol%), ethanol (780.0 μL, 13.2 mmol, 2.2 eq) as additive and dichloromethane (36 mL) as solvent. Then, phenylacetaldehyde **S13** (735.0 μL, 6.6 mmol, 1.1 eq) was added to the reaction mixture. Afterwards, the Schlenk was sealed and set under agitation at room temperature during 3 h. When the reaction was complete, the solvent was removed under reduced pressure. Finally, reaction mixture was purified by silica gel column chromatography (*n*-heptane / ethyl acetate mixtures) obtaining the corresponding product **3p'** in 70% yield.

3. ADDITIONAL TABLES

Table S1. Study of the solvent effect in the cobalt-catalyzed reductive C-H alkylation of 2-methyl-1*H*-indole **1a** with acetic acid **2a** and molecular hydrogen

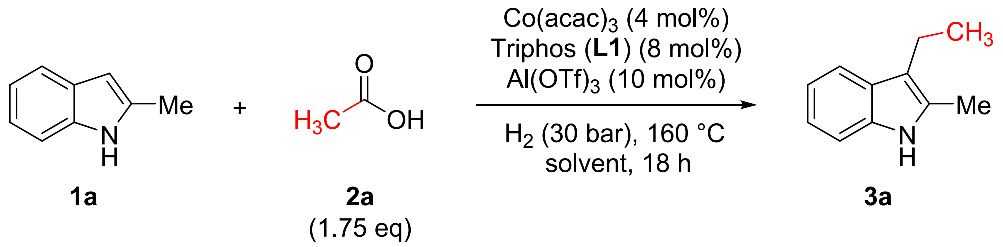

Entry[a]	Solvent	Conv. (%)	**3a** (%)[b]
1	THF	>99	70
2	THF:H_2O (10:1)	48	7
3	1,4-dioxane	>99	72
4	Ethylenglycol diethyl ether	>99	74
5	Tetrahydropirane	>99	81
**6**	**MCPE**	**>99**	**83**
7	2-Me-THF	>99	78
8	*n*-Bu_2O	>99	65
9	Toluene	>99	76
10	DMF	2	-
11	MCPE:H_2O (10:1)	13	2

[a] Standard reaction conditions: 2-methyl-1*H*-indole **1a** (67.0 mg, 0.5 mmol), $Co(acac)_3$ (7.2 mg, 0.02 mmol, 4 mol%), Triphos (**L1**) (25.0 mg, 0.04 mmol, 8 mol%, 2 eq to Co), $Al(OTf)_3$ (23.8 mg, 0.05 mmol, 10 mol%, 2.5 eq to Co), solvent (2 mL), acetic acid **2a** (50.3 μL, 0.875 mmol, 1.75 eq) and H_2 (30 bar) at 160 °C during 18 h. [b] Conversions of **1a** and yields of product **3a** were calculated by GC using hexadecane as internal standard.

4. ADDITIONAL SCHEMES

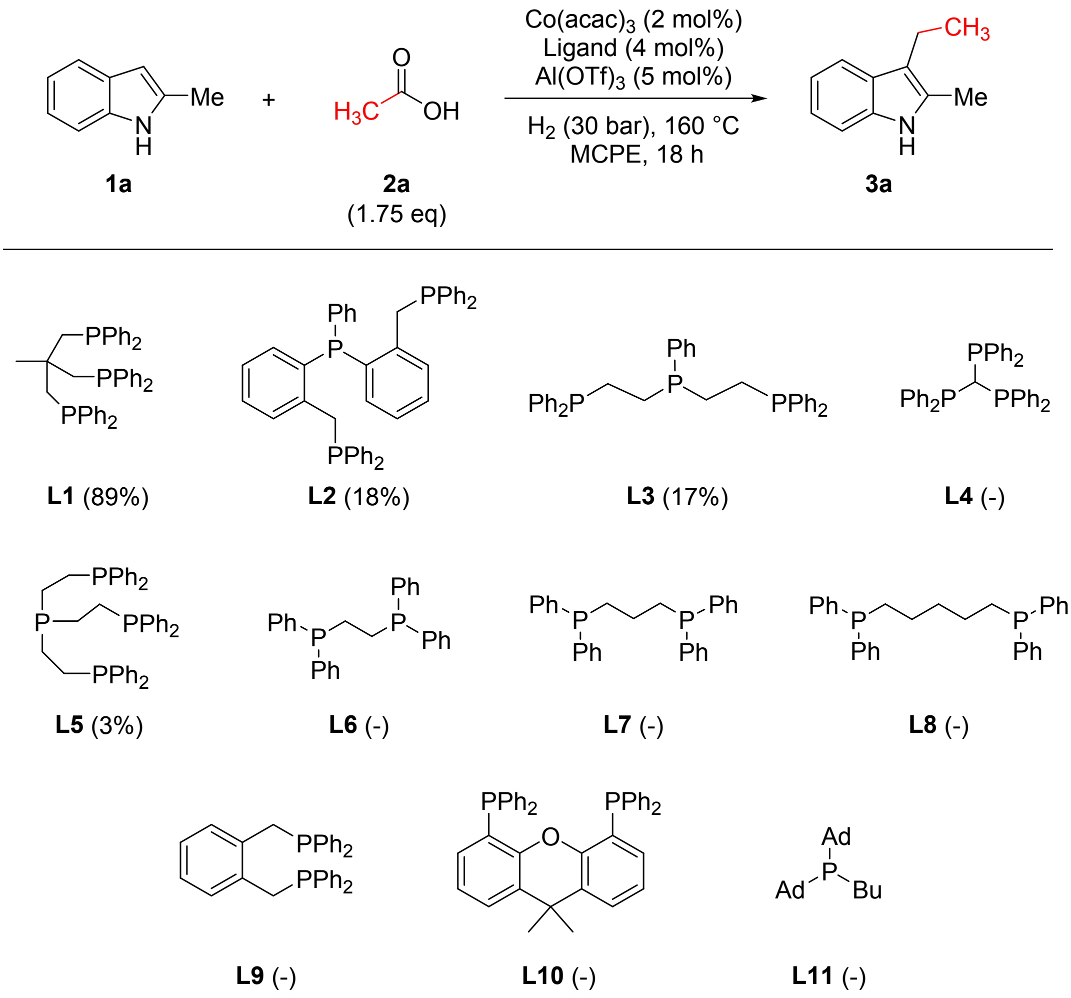

Scheme S1. Influence of the ligand in the formation of product **3a** by the cobalt-catalyzed reductive C-H alkylation of 2-methyl-1*H*-indole **1a** with acetic acid **2a**. Standard reaction conditions: 2-methyl-1*H*-indole **1a** (67.0 mg, 0.5 mmol), $Co(acac)_3$ (3.6 mg, 0.01 mmol, 2 mol%), ligand (0.02 mmol, 4 mol%, 2 eq to Co), $Al(OTf)_3$ (11.9 mg, 0.025 mmol, 5 mol%, 2.5 eq to Co), MCPE (2 mL), acetic acid **2a** (50.3 μL, 0.875 mmol, 1.75 eq) and H_2 (30 bar) at 160 °C during 18 h. In parentheses is shown the yield of product **3a** calculated by GC using hexadecane as internal standard.

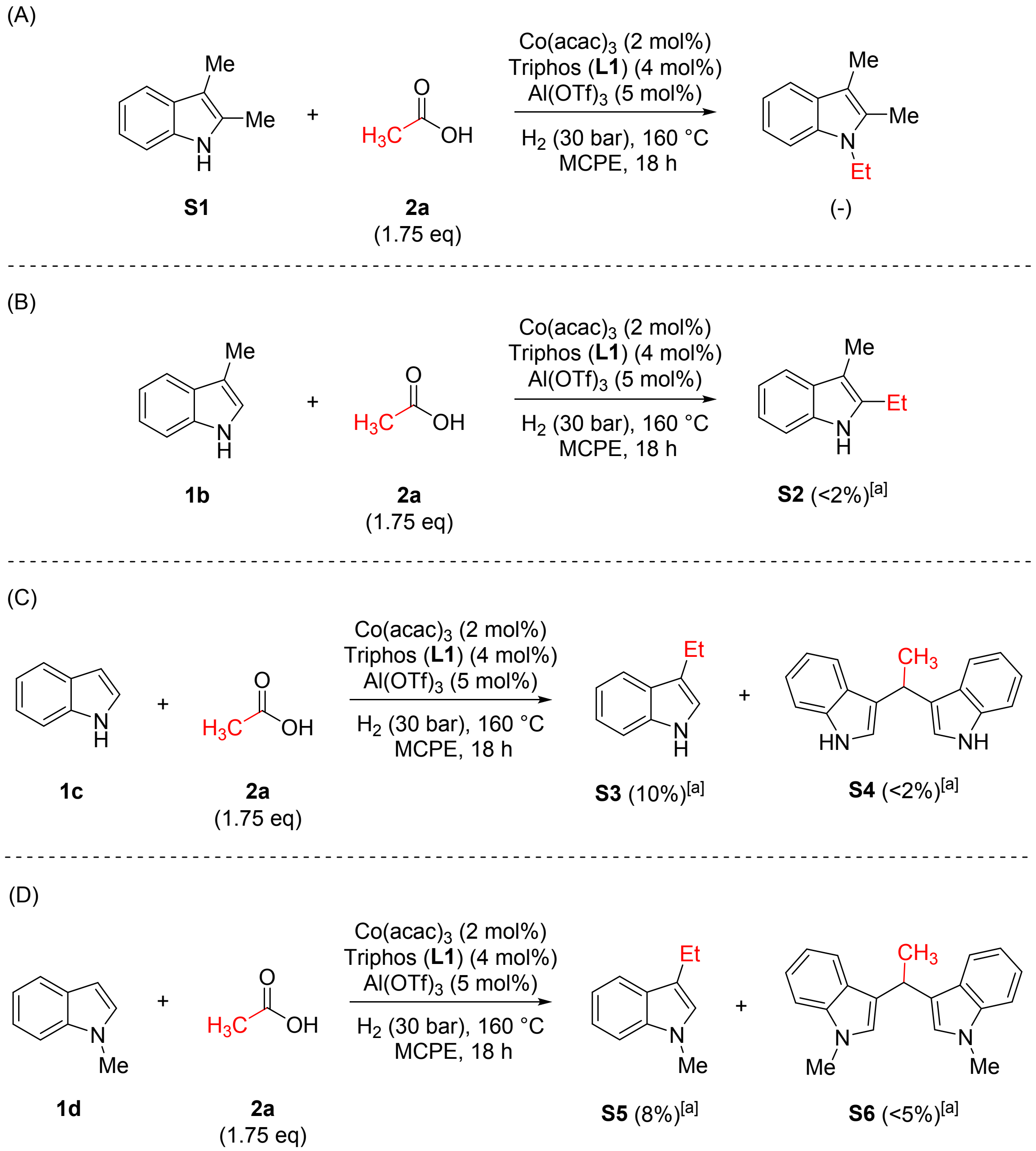

Scheme S2. Catalytic studies for the cobalt-catalyzed reductive C-H alkylation of 2,3-dimethyl-1*H*-indole **S1** (eq. A), 3-methyl-1*H*-indole **1b** (eq. B), 1*H*-indole **1c** (eq. C) and 1-methylindole **1d** (eq. D) with acetic acid **2a**. Standard reaction conditions: indole derivative (0.5 mmol), $Co(acac)_3$ (3.6 mg, 0.01 mmol, 2 mol%), Triphos (**L1**) (12.5 mg, 0.02 mmol, 4 mol%, 2 eq to Co), $Al(OTf)_3$ (11.9 mg, 0.025 mmol, 5 mol%, 2.5 eq to Co), MCPE (2 mL), acetic acid **2a** (50.3 µL, 0.875 mmol, 1.75 eq) and H_2 (30 bar) at 160 °C during 18 h. [a] The yield of product **S2**, **S3**, **S4**, **S5** and **S6** was calculated by GC using hexadecane as internal standard.

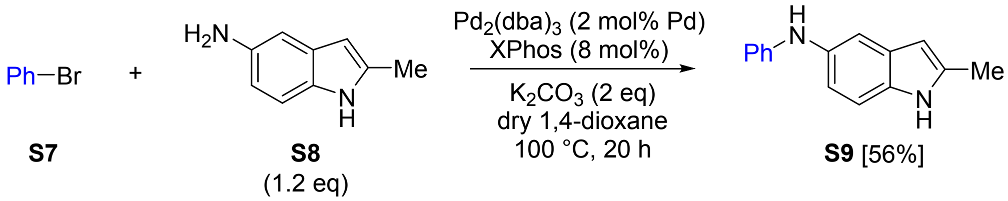

Scheme S3. Synthesis of 5-aminophenyl-2-methyl-1*H*-indole **S9** using Buchwald-Hartwig amination. In brackets is shown the isolated yield of the product after purification by column chromatography on silica using (*n*-heptane/ethyl acetate) mixtures as eluent. Reaction conditions: bromobenzene **S7** (268.0 µL, 2.5 mmol), 2-methyl-1*H*-indol-5-amine **S8** (448.8 mg, 3.0 mmol, 1.2 eq), $Pd_2(dba)_3$ (22.8 mg, 0.025 mmol, 1 mol%, 2 mol% Pd), XPhos (96.3 mg, 0.2 mmol, 8 mol%), K_2CO_3 (691.3 mg, 5.0 mmol, 2 eq), dry 1,4-dioxane (15 mL) at 100 °C during 20 h.

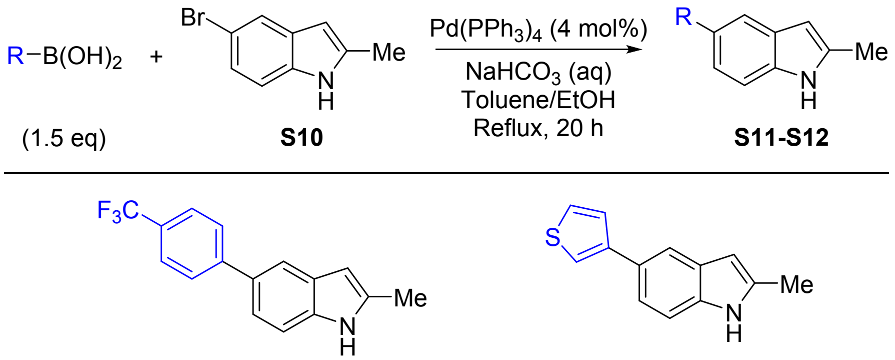

S11 [43%]

S12 [70%]

Scheme S4. Synthesis of **i**ndole derivatives **S11** and **S12** using Suzuki-Miyaura cross-coupling. In brackets is shown the isolated yield of the products after purification by column chromatography on silica using (*n*-heptane/ethyl acetate) mixtures as eluent. Standard reaction conditions: 5-bromo-2-methyl-1*H*-indole **S10** (552.8 mg, 2.5 mmol), boronic acid (3.75 mmol, 1.5 eq), $Pd(PPh_3)_4$ (116.0 mg, 0.1 mmol, 4 mol%), $NaHCO_3$ (25 mL saturated solution in water), dry toluene (50 mL) and dry ethanol (15 mL) at reflux during 20 h.

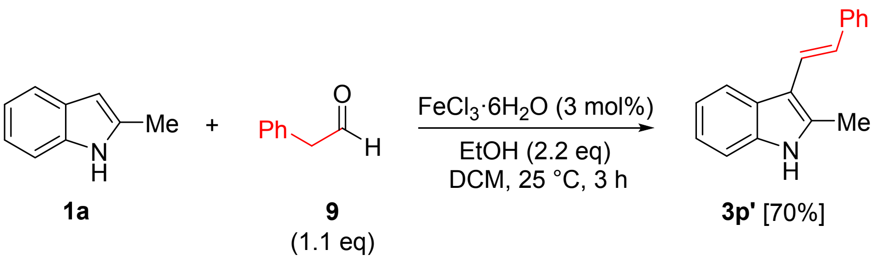

Scheme S5. Synthesis of (*E*)-2-methyl-3-styryl-1*H*-indole **3p'** through iron-catalyzed alkenylation of 2-methyl-1*H*-indole **1a** with phenylacetaldehyde **9**. In brackets is shown the isolated yield of the product after purification by column chromatography on silica using (*n*-heptane/ethyl acetate) mixtures as eluent. Reaction conditions: 2-methyl-1*H*-indole **1a** (804.0 mg, 6.0 mmol), $FeCl_3{\cdot}6H_2O$ (48.7 mg, 0.18 mmol, 3 mol%), ethanol (780.0 mL, 13.2 mmol, 2.2 eq), phenylacetaldehyde **9** (735.0 mL, 6.6 mmol, 1.1 eq) and dichloromethane (36 mL) at room temperature during 3 h.

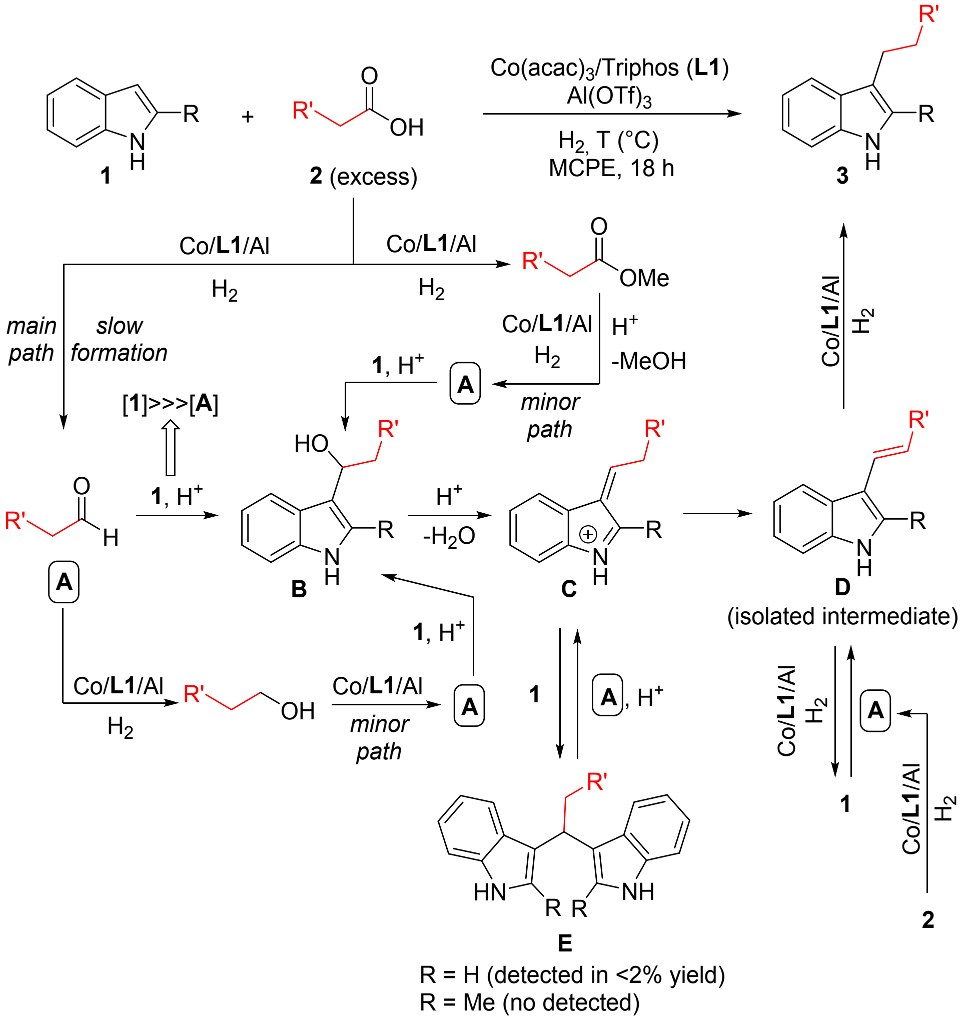

Scheme S6. Possible reaction mechanism for the [Co/**L1**/Al(OTf)$_3$]-catalyzed reductive alkylation of indoles with carboxylic acids. Extended version that contains the minor pathways that are involved in the general process.

5. ADDITIONAL FIGURES

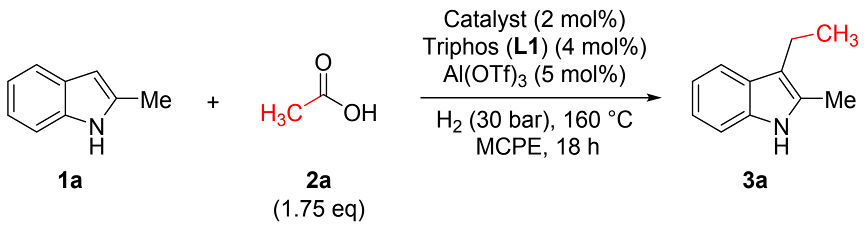

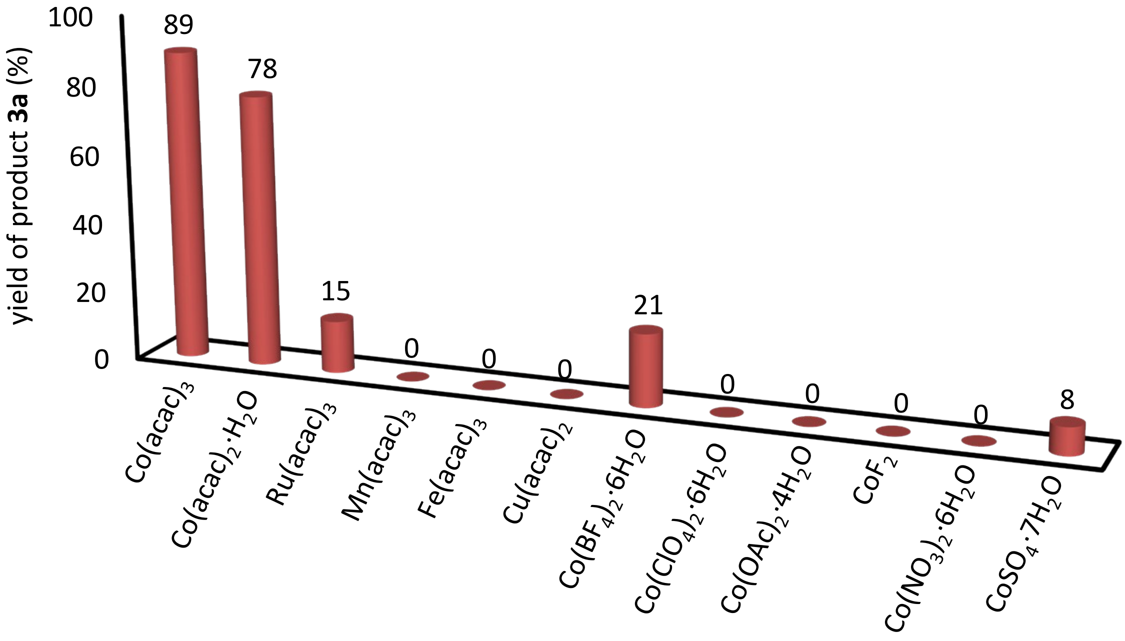

Figure S1. Influence of the metal precatalyst in the formation of product **3a** by the reductive C-H alkylation of 2-methyl-1*H*-indole **1a** with acetic acid **2a**. Standard reaction conditions: 2-methyl-1*H*-indole **1a** (67.0 mg, 0.5 mmol), metal precatalyst (0.01 mmol, 2 mol%), Triphos (**L1**) (12.5 mg, 0.02 mmol, 4 mol%, 2 eq to M), $Al(OTf)_3$ (11.9 mg, 0.025 mmol, 5 mol%, 2.5 eq to M), MCPE (2 mL), acetic acid **2a** (50.3 µL, 0.875 mmol, 1.75 eq) and H_2 (30 bar) at 160 °C during 18 h. Yields of product **3a** were calculated by GC using hexadecane as internal standard.

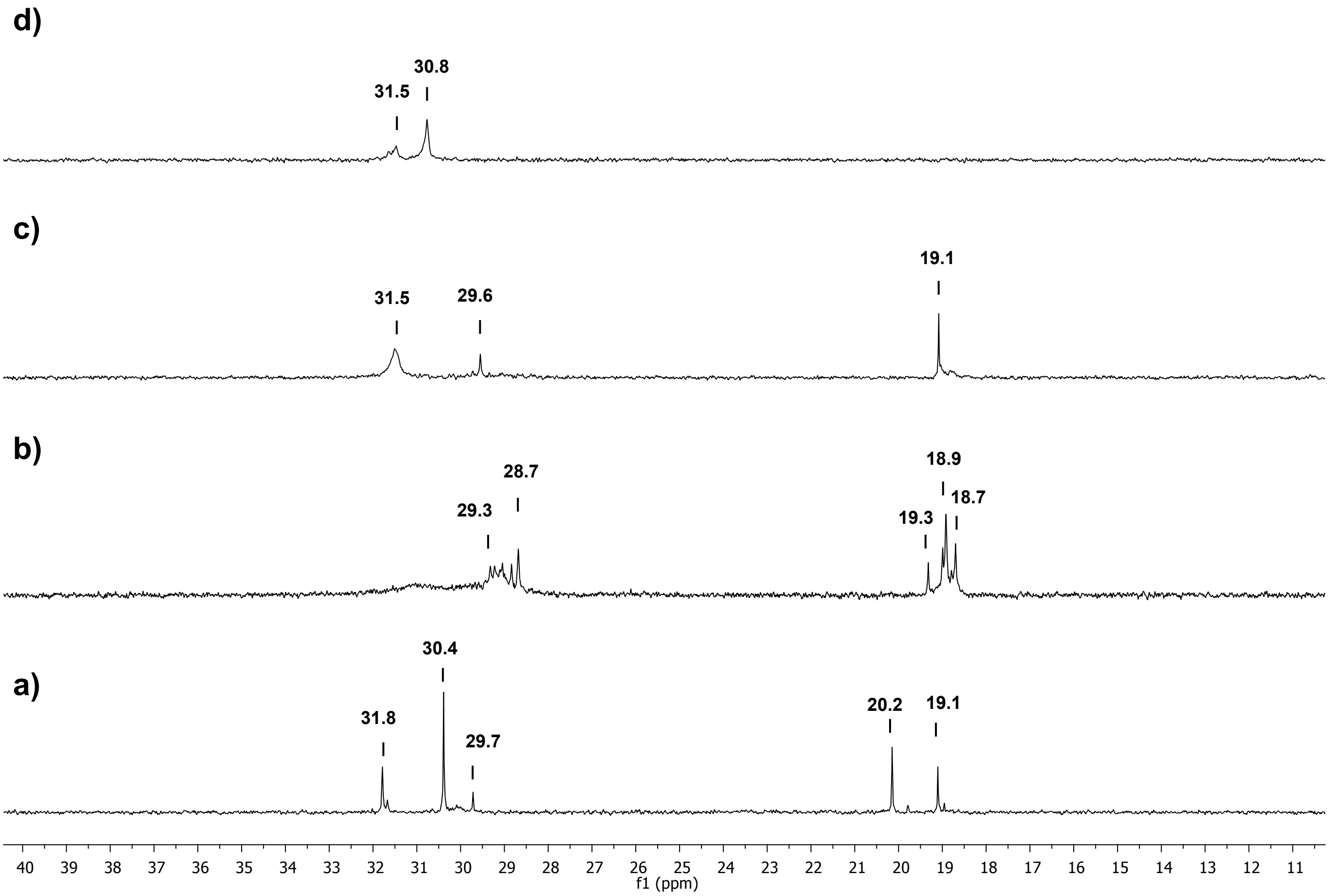

Figure S2. $^{31}P\{^{1}H\}$ NMR (122 MHz, CD_3OD) of: a) Standard reaction mixture: [Co(acac)$_3$ (7.2 mg, 0.02 mmol, 4 mol%), Triphos (**L1**) (25.0 mg, 0.04 mmol, 8 mol%, 2 eq to Co), Al(OTf)$_3$ (23.8 mg, 0.05 mmol, 10 mol%, 2.5 eq to Co), 2-methyl-1*H*-indole **1a** (67.0 mg, 0.5 mmol) and acetic acid **2a** (50.3 μL, 0.875 mmol, 1.75 eq) in MCPE (2mL), at 160 °C, under H_2 (30 bar) during 18 h] after evaporation of MCPE; b) Standard reaction mixture without 2-methyl-1*H*-indole **1a** after evaporation of MCPE; c) Standard reaction mixture without 2-methyl-1*H*-indole **1a** and acetic acid **2a** after evaporation of MCPE; d) Standard reaction mixture without 2-methyl-1*H*-indole **1a**, acetic acid **2a** and Al(OTf)$_3$ after evaporation of MCPE.

6. CHARACTERIZATION DATA OF THE ISOLATED PRODUCTS

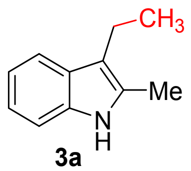

3a

3-ethyl-2-methyl-1*H*-indole (3a).[5] Isolated yield: 80%. GC-MS (m/z, $M^{+\cdot}$ 159), major peaks found: 159 (35%), 144 (100%), 128 (5%), 115 (10%), 77 (8%). (The NMR spectrum is consistent with the reported data). ^{1}H NMR (300 MHz, $CDCl_3$) δ: 7.60-7.34 (m, NH+1H), 7.13-7.09 (m, 1H), 7.04-7.00 (m, 1H), 7.00-6.95 (m, 1H), 2.61 (q, *J* = 7.6, 2H), 2.21 (s, 3H), 1.13 (t, *J* = 7.6, 3H). ^{13}C NMR (75 MHz, $CDCl_3$) δ: 135.38, 130.23, 128.60, 120.91, 119.08, 118.20, 114.08, 110.26, 17.50, 15.56, 11.64. HRMS (ESI) [$M+Na^+$; calculated for $C_{11}H_{13}N$: 159.1042] found m/z 159.1043.

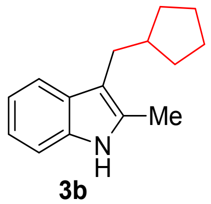

3b

3-(cyclopentylmethyl)-2-methyl-1*H*-indole (3b). Isolated yield: 80%. GC-MS (m/z, $M^{+\cdot}$ 213), major peaks found: 213 (18%), 144 (100%), 128 (4%), 115 (5%), 77 (5%). ^{1}H NMR (300 MHz, $CDCl_3$) δ: 7.48-7.40 (m, 2H), 7.12-7.07 (m, 1H), 7.04-6.94 (m, 2H), 2.58 (d, *J* = 7.3, 2H), 2.21 (s, 3H), 2.16-2.03 (m, 1H), 1.64-1.50 (m, 4H), 1.47-1.35 (m, 2H), 1.22-1.15 (m, 2H). ^{13}C NMR (75 MHz, $CDCl_3$) δ: 135.28, 130.96, 129.20, 120.77, 118.96, 118.43, 112.19, 110.20, 41.90, 32.75, 29.99, 24.98, 11.90. HRMS (ESI) [M^+; calculated for $C_{15}H_{19}N$: 213.1512] found m/z 213.1514.

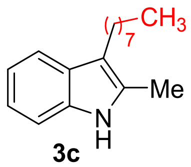

3c

2-methyl-3-octyl-1*H*-indole (3c). Isolated yield: 67%. GC-MS (m/z, $M^{+\cdot}$ 243), major peaks found: 243 (20%), 144 (100%), 130 (5%), 115 (5%). ^{1}H NMR (300 MHz, $CDCl_3$) δ: 7.50 (bs, NH), 7.44-7.39 (m, 1H), 7.16-7.10 (m, 1H), 7.04-7.00 (m, 1H), 7.00-6.94 (m, 1H), 2.58 (t, *J* = 7.4, 2H), 2.23 (s, 3H), 1.52 (quint, *J* = 7.1, 2H), 1.18 (bs, 10H), 0.84-0.74 (m, 3H). ^{13}C NMR (75 MHz, $CDCl_3$) δ: 135.33, 130.67, 128.93, 120.81, 119.00, 118.27, 112.57, 110.22, 32.08, 30.94, 29.80, 29.72, 29.52, 24.27, 22.83, 14.26, 11.75. HRMS (ESI) [M^+; calculated for $C_{17}H_{25}N$: 243.1982] found m/z 243.1988.

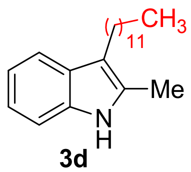

3d

2-methyl-3-dodecyl-1*H*-indole (3d). Isolated yield: 74%. GC-MS (m/z, $M^{+\cdot}$ 299), major peaks found: 299 (15%), 144 (100%), 130 (5%). ^{1}H NMR (300 MHz, $CDCl_3$) δ: 7.47 (bs, NH), 7.43-7.38 (m, 1H), 7.12-7.07 (m, 1H), 7.04-6.99 (m, 1H), 6.99-6.93 (m, 1H), 2.57 (t, *J* = 8.2, 2H), 2.20 (s, 3H), 1.58-1.45 (m, 2H), 1.17 (bs, 18H), 0.80 (t, *J* = 7.4, 3H). ^{13}C NMR (75 MHz, $CDCl_3$) δ: 135.34, 130.67, 128.92, 120.78, 118.98, 118.26, 112.51, 110.24, 32.08, 30.94, 29.86, 29.80, 29.77, 29.52, 24.27, 22.85, 14.27, 11.69. HRMS (ESI) [$M+Na^+$; calculated for $C_{21}H_{33}N$: 299.2607] found m/z 299.2607.

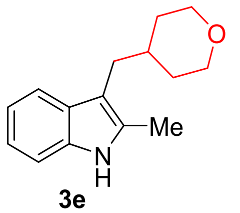

3e

2-methyl-3-((tetrahydro-2*H*-pyran-4-yl)methyl)-1*H*-indole (3e). Isolated yield: 59%. GC-MS (m/z, $M^{+\cdot}$ 229), major peaks found: 229 (18%), 144 (100%), 115 (5%). ^{1}H NMR (300 MHz, $CDCl_3$) δ: 7.79 (bs, NH), 7.42-7.36 (m, 1H), 7.16-7.10 (m, 1H), 7.06-6.95 (m, 2H), 3.86 (dd, *J* = 11.0, 3.8, 2H), 3.22 (td, *J* = 11.9, 2.1, 2H), 2.53 (d, *J* = 7.1, 2H), 2.23 (s, 3H), 1.83-1.65 (m, 1H), 1.51 (dd, *J* = 13.0, 1.9, 2H), 1.37-1.21 (m, 2H). ^{13}C NMR (75 MHz, $CDCl_3$) δ: 135.32, 131.64, 129.10, 120.88, 119.05, 118.22, 110.28, 109.87, 68.20, 36.70, 33.43, 31.64, 11.90. HRMS (ESI) [M^+; calculated for $C_{15}H_{19}ON$: 229.1461] found m/z 229.1464.

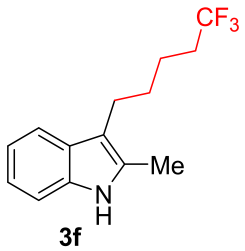

3f

2-methyl-3-(5,5,5-trifluoropentyl)-1*H*-indole (3f). Isolated yield: 74%. GC-MS (m/z, $M^{+\cdot}$ 255), major peaks found: 255 (35%), 144 (100%), 128 (5%), 115 (7%), 77 (7%). ^{1}H NMR (300 MHz, $CDCl_3$) δ: 7.67 (bs, NH), 7.50-7.45 (m, 1H), 7.27-7.23 (m, 1H), 7.15-7.04 (m, 2H), 2.72 (t, *J* = 7.1, 2H), 2.34 (s, 3H), 2.15-2.00 (m, 2H), 1.74-1.67 (m, 2H), 1.66-1.58 (m, 3H). ^{13}C NMR (75 MHz, $CDCl_3$) δ: 135.38, 131.03, 128.69, 127.39 (q, $J^1_{C\text{-}F}$ = 276.4, CF_3), 121.07, 119.22, 118.07, 111.46, 110.35, 33.81 (q, $J^2_{C\text{-}F}$ = 28.3), 29.85, 23.87, 21.84 (q, $J^3_{C\text{-}F}$ = 2.8), 11.74. ^{19}F NMR (282 MHz, $CDCl_3$) δ: -65.89 (t, *J* = 11.0, 3F). HRMS (ESI) [M^+; calculated for $C_{14}H_{16}NF_3$: 255.1229] found m/z 255.1229.

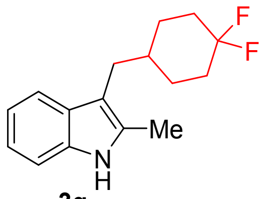

3g

3-((4,4-difluorocyclohexyl)methyl)-2-methyl-1*H*-indole (3g). Isolated yield: 70%. GC-MS (m/z, $M^{+\cdot}$ 263), major peaks found: 263 (18%), 144 (100%), 115 (5%), 77 (5%). ^{1}H NMR (300 MHz, $CDCl_3$) δ: 7.73 (bs, NH), 7.49 (dd, *J* =

6.6, 1.9, 1H), 7.30-7.25 (m, 1H), 7.18-7.06 (m, 2H), 2.64 (d, J = 6.9, 2H), 2.37 (s, 3H), 2.14-2.01 (m, 2H), 1.86-1.74 (m, 3H), 1.73-1.58 (m, 2H), 1.45-1.34 (m, 2H). ^{13}C NMR (75 MHz, $CDCl_3$) δ: 135.32, 131.53, 129.04, 124.14 (dd, $J^1_{C\text{-}F}$ = 241.8, 239.4), 121.06, 119.21, 118.25, 110.47, 110.32, 37.56 (d, $J^4_{C\text{-}F}$ = 1.0), 33.70 (dd, $J^2_{C\text{-}F}$ = 25.3, 22.3), 30.67 (d, $J^5_{C\text{-}F}$ = 2.8), 29.31 (d, $J^3_{C\text{-}F}$ = 9.7), 11.96. ^{19}F NMR (282 MHz, $CDCl_3$) δ: -90.96 (d, J = 234.5, 1F), -99.93-(-103.26) (m, 1F). HRMS (ESI) [M$^+$; calculated for $C_{16}H_{19}NF_2$: 263.1480] found m/z 263.1477.

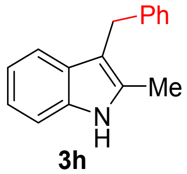

3h

3-benzyl-2-methyl-1*H*-indole (3h).[6] Isolated yield: 75%. GC-MS (m/z, M$^{+\cdot}$ 221), major peaks found: 221 (95%), 206 (40%), 178 (15%), 144 (100%), 128 (5%), 115 (8%), 102 (10%), 77 (8%). (The NMR spectrum is consistent with the reported data). ^{1}H NMR (300 MHz, $CDCl_3$) δ: 7.59 (bs, NH), 7.31 (d, J = 7.8, 1H), 7.18-7.11 (m, 5H), 7.10-7.04 (m, 1H), 7.01 (td, J = 7.5, 1.3, 1H), 6.94 (td, J = 7.5, 1.2, 1H), 3.97 (s, 2H), 2.26 (s, 3H). ^{13}C NMR (75 MHz, $CDCl_3$) δ: 141.77, 135.38, 131.76, 129.00, 128.40, 128.38, 125.78, 121.10, 119.36, 118.49, 110.65, 110.26, 30.22, 11.90. HRMS (ESI) [M$^+$; calculated for $C_{16}H_{15}N$: 221.1199] found m/z 221.1200.

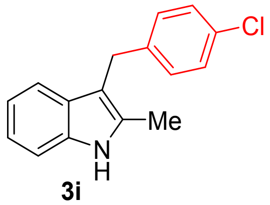

3i

3-(4-chlorobenzyl)-2-methyl-1*H*-indole (3i). Isolated yield: 64%. GC-MS (m/z, M$^{+\cdot}$ 255), major peaks found: 257 (25%), 255 (75%), 242 (10%), 240 (30%), 204 (10%), 144 (100%), 108 (18%). ^{1}H NMR (300 MHz, $CDCl_3$) δ: 7.59 (bs, NH), 7.24 (d, J = 7.8, 1H), 7.17-7.12 (m, 1H), 7.11-7.06 (m, 2H), 7.05-6.98 (m, 3H), 6.97-6.90 (m, 1H), 3.91 (s, 2H), 2.22 (s, 3H). ^{13}C NMR (75 MHz, $CDCl_3$) δ: 140.21, 135.37, 131.85, 131.42, 129.69, 128.77, 128.45, 121.24, 119.47, 118.32, 110.36, 110.10, 29.57, 11.83. HRMS (ESI) [M$^+$; calculated for $C_{16}H_{14}NCl$ (^{37}Cl): 257.0779] found m/z 257.0795 and [M$^+$; calculated for $C_{16}H_{14}NCl$ (^{35}Cl): 255.0809] found m/z 255.0814.

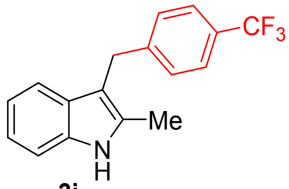

3j

2-methyl-3-(4-(trifluoromethyl)benzyl)-1*H*-indole (3j).[7] Isolated yield: 49%. GC-MS (m/z, M$^{+\cdot}$ 289), major peaks found: 289 (100%), 274 (35%), 144 (100%), 102 (7%). (The NMR spectrum is consistent with the reported data). ^{1}H NMR (300 MHz, $CDCl_3$) δ: 7.79 (bs, NH), 7.52 (d, J = 8.1, 2H), 7.40 (d, J = 7.6, 1H), 7.37-7.28 (m, 3H), 7.21-7.14 (m, 1H), 7.09 (ddd, J = 8.1, 7.1, 1.1, 1H), 4.15 (s, 2H), 2.40 (s, 3H). ^{13}C NMR (75 MHz, $CDCl_3$) δ: 145.90, 135.41, 132.04, 128.73, 128.61, 127.93, 125.33 (q, J = 3.8), 124.53 (q, $J^1_{C\text{-}F}$ = 271.8, CF_3), 121.37, 119.58, 118.24, 110.43,

109.63, 30.07, 11.83. ^{19}F NMR (282 MHz, $CDCl_3$) δ: -61.73 (s, 3F). HRMS (ESI) [M^+; calculated for $C_{17}H_{14}NF_3$: 289.1072] found m/z 289.1066.

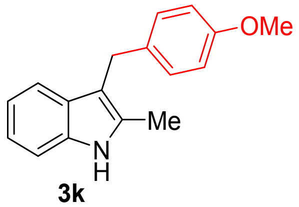

3k

3-(3-methoxybenzyl)-2-methyl-1*H*-indole (3k).[6] Isolated yield: 46%. GC-MS (m/z, $M^{+\cdot}$ 251), major peaks found: 251 (100%), 236 (40%), 220 (8%), 206 (8%), 192 (8%), 144 (95%), 130 (7%). (The NMR spectrum is consistent with the reported data). ^{1}H NMR (300 MHz, $CDCl_3$) δ: 7.68 (s, 1H), 7.32-7.27 (m, 1H), 7.18-7.13 (m, 1H), 7.06-7.01 (m, 2H), 7.01-6.97 (m, 1H), 6.96-6.90 (m, 1H), 6.71-6.65 (m, 2H), 3.91 (s, 2H), 3.65 (s, 3H), 2.26 (s, 3H). ^{13}C NMR (75 MHz, $CDCl_3$) δ: 157.72, 135.39, 133.89, 131.61, 129.22, 128.96, 121.04, 119.29, 118.47, 113.79, 110.97, 110.24, 55.34, 29.30, 11.88. HRMS (ESI) [M^+; calculated for $C_{17}H_{17}ON$: 251.1304] found m/z 251.1310.

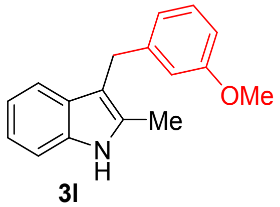

3l

3-(3-methoxybenzyl)-2-methyl-1*H*-indole (3l).[6] Isolated yield: 68%. GC-MS (m/z, $M^{+\cdot}$ 251), major peaks found: 251 (100%), 236 (40%), 220 (8%), 206 (8%), 192 (8%), 144 (95%), 130 (7%). (The NMR spectrum is consistent with the reported data). ^{1}H NMR (300 MHz, $CDCl_3$) δ: 7.53 (bs, NH), 7.30 (d, J = 8.3, 1H), 7.12-7.07 (m, 1H), 7.04 (t, J = 7.9, 1H), 7.01-6.95 (m, 1H), 6.95-6.89 (m, 1H), 6.74-6.66 (m, 2H), 6.59 (dd, J = 8.0, 2.4, 1H), 3.92 (s, 2H), 3.61 (s, 3H), 2.19 (s, 3H). ^{13}C NMR (75 MHz, $CDCl_3$) δ: 159.68, 143.53, 135.35, 131.79, 129.33, 128.94, 121.03, 120.89, 119.31, 118.40, 114.40, 110.80, 110.37, 110.26, 55.16, 30.20, 11.78. HRMS (ESI) [$M+Na^+$; calculated for $C_{17}H_{17}ON$: 251.1304] found m/z 251.1309.

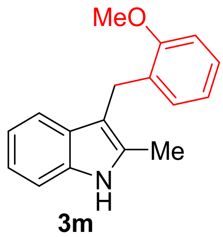

3m

3-(2-methoxybenzyl)-2-methyl-1*H*-indole (3m).[6] Isolated yield: 47%. GC-MS (m/z, $M^{+\cdot}$ 251), major peaks found: 251 (100%), 236 (50%), 220 (20%), 144 (85%), 130 (48%). (The NMR spectrum is consistent with the reported data). ^{1}H NMR (300 MHz, $CDCl_3$) δ: 7.74 (bs, NH), 7.33 (d, J = 7.8, 1H), 7.16 (d, J = 8.5, 1H), 7.09-7.04 (m, 1H), 7.03-6.99 (m, 1H), 6.96 (dd, J = 7.8, 1.2, 1H), 6.94-6.88 (m, 1H), 6.77 (d, J = 8.5, 1H), 6.69 (d, J = 7.4, 1H), 3.94 (s, 2H), 3.79 (s, 3H), 2.25 (s, 3H). ^{13}C NMR (75 MHz, $CDCl_3$) δ: 157.31, 135.41, 132.13, 129.78, 129.39, 129.28, 126.84, 120.88,

120.40, 119.14, 118.67, 110.18, 109.92, 109.78, 55.36, 23.87, 11.83. HRMS (ESI) [M+; calculated for $C_{17}H_{17}ON$: 251.1305] found m/z 251.1323.

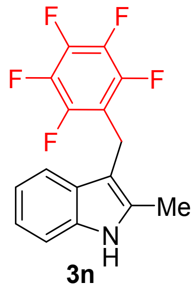

3n

2-methyl-3-((perfluorophenyl)methyl)-1*H*-indole (3n). Isolated yield: 35%. GC-MS (m/z, $M^{+\cdot}$ 311), major peaks found: 311 (100%), 296 (25%), 276 (10%), 219 (10%), 144 (95%), 120 (10%). ^{1}H NMR (300 MHz, $CDCl_3$) δ: 7.63 (bs, NH), 7.47-7.37 (m, 1H), 7.15-7.11 (m, 1H), 7.06-7.01 (m, 1H), 7.01-6.96 (m, 1H), 3.96 (s, 2H), 2.36 (s, 3H). ^{13}C NMR (75 MHz, $CDCl_3$) δ: 147.35-143.17 (m), 141.95-137.56 (m), 139.71-135.53 (m), 135.12, 132.34, 127.97, 121.49, 119.78, 117.79 (t, J^6_{C-F} = 3.2), 115.30-114.29 (m), 110.39, 107.34, 17.48, 11.76 (t, J^6_{C-F} = 2.6). ^{19}F NMR (282 MHz, $CDCl_3$): -142.36 (dd, *J* = 22.5, 8.0, 2F), -157.75 (t, *J* = 20.9, 1F), -162.42 (td, *J* = 22.2, 7.9, 2F). HRMS (ESI) [M+; calculated for $C_{16}H_{10}NF_5$: 311.0728] found m/z 311.0727.

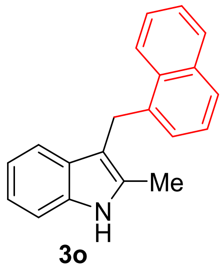

3o

2-methyl-3-(naphthalen-1-ylmethyl)-1H-indole (3o).[7] Isolated yield: 73%. GC-MS (m/z, $M^{+\cdot}$ 271), major peaks found: 271 (100%), 256 (50%), 207 (10%), 144 (100%), 127 (18%). (The NMR spectrum is consistent with the reported data). ^{1}H NMR (300 MHz, $CDCl_3$) δ: 8.38-8.30 (m, 1H), 8.00-7.94 (m, 1H), 7.79 (d, *J* = 8.2, 1H), 7.72 (bs, NH), 7.67-7.55 (m, 2H), 7.43 (d, *J* = 7.8, 1H), 7.40-7.31 (m, 2H), 7.27-7.15 (m, 2H), 7.14-7.07 (m, 1H), 4.60 (s, 2H), 2.32 (s, 3H). ^{13}C NMR (75 MHz, $CDCl_3$) δ: 136.73, 135.43, 133.78, 132.33, 132.30, 129.27, 128.83, 126.61, 125.95, 125.75, 125.60, 125.56, 123.68, 121.11, 119.37, 118.56, 110.32, 109.22, 27.15, 11.85. HRMS (ESI) [M+; calculated for $C_{20}H_{17}N$: 271.1355] found m/z 271.1356.

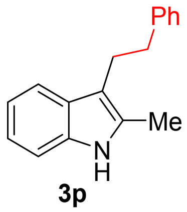

3p

2-methyl-3-phenethyl-1*H*-indole (3p).[8] Isolated yield: 78%. GC-MS (m/z, $M^{+\cdot}$ 235), major peaks found: 235 (15%), 144 (100%), 115 (5%), 91 (7%). (The NMR spectrum is consistent with the reported data). ^{1}H NMR (300 MHz, $CDCl_3$) δ: 7.46-7.41 (m, 1H), 7.36 (bs, NH), 7.17-7.06 (m, 4H), 7.05-6.95 (m, 4H), 2.90-2.83 (m, 2H), 2.82-2.74 (m, 2H), 1.93 (s, 3H). ^{13}C NMR (75 MHz, $CDCl_3$) δ: 142.56, 135.33, 131.34, 128.74, 128.62, 128.29, 125.82, 120.90, 119.13,

118.01, 111.10, 110.32, 37.05, 26.65, 11.31. HRMS (ESI) [M^+; calculated for $C_{17}H_{17}N$: 235.1355] found m/z 235.1358.

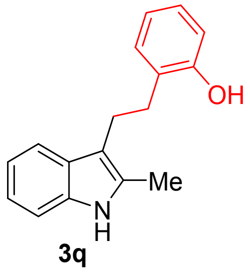

3q

2-(2-(2-methyl-1*H*-indol-3-yl)ethyl)phenol (3q). Isolated yield: 70%. GC-MS (m/z, $M^{+\cdot}$ 251), major peaks found: 251 (15%), 207 (5%), 144 (100%), 115 (5%), 77 (5%). ^1H NMR (300 MHz, $CDCl_3$) δ: 7.58 (bs, NH), 7.49 (dd, *J* = 6.2, 2.7, 1H), 7.17 (dd, *J* = 6.4, 2.4, 1H), 7.08-6.94 (m, 4H), 6.76 (td, *J* = 7.4, 1.2, 1H), 6.61 (dd, *J* = 8.4, 1.1, 1H), 4.50 (bs, OH), 2.96-2.87 (m, 2H), 2.86-2.79 (m, 2H), 1.97 (s, 3H). ^{13}C NMR (75 MHz, $CDCl_3$) δ: 135.43, 131.83, 131.69, 130.76, 128.25, 127.41, 126.21, 121.25, 120.93, 119.42, 117.96, 115.56, 110.90, 110.42, 31.51, 25.00, 11.21. HRMS (ESI) [M^+; calculated for $C_{17}H_{17}ON$: 251.1304] found m/z 251.1307.

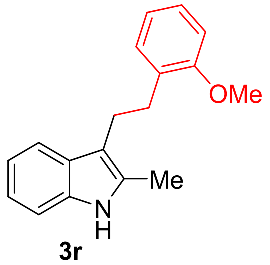

3r

3-(2-methoxyphenethyl)-2-methyl-1*H*-indole (3r). Isolated yield: 55%. GC-MS (m/z, $M^{+\cdot}$ 265), major peaks found: 265 (15%), 144 (100%), 115 (5%), 91 (7%). ^1H NMR (300 MHz, $CDCl_3$) δ: 7.35 (dd, *J* = 6.7, 1.9, 1H), 7.25 (bs, NH), 7.09 (dd, *J* = 6.7, 1.6, 1H), 7.02-6.96 (m, 1H), 6.89 (dd, *J* = 7.0, 1.4, 1H), 6.84 (dd, *J* = 6.7, 1.2, 1H), 6.78 (dd, *J* = 7.3, 1.7, 1H), 6.73 (d, *J* = 8.1, 1H), 6.66-6.60 (m, 1H), 3.64 (s, 3H), 2.78 (dd, *J* = 11.7, 4.3, 2H), 2.71 (dd, *J* = 11.2, 4.9, 2H), 1.98 (s, 3H). ^{13}C NMR (75 MHz, $CDCl_3$) δ: 158.98, 137.06, 132.50, 131.91, 131.22, 128.06, 121.43, 121.25, 121.02, 119.16, 118.55, 111.75, 111.25, 111.11, 55.67, 32.96, 25.81, 11.05. HRMS (ESI) [M^+; calculated for $C_{18}H_{19}ON$: 265.1461] found m/z 265.1462.

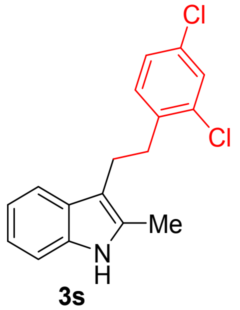

3s

3-(2,4-dichlorophenethyl)-2-methyl-1*H*-indole (3s). Isolated yield: 72%. GC-MS (m/z, $M^{+\cdot}$ 304), major peaks found: 304 (5%), 303 (6%), 159 (5%), 144 (100%). ^1H NMR (300 MHz, $CDCl_3$) δ: 7.58 (bs, NH), 7.49-7.43 (m, 1H),

7.29 (d, *J* = 2.2, 1H), 7.20-7.15 (m, 1H), 7.08-7.00 (m, 1H), 7.03-7.00 (m, 1H), 6.98 (dd, *J* = 8.2, 2.2, 1H), 6.82 (d, *J* = 8.2, 1H), 2.89 (s, 4H), 2.06 (s, 3H). ^{13}C NMR (75 MHz, $CDCl_3$) δ: 138.44, 135.37, 134.65, 132.29, 131.82, 131.54, 129.18, 128.59, 126.88, 121.11, 119.32, 117.98, 110.68, 110.34, 34.42, 24.38, 11.42. HRMS (ESI) [M^+; calculated for $C_{17}H_{15}NCl_2$ (^{35}Cl, ^{35}Cl): 303.0576] found m/z 303.0573 and [M^+; calculated for $C_{17}H_{15}NCl$ (^{37}Cl, ^{35}Cl): 305.0546] found m/z 305.0547.

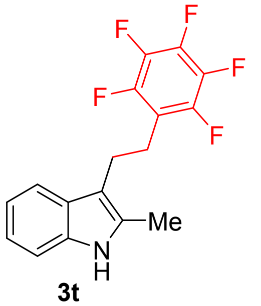

3t

2-methyl-3-(2-(perfluorophenyl)ethyl)-1*H*-indole (3t). Isolated yield: 42%. GC-MS (m/z, $M^{+\cdot}$ 325), major peaks found: 325 (20%), 181 (10%), 144 (100%), 115 (5%). ^{1}H NMR (300 MHz, $CDCl_3$) δ: 7.64 (bs, NH), 7.40 (dd, *J* = 6.3, 2.1, 1H), 7.19-7.15 (m, 1H), 7.07-7.02 (m, 1H), 7.02-6.99 (m, 1H), 2.87 (s, 4H), 2.23 (s, 3H). ^{13}C NMR (75 MHz, $CDCl_3$) δ: 135.30, 131.36, 128.41, 121.28, 119.49, 117.64, 110.38, 110.23, 106.02, 24.18, 23.66, 11.43. Aromatic C-F signals were not well observed. ^{19}F NMR (282 MHz, $CDCl_3$): -144.29 (dd, *J* = 22.6, 8.4, 2F), -157.68 (t, *J* = 20.9, 1F), -162.67 (td, *J* = 21.8, 7.9, 2F). HRMS (ESI) [M^+; calculated for $C_{17}H_{12}NF_5$: 325.0884] found m/z 325.0883.

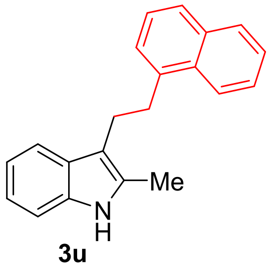

3u

2-methyl-3-(2-(naphthalen-1-yl)ethyl)-1*H*-indole (3u). Isolated yield: 64%. GC-MS (m/z, $M^{+\cdot}$ 285), major peaks found: 285 (15%), 144 (100%), 115 (7%), 77 (4%). ^{1}H NMR (300 MHz, $CDCl_3$) δ: 8.06-8.00 (m, 1H), 7.81-7.75 (m, 1H), 7.63 (d, *J* = 8.2, 1H), 7.56-7.50 (m, 1H), 7.47-7.36 (m, 3H), 7.29-7.23 (m, 1H), 7.18-7.11 (m, 2H), 7.10-7.03 (m, 2H), 3.28 (dd, *J* = 8.9, 6.5, 2H), 3.03 (dd, *J* = 9.0, 6.5, 2H), 1.88 (s, 3H). ^{13}C NMR (75 MHz, $CDCl_3$) δ: 138.57, 135.38, 133.94, 132.04, 128.87, 128.67, 126.65, 126.35, 125.82, 125.67, 125.49, 123.89, 121.00, 119.26, 118.03, 110.38, 34.13, 25.72, 11.34. HRMS (ESI) [M^+; calculated for $C_{21}H_{19}N$: 285.1512] found m/z 285.1510.

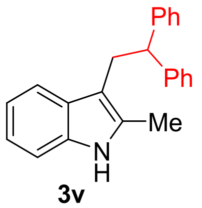

3v

3-(2,2-diphenylethyl)-2-methyl-1*H*-indole (3v). Isolated yield: 70%. GC-MS (m/z, $M^{+\cdot}$ 311), major peaks found: 311 (5%), 165 (7%), 144 (100%). ^{1}H NMR (300 MHz, $CDCl_3$) δ: 7.38-7.32 (m, 1H), 7.23 (bs, NH), 7.16-7.00 (m, 11H), 7.00-6.91 (m, 2H), 4.18 (t, *J* = 7.5, 1H), 3.27 (d, *J* = 7.6, 2H), 1.58 (s, 3H). ^{13}C NMR (75 MHz, $CDCl_3$) δ: 145.19, 135.32, 132.19, 128.81, 128.32, 128.23, 126.12, 120.85, 119.12, 118.00, 110.28, 109.59, 51.63, 31.44, 11.07. HRMS (ESI) [M+Na$^+$; calculated for $C_{23}H_{21}N$: 311.1668] found m/z 311.1666.

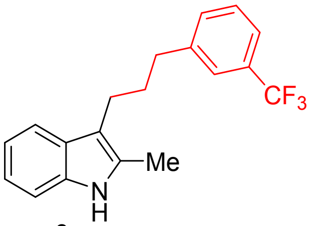

3w

2-methyl-3-(3-(3-(trifluoromethyl)phenyl)propyl)-1*H*-indole (3w). Isolated yield: 72%. GC-MS (m/z, $M^{+\cdot}$ 317), major peaks found: 317 (25%), 157 (5%), 144 (100%), 130 (7%). ^{1}H NMR (300 MHz, $CDCl_3$) δ: 7.62 (bs, NH), 7.41-7.30 (m, 4H), 7.29-7.25 (m, 1H), 7.20-7.15 (m, 1H), 7.06-6.95 (m, 2H), 2.69-2.65 (m, 2H), 2.65-2.60 (m, 2H), 2.24 (s, 3H), 1.97-1.85 (m, 2H). ^{13}C NMR (75 MHz, $CDCl_3$) δ: 143.56, 135.41, 131.94, 130.98, 130.84, 128.82, 128.75, 128.01 (q, $J^1_{C\text{-}F}$ = 272.0, CF_3), 125.21 (q, $J^3_{C\text{-}F}$ = 3.5), 122.66 (q, $J^3_{C\text{-}F}$ = 3.8), 121.05 (s), 119.21, 118.17, 111.59, 110.32, 35.53, 31.98, 23.82, 11.79. ^{19}F NMR (282 MHz, $CDCl_3$) δ: -62.09 (s, 3F). HRMS (ESI) [M$^+$; calculated for $C_{19}H_{18}NF_3$: 317.1385] found m/z 317.1390.

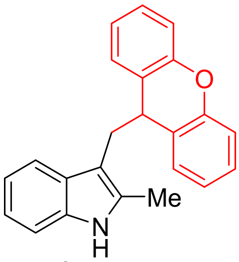

3x

3-((9*H*-xanthen-9-yl)methyl)-2-methyl-1*H*-indole (3x). Isolated yield: 55%. GC-MS (m/z, $M^{+\cdot}$ 325), major peaks found: 325 (5%), 207 (5%), 181 (25%), 144 (100%), 115 (5%). ^{1}H NMR (300 MHz, $CDCl_3$) δ: 7.40 (bs, NH), 7.34-7.29 (m, 1H), 7.14-7.08 (m, 2H), 7.05 (dd, *J* = 5.2, 1.6, 1H), 7.00 (td, *J* = 7.1, 1.3, 2H), 6.97-6.93 (m, 2H), 6.84 (td, *J* = 7.3, 1.3, 2H), 6.77 (dd, *J* = 7.6, 1.7, 2H), 4.03 (t, *J* = 7.1, 1H), 2.86 (d, *J* = 7.1, 2H), 1.43 (s, 3H). ^{13}C NMR (75 MHz, $CDCl_3$) δ: 152.34, 135.23, 133.25, 129.05, 128.73, 127.57, 125.92, 122.96, 120.98, 119.27, 117.82, 116.16, 110.31, 108.34, 40.65, 36.21, 10.67. HRMS (ESI) [M+H$^+$; calculated for $C_{23}H_{19}ON$: 326.1539] found 326.1541.

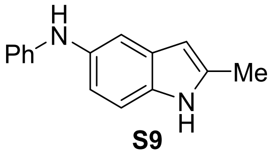

S9

5-aminophenyl-2-methyl-1*H*-indole (S9). Isolated yield: 56%. GC-MS (m/z, $M^{+\cdot}$ 222), major peaks found: 222 (100%), 206 (20%), 180 (5%), 103 (10%). ^{1}H NMR (300 MHz, $CDCl_3$) δ: 7.81 (bs, NH), 7.32 (d, *J* = 2.0, 1H), 7.24-7.18

(m, 3H), 6.98-6.93 (m, 3H), 6.82 (tt, J = 7.4, 1.0, 1H), 6.25-6.08 (m, 1H), 6.00 (bs, NH), 2.44 (s, 3H). ^{13}C NMR (75 MHz, $CDCl_3$) δ: 146.33, 136.15, 134.81, 133.17, 129.91, 129.35, 119.28, 117.28, 115.52, 112.84, 110.89, 100.44, 13.90. HRMS (ESI) [M$^+$; calculated for $C_{15}H_{14}N_2$: 222.1151] found m/z 222.1148.

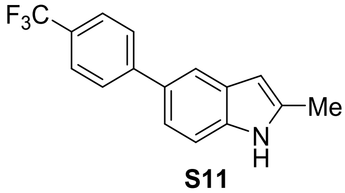

S11

2-methyl-5-(4-(trifluoromethyl)phenyl)-1H-indole (S11). Isolated yield: 43%. GC-MS (m/z, M$^{+\cdot}$ 275), major peaks found: 275 (100%), 256 (5%), 204 (7%), 137 (8%), 127 (8%), 112 (5%), 102 (5%). ^{1}H NMR (300 MHz, $CDCl_3$) δ: 7.91 (bs, NH), 7.77-7.72 (m, 3H), 7.68 (d, J = 8.3, 2H), 7.37-7.36 (m, 2H), 6.30 (s, 1H), 2.48 (s, 3H). ^{13}C NMR (75 MHz, $CDCl_3$): 146.35, 136.35, 136.12, 131.74, 129.79, 128.29 (q, $J^2_{C\text{-}F}$ = 32.2), 127.53, 125.64 (q, $J^3_{C\text{-}F}$ = 3.8), 124.68 (q, $J^1_{C\text{-}F}$ = 271.9), 120.75, 118.63, 110.75, 101.04, 13.93. ^{19}F NMR (282 MHz, $CDCl_3$) δ: -61.74 (s, 3F). HRMS (ESI) [M$^+$; calculated for $C_{16}H_{12}NF_3$: 275.0916] found m/z 213.0912.

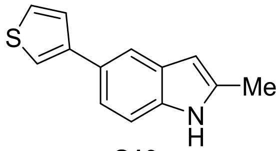

S10

2-methyl-5-(thiophen-3-yl)-1*H*-indole (S12).[3] Isolated yield: 70%. GC-MS (m/z, M$^{+\cdot}$ 213), major peaks found: 213 (100%), 184 (5%), 167 (7%), 106 (5%). (The NMR spectrum is consistent with the reported data). ^{1}H NMR (300 MHz, $CDCl_3$) δ: 7.65 (bs, NH+1H), 7.34 (d, J = 4.3, 1H), 7.31-7.24 (m, 3H), 7.15 (d, J = 8.4, 1H), 6.14 (s, 1H), 2.32 (s, 3H). ^{13}C NMR (75 MHz, $CDCl_3$) δ: 143.90, 135.95, 135.53, 129.58, 128.03, 126.93, 125.80, 120.29, 118.69, 117.58, 110.54, 100.79, 13.87. HRMS (ESI) [M$^+$; calculated for $C_{13}H_{11}NS$: 213.0606] found m/z 213.0606.

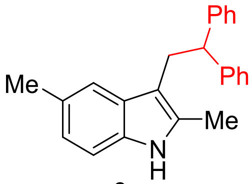

3y

3-(2,2-diphenylethyl)-2,5-dimethyl-1*H*-indole (3y). Isolated yield: 67%. GC-MS (m/z, M$^{+\cdot}$ 325), major peaks found: 325 (5%), 158 (100%), 143 (7%), 115 (5%). ^{1}H NMR (300 MHz, $CDCl_3$) δ: 7.35 (bs, NH), 7.16-7.12 (m, 4H), 7.11-7.06 (m, 6H), 7.02 (d, J = 8.2, 2H), 6.84 (dd, J = 8.2, 1.7, 1H), 4.18 (t, J = 7.5, 1H), 3.27 (d, J = 7.5, 2H), 2.34 (s, 3H), 1.59 (s, 3H). ^{13}C NMR (75 MHz, $CDCl_3$) δ: 145.33, 133.66, 132.34, 129.11, 128.41, 128.26, 128.22, 126.10, 122.32, 117.85, 109.88, 109.22, 51.59, 31.45, 21.67, 11.11. HRMS (ESI) [M$^+$; calculated for $C_{24}H_{23}N$: 325.1825] found m/z 325.1828.

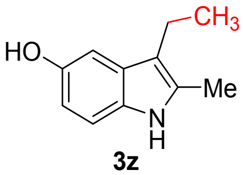

3z

3-ethyl-2-methyl-1*H*-indol-5-ol (3z).[9] Isolated yield: 73%. GC-MS (m/z, $M^{+\cdot}$ 175), major peaks found: 175 (35%), 160 (100%), 146 (5%), 131 (7%), 117 (4%). (The NMR spectrum is consistent with the reported data). ^{1}H NMR (300 MHz, CD_3OD) δ: 7.02 (dd, *J* = 8.5, 0.5, 1H), 6.81 (d, *J* = 2.0, 1H), 6.55 (dd, *J* = 8.5, 2.4, 1H), 2.59 (q, *J* = 7.5, 2H), 2.27 (s, 3H), 1.15 (t, *J* = 7.5, 3H). ^{13}C NMR (75 MHz, CD_3OD) δ: 150.61, 132.50, 132.00, 130.34, 113.25, 111.50, 110.55, 103.18, 18.28, 15.85, 11.29. HRMS (ESI) [M^+; calculated for $C_{11}H_{13}ON$: 175.0991] found m/z 175.0992.

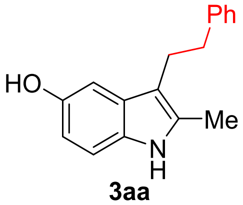

3aa

2-methyl-3-phenethyl-1*H*-indol-5-ol (3aa). Isolated yield: 74%. GC-MS (m/z, $M^{+\cdot}$ 251), major peaks found: 251 (10%), 160 (100%), 91 (5%). ^{1}H NMR (300 MHz, $CDCl_3$) δ: 7.21-7.14 (m, 2H), 7.11 (dt, *J* = 5.7, 2.4, 1H), 7.05-7.01 (m, 3H), 6.86 (d, *J* = 2.3, 1H), 6.57 (dd, *J* = 8.5, 2.3, 1H), 2.94-2.73 (m, 4H), 1.97 (s, 3H). ^{13}C NMR (75 MHz, $CDCl_3$) δ: 150.77, 143.83, 133.76, 132.02, 130.52, 129.68, 129.07, 126.58, 111.57, 110.60, 110.40, 103.10, 37.91, 27.82, 11.14. HRMS (ESI) [M^+; calculated for $C_{17}H_{17}ON$: 251.1304] found m/z 251.1309.

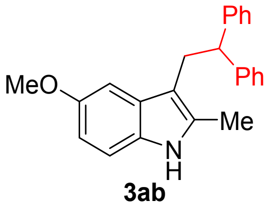

3ab

3-(2,2-diphenylethyl)-5-methoxy-2-methyl-1*H*-indole (3ab). Isolated yield: 67%. GC-MS (m/z, $M^{+\cdot}$ 341), major peaks found: 341 (5%), 174 (100%), 159 (10%), 131 (8%). ^{1}H NMR (300 MHz, $CDCl_3$) δ: 7.29 (bs, NH), 7.17-7.03 (m, 10H), 6.96 (d, *J* = 8.6, 1H), 6.74 (d, *J* = 2.2, 1H), 6.65 (dd, *J* = 8.6, 2.4, 1H), 4.15 (t, *J* = 7.5, 1H), 3.69 (s, 3H), 3.26 (d, *J* = 7.5, 2H), 1.63 (s, 3H). ^{13}C NMR (75 MHz, $CDCl_3$) δ: 153.87, 145.28, 133.15, 130.44, 129.34, 128.37, 128.26, 126.15, 110.84, 110.33, 109.64, 100.60, 56.10, 51.61, 31.50, 11.22. HRMS (ESI) [M^+; calculated for $C_{24}H_{23}ON$: 341.1774] found m/z 341.1775.

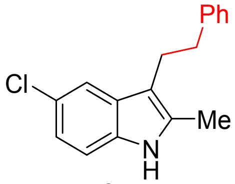

3ac

5-chloro-2-methyl-3-phenethyl-1*H*-indole (3ac). Isolated yield: 56%. GC-MS (m/z, $M^{+\cdot}$ 269), major peaks found: 271 (3%), 269 (9%), 180 (33%), 178 (100%), 143 (9%), 115 (6%), 91 (7%). ^{1}H NMR (300 MHz, $CDCl_3$) δ: 7.68 (bs, NH), 7.46 (d, *J* = 1.9, 1H), 7.27-7.25 (m, 1H), 7.25-7.21 (m, 2H), 7.20-7.15 (m, 2H), 7.14-7.12 (m, 1H), 7.07 (dd, *J* = 8.5, 2.0, 1H), 2.99-2.91 (m, 2H), 2.91-2.84 (m, 2H), 2.07 (s, 3H). ^{13}C NMR (75 MHz, $CDCl_3$) δ: 142.23, 133.68, 133.09, 129.87, 128.78, 128.34, 125.94, 124.89, 121.08, 117.59, 111.20, 111.13, 36.95, 26.56, 11.46. HRMS (ESI) [M-H$^-$; calculated for $C_{17}H_{16}NCl$ (^{37}Cl): 268.0898] found 268.0903 and [M-H$^-$; calculated for $C_{17}H_{16}NCl$ (^{35}Cl): 270.0874] found 270.0881.

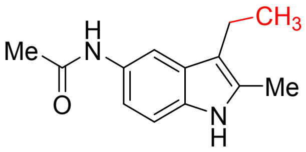

3ad

N-(3-ethyl-2-methyl-1*H*-indol-5-yl)acetamide (3ad). Isolated yield: 38%. GC-MS (m/z, $M^{+\cdot}$ 216), major peaks found: 216 (60%), 201 (100%), 173 (15%), 159 (50%), 130 (10%). ^{1}H NMR (300 MHz, $CDCl_3$) δ: 8.06 (bs, NH), 7.79 (s, 1H), 7.66 (bs, NH), 7.48-7.32 (m, 1H), 7.25 (s, 1H), 2.79 (q, *J* = 7.6, 2H), 2.46 (s, 3H), 2.30 (s, 3H), 1.32 (t, *J* = 7.6, 3H). ^{13}C NMR (75 MHz, $CDCl_3$) δ: 168.69, 132.93, 131.53, 129.76, 128.67, 115.37, 114.14, 110.79, 110.32, 24.51, 17.41, 15.49, 11.65. HRMS (ESI) [M+Na$^+$; calculated for $C_{13}H_{16}ON_2$: 216.1257] found m/z 216.1254.

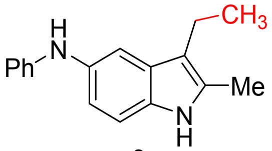

3ae

5-aminophenyl-3-ethyl-2-methyl-1*H*-indole (3ae). Isolated yield: 55%. GC-MS (m/z, $M^{+\cdot}$ 250), major peaks found: 250 (80%), 235 (100%), 220 (8%), 117 (15%), 92 (5%). ^{1}H NMR (300 MHz, $CDCl_3$) δ: 7.62 (bs, NH), 7.34 (d, *J* = 2.0, 1H), 7.26-7.21 (m, 2H), 7.20-7.19 (m, 1H), 6.99-6.91 (m, 3H), 6.81 (tt, *J* = 7.5, 1.1, 1H), 5.63 (bs, NH), 2.68 (q, *J* = 7.5, 2H), 2.37 (s, 3H), 1.22 (t, *J* = 7.5, 3H). ^{13}C NMR (75 MHz, $CDCl_3$) δ: 146.89, 134.27, 132.38, 131.29, 129.35, 129.29, 118.74, 117.44, 114.93, 113.97, 111.82, 110.83, 17.48, 15.53, 11.69. HRMS (ESI) [M$^+$; calculated for $C_{17}H_{18}N_2$: 250.1464] found m/z 250.1469.

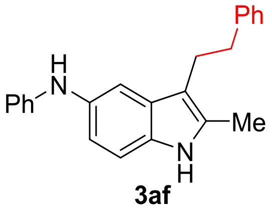

3af

5-aminophenyl-2-methyl-3-phenethyl-1*H*-indole (3af). Isolated yield: 72%. GC-MS (m/z, $M^{+\cdot}$ 326), major peaks found: 326 (25%), 235 (100%), 207 (5%), 117 (5%), 91 (5%). ^{1}H NMR (300 MHz, $CDCl_3$) δ: 7.47 (bs, NH), 7.21 (d, *J* = 2.1, 1H), 7.18-7.06 (m, 6H), 7.05-7.00 (m, 2H), 6.88-6.79 (m, 3H), 6.74-6.67 (m, 1H), 5.50 (bs, NH), 2.87-2.80 (m, 2H), 2.80-2.74 (m, 2H), 1.98 (s, 3H). ^{13}C NMR (75 MHz, $CDCl_3$) δ: 146.80, 142.48, 134.37, 132.37, 132.33,

129.39, 129.36, 128.77, 128.31, 125.84, 118.81, 117.36, 114.97, 111.55, 111.11, 110.86, 37.04, 26.68, 11.47. HRMS (ESI) [M+; calculated for $C_{23}H_{22}N_2$: 326.1779] found m/z 326.1784.

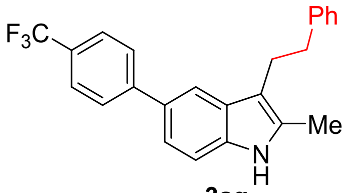

3ag

2-methyl-3-phenethyl-5-(4-(trifluoromethyl)phenyl)-1*H*-indole (3ag). Isolated yield: 70%. GC-MS (m/z, $M^{+\cdot}$ 379), major peaks found: 379 (10%), 288 (100%), 207 (5%), 91 (10%). ^{1}H NMR (300 MHz, $CDCl_3$) δ: 7.78-7.73 (m, NH+2H), 7.72-7.67 (m, 3H), 7.38-7.35 (m, 2H), 7.28-7.26 (m, 1H), 7.26-7.24 (m, 1H), 7.23-7.19 (m, 1H), 7.18-7.12 (m, 2H), 3.08-3.00 (m, 2H), 2.98-2.90 (m, 2H), 2.14 (s, 3H). ^{13}C NMR (75 MHz, $CDCl_3$): 146.51, 142.38, 135.33, 132.59, 131.27, 129.32, 128.84, 128.35, 128.28 (d, J^2_{C-F} = 32.3), 127.61, 125.95, 125.64 (q, J^3_{C-F} = 3.8), 124.69 (q, J^1_{C-F} = 271.7), 120.66, 116.96, 111.81, 110.70, 37.14, 26.57, 11.52. ^{19}F NMR (282 MHz, $CDCl_3$) δ: -61.73 (s, 3F). HRMS (ESI) [M+; calculated for $C_{24}H_{20}NF_3$: 379.1542] found m/z 379.1540.

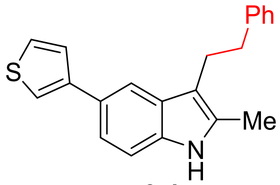

3ah

2-methyl-3-phenethyl-5-(thiophen-3-yl)-1*H*-indole (3ah). Isolated yield: 68%. GC-MS (m/z, $M^{+\cdot}$ 317), major peaks found: 317(15%), 226 (100%), 207 (8%), 91 (8%). ^{1}H NMR (300 MHz, $CDCl_3$) δ: 7.59 (d, *J* = 1.3, 1H), 7.49 (bs, NH), 7.35 (dd, *J* = 4.4, 2.0, 1H), 7.30-7.24 (m, 3H), 7.19-7.08 (m, 4H), 7.06-7.01 (m, 2H), 2.94-2.86 (m, 2H), 2.86-2.77 (m, 2H), 1.96 (s, 3H). ^{13}C NMR (75 MHz, $CDCl_3$) δ: 144.08, 142.46, 134.76, 129.09, 128.81, 128.31, 127.57, 127.04, 125.88, 125.82, 120.24, 118.72, 115.95, 110.51, 37.08, 26.58, 11.44. HRMS (ESI) [M+; calculated for $C_{21}H_{19}NS$: 317.1233] found m/z 317.1239.

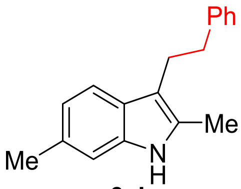

3ai

2,6-dimethyl-3-phenethyl-1*H*-indole (3ai). Isolated yield: 80%. GC-MS (m/z, $M^{+\cdot}$ 249), major peaks found: 249 (18%), 158 (100%), 143 (6%), 115 (6%), 91 (8%). ^{1}H NMR (300 MHz, $CDCl_3$) δ: 7.32 (d, *J* = 7.9, 1H), 7.24 (bs, NH), 7.15-7.10 (m, 2H), 7.10-7.05 (m, 1H), 7.05-7.00 (m, 2H), 6.88-6.80 (m, 2H), 2.88-2.81 (m, 2H), 2.81-2.74 (m, 2H), 2.35 (s, 3H), 1.91 (s, 3H). ^{13}C NMR (75 MHz, $CDCl_3$) δ: 142.63, 135.80, 130.54, 130.52, 128.75, 128.28, 126.47,

125.78, 120.75, 117.70, 110.90, 110.47, 37.08, 26.73, 21.81, 11.28. HRMS (ESI) [M+; calculated for $C_{18}H_{19}N$: 249.1512] found m/z 249.1517.

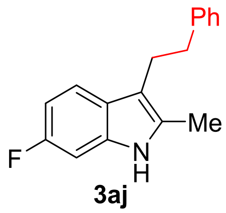

3aj

6-fluoro-2-methyl-3-phenethyl-1*H*-indole (3aj). Isolated yield: 70%. GC-MS (m/z, $M^{+\cdot}$ 253), major peaks found: 253 (15%), 162 (100%), 91 (7%). ^1H NMR (300 MHz, $CDCl_3$) δ: 7.40 (bs, NH), 7.27 (dd, J = 8.5, 5.3, 1H), 7.18-7.06 (m, 3H), 7.03-6.98 (m, 2H), 6.81-6.70 (m, 2H), 2.88-2.80 (m, 2H), 2.80-2.72 (m, 2H), 1.93 (s, 3H). ^{13}C NMR (75 MHz, $CDCl_3$) δ: 159.46 (d, $J^1_{C\text{-}F}$ = 235.7), 142.36, 135.15 (d, $J^3_{C\text{-}F}$ = 12.4), 131.59 (d, $J^4_{C\text{-}F}$ = 3.6), 128.74, 128.33, 125.90, 125.21, 118.50 (d, $J^3_{C\text{-}F}$ = 9.9), 111.07, 107.48 (d, $J^2_{C\text{-}F}$ = 24.2), 96.86 (d, $J^2_{C\text{-}F}$ = 26.0), 36.99, 26.56, 11.32. ^{19}F NMR (282 MHz, $CDCl_3$) δ: -122.34-(-122.46) (m, 1F). HRMS (ESI) [M+; calculated for $C_{17}H_{16}NF$: 253.1261] found m/z 253.1265.

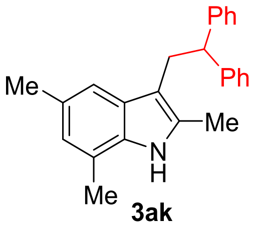

3ak

3-(2,2-diphenylethyl)-2,5,7-trimethyl-1*H*-indole (3ak). Isolated yield: 63%. GC-MS (m/z, $M^{+\cdot}$ 339), major peaks found: 339 (5%), 281 (5%), 253 (4%), 207 (8%), 172 (100%), 157 (8%). ^1H NMR (300 MHz, $CDCl_3$) δ: 7.28 (bs, NH), 7.15 (dt, J = 7.0, 1.5, 2H), 7.13-7.10 (m, 5H), 7.09-7.06 (m, 3H), 7.01 (s, 1H), 6.67 (s, 1H), 4.19 (t, J = 7.5, 1H), 3.26 (d, J = 7.5 Hz, 2H), 2.32 (s, 3H), 2.29 (s, 3H), 1.60 (s, 3H). ^{13}C NMR (75 MHz, $CDCl_3$) δ: 145.38, 133.15, 131.98, 128.67, 128.50, 128.42, 128.21, 126.08, 123.28, 118.96, 115.56, 109.74, 51.62, 31.63, 21.61, 16.66, 11.16. HRMS (ESI) [M+; calculated for $C_{25}H_{25}N$: 339.1981] found m/z 339.1990.

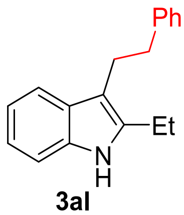

3al

2-ethyl-3-phenethyl-1*H*-indole (3al). Isolated yield: 79%. GC-MS (m/z, $M^{+\cdot}$ 249), major peaks found: 249 (15%), 158 (100%), 143 (20%), 91 (5%). ^1H NMR (300 MHz, $CDCl_3$) δ: 7.64 (bs, NH), 7.49-7.43 (m, 1H), 7.19-7.10 (m, 4H), 7.08-6.99 (m, 4H), 2.95-2.86 (m, 2H), 2.85-2.77 (m, 2H), 2.44 (q, J = 7.6, 2H), 1.02 (t, J = 7.6, 3H). ^{13}C NMR (75 MHz, $CDCl_3$) δ: 142.61, 137.05, 135.38, 128.69, 128.62, 128.33, 125.84, 120.98, 119.14, 118.23, 110.44, 37.33, 26.66, 19.24, 14.20. HRMS (ESI) [M+; calculated for $C_{18}H_{19}N$: 249.1512] found m/z 249.1519.

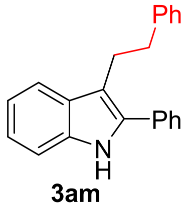

3am

3-phenethyl-2-phenyl-1*H*-indole (3am). Isolated yield: 76%. GC-MS (m/z, $M^{+\cdot}$ 297), major peaks found: 297 (10%), 206 (100%), 178 (10%), 91 (5%). ^{1}H NMR (300 MHz, $CDCl_3$) δ: 7.88 (bs, NH), 7.58 (d, *J* = 8.2, 1H), 7.37-7.23 (m, 6H), 7.20-7.05 (m, 7H), 3.09 (dd, *J* = 10.4, 6.1, 2H), 2.94 (dd, *J* = 9.9, 5.9, 2H). ^{13}C NMR (75 MHz, $CDCl_3$) δ: 142.35, 136.02, 134.67, 133.29, 129.17, 128.90, 128.60, 128.46, 128.06, 127.71, 126.01, 122.38, 119.72, 119.26, 112.90, 110.99, 37.17, 26.96. HRMS (ESI) [M^+; calculated for $C_{22}H_{19}N$: 297.1512] found m/z 297.1519.

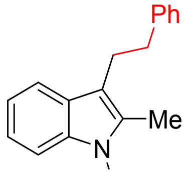

3an

1,2-dimethyl-3-phenethyl-1*H*-indole (3an).[10] Isolated yield: 68%. GC-MS (m/z, $M^{+\cdot}$ 249), major peaks found: 249 (15%), 158 (100%), 143 (12%), 115 (5%), 91 (5%). ^{1}H NMR (300 MHz, $CDCl_3$) δ: 7.46 (d, *J* = 7.5, 1H), 7.19-7.12 (m, 3H), 7.11-7.03 (m, 4H), 7.02-6.96 (m, 1H), 3.48 (s, 3H), 3.01-2.86 (m, 2H), 2.83-2.75 (m, 2H), 2.02 (s, 3H). ^{13}C NMR (75 MHz, $CDCl_3$) δ: 142.64, 136.69, 133.19, 128.72, 128.32, 127.65, 125.83, 120.55, 118.74, 117.96, 110.54, 108.65, 37.50, 29.52, 27.05, 9.98. HRMS (ESI) [M^+; calculated for $C_{18}H_{19}N$: 249.1512] found m/z 249.1530.

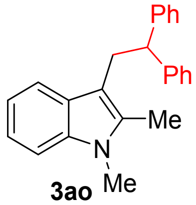

3ao

3-(2,2-diphenylethyl)-1,2-dimethyl-1*H*-indole (3ao). Isolated yield: 59%. GC-MS (m/z, $M^{+\cdot}$ 325), major peaks found: 325 (5%), 281 (5%), 253 (5%), 207 (8%), 158 (100%), 143 (8%), 115 (5%). ^{1}H NMR (300 MHz, $CDCl_3$) δ: 7.38 (d, *J* = 7.7, 1H), 7.18-7.11 (m, 5H), 7.10-7.04 (m, 7H), 6.96 (td, *J* = 7.4, 6.9, 1.2, 1H), 4.18 (t, *J* = 7.5, 1H), 3.43 (s, 3H), 3.33 (d, *J* = 7.5, 2H), 1.65 (s, 3H). ^{13}C NMR (75 MHz, $CDCl_3$) δ: 145.20, 136.68, 134.05, 128.30, 128.15, 127.81, 126.03, 120.35, 118.61, 117.91, 108.90, 108.50, 51.90, 31.74, 29.47, 9.58. HRMS (ESI) [$M+H^+$; calculated for $C_{24}H_{23}N$: 326.1903] found 326.1906.

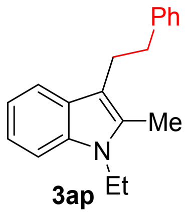

3ap

1-ethyl-2-methyl-3-phenethyl-1*H*-indole (3ap). Isolated yield: 49%. GC-MS (m/z, $M^{+\cdot}$ 263), major peaks found: 263 (10%), 172 (100%), 144 (11%), 115 (5%), 91 (5%). ^{1}H NMR (300 MHz, $CDCl_3$) δ: ^{1}H NMR (300 MHz, Chloroform-*d*) δ 7.48 (d, *J* = 7.3, 1H), 7.20-7.12 (m, 3H), 7.12-7.06 (m, 2H), 7.05-6.97 (m, 3H), 3.97 (q, *J* = 7.2, 2H), 2.95-2.87 (m, 2H), 2.83-2.75 (m, 2H), 1.98 (s, 3H), 1.18 (t, *J* = 7.2, 3H). ^{13}C NMR (75 MHz, $CDCl_3$) δ: 142.63, 135.58, 132.48, 128.78, 128.26, 127.85, 125.79, 120.48, 118.66, 118.05, 110.60, 108.71, 37.77, 37.41, 27.03, 15.45, 9.73. HRMS (ESI) [M^+; calculated for $C_{19}H_{21}N$: 263.1669] found m/z 263.1674.

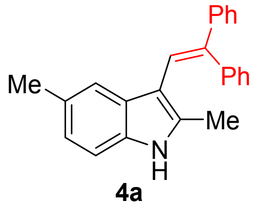

4a

3-(2,2-diphenylvinyl)-2,5-dimethyl-1*H*-indole (4a). Isolated yield: 70%. GC-MS (m/z, $M^{+\cdot}$ 323), major peaks found: 323 (100%), 308 (10%), 293 (8%), 246 (6%), 231 (10%), 217 (9%), 146 (6%). ^{1}H NMR (300 MHz, $CDCl_3$) δ: 7.37 (bs, NH), 7.32-7.26 (m, 2H), 7.26-7.17 (m, 3H), 7.13-7.03 (m, 5H), 6.95-6.86 (m, 2H), 6.81-6.76 (m, 2H), 2.18 (s, 3H), 1.78 (s, 3H). ^{13}C NMR (75 MHz, $CDCl_3$) δ: 144.53, 141.86, 140.80, 133.60, 133.51, 130.92, 128.78, 128.35, 128.25, 128.17, 127.11, 126.65, 122.71, 121.55, 119.51, 110.97, 109.72, 21.48, 12.85. HRMS (ESI) [M^+; calculated for $C_{24}H_{21}N$: 323.1668] found m/z 323.1665.

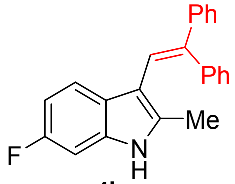

4b

3-(2,2-diphenylvinyl)-6-fluoro-2-methyl-1*H*-indole (4b). Isolated yield: 88%. GC-MS (m/z, $M^{+\cdot}$ 327), major peaks found: 327 (100%), 312 (20%), 250 (8%), 235 (18%), 148 (8%). ^{1}H NMR (300 MHz, $CDCl_3$) δ: 7.70 (bs, NH), 7.46-7.31 (m, 5H), 7.25-7.15 (m, 5H), 7.03-6.94 (m, 2H), 6.89 (dd, *J* = 9.5, 2.3, 1H), 6.69 (ddd, *J* = 9.7, 8.8, 2.3, 1H), 2.00 (s, 3H). ^{13}C NMR (75 MHz, $CDCl_3$) δ: 159.52 (d, $J^1_{C\text{-}F}$ = 236.7), 144.33, 141.55, 135.17 (d, $J^3_{C\text{-}F}$ = 12.4), 133.78 (d, $J^4_{C\text{-}F}$ = 3.2), 130.81, 128.41, 128.25, 128.22, 127.32, 126.92, 125.05, 124.36, 120.89, 120.22 (d, $J^3_{C\text{-}F}$ = 9.9), 111.44, 107.99 (d, $J^2_{C\text{-}F}$ = 23.8), 96.68 (d, $J^2_{C\text{-}F}$ = 26.1), 12.82. ^{19}F NMR (282 MHz, $CDCl_3$) δ: -121.15-(-122.53) (m, 1F). HRMS (ESI) [M^+; calculated for $C_{23}H_{18}NF$: 327.1417] found m/z 327.1425.

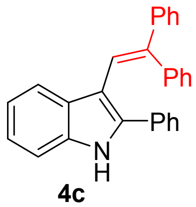

4c

3-(2,2-diphenylvinyl)-2-phenyl-1*H*-indole (4c).[11] Isolated yield: 80%. GC-MS (m/z, $M^{+\cdot}$ 371), major peaks found: 371 (100%), 294 (15%), 204 (8%), 146 (10%). (The NMR spectrum is consistent with the reported data). ^{1}H NMR (300 MHz, $CDCl_3$) δ: 7.88 (bs, NH), 7.42 (dd, *J* = 8.1, 1.3, 2H), 7.31-7.15 (m, 8H), 7.11 (d, *J* = 8.1, 1H), 7.08-7.02 (m, 2H), 7.00-6.84 (m, 6H), 6.77-6.68 (m, 1H). ^{13}C NMR (75 MHz, $CDCl_3$) δ: 144.15, 142.62, 141.16, 136.74, 135.97, 132.85, 130.57, 128.87, 128.49, 128.16, 127.94, 127.90, 127.79, 127.27, 127.21, 126.88, 122.39, 121.58, 120.98, 119.94, 112.32, 110.65. HRMS (ESI) [M^+; calculated for $C_{28}H_{21}N$: 371.1668] found m/z 371.1681.

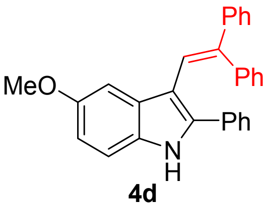

4d

3-(2,2-diphenylvinyl)-5-methoxy-2-phenyl-1*H*-indole (4d). Isolated yield: 68%. GC-MS (m/z, $M^{+\cdot}$ 401), major peaks found: 401 (5%), 167 (100%), 152 (10%), 69 (12%). ^{1}H NMR (300 MHz, $CDCl_3$) δ: 7.68 (dd, *J* = 8.3, 1.3, 2H), 7.51-7.44 (m, 4H), 7.43-7.38 (m, 4H), 7.37-7.32 (m, 3H), 7.26-7.18 (m, 4H), 7. 08 (bs, NH), 6.80 (dd, *J* = 8.7, 2.5, 1H), 6.43 (d, *J* = 2.4, 1H), 3.56 (s, 3H). ^{13}C NMR (75 MHz, $CDCl_3$) δ: 154.00, 144.35, 141.57, 141.54, 137.97, 132.90, 131.04, 130.62, 128.93, 128.61, 128.18, 128.14, 127.95, 127.82, 127.22, 127.09, 127.02, 122.12, 113.18, 112.22, 111.42, 102.35, 55.64. HRMS (ESI) [M^+; calculated for $C_{29}H_{23}ON$: 401.1774] found m/z 401.1769.

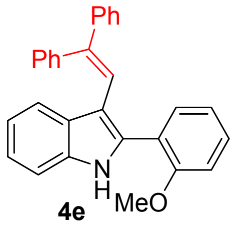

4e

3-(2,2-diphenylvinyl)-2-(2-methoxyphenyl)-1*H*-indole (4e). Isolated yield: 63%. GC-MS (m/z, $M^{+\cdot}$ 401), major peaks found: 401 (100%), 386 (5%), 341 (5%), 309 (7%), 281 (9%), 253 (5%), 207 (15%), 165 (5%). ^{1}H NMR (300 MHz, $CDCl_3$) δ: 9.18 (bs, NH), 7.98 (dd, *J* = 7.6, 1.6, 1H), 7.65-7.60 (m, 2H), 7.58-7.51 (m, 4H), 7.51-7.46 (m, 1H), 7.40-7.34 (m, 3H), 7.33-7.27 (m, 5H), 7.21 (td, *J* = 7.5, 1.1, 1H), 7.15 (d, *J* = 8.3, 1H), 7.11-7.05 (m, 1H), 4.06 (s, 3H). ^{13}C NMR (75 MHz, $CDCl_3$) δ: 156.44, 144.39, 142.02, 141.26, 135.57, 134.13, 131.91, 130.47, 129.09, 128.49, 128.12, 127.87, 127.14, 126.60, 126.49, 122.28, 122.09, 121.28, 121.24, 120.60, 119.45, 112.74, 111.56, 110.49, 55.84. HRMS (ESI) [M^+; calculated for $C_{29}H_{23}ON$: 401.1774] found m/z 401.1768.

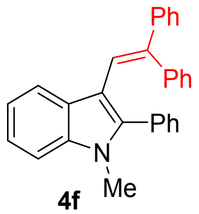

4f

3-(2,2-diphenylvinyl)-2-phenyl-1*H*-indole (4f). Isolated yield: 70%. GC-MS (m/z, $M^{+\cdot}$ 385), major peaks found: 385 (100%), 369 (8%), 307 (10%), 291 (15%), 218 (7%), 193 (6%), 146 (7%). ^{1}H NMR (300 MHz, $CDCl_3$) δ: 7.28-7.11 (m, 12H), 7.06 (dd, *J* = 6.9, 1.1, 1H), 6.97 (s, 4H), 6.90 (d, *J* = 8.0, 1H), 6.81-6.77 (m, 1H), 6.76 (s, 1H), 3.47 (s, 3H). ^{13}C NMR (75 MHz, $CDCl_3$) δ: 144.55, 141.55, 141.17, 140.45, 137.58, 131.77, 130.75, 130.70, 128.41, 128.38, 128.02, 127.87, 126.96, 126.58, 126.14, 121.86, 121.71, 120.87, 119.76, 112.55, 109.27, 31.22. HRMS (ESI) [M^+; calculated for $C_{29}H_{23}N$: 385.1825] found m/z 385.1832.

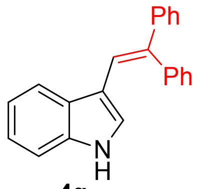

4g

3-(2,2-diphenylvinyl)-1*H*-indole (4g). Isolated yield: 58%. GC-MS (m/z, $M^{+\cdot}$ 295), major peaks found: 295 (100%), 280 (5%), 217 (15%), 189 (5%), 146 (6%). ^{1}H NMR (300 MHz, $CDCl_3$) δ: 7.68-7.59 (m, NH+1H), 7.36-7.30 (m, 2H), 7.30-7.25 (m, 3H), 7.25-7.22 (m, 3H), 7.21-7.18 (m, 2H), 7.17-7.12 (m, 1H), 7.12-7.09 (m, 1H), 7.08 (d, *J* = 2.2, 1H), 7.06-7.01 (m, 1H), 6.03 (d, *J* = 2.4, 1H). ^{13}C NMR (75 MHz, $CDCl_3$) δ: 142.98, 142.06, 138.28, 135.19, 130.17, 129.26, 128.36, 127.74, 127.33, 126.85, 126.80, 123.31, 122.50, 120.16, 118.70, 118.59, 113.98, 111.16. HRMS (ESI) [M^+; calculated for $C_{22}H_{17}N$: 295.1355] found m/z 295.1357.

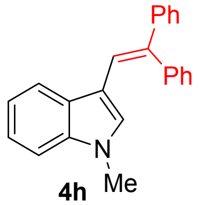

4h

3-(2,2-diphenylvinyl)-1-methyl-1*H*-indole (4h). Isolated yield: 67%. GC-MS (m/z, $M^{+\cdot}$ 309), major peaks found: 309 (100%), 293 (12%), 231 (5%), 217 (6%), 146 (6%), 217 (9%), 146 (6%). ^{1}H NMR (300 MHz, $CDCl_3$) δ: 7.64 (dt, *J* = 7.7, 1.0, 1H), 7.39-7.34 (m, 1H), 7.34-7.28 (m, 4H), 7.26-7.20 (m, 5H), 7.20-7.14 (m, 1H), 7.14-7.09 (m, 3H), 7.05 (ddd, *J* = 8.1, 4.8, 3.4, 1H), 5.90 (s, 1H), 3.42 (s, 3H). ^{13}C NMR (75 MHz, $CDCl_3$) δ: 143.09, 142.20, 137.32, 136.21, 130.17, 129.33, 128.45, 128.34, 127.97, 127.35, 126.70, 126.67, 122.11, 119.79, 118.79, 118.61, 112.58, 109.34, 32.97. HRMS (ESI) [M^+; calculated for $C_{23}H_{19}N$: 309.1512] found m/z 309.1521.

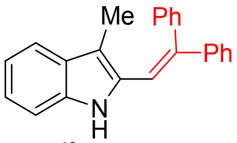

4i

2-(2,2-diphenylvinyl)-3-methyl-1*H*-indole (4i). Isolated yield: 54%. GC-MS (m/z, $M^{+\cdot}$ 309), major peaks found: 309 (100%), 294 (20%), 217 (18%), 146 (10%), 73 (8%). ^{1}H NMR (300 MHz, $CDCl_3$) δ: 7.41-7.35 (m, NH+3H), 7.30-7.23 (m, 5H), 7.23-7.20 (m, 1H), 7.20-7.14 (m, 1H), 7.04 (s, 1H), 7.00-6.87 (m, 3H), 6.78 (dd, *J* = 6.9, 1.3, 1H), 2.32 (s, 3H). ^{13}C NMR (75 MHz, $CDCl_3$) δ: 142.56, 140.29, 138.12, 135.95, 131.87, 130.48, 129.36, 128.47, 128.38, 128.32, 127.49, 127.16, 123.05, 119.31, 118.83, 116.00, 114.23, 110.46, 9.07. HRMS (ESI) [M^+; calculated for $C_{23}H_{19}N$: 309.1512] found m/z 309.1518.

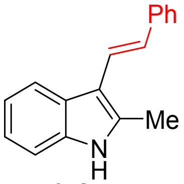

3p'

(*E*)-2-methyl-3-styryl-1*H*-indole (3p').[4] Isolated yield: 72%. GC-MS (m/z, $M^{+\cdot}$ 233), major peaks found: 233 (100%), 218 (45%), 189 (7%), 154 (5%), 130 (7%), 109 (10%). (The NMR spectrum is consistent with the reported data). ^{1}H NMR (300 MHz, $CDCl_3$) δ: 7.86 (dd, *J* = 5.7, 2.1, 1H), 7.60 (bs, NH), 7.45-7.40 (m, 2H), 7.26 (t, *J* = 7.6, 2H), 7.18 (d, *J* = 16.5, 1H), 7.14-7.05 (m, 4H), 7.00 (d, *J* = 16.5, 1H), 2.33 (s, 3H). ^{13}C NMR (75 MHz, $CDCl_3$) δ: 139.10, 135.60, 134.77, 128.75, 126.62, 126.54, 125.76, 125.26, 121.83, 121.76, 120.50, 119.77, 111.02, 110.72, 12.41. HRMS (ESI) [M^+; calculated for $C_{17}H_{15}N$: 233.1199] found m/z 233.1198.

7. REFERENCES

[1] Dutta Chowdhury, A.; Jackstell, R.; Beller, M. *ChemCatChem* **2014**, *6*, 3360

[2] Yeung, S. Y.; Kampmann, S.; Stubbs, K. A.; Skelton, B. W.; Kaskow, B. J.; Abraham, L. J. Stewart, S. G. *Med. Chem. Commun.* **2011**, *2*, 1073

[3] Mattson, C.; Svensson, P.; Boettcher, H.; Sonesson, C. *Eur. J. Med. Chem.* **2013**, *63*, 578

[4] Yang, Q.; Wang, L.; Guo, T.; Yu, Z. *J. Org. Chem.* **2012**, *77*, 8355

[5] Tursky, M.; Lorentz-Petersen, L. L. R.; Olsen, L. B.; Madsen, R. *Org. Biomol. Chem.* **2010**, *8*, 5576

[6] Cao, L-L.; Wang, D.-S.; Jiang, G.-F.; Zhou, Y.-G. *Tetrahedron Lett.* **2011**, *52*, 2837

[7] Panther, J.; Mueller, T. J. J. *Synthesis* **2016**, *48*, 974

[8] Kawade, R. K.; Huang, P.-H.; Karad, S. N.; Liu, R.-S. *Org. Biomol. Chem.* **2014**, *12*, 737

[9] Monti, S. A.; Castillo, G. D., Jr. *J. Org. Chem.* **1970**, *35*, 3764

[10] Zhou, S.; Wang, J.; Wang, L.; Chen, K.; Song, C.; Zhu, J. *Org. Lett.* **2016**, *18*, 3806

[11] Paspirgelyte, R.; Grazulevicius, J. V.; Grigalevicius, S.; Jankauskas, V. *React. Funct. Polym.* **2009**, *69*, 183

8. NMR SPECTRA OF THE ISOLATED PRODUCTS

^{1}H NMR

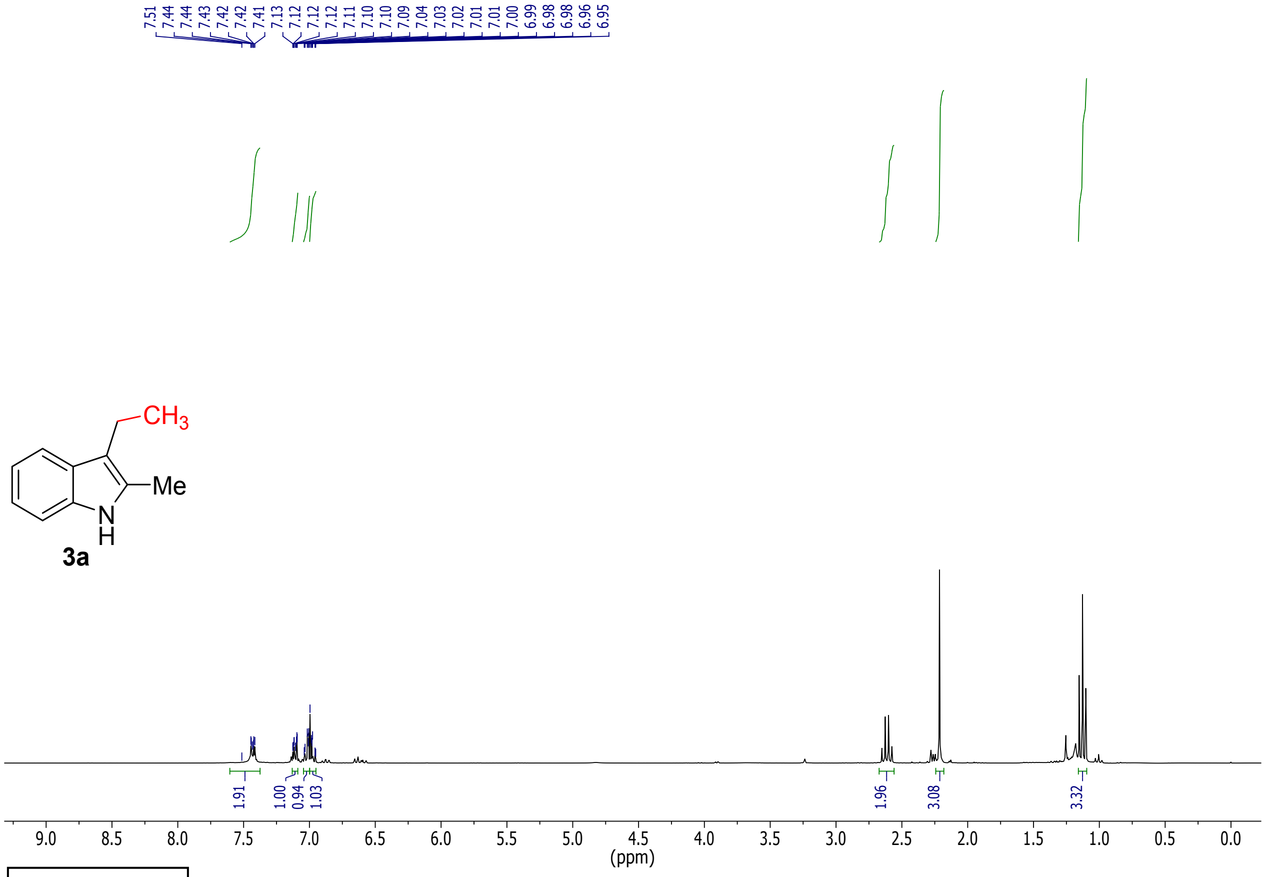

^{13}C NMR

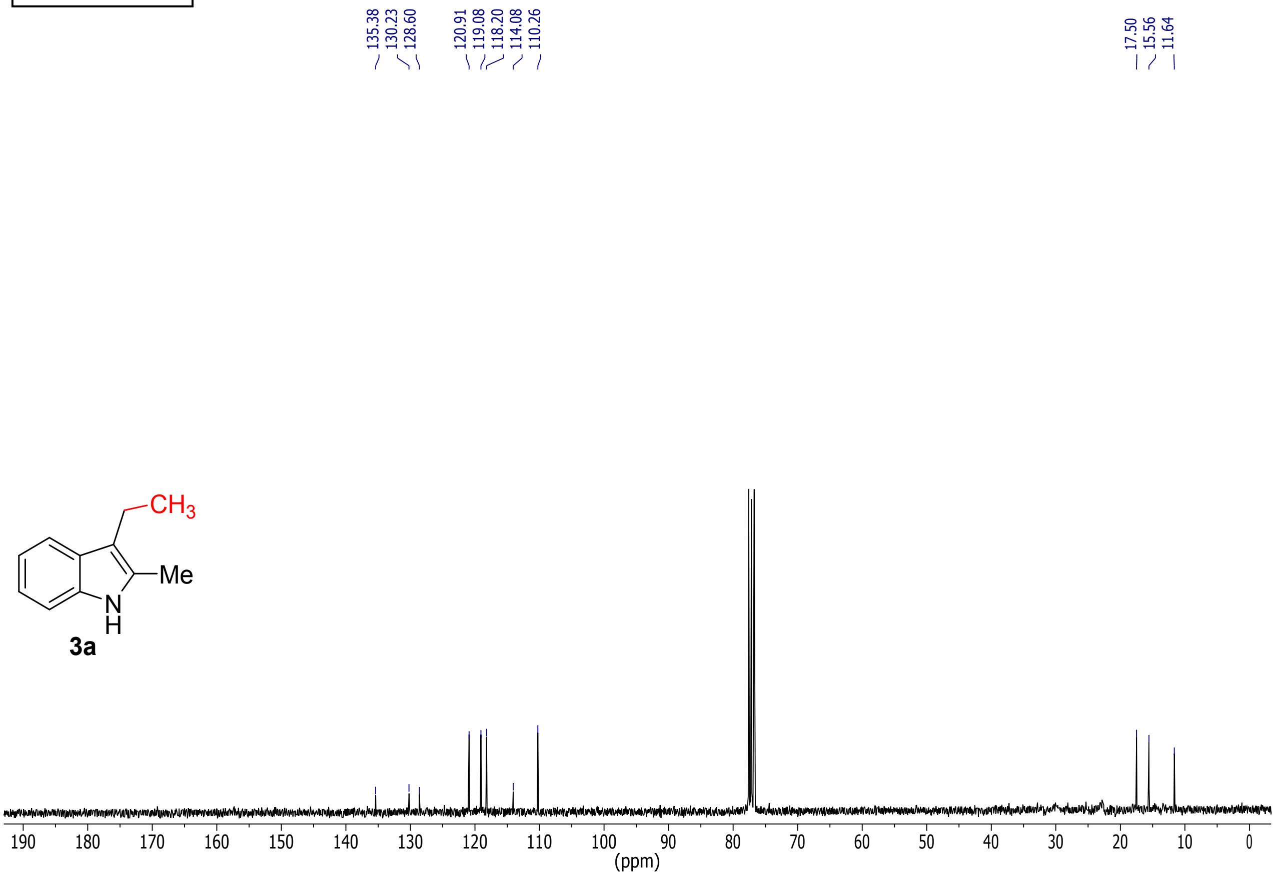

^{1}H NMR

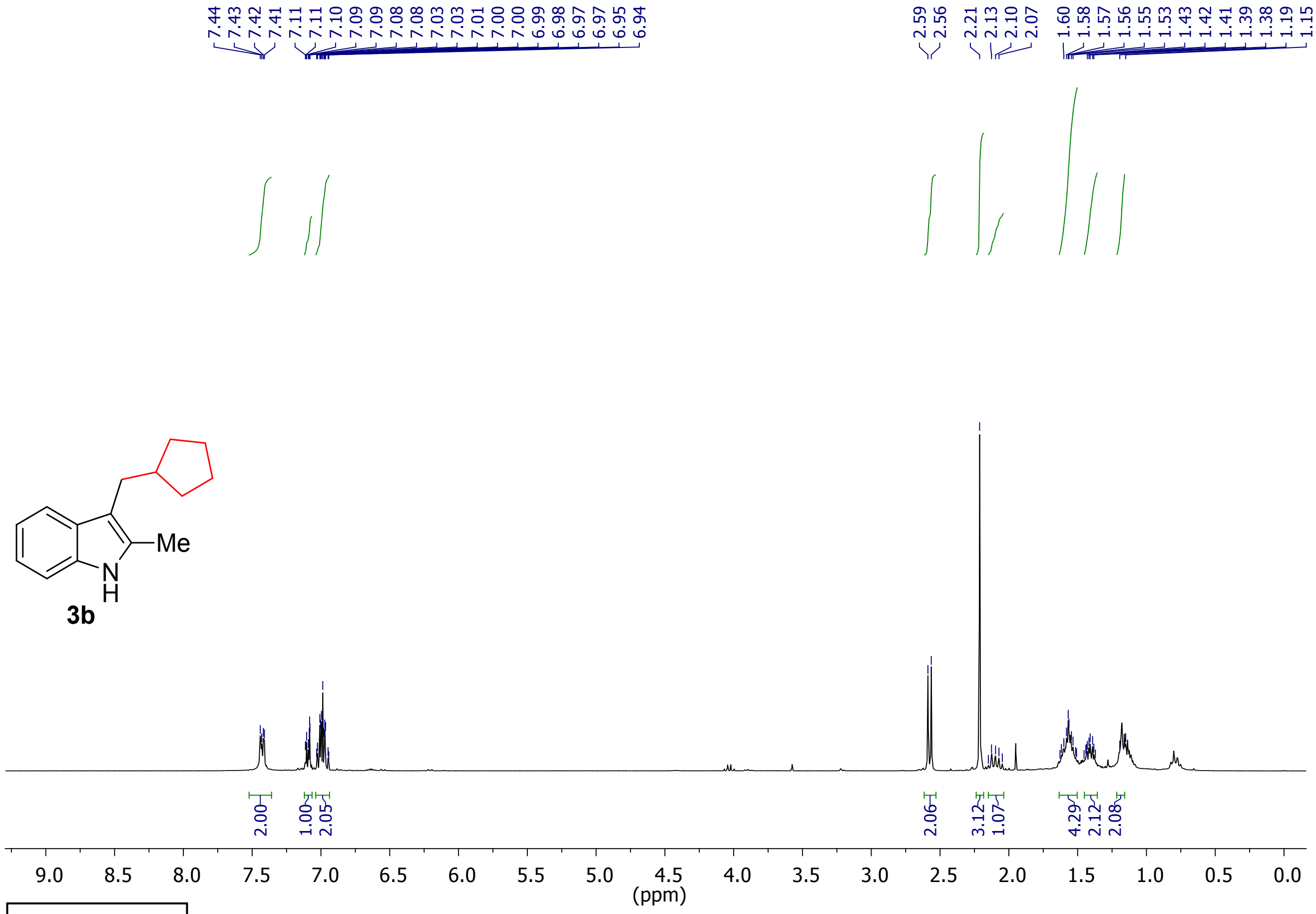

^{13}C NMR

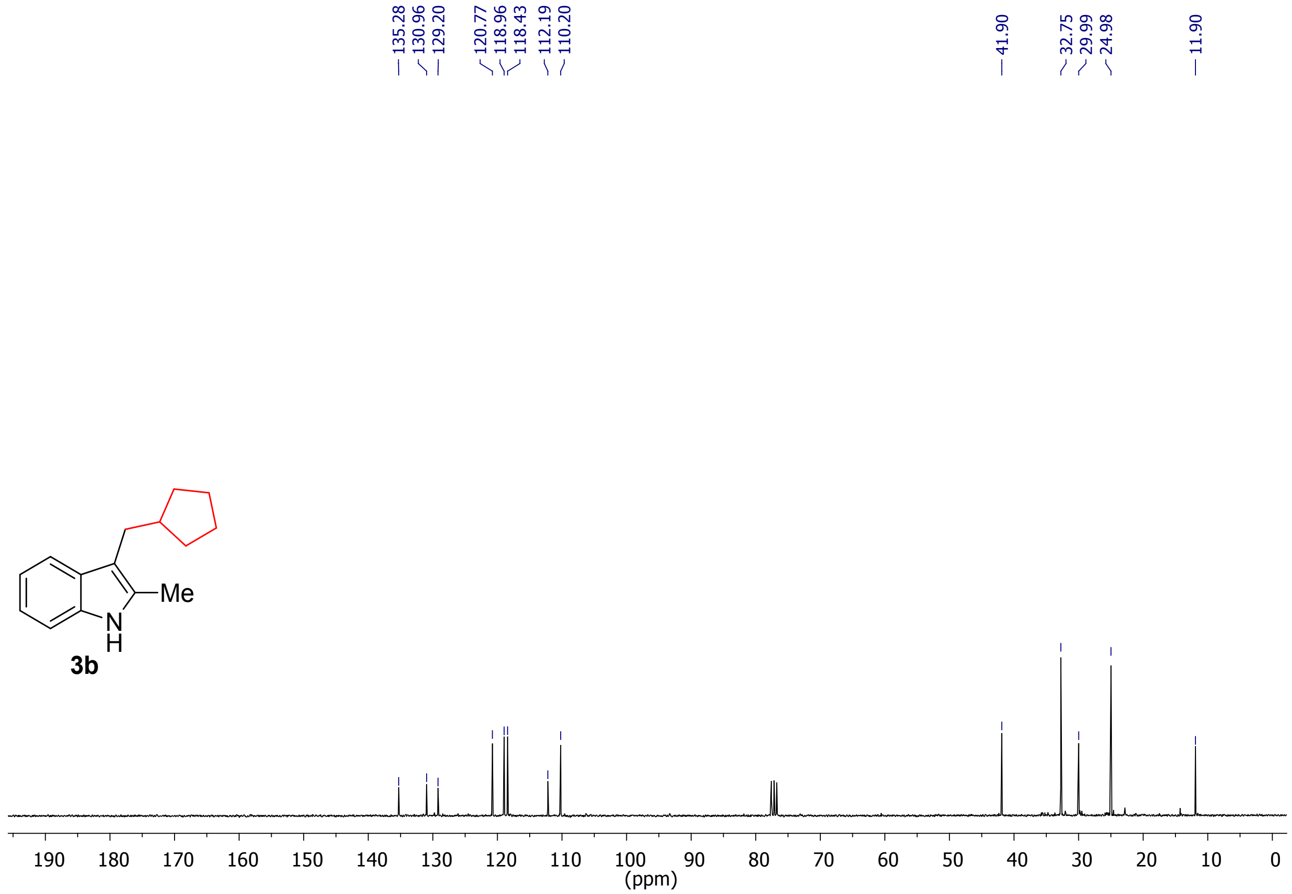

^{1}H NMR

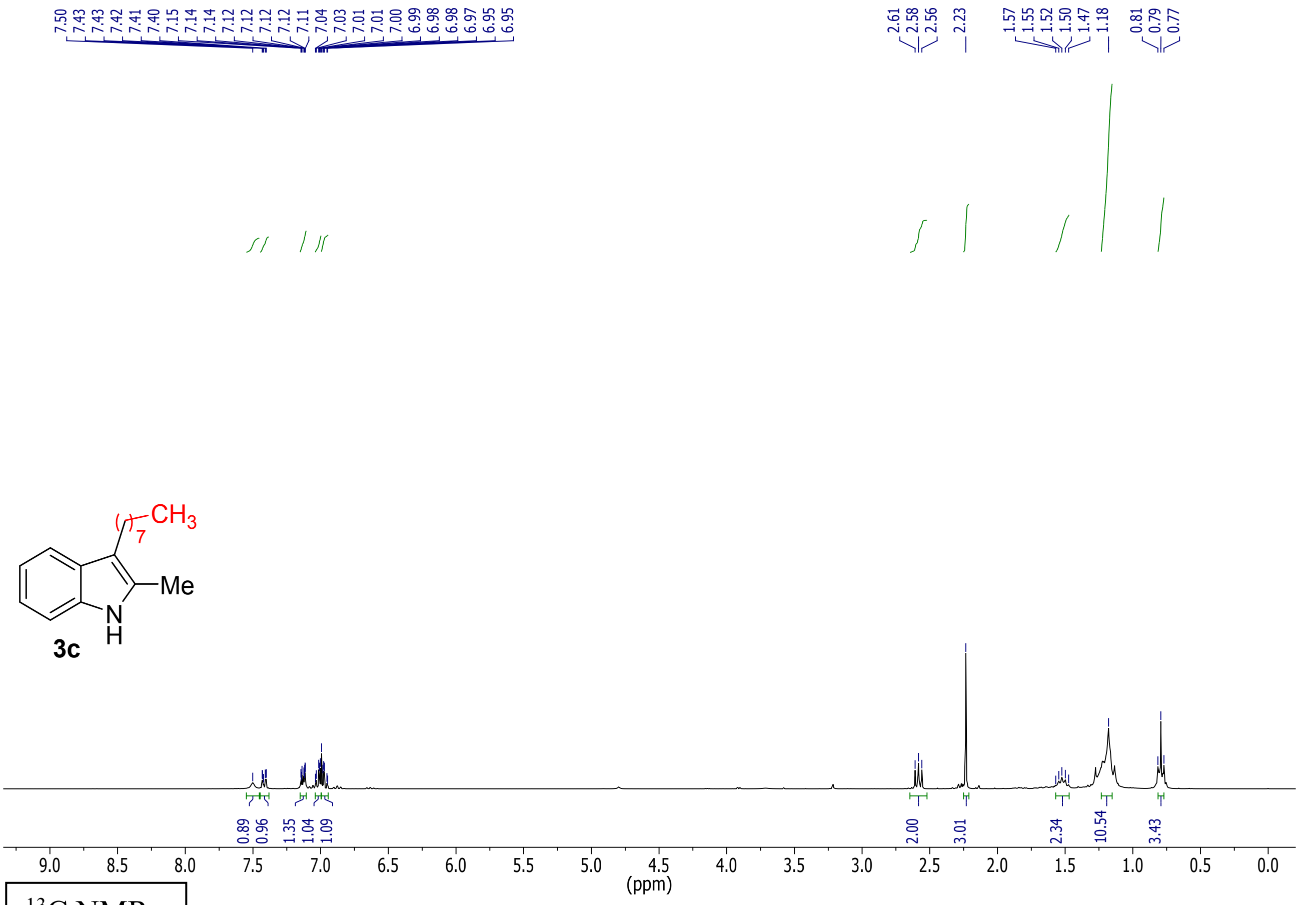

^{13}C NMR

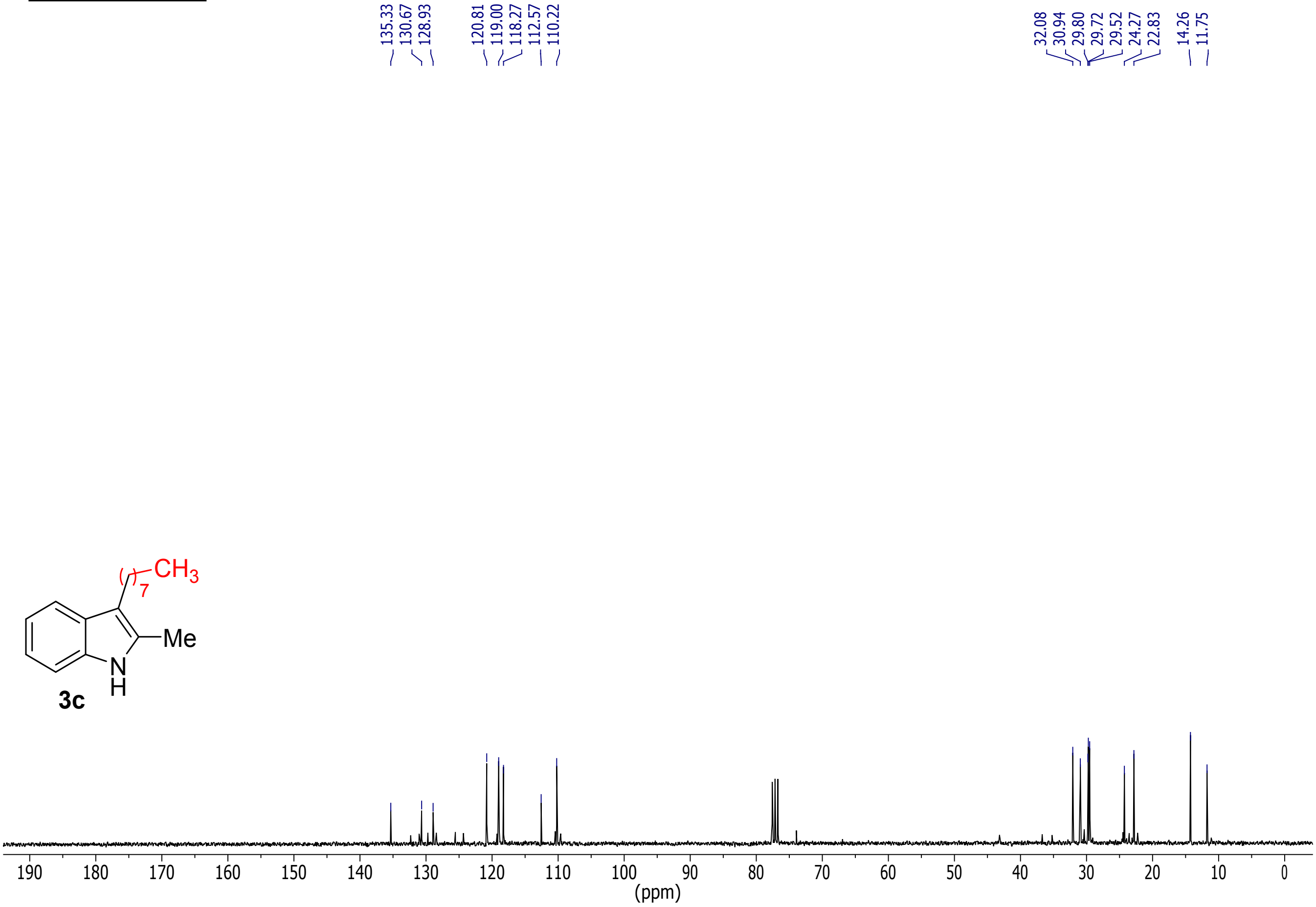

^{1}H NMR

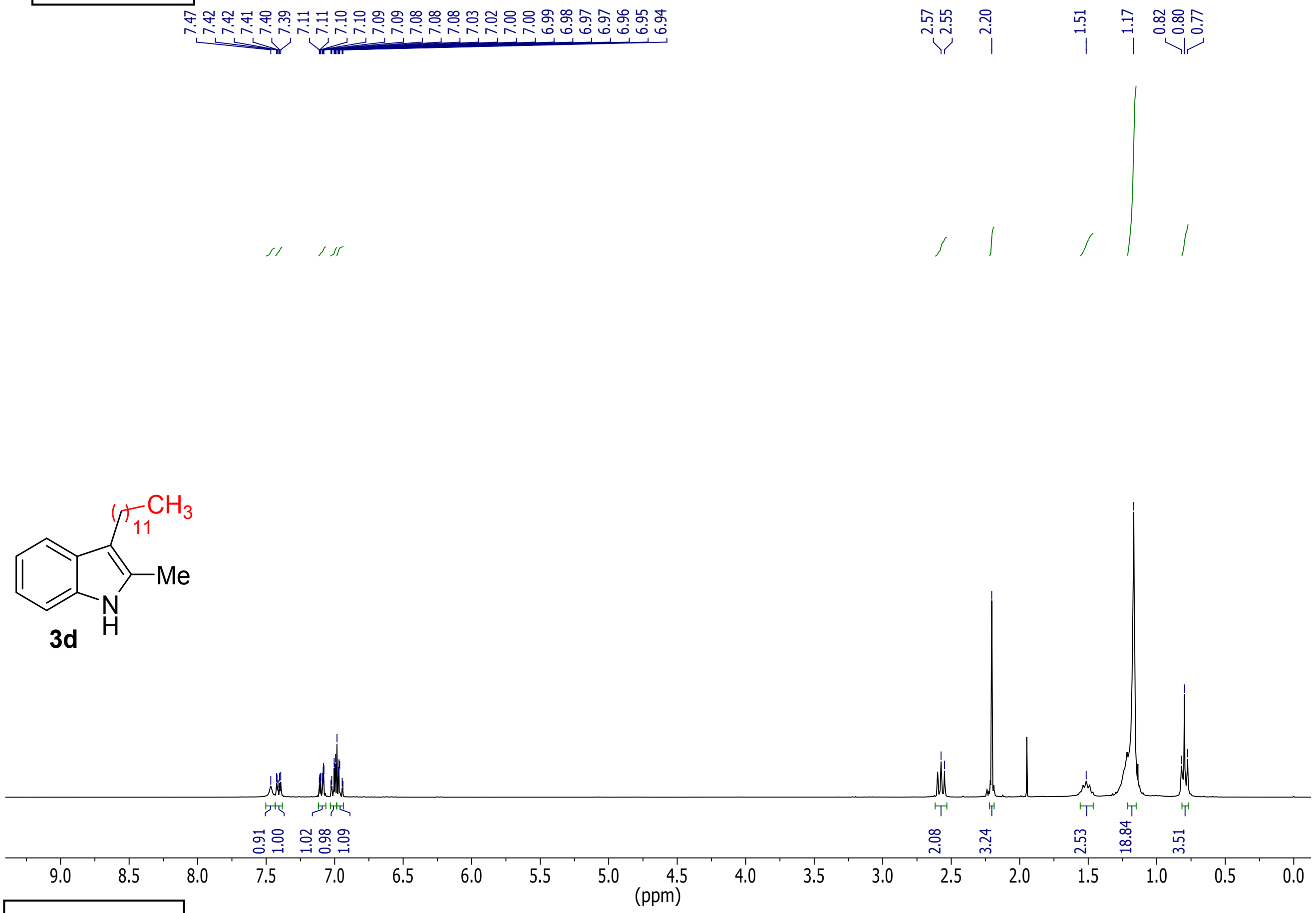

3d

^{13}C NMR

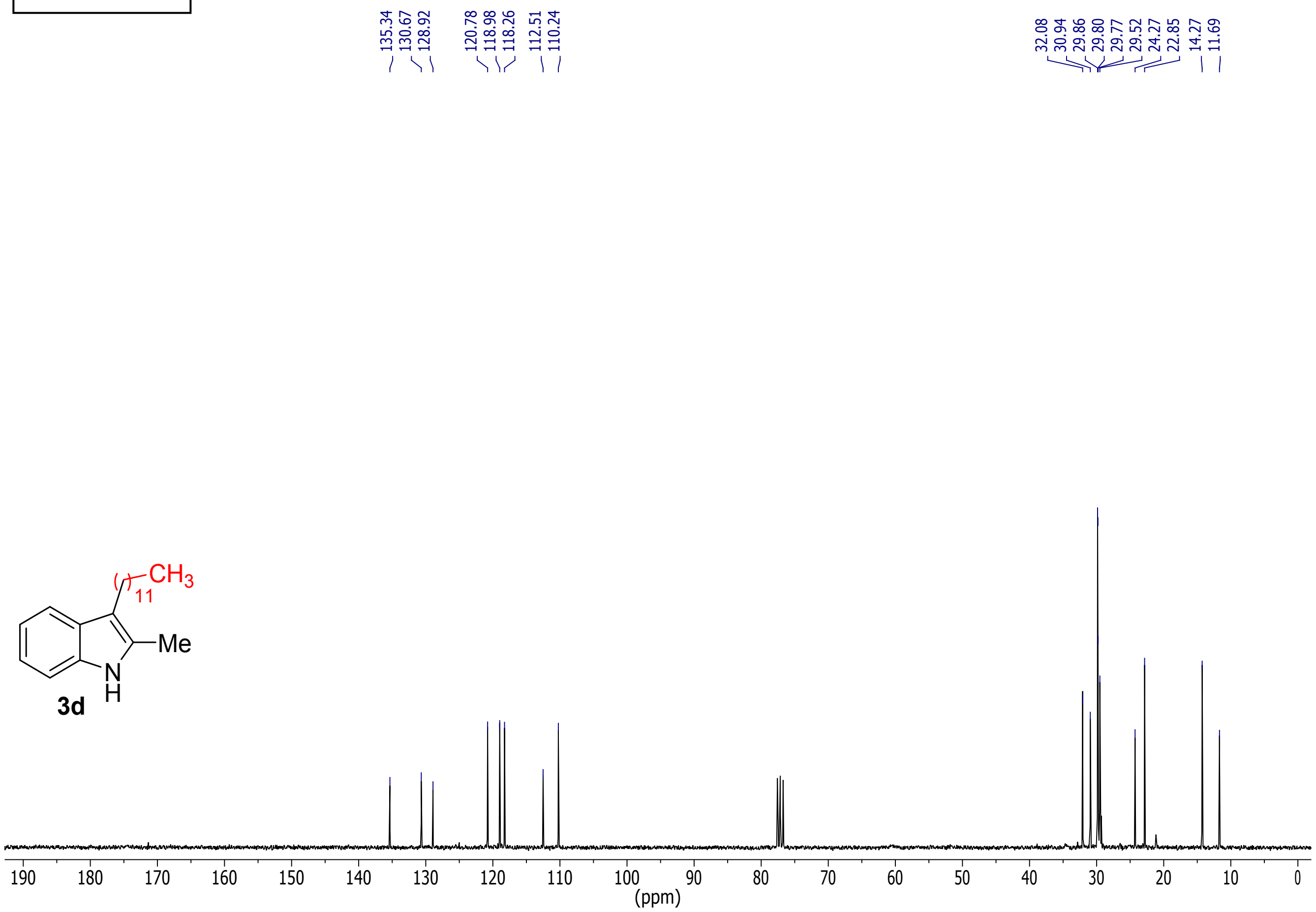

3d

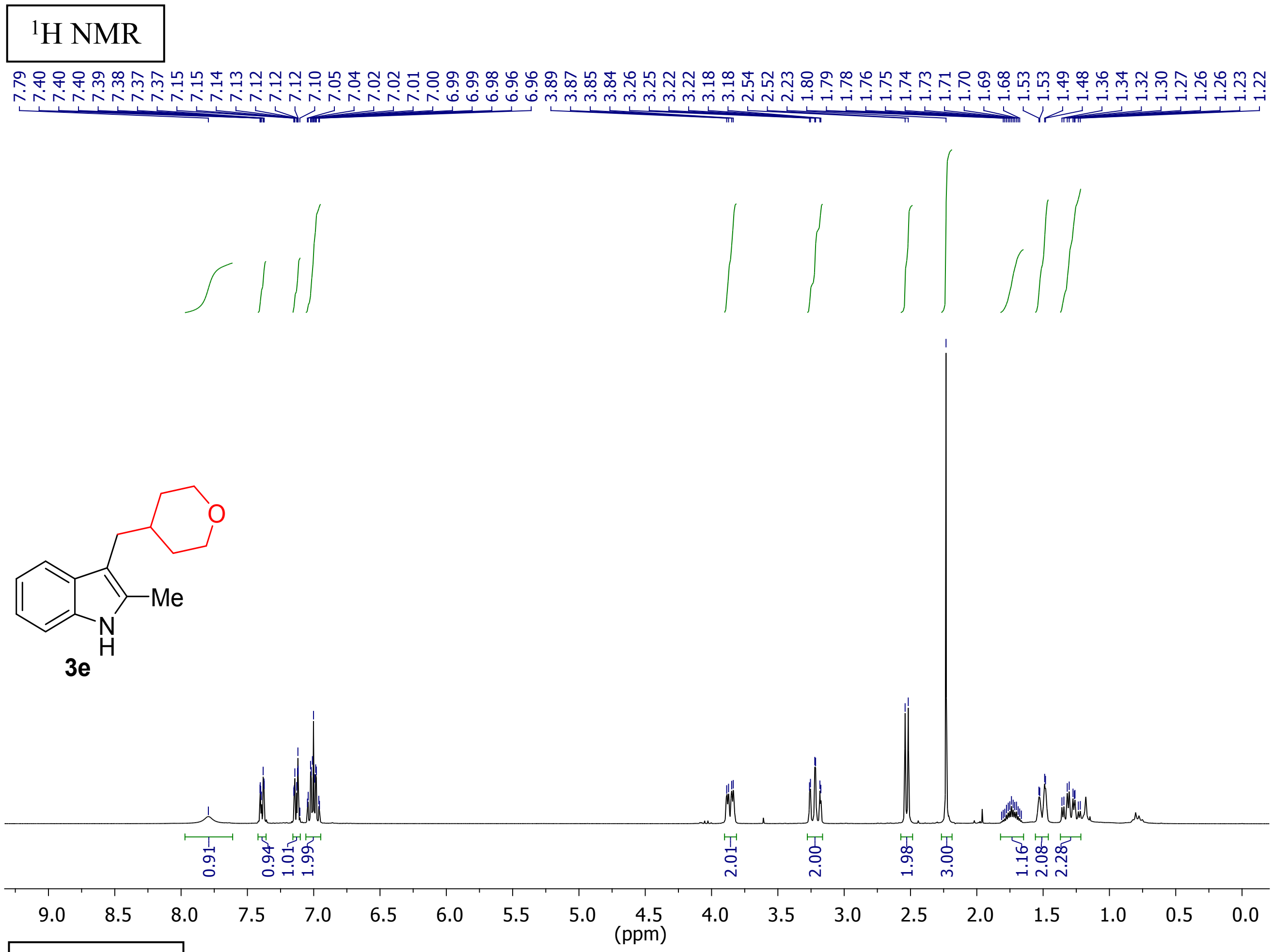
^{1}H NMR
7.79 7.40 7.40 7.40 7.39 7.38 7.37 7.37 7.15 7.15 7.14 7.13 7.12 7.12 7.12 7.10 7.05 7.04 7.02 7.02 7.01 7.00 6.99 6.99 6.98 6.96 3.89 3.87 3.85 3.84 3.26 3.25 3.22 3.22 3.18 3.18 2.54 2.52 2.23 1.80 1.79 1.78 1.76 1.75 1.74 1.73 1.71 1.70 1.69 1.68 1.53 1.53 1.49 1.48 1.36 1.34 1.32 1.30 1.27 1.26 1.26 1.23 1.22
0.91 0.94 1.01 1.99 2.01 2.00 1.98 3.00 1.16 2.08 2.28
O
Me
N
H
3e
9.0 8.5 8.0 7.5 7.0 6.5 6.0 5.5 5.0 4.5 4.0 3.5 3.0 2.5 2.0 1.5 1.0 0.5 0.0
(ppm)

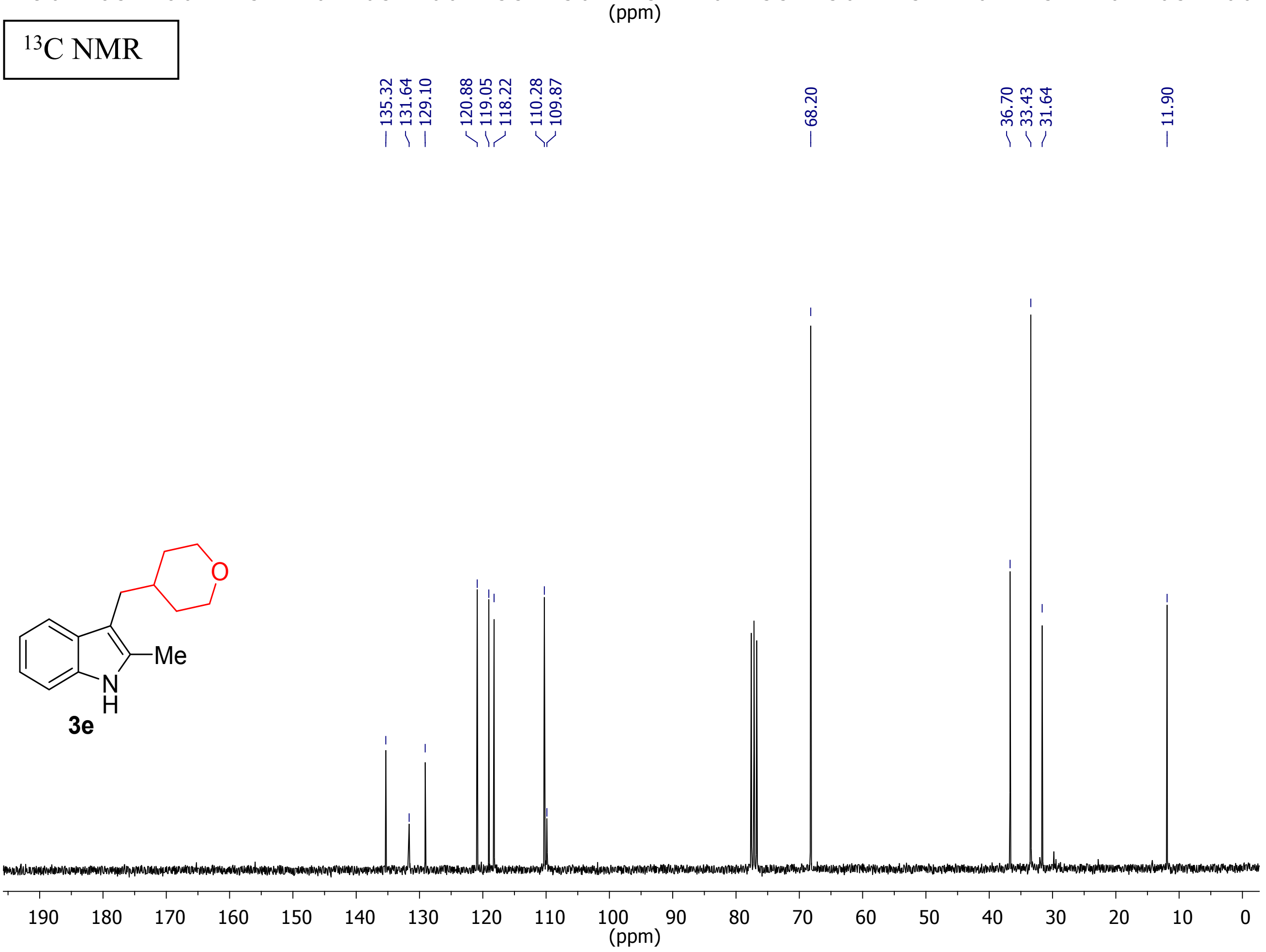
^{13}C NMR
135.32 131.64 129.10 120.88 119.05 118.22 110.28 109.87 68.20 36.70 33.43 31.64 11.90
O
Me
N
H
3e
190 180 170 160 150 140 130 120 110 100 90 80 70 60 50 40 30 20 10 0
(ppm)

¹H NMR

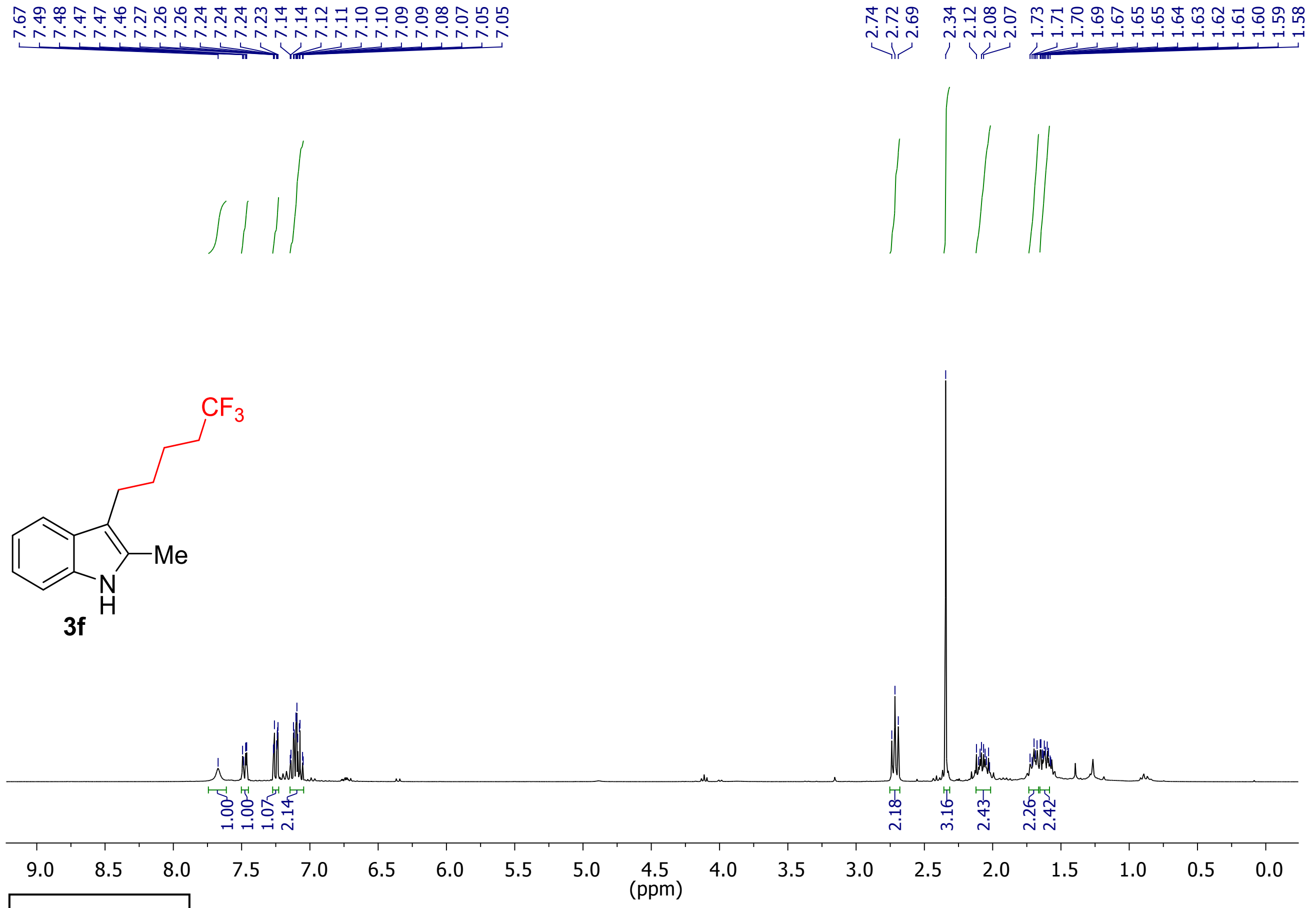

¹³C NMR

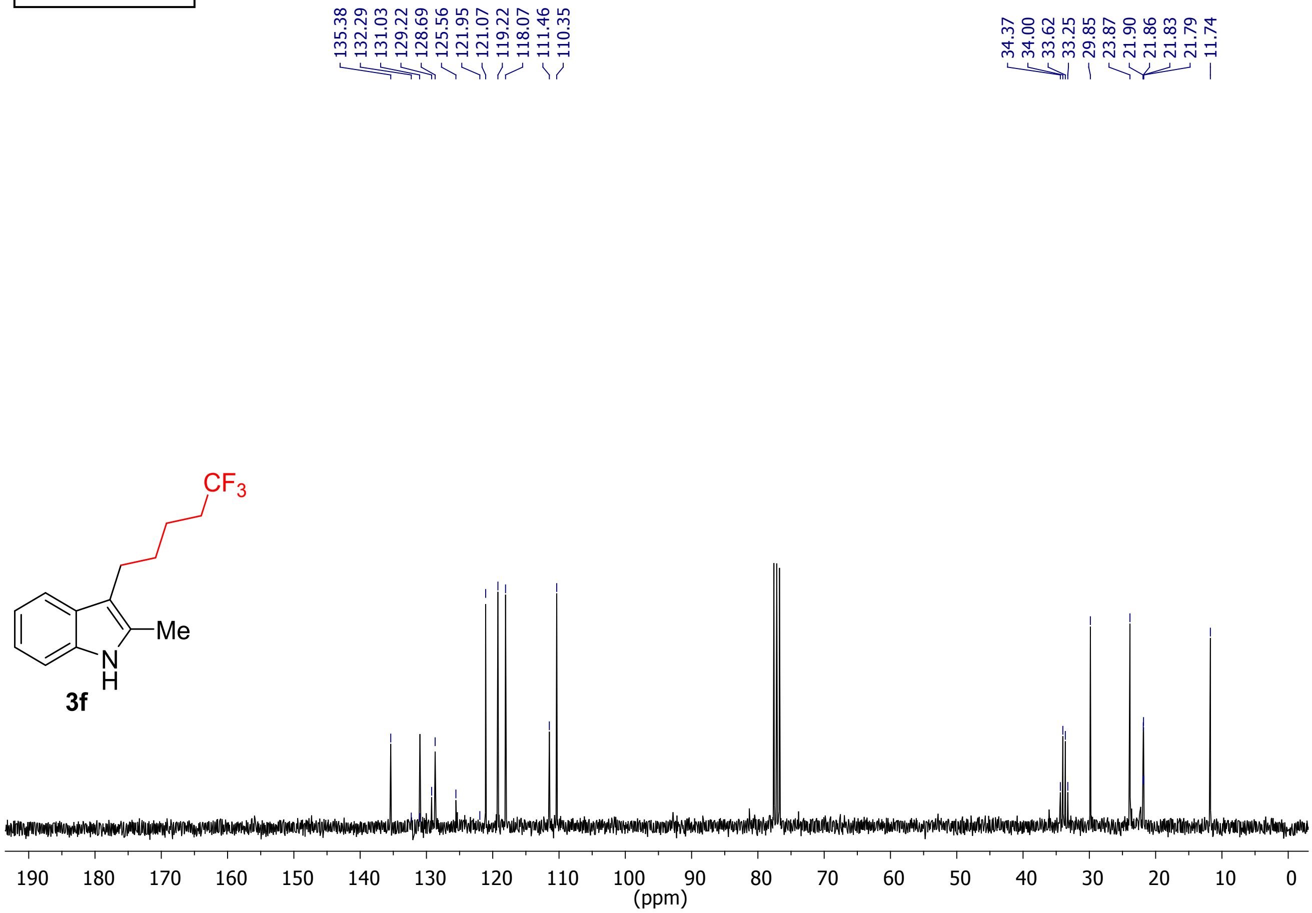

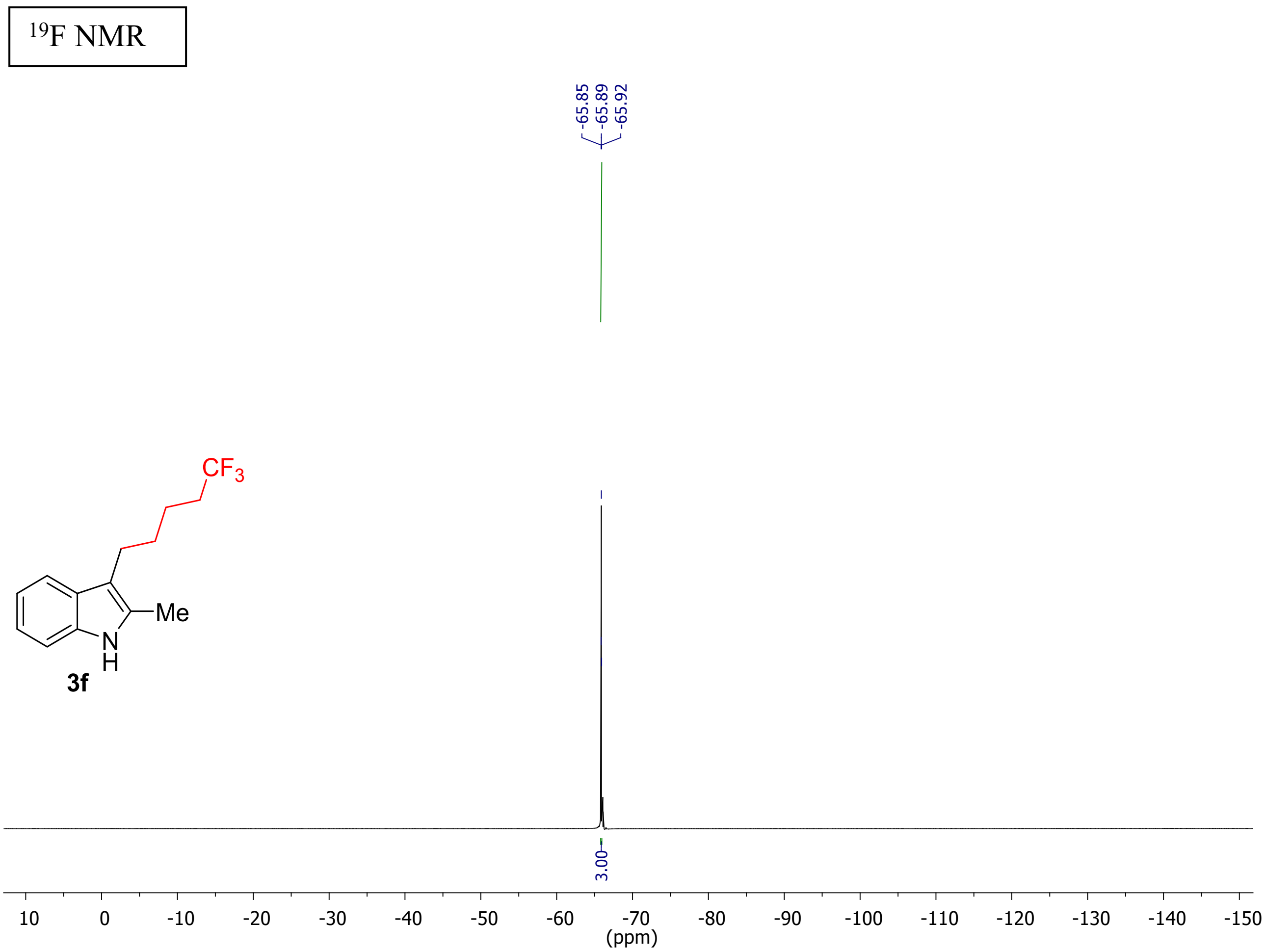

19F NMR
-65.85
-65.89
-65.92
CF3
Me
N
H
3.00
10
0
-10
-20
-30
-40
-50
-60
-70
-80
-90
-100
-110
-120
-130
-140
-150
(ppm)

3f

^{1}H NMR

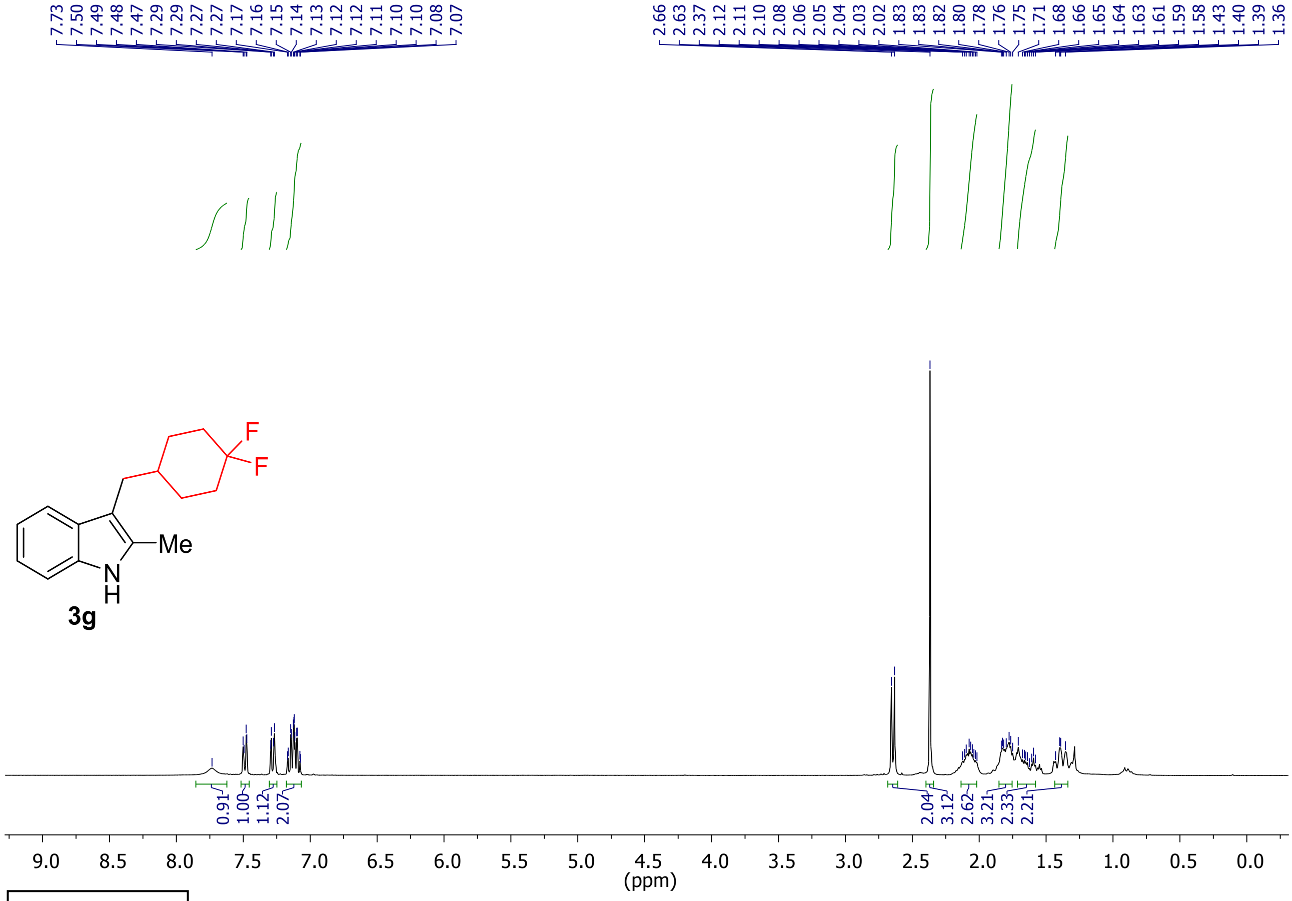

^{13}C NMR

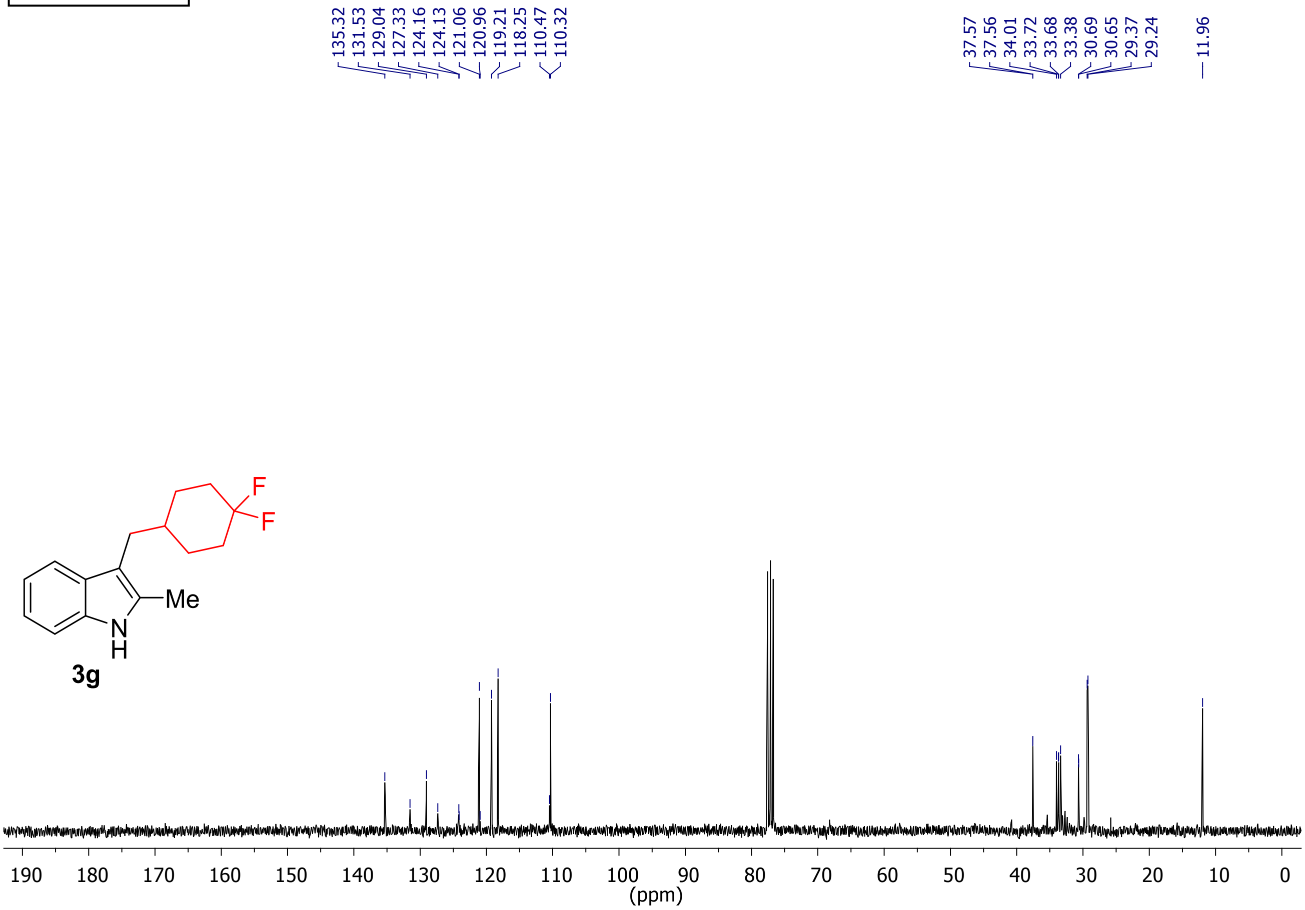

^{19}F NMR

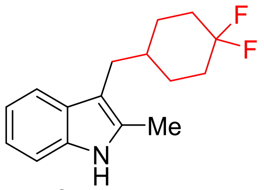

3g

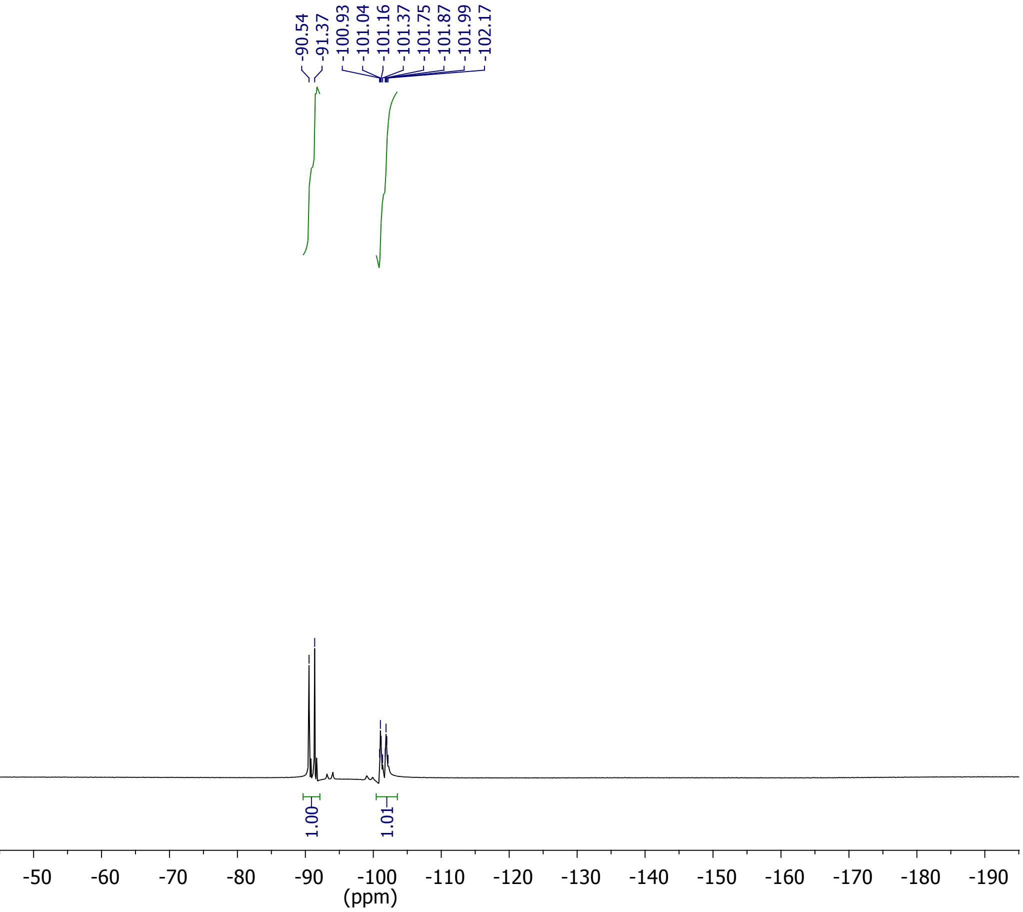
-90.54
-91.37
-100.93
-101.04
-101.16
-101.37
-101.75
-101.87
-101.99
-102.17
1.00
1.01
-10
-20
-30
-40
-50
-60
-70
-80
-90
-100
-110
-120
-130
-140
-150
-160
-170
-180
-190
(ppm)

^{1}H NMR

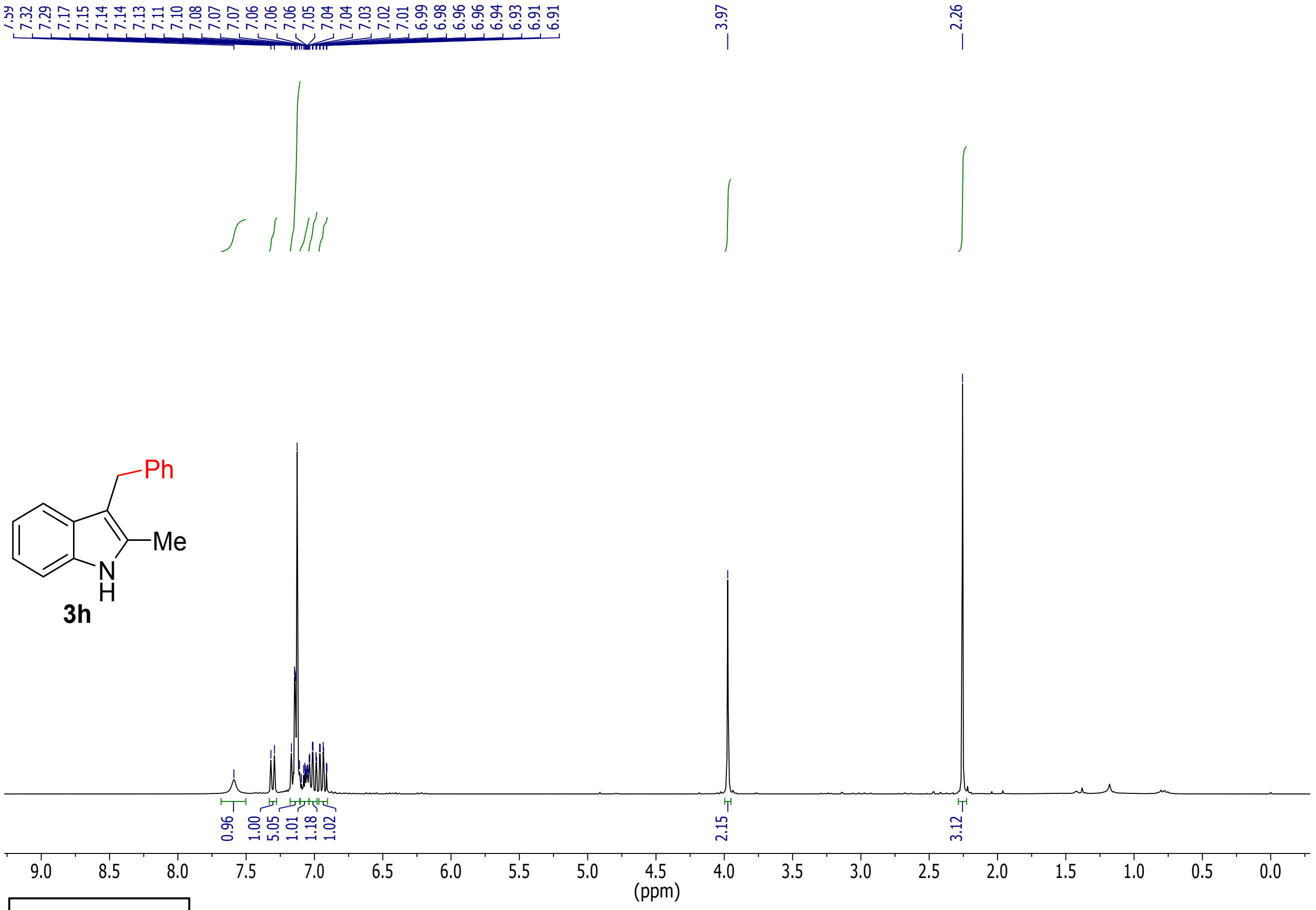

^{13}C NMR

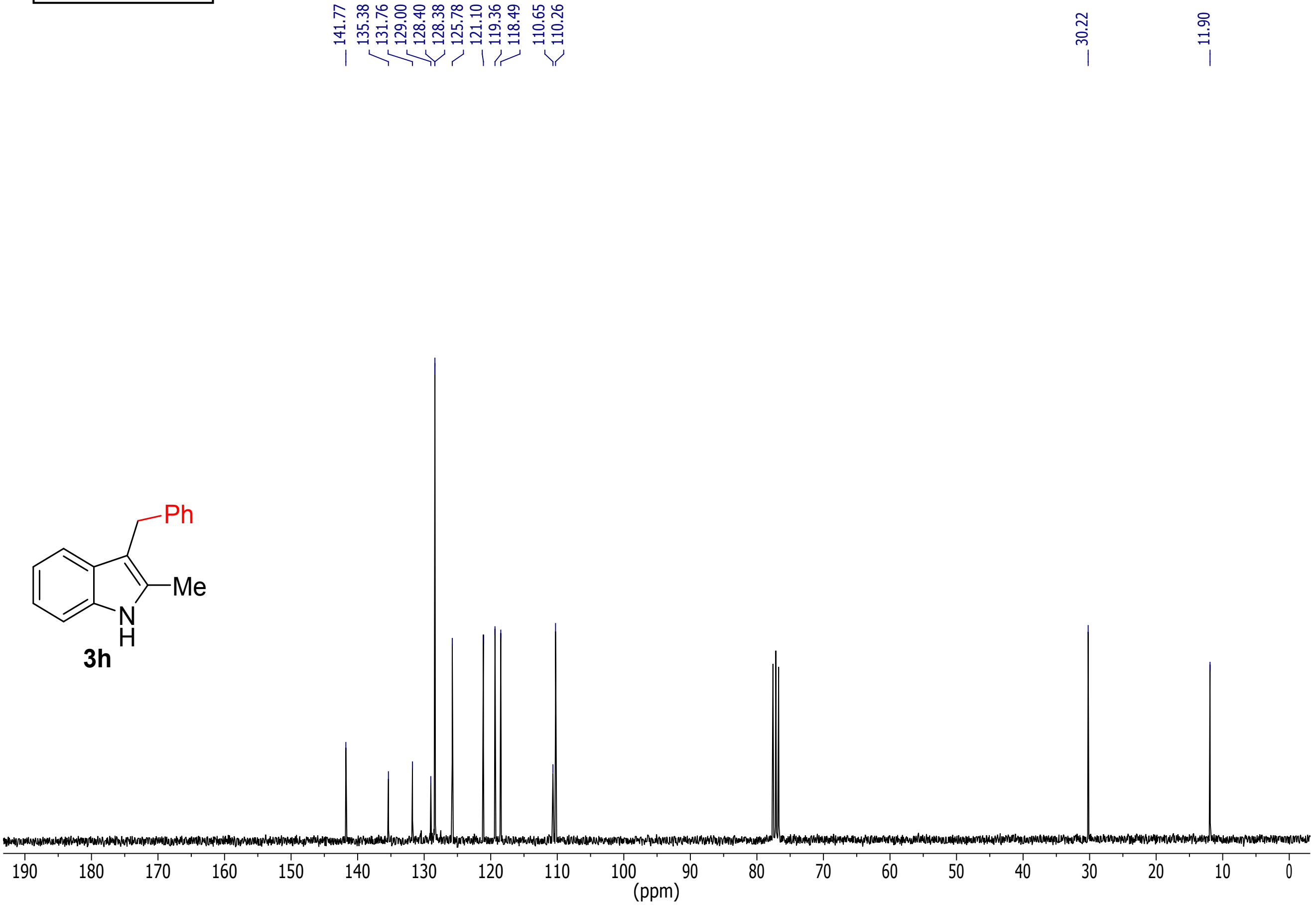

^{1}H NMR

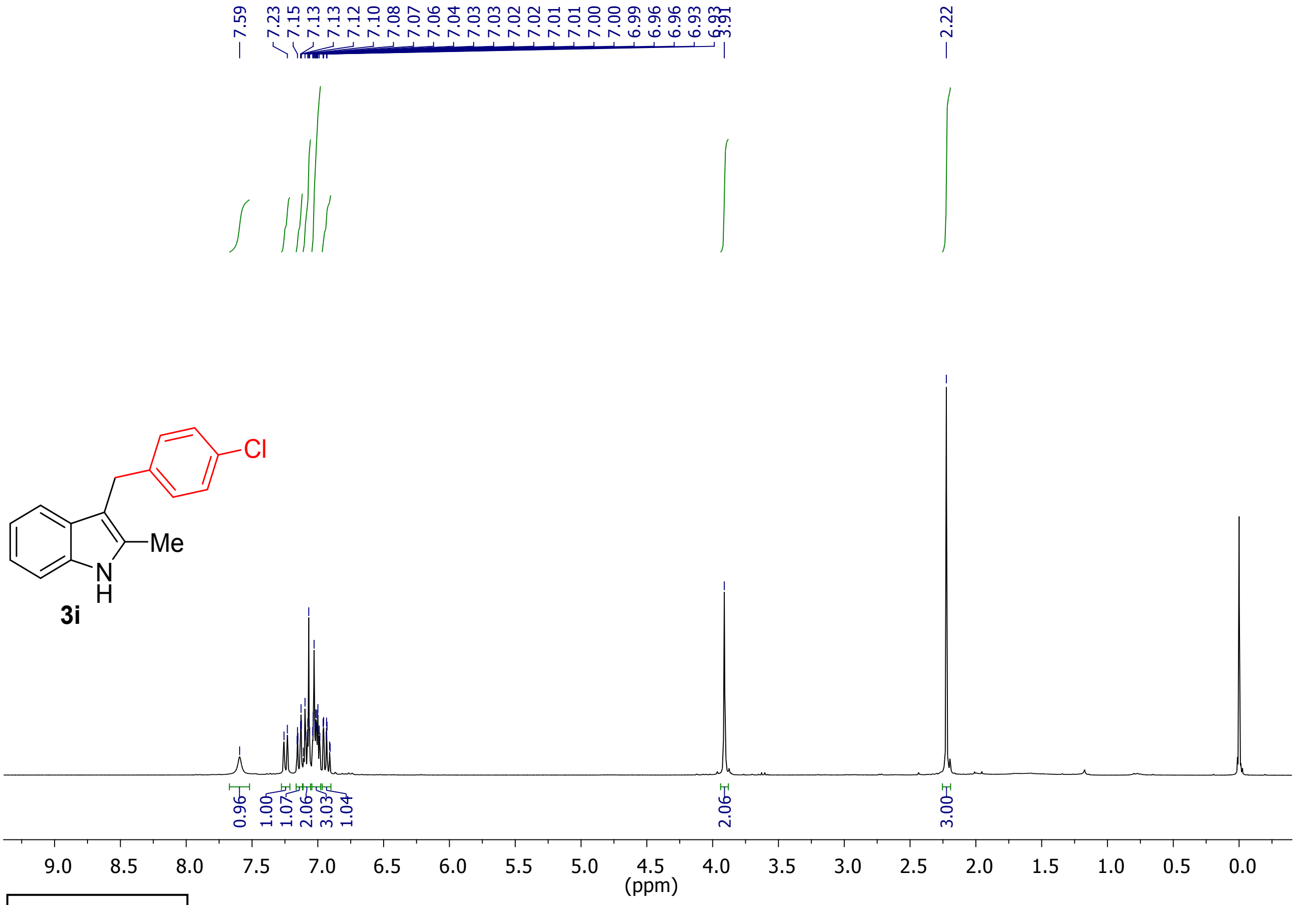

^{13}C NMR

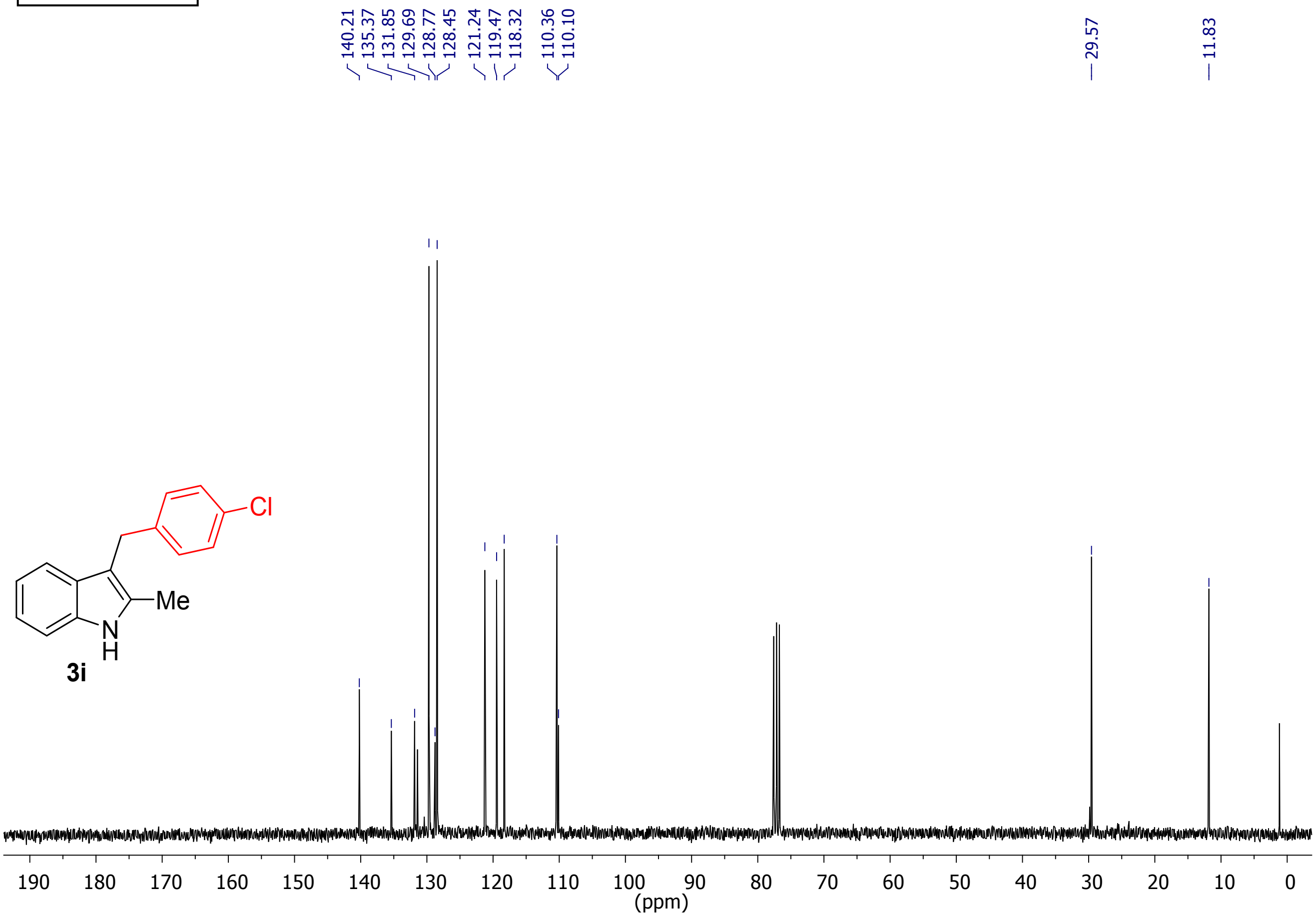

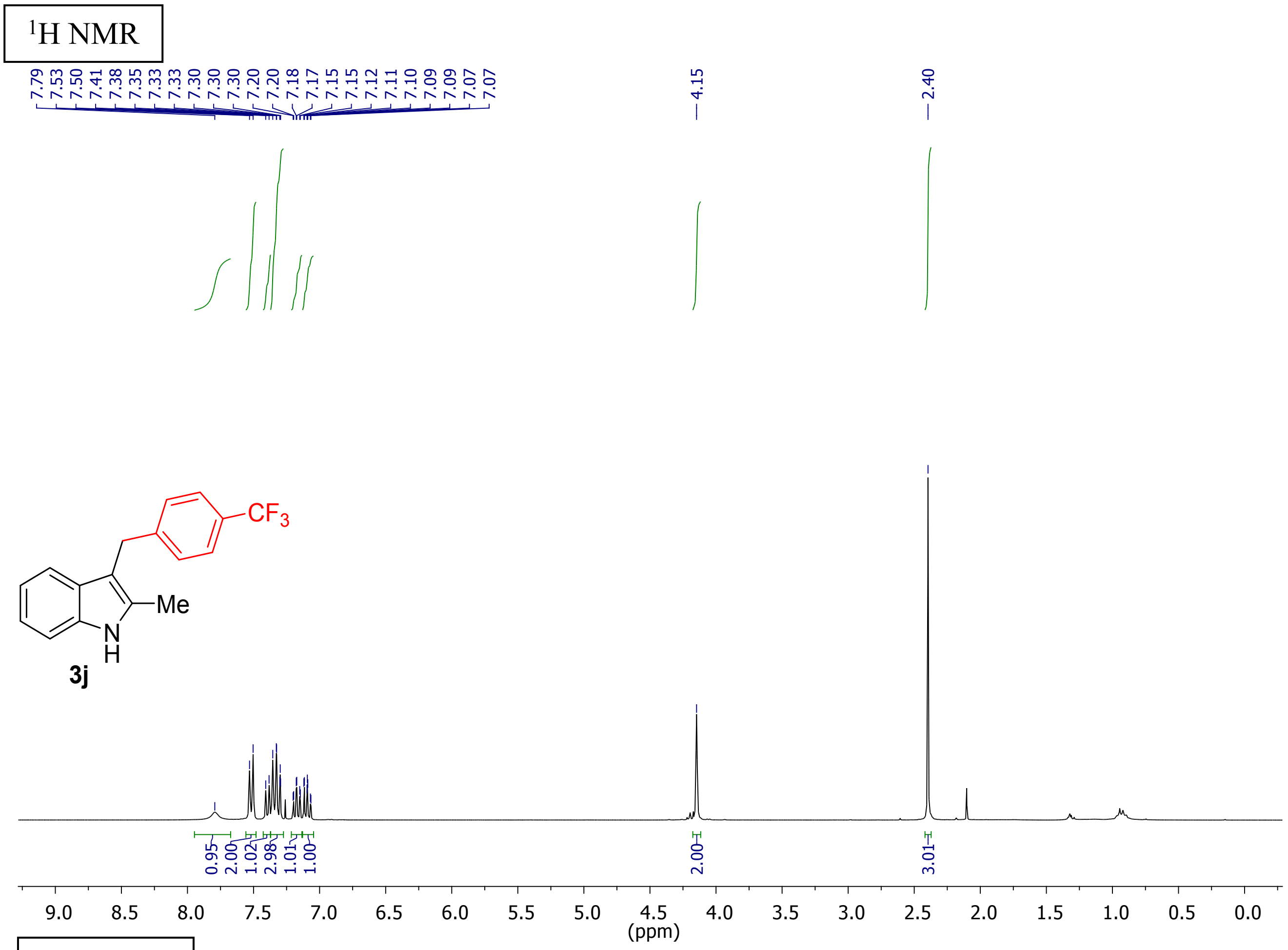

^{1}H NMR
7.79 7.53 7.50 7.41 7.38 7.35 7.33 7.33 7.30 7.30 7.30 7.20 7.20 7.18 7.17 7.15 7.15 7.12 7.11 7.10 7.09 7.09 7.07 7.07
4.15
2.40
CF3
Me
N
H
3j
0.95 2.00 1.02 2.98 1.01 1.00
2.00
3.01
9.0 8.5 8.0 7.5 7.0 6.5 6.0 5.5 5.0 4.5 4.0 3.5 3.0 2.5 2.0 1.5 1.0 0.5 0.0
(ppm)

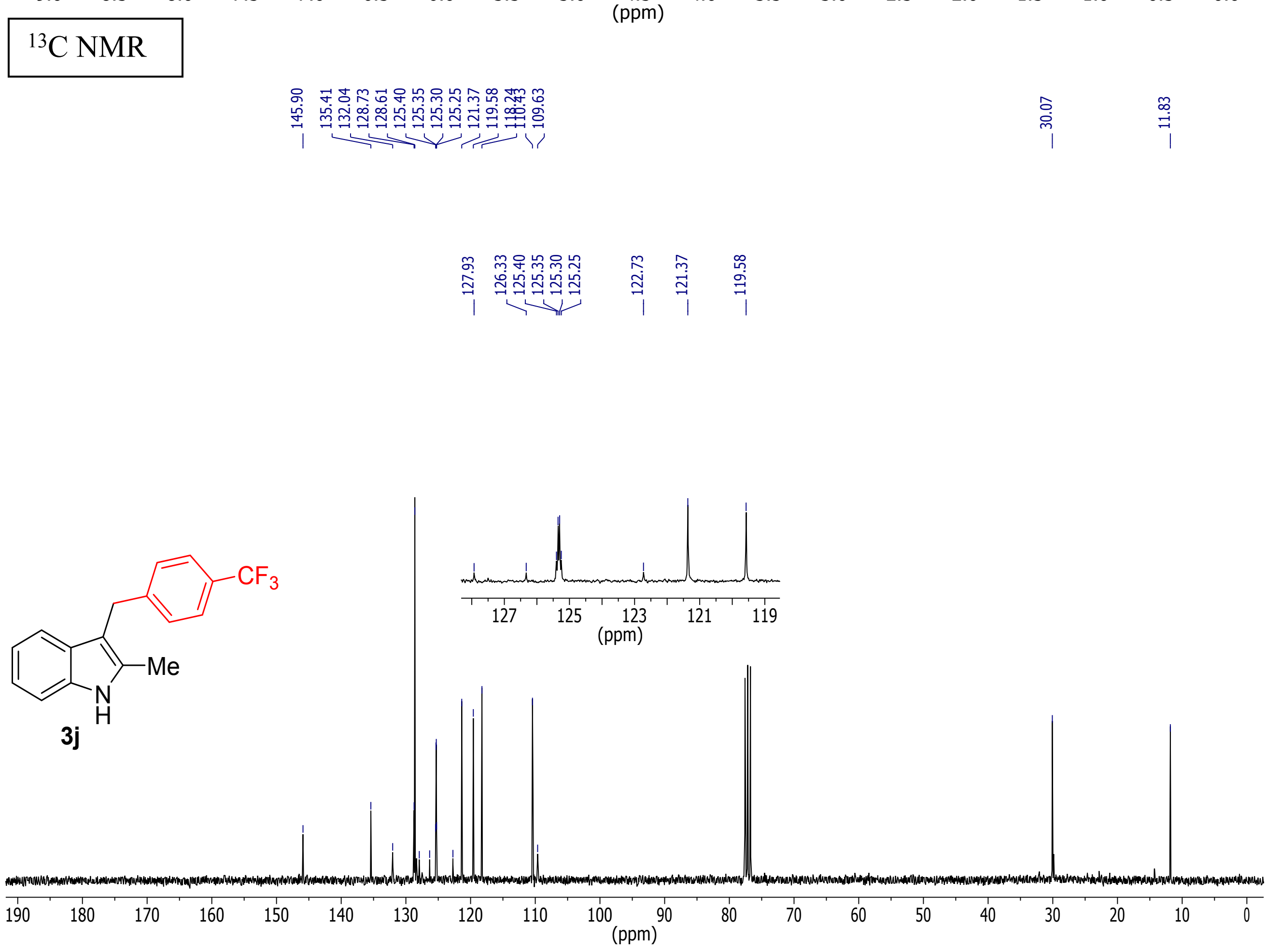

^{13}C NMR
145.90 135.41 132.04 128.73 128.61 125.40 125.35 125.30 125.25 121.37 119.58 118.24 110.43 109.63
30.07
11.83
127.93 126.33 125.40 125.35 125.30 125.25 122.73 121.37 119.58
127 125 123 121 119
(ppm)
CF3
Me
N
H
3j
190 180 170 160 150 140 130 120 110 100 90 80 70 60 50 40 30 20 10 0
(ppm)

¹⁹F NMR

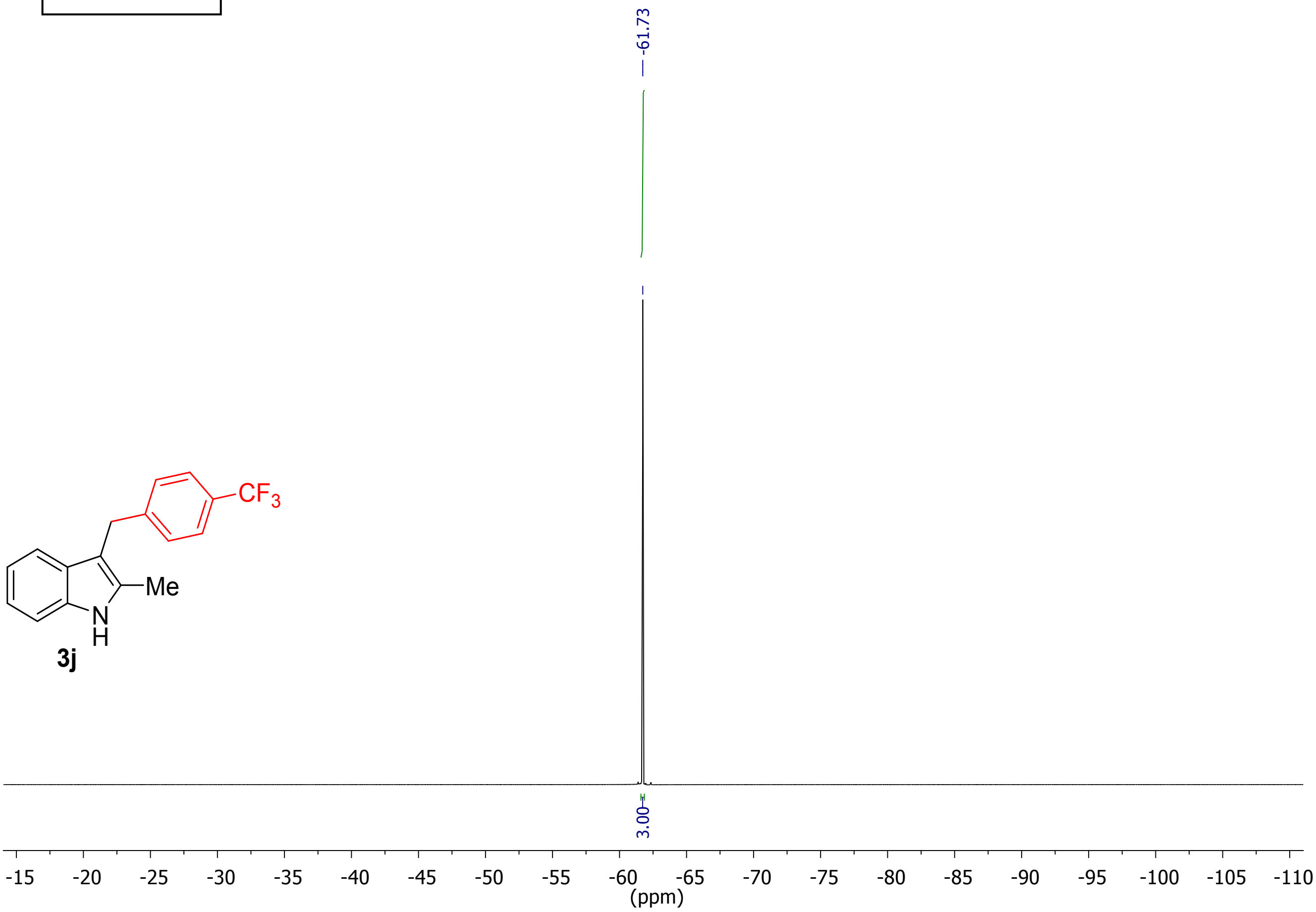
-61.73
CF$_3$
Me
N
H
3.00
-15 -20 -25 -30 -35 -40 -45 -50 -55 -60 -65 -70 -75 -80 -85 -90 -95 -100 -105 -110
(ppm)

3j

^{1}H NMR

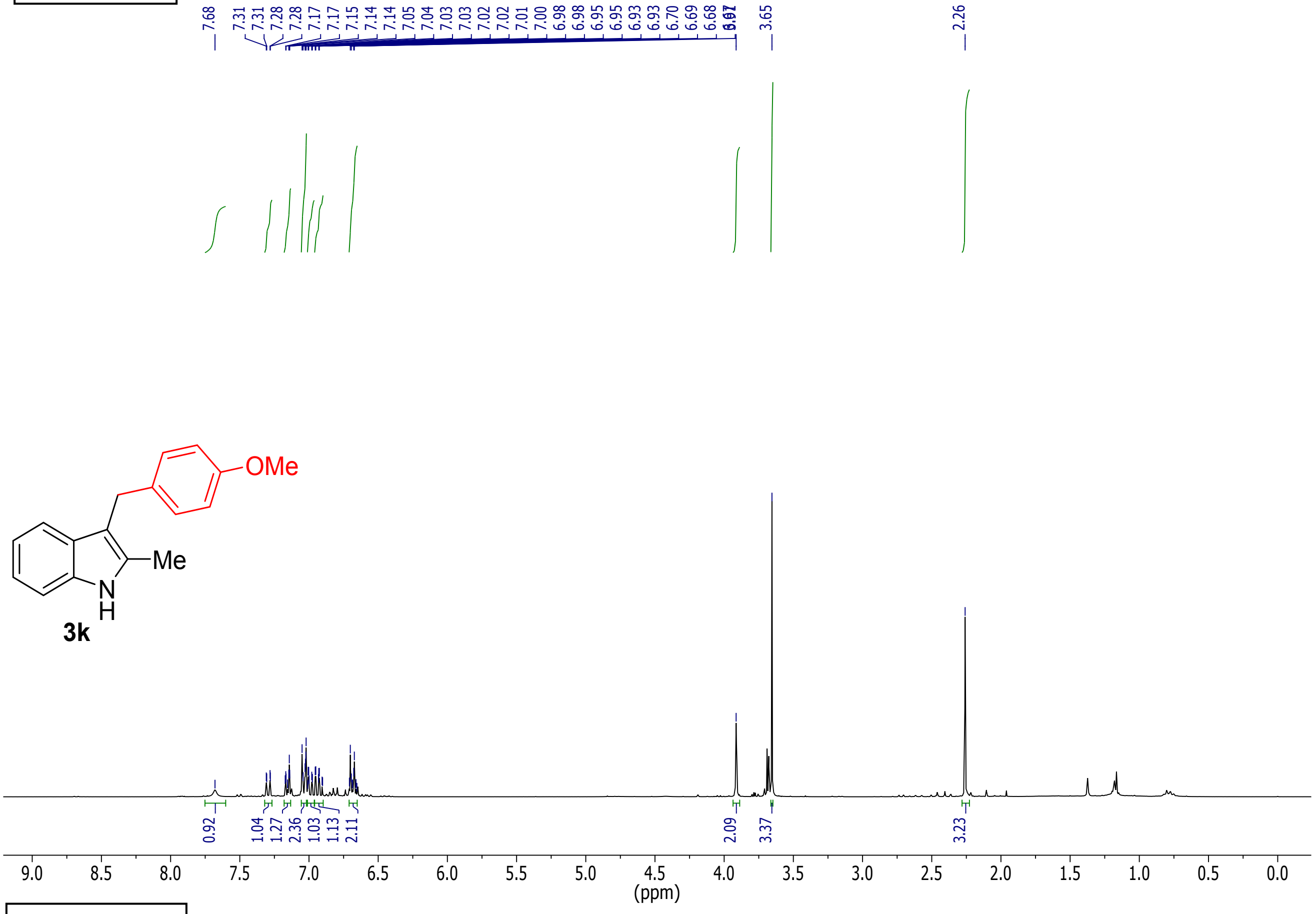

^{13}C NMR

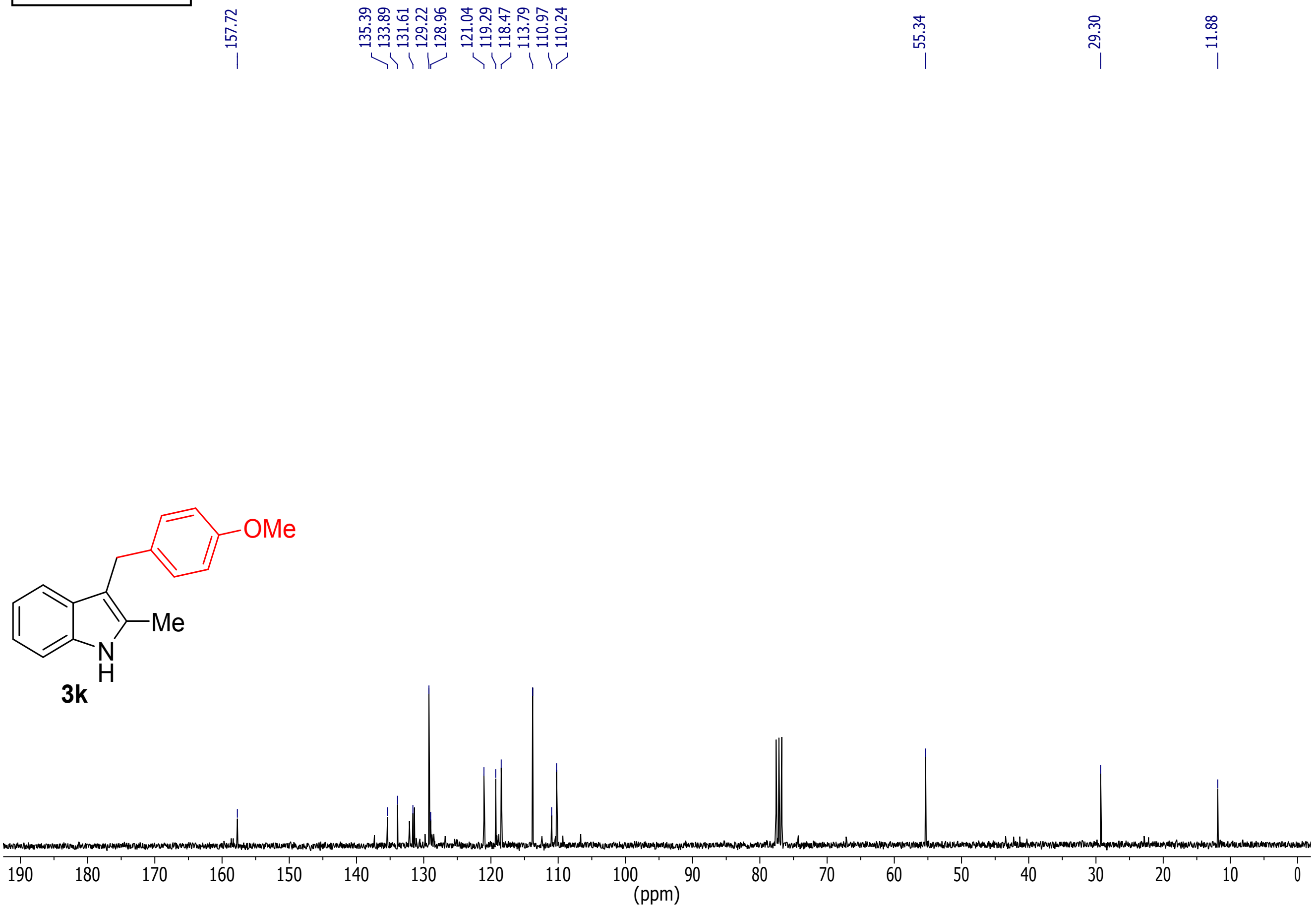

^{1}H NMR

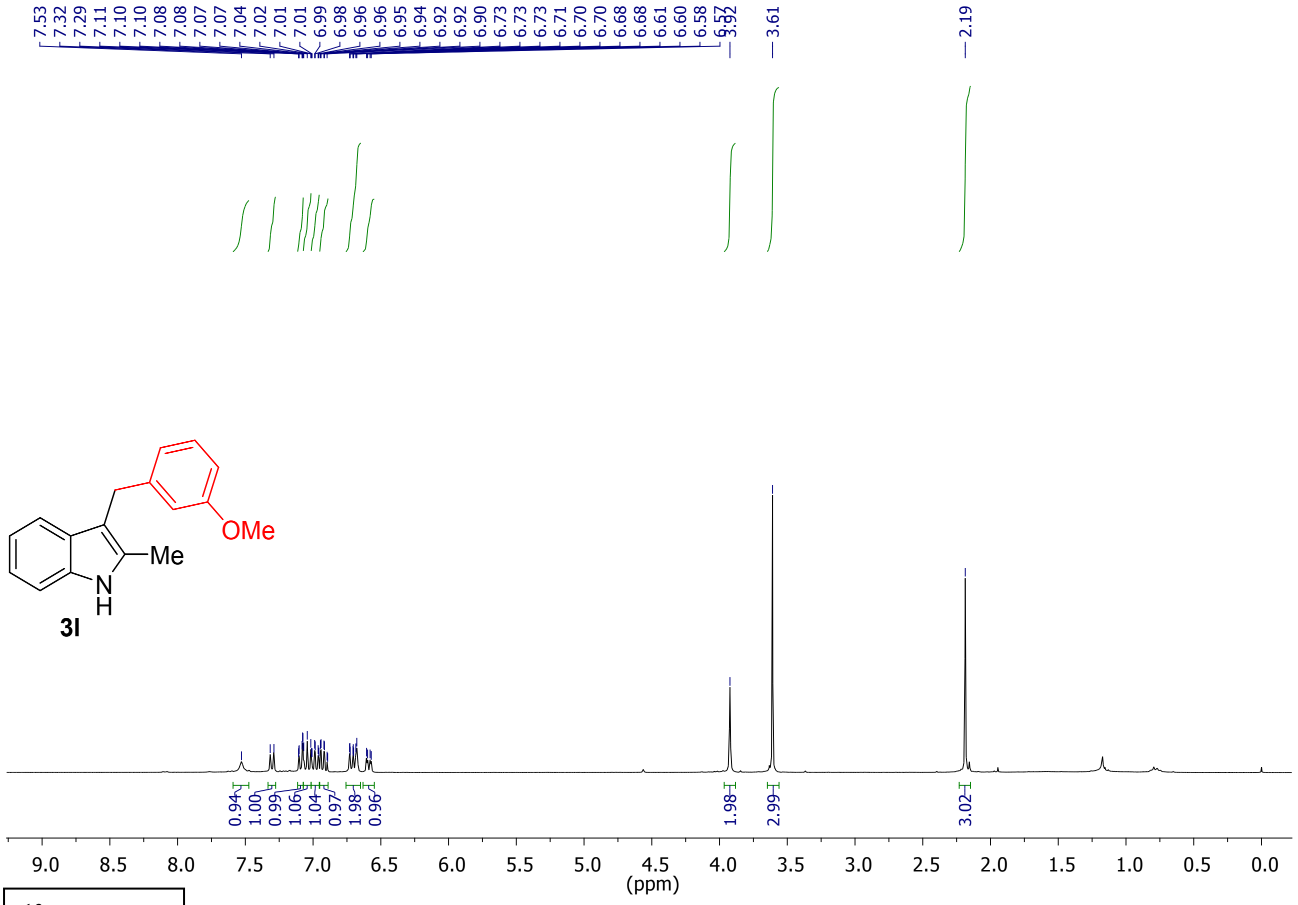

^{13}C NMR

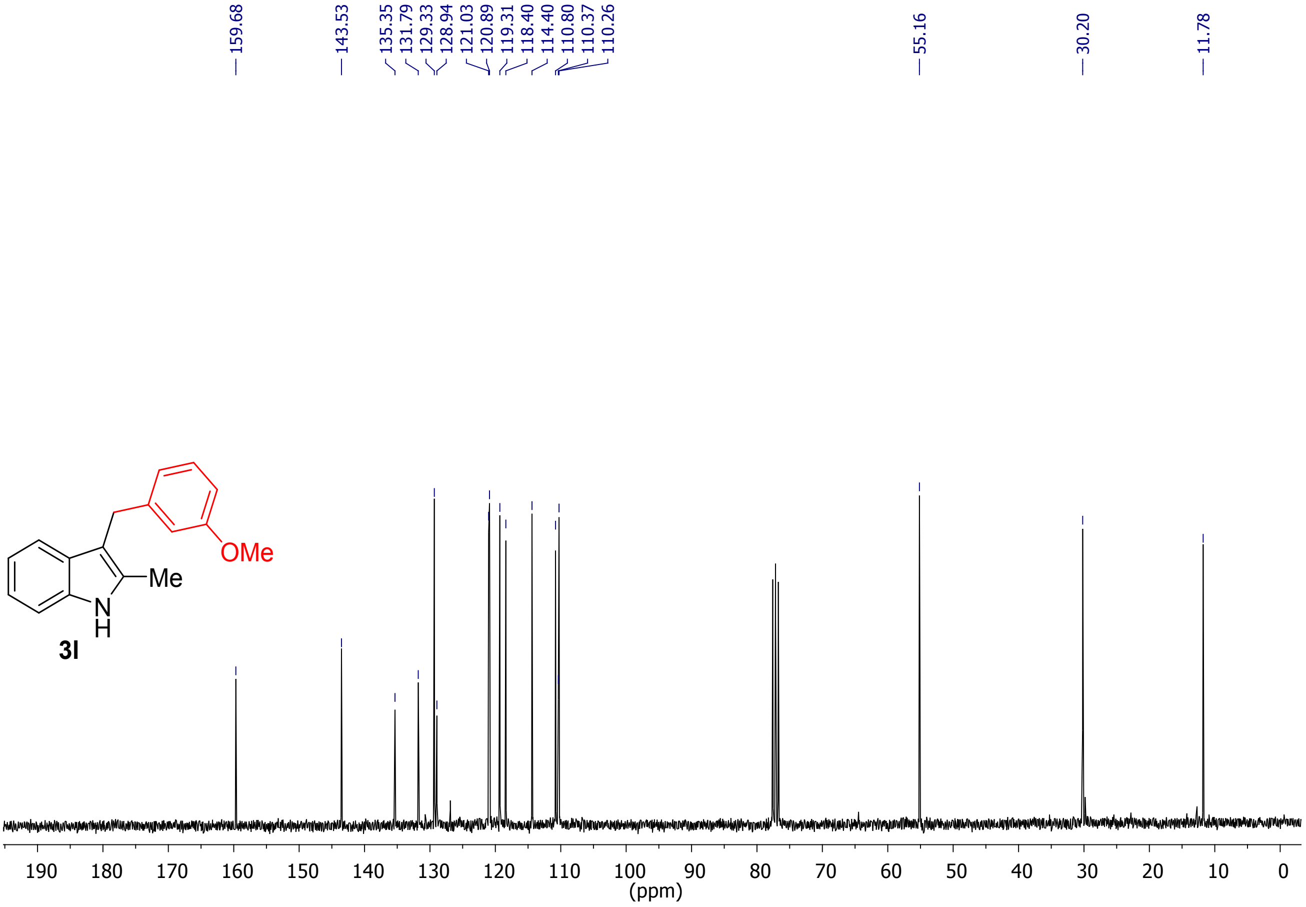

^{1}H NMR

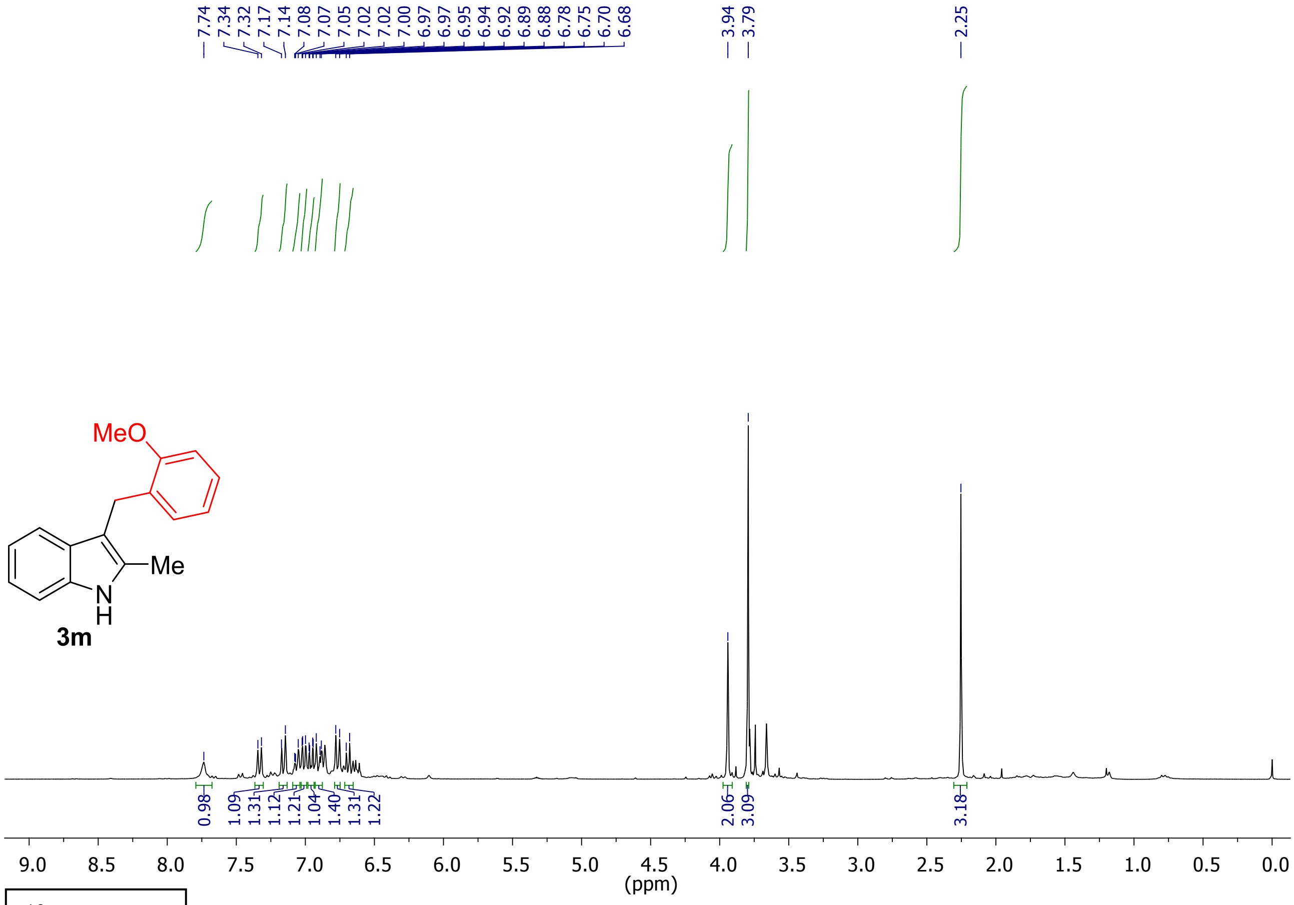

^{13}C NMR

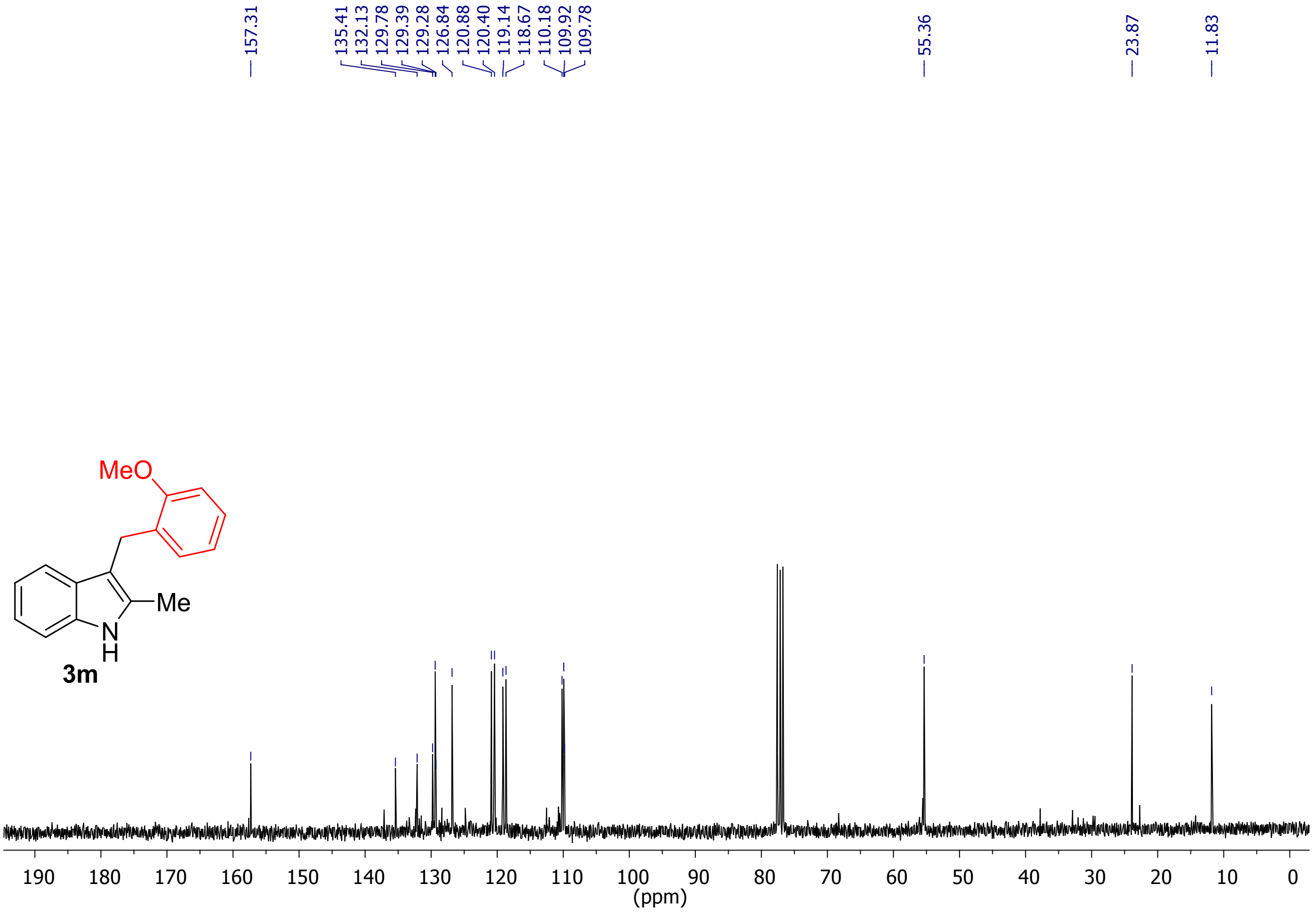

^{1}H NMR

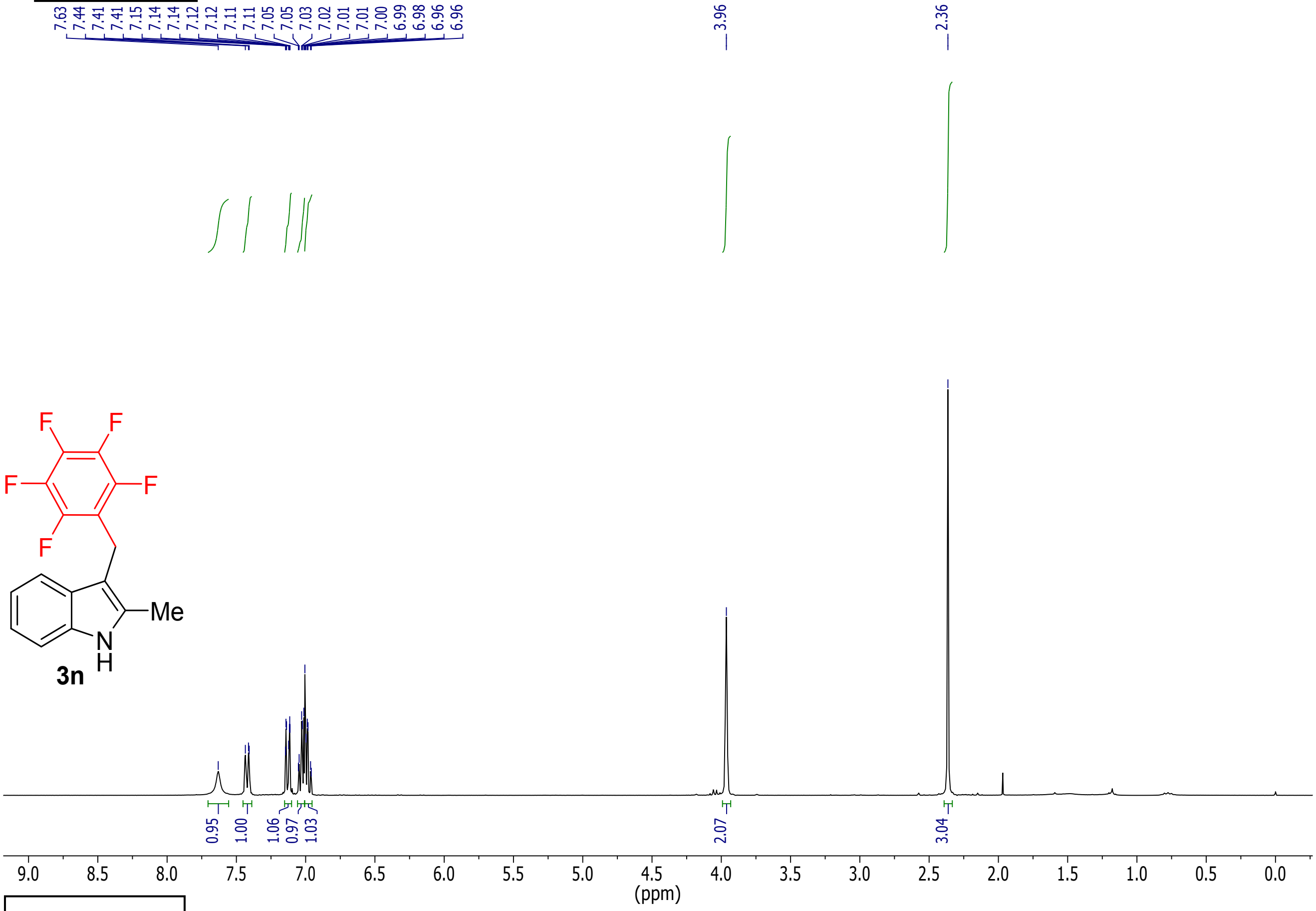

^{13}C NMR

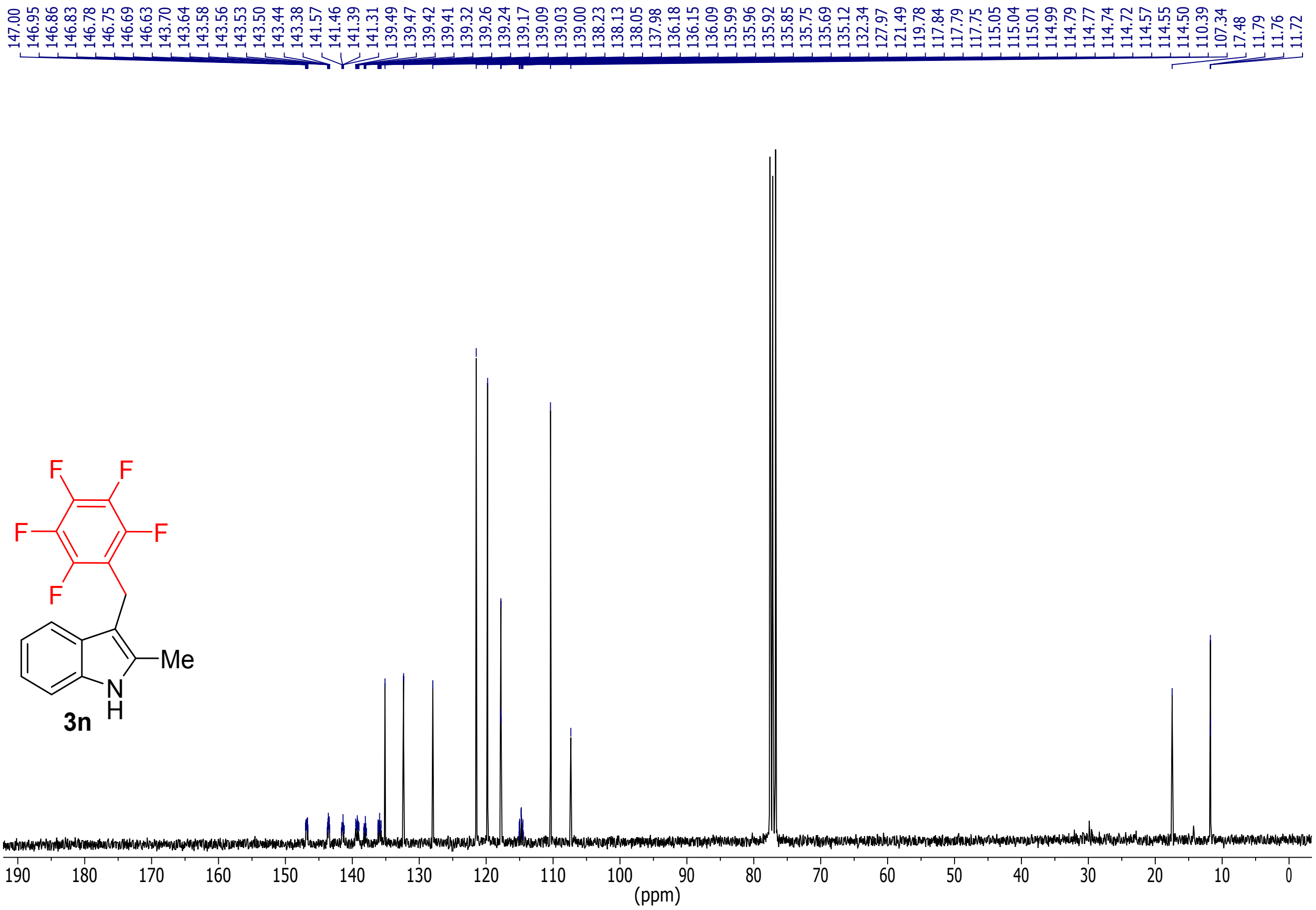

^{19}F NMR

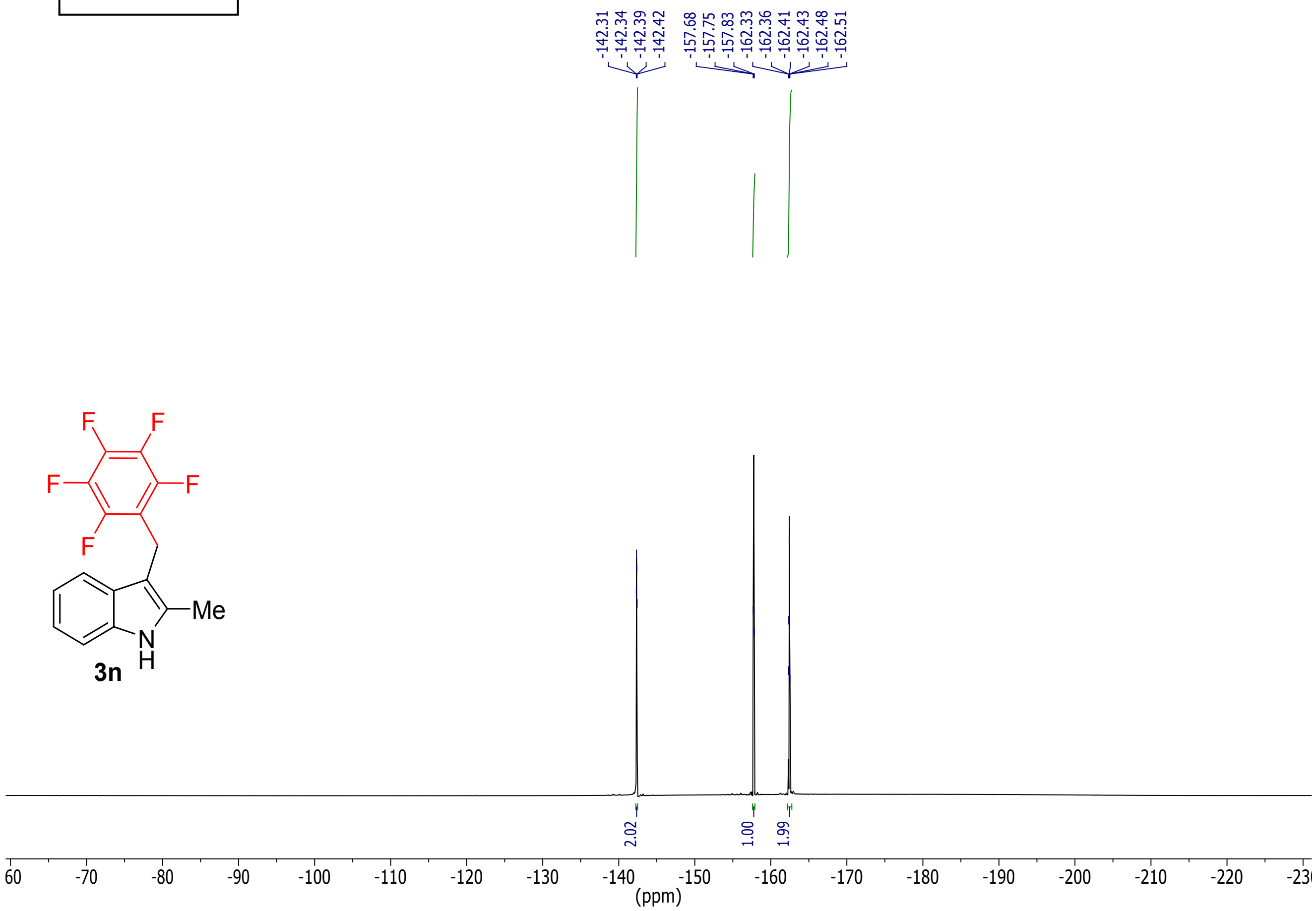

-142.31
-142.34
-142.39
-142.42
-157.68
-157.75
-157.83
-162.33
-162.36
-162.41
-162.43
-162.48
-162.51
F
F
F
F
F
Me
N
H
3n
2.02
1.00
1.99
60
-70
-80
-90
-100
-110
-120
-130
-140
-150
-160
-170
-180
-190
-200
-210
-220
(ppm)

^{1}H NMR

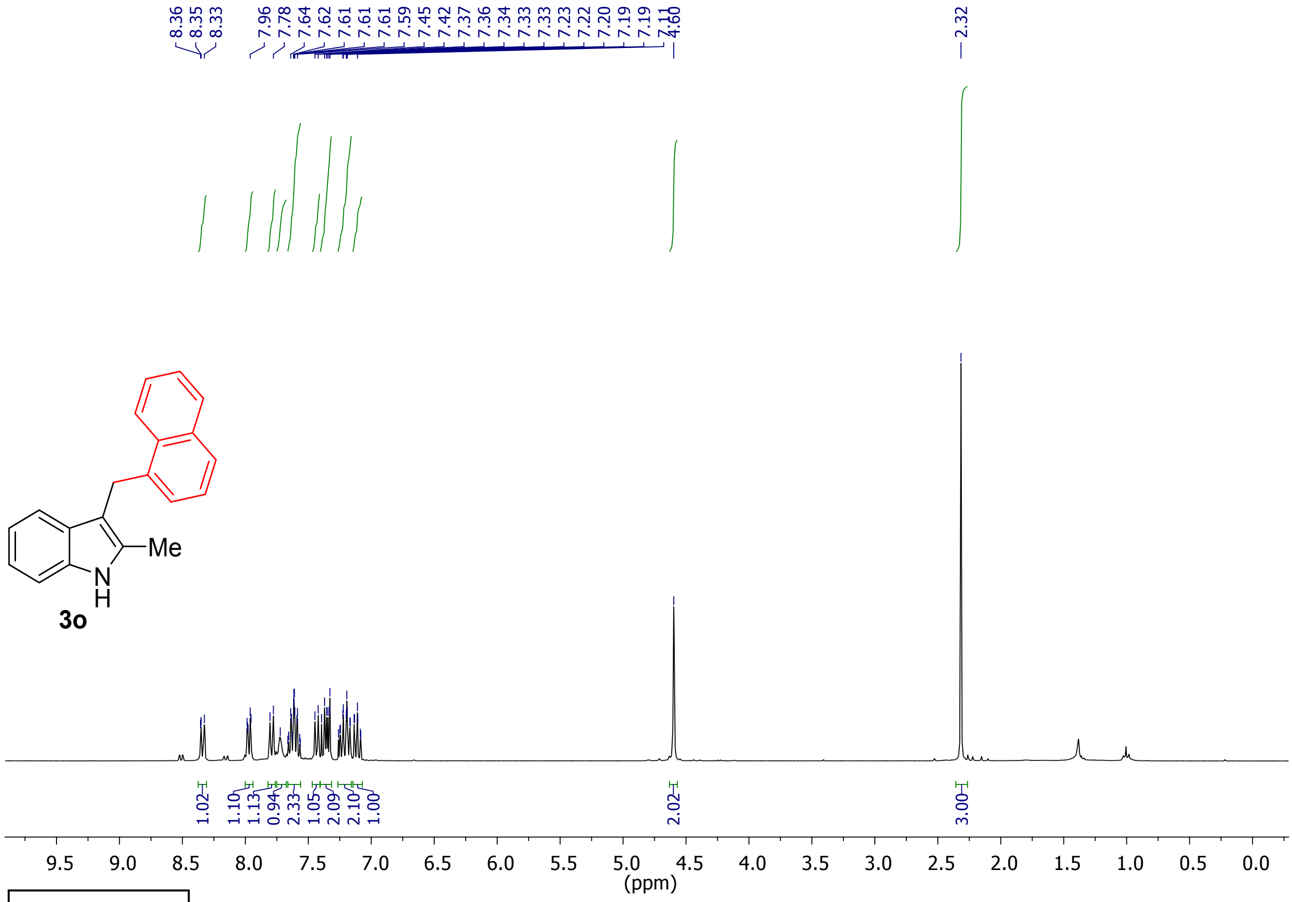

^{13}C NMR

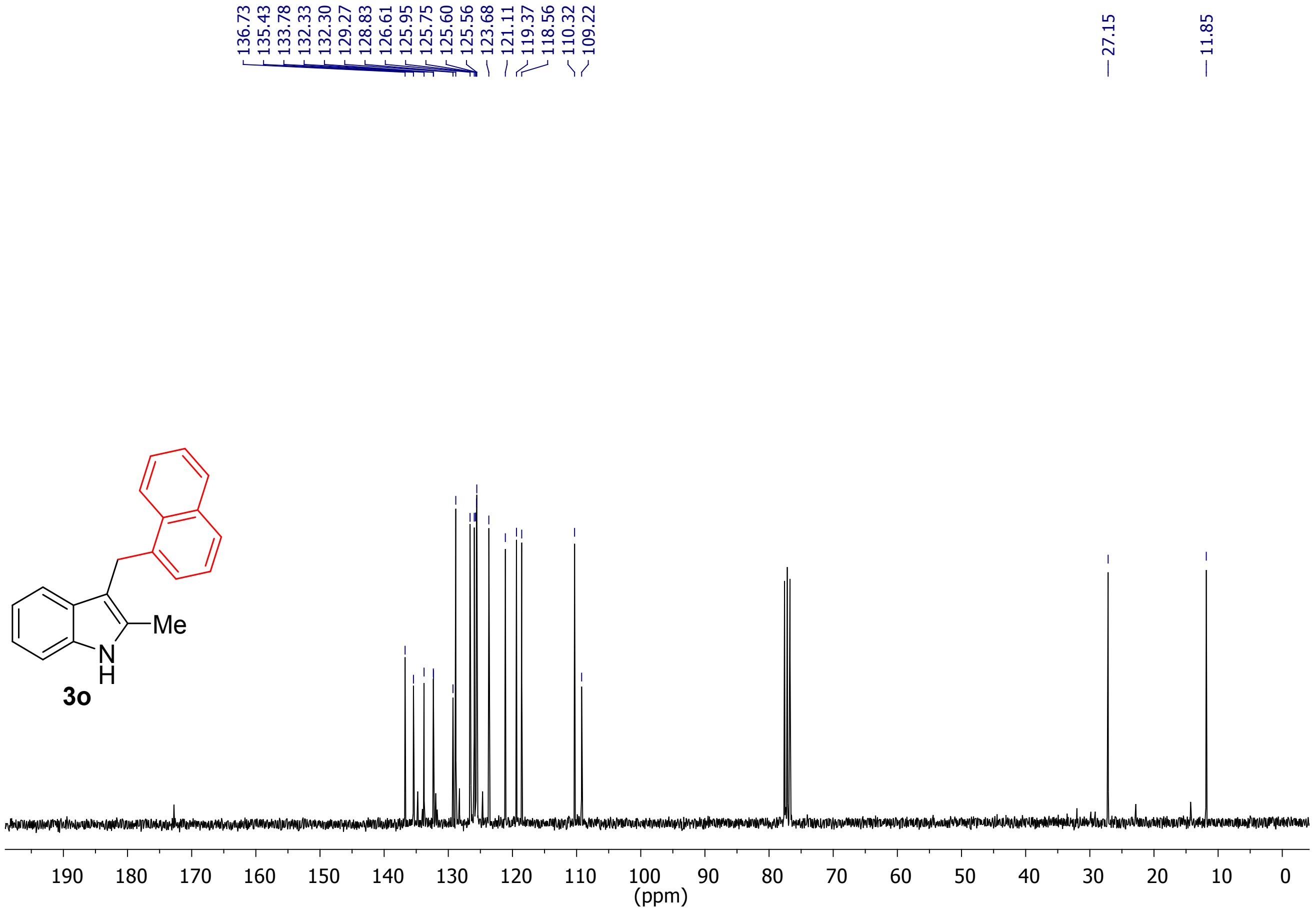

^{1}H NMR

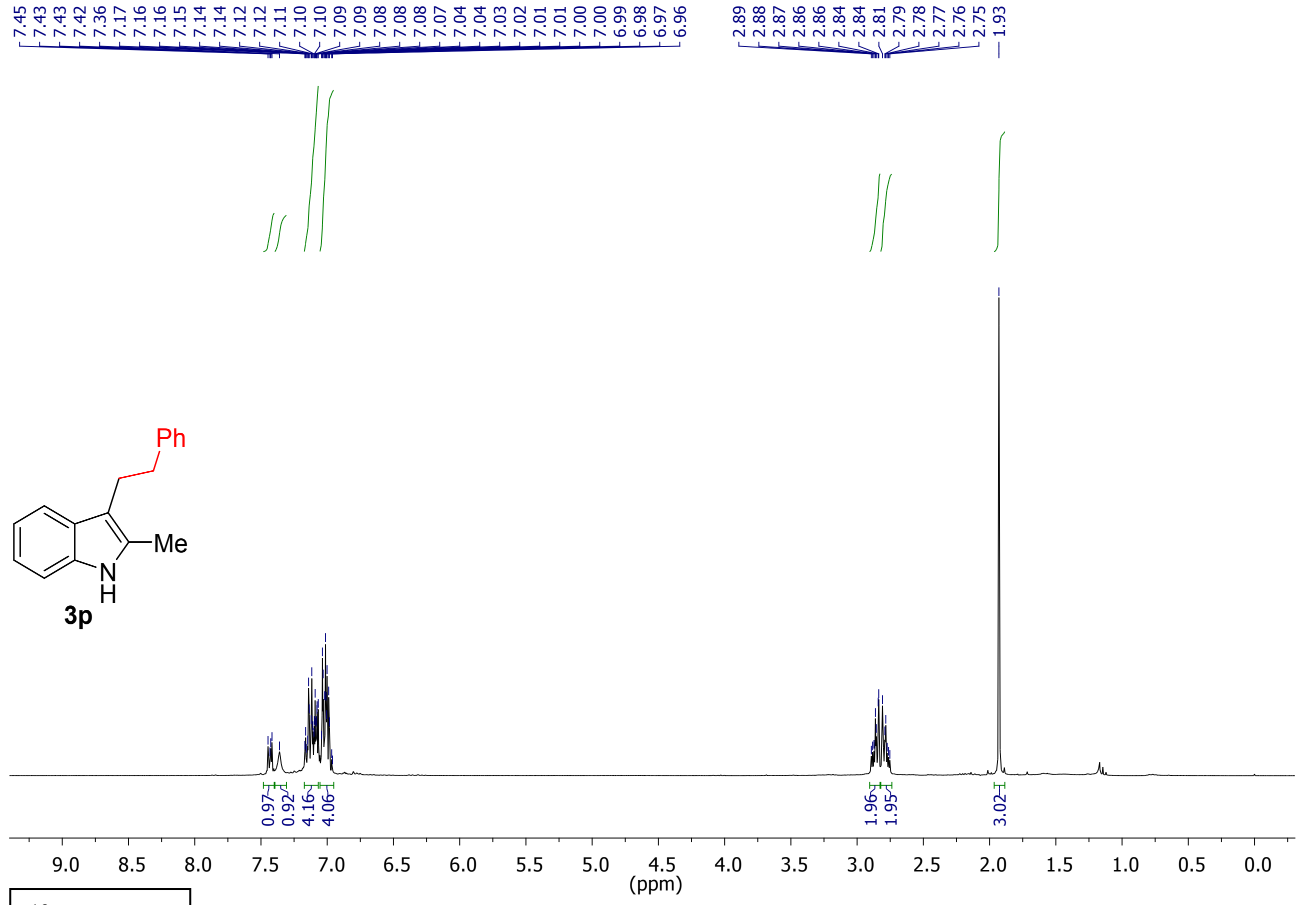

^{13}C NMR

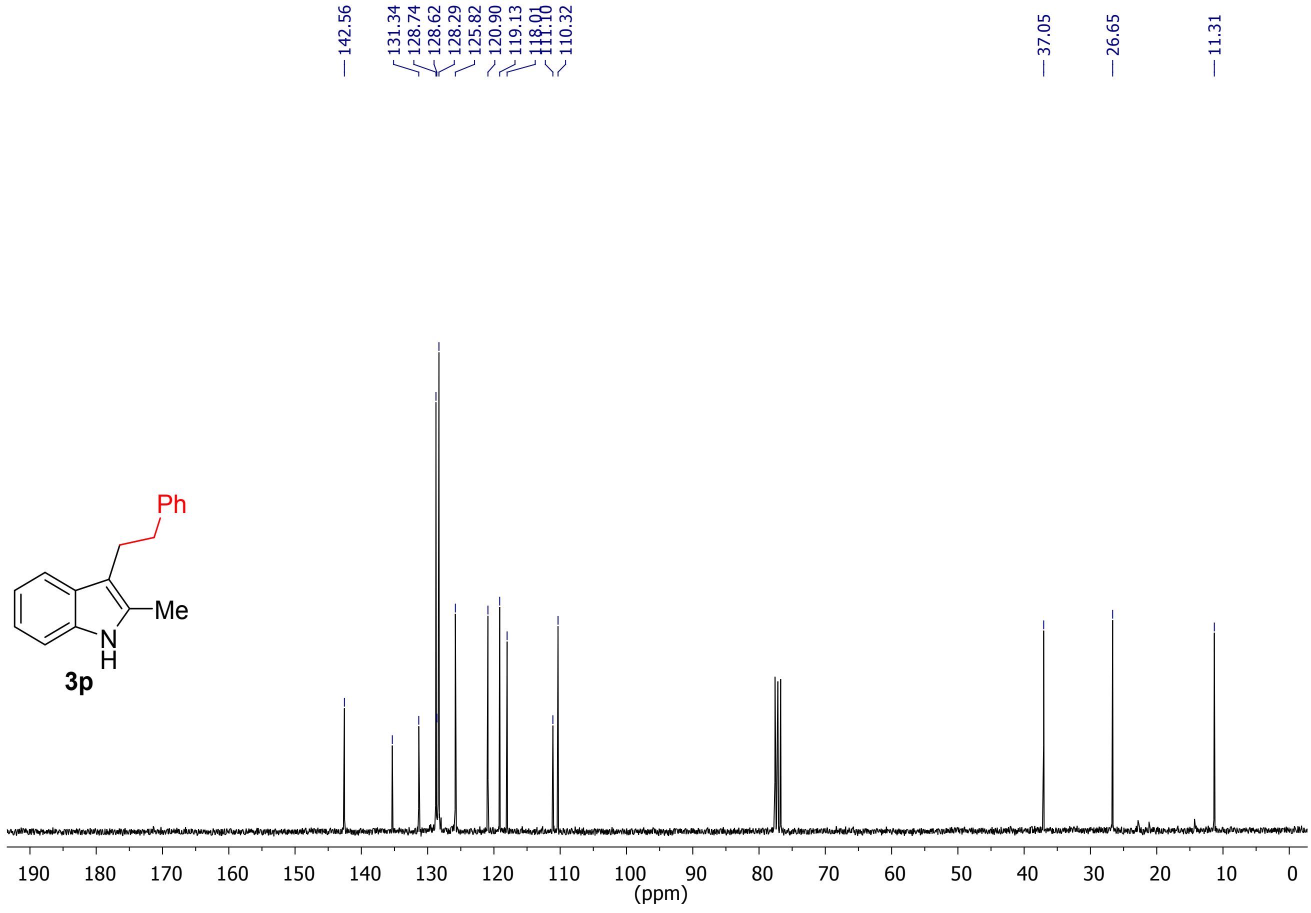

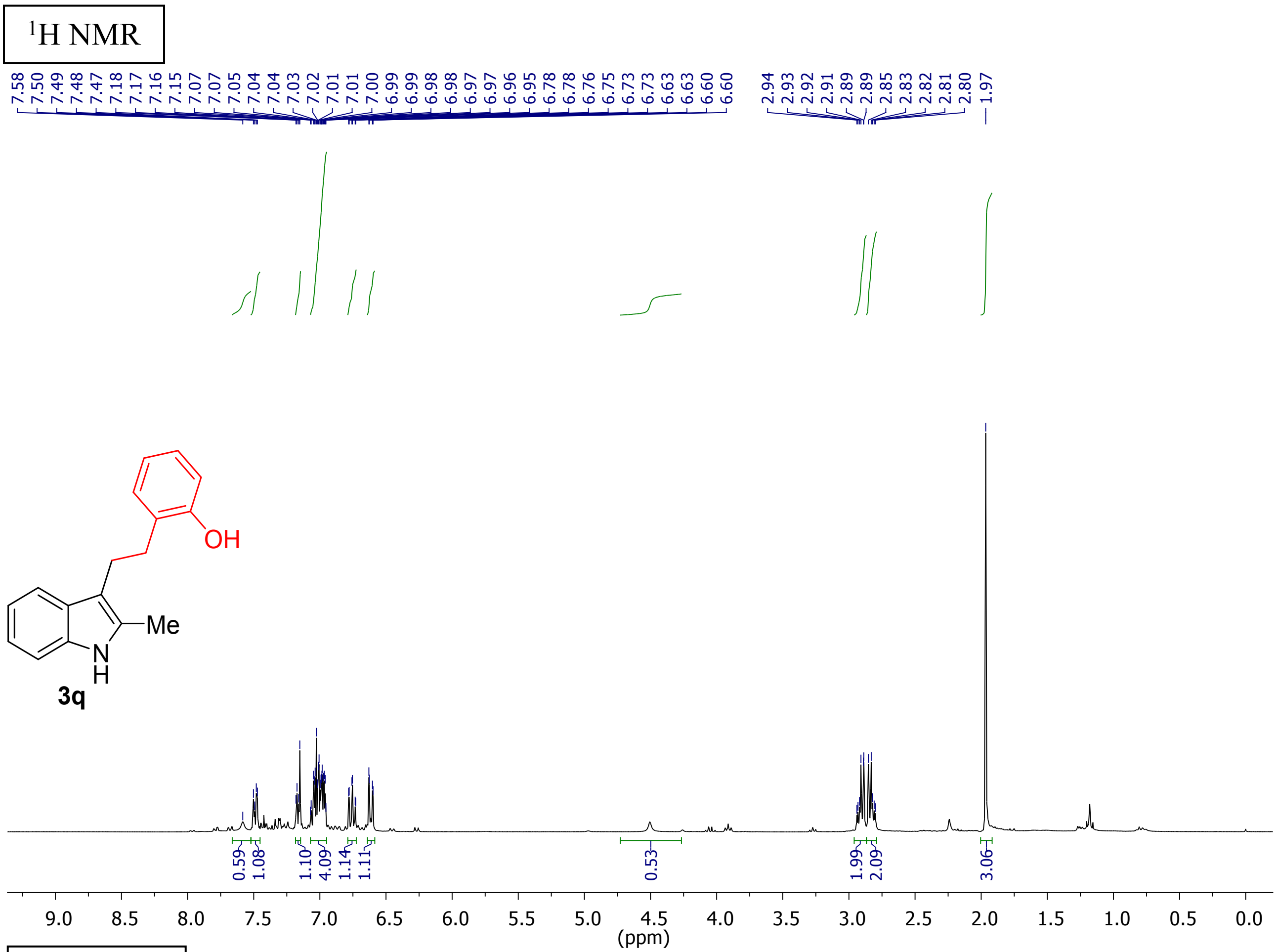

^{1}H NMR
7.58 7.50 7.49 7.48 7.47 7.18 7.17 7.16 7.15 7.07 7.07 7.05 7.04 7.04 7.03 7.02 7.01 7.01 7.00 6.99 6.99 6.98 6.98 6.97 6.97 6.96 6.95 6.78 6.78 6.76 6.75 6.73 6.73 6.63 6.63 6.60 6.60
2.94 2.93 2.92 2.91 2.89 2.89 2.85 2.83 2.82 2.81 2.80
1.97
OH
Me
N
H
3q
0.59 1.08 1.10 4.09 1.14 1.11 0.53 1.99 2.09 3.06
9.0 8.5 8.0 7.5 7.0 6.5 6.0 5.5 5.0 4.5 4.0 3.5 3.0 2.5 2.0 1.5 1.0 0.5 0.0
(ppm)

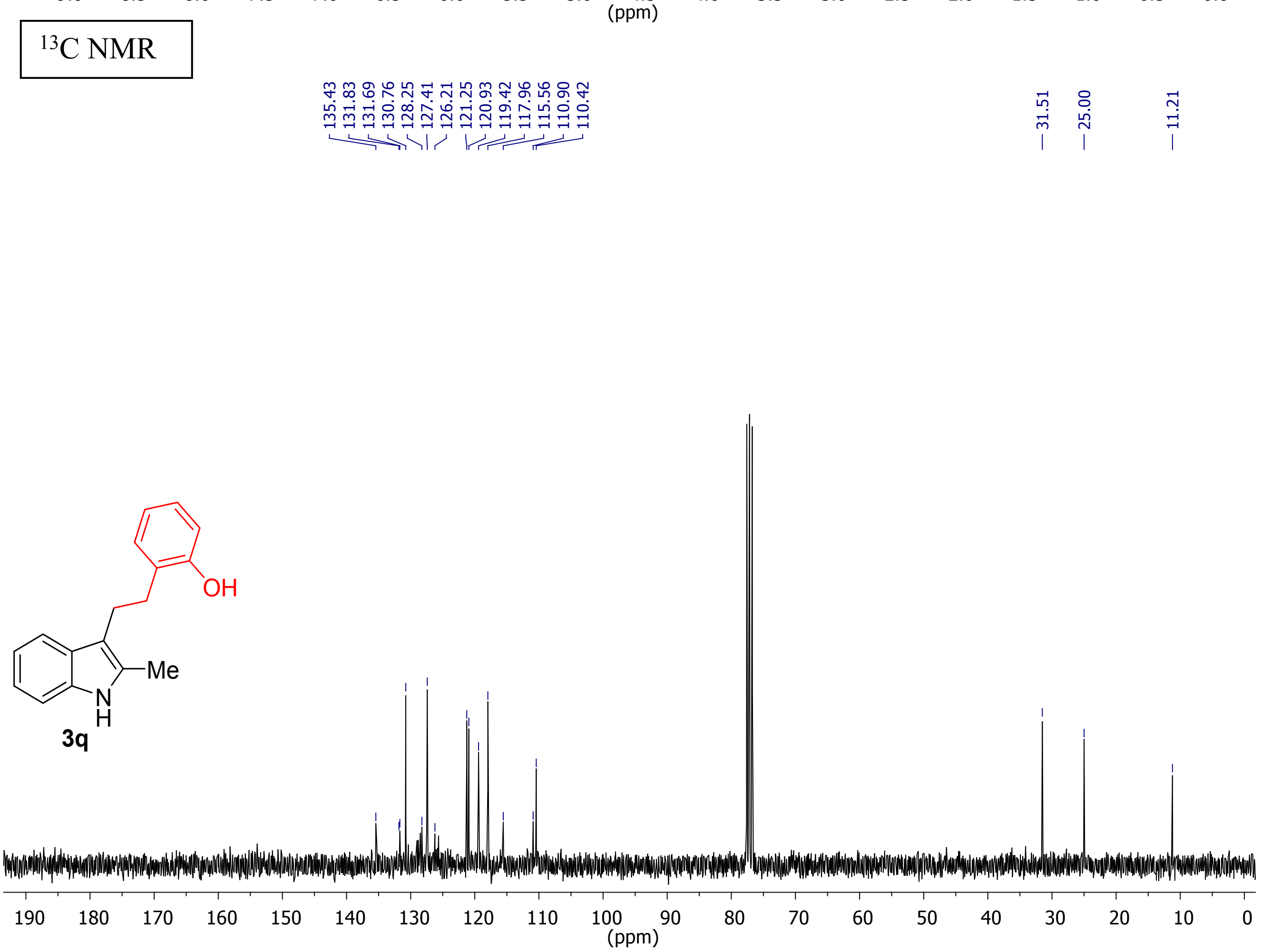

^{13}C NMR
135.43 131.83 131.69 130.76 128.25 127.41 126.21 121.25 120.93 119.42 117.96 115.56 110.90 110.42
31.51
25.00
11.21
OH
Me
N
H
3q
190 180 170 160 150 140 130 120 110 100 90 80 70 60 50 40 30 20 10 0
(ppm)

¹H NMR

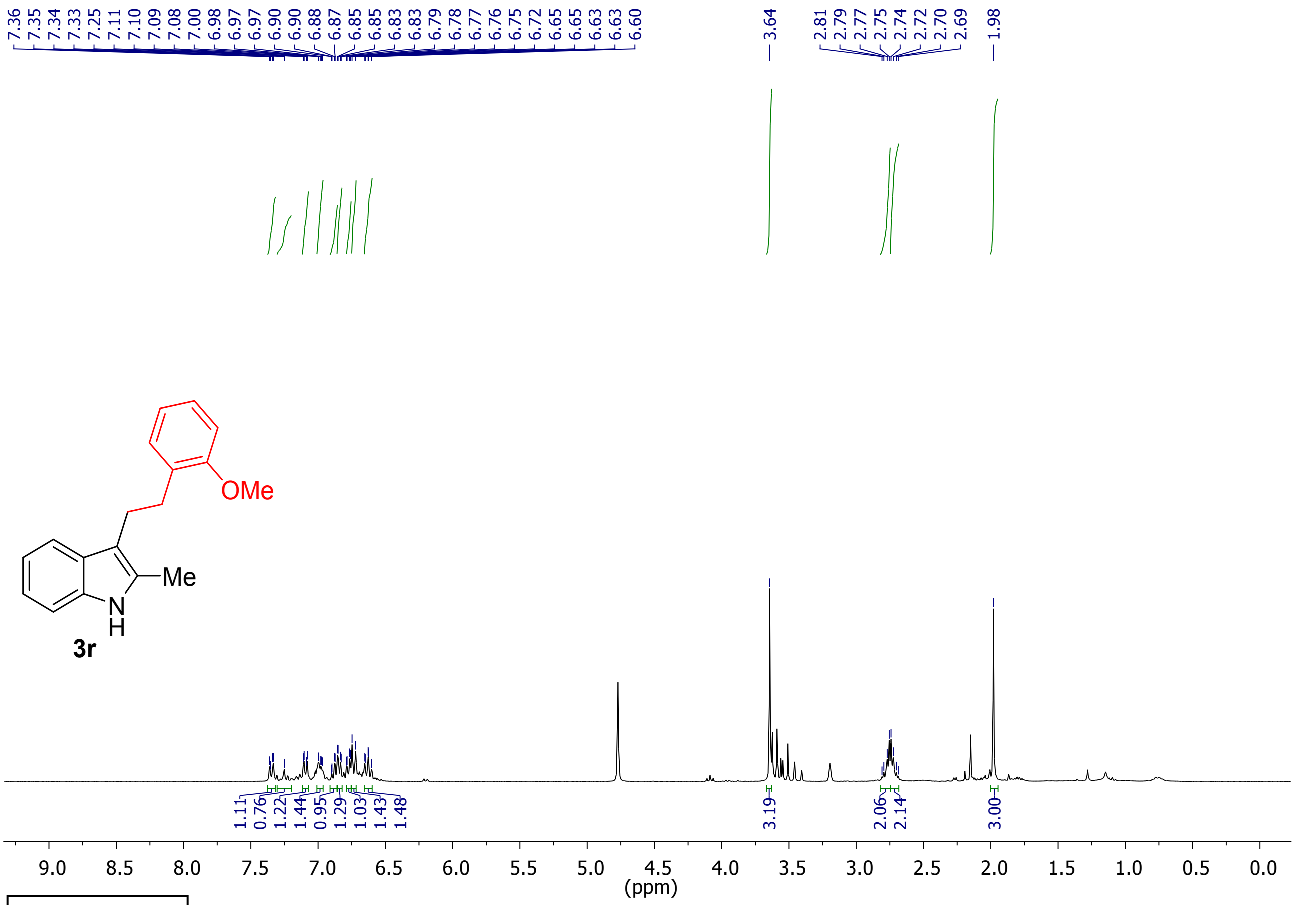

¹³C NMR

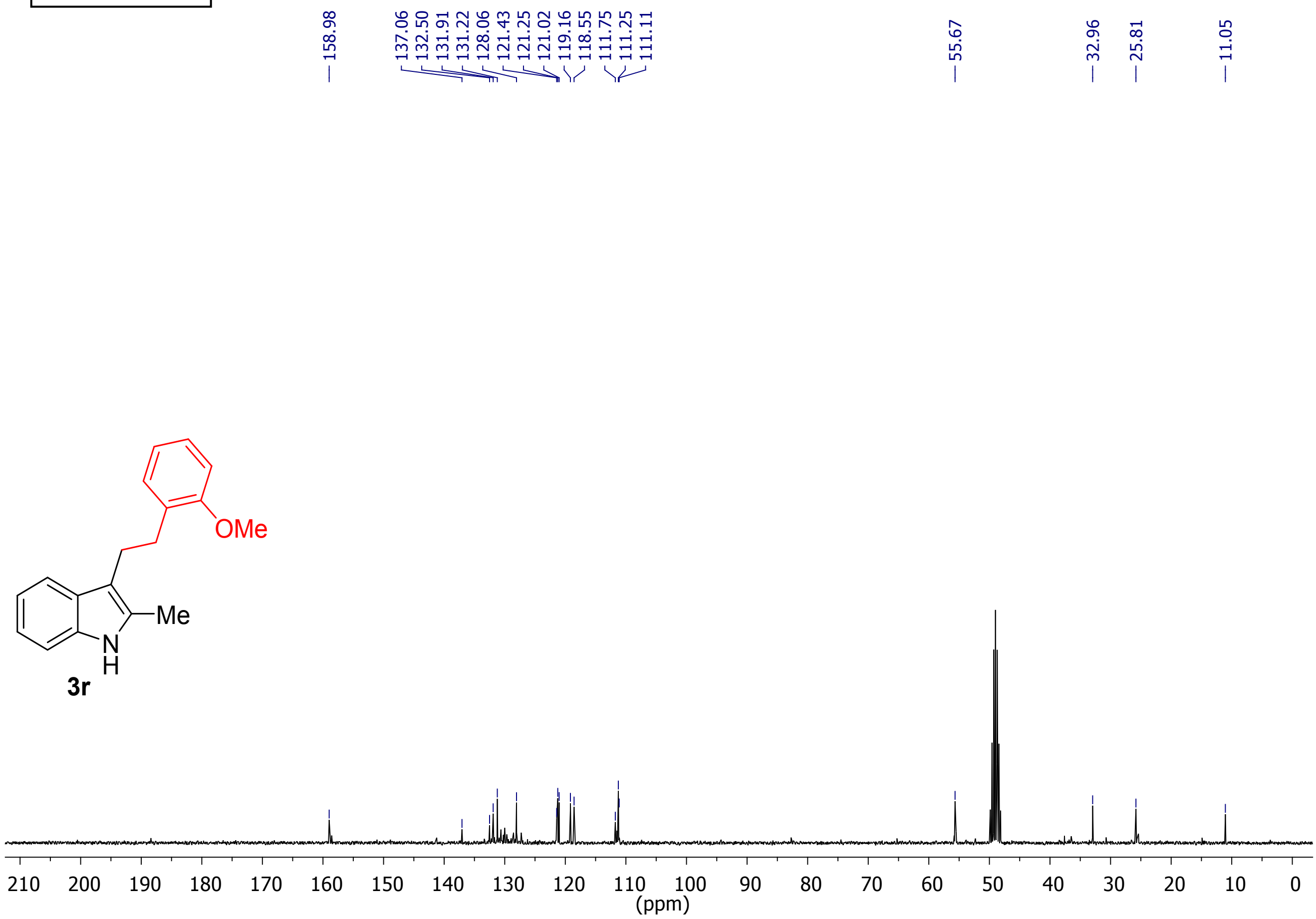

^{1}H NMR

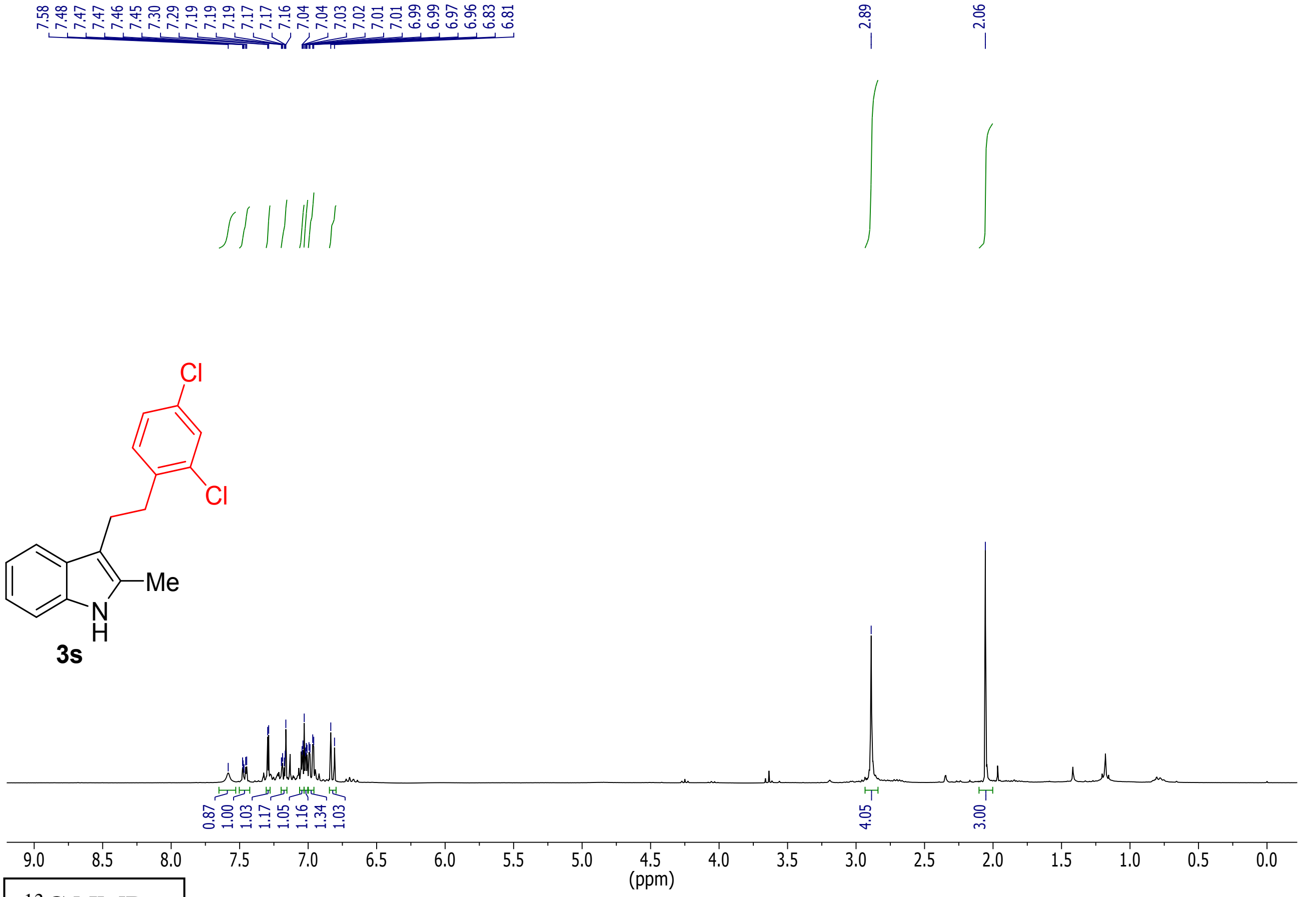

^{13}C NMR

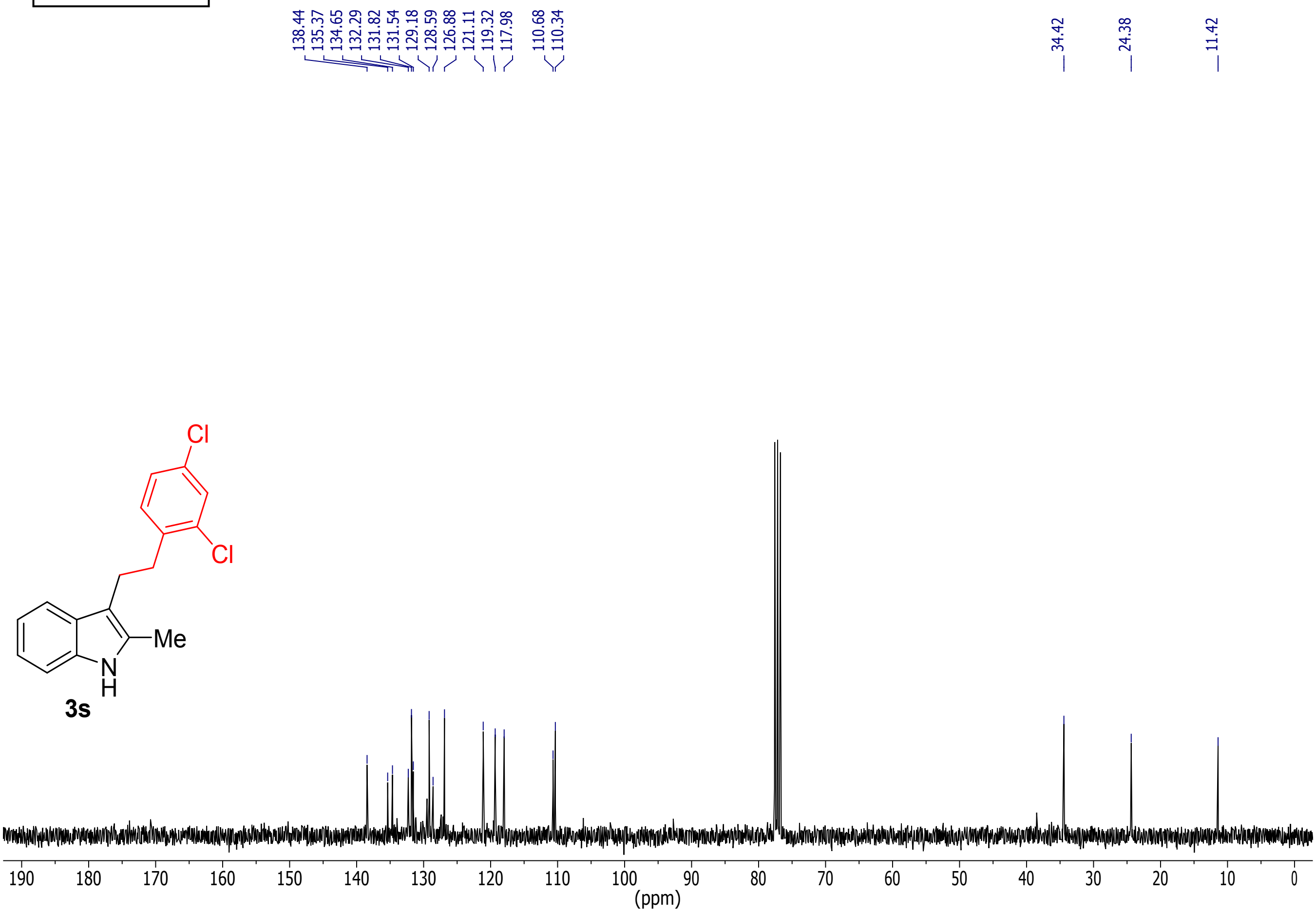

^{1}H NMR

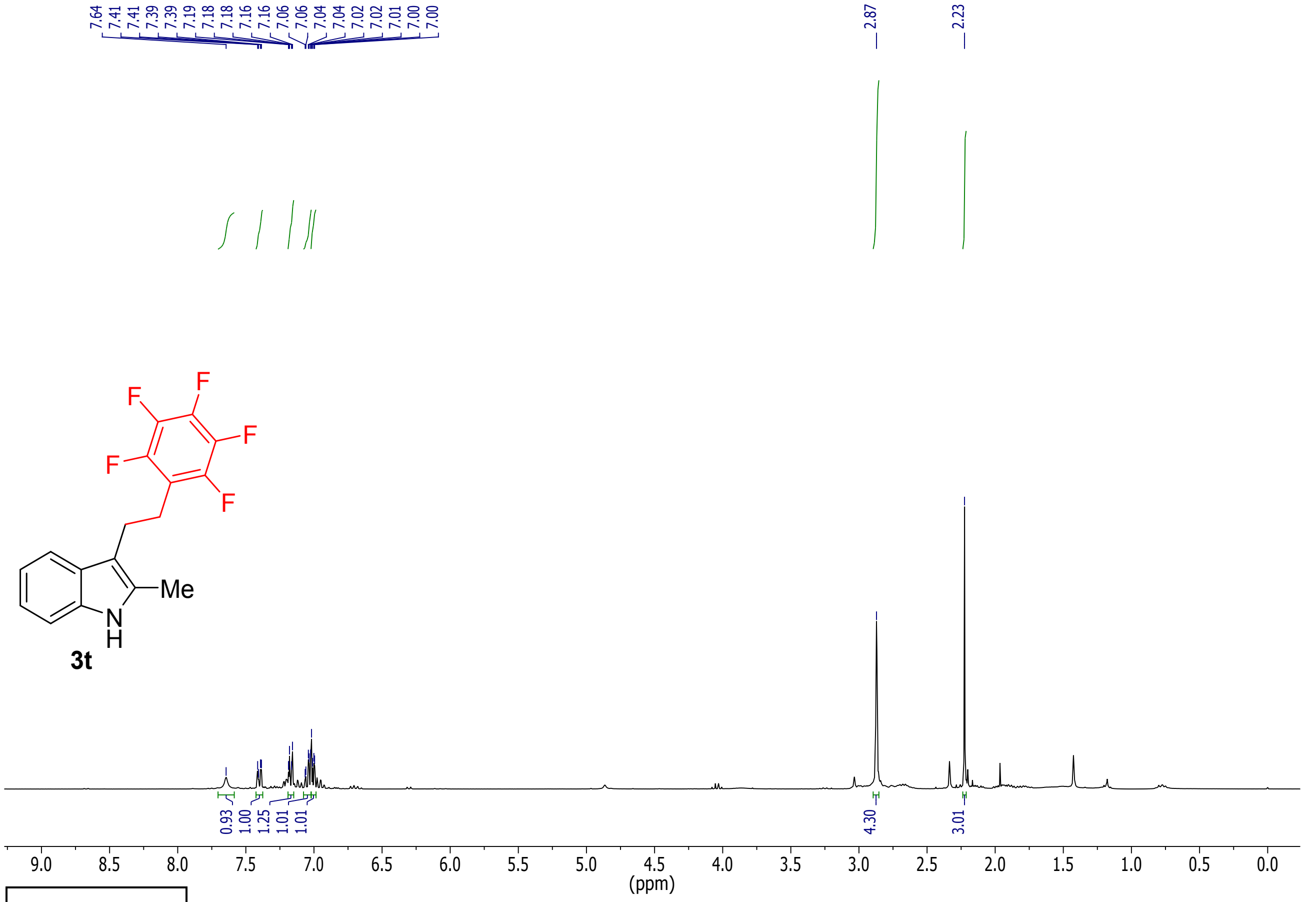

^{13}C NMR

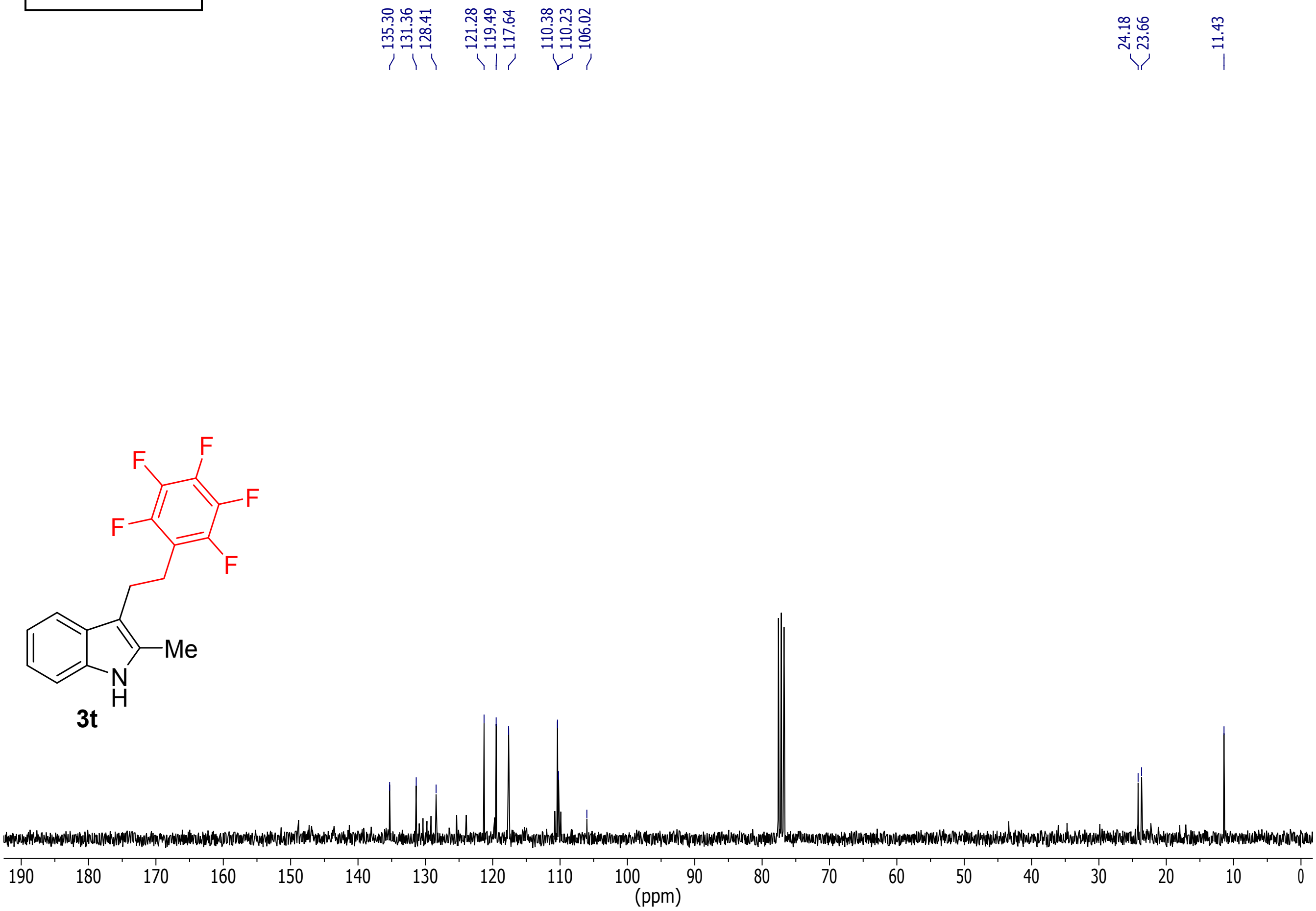

^{19}F NMR

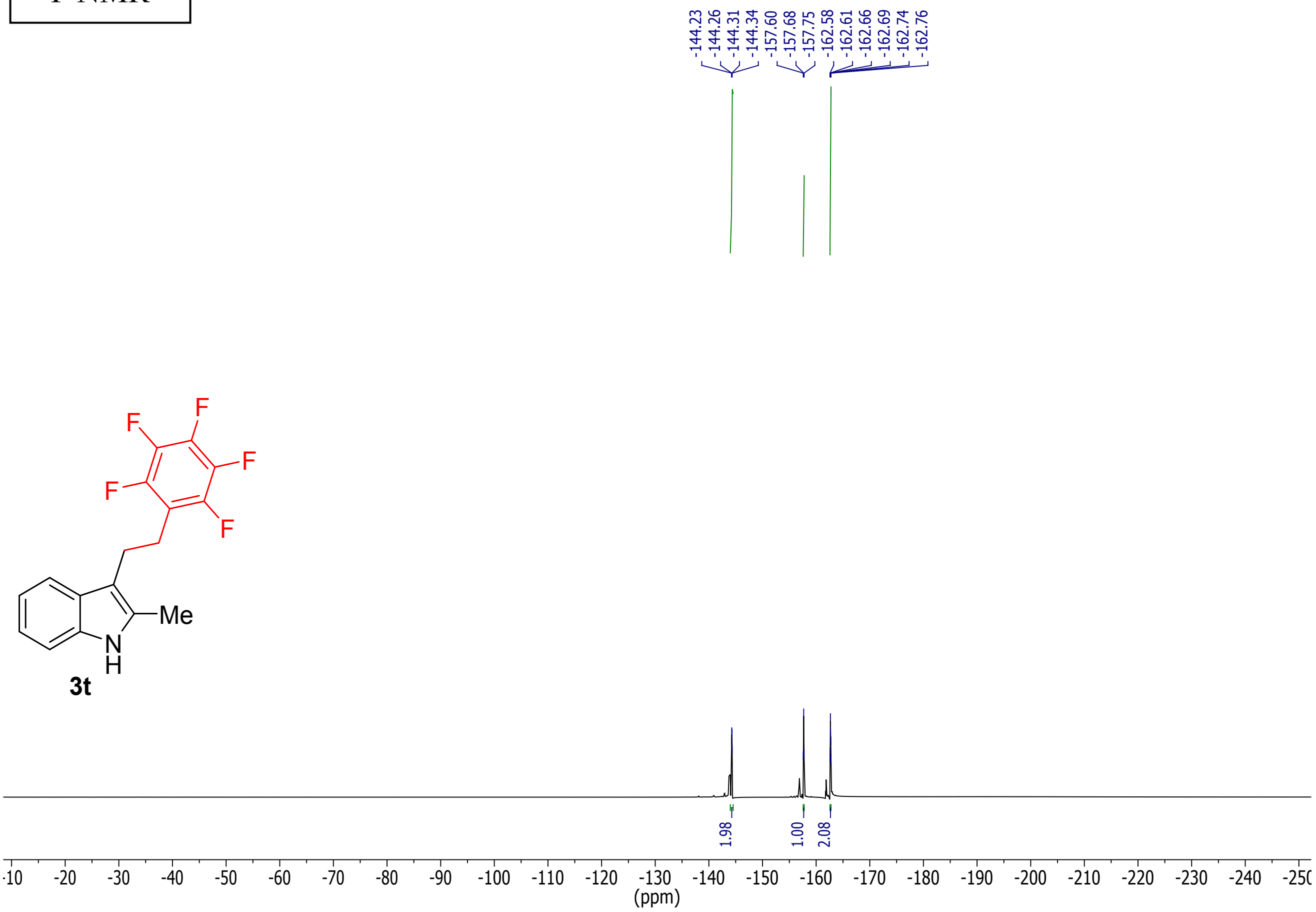
-144.23
-144.26
-144.31
-144.34
-157.60
-157.68
-157.75
-162.58
-162.61
-162.66
-162.69
-162.74
-162.76
1.98
1.00
2.08
-10 -20 -30 -40 -50 -60 -70 -80 -90 -100 -110 -120 -130 -140 -150 -160 -170 -180 -190 -200 -210 -220 -230 -240 -250
(ppm)
F
F
F
F
F
Me
N
H

3t

^1H NMR

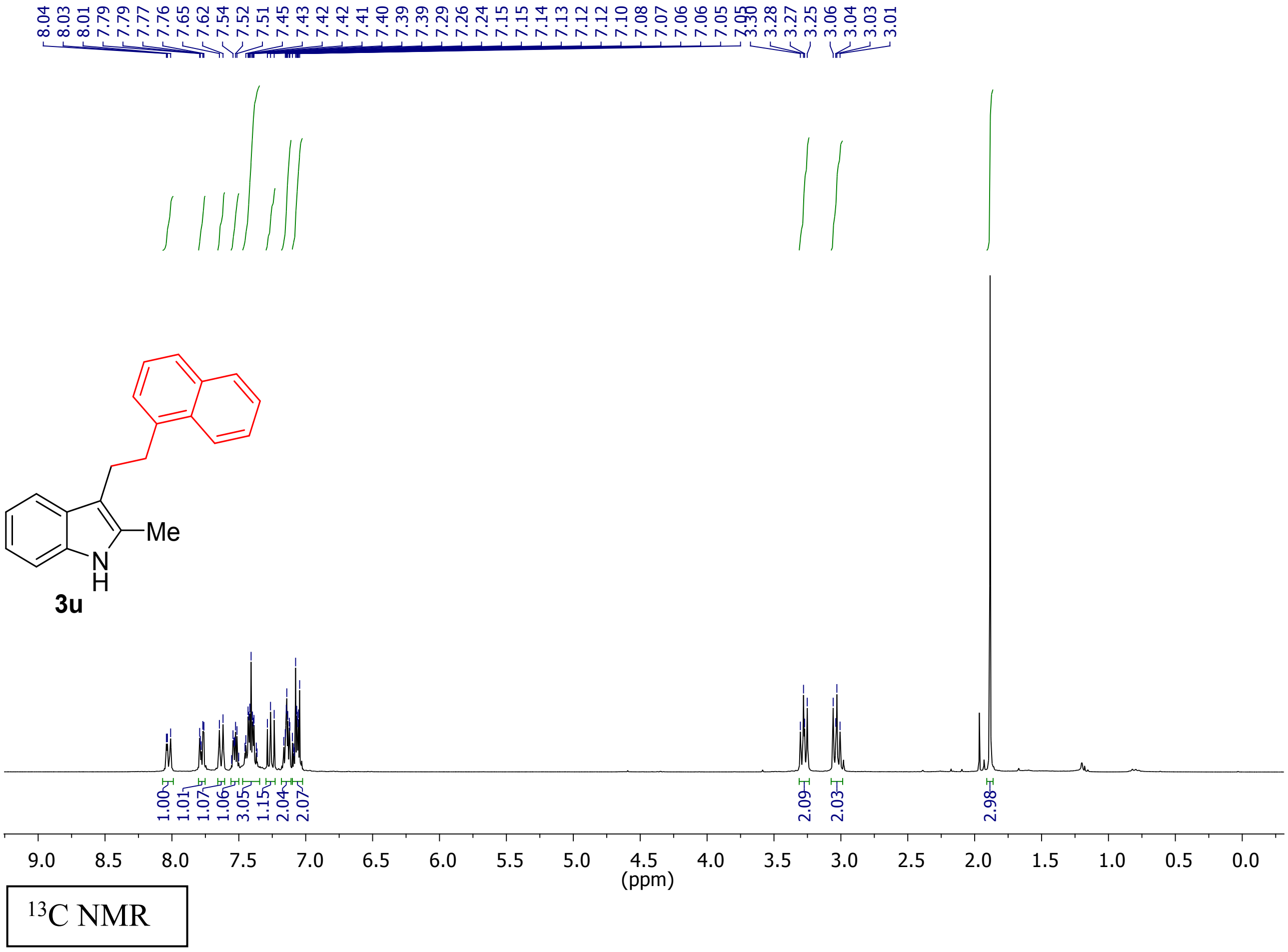

^{13}C NMR

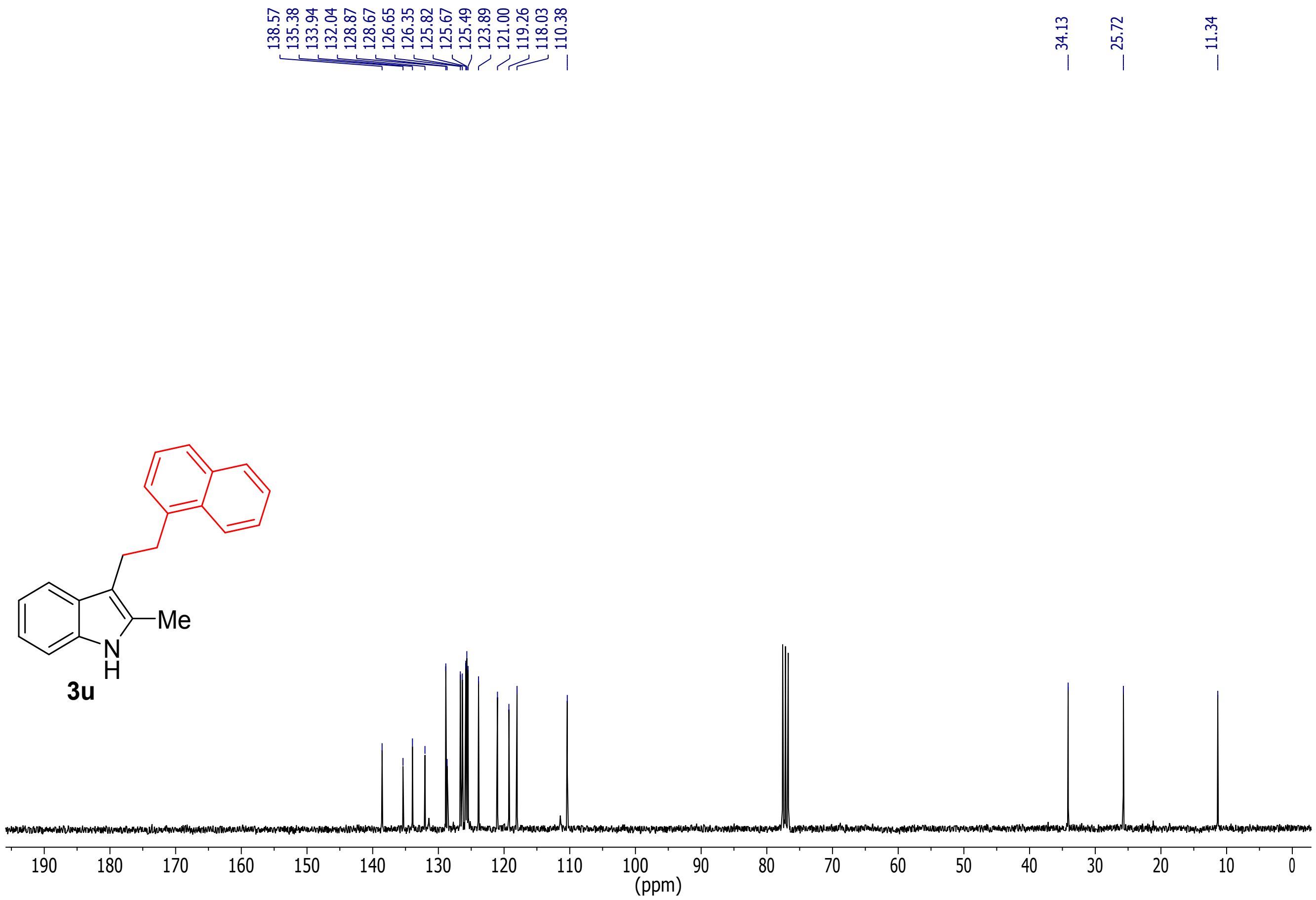

^{1}H NMR

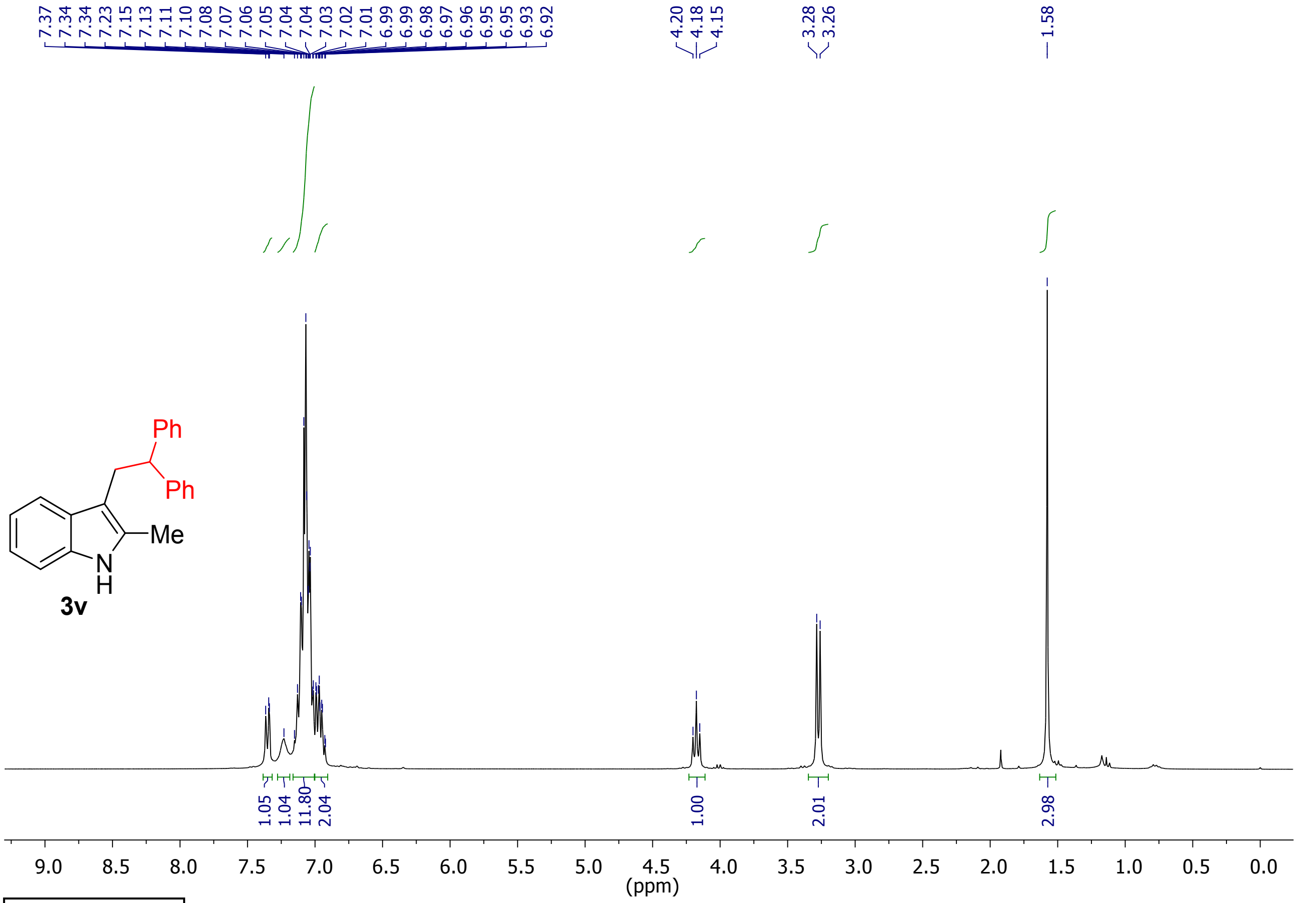

^{13}C NMR

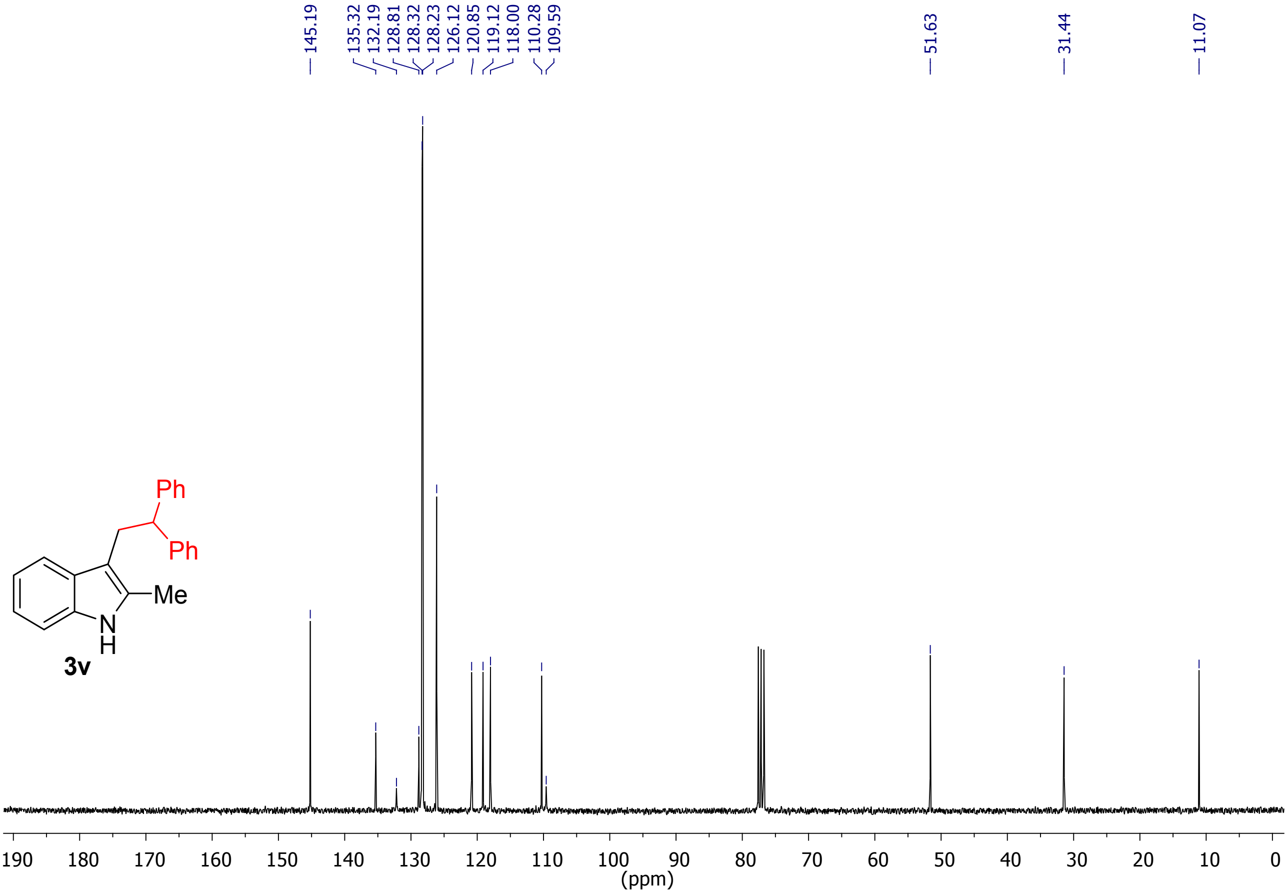
145.19
135.32
132.19
128.81
128.32
128.23
126.12
120.85
119.12
118.00
110.28
109.59
51.63
31.44
11.07
Ph
Ph
Me
N
H
3v
190 180 170 160 150 140 130 120 110 100 90 80 70 60 50 40 30 20 10 0
(ppm)

^{1}H NMR

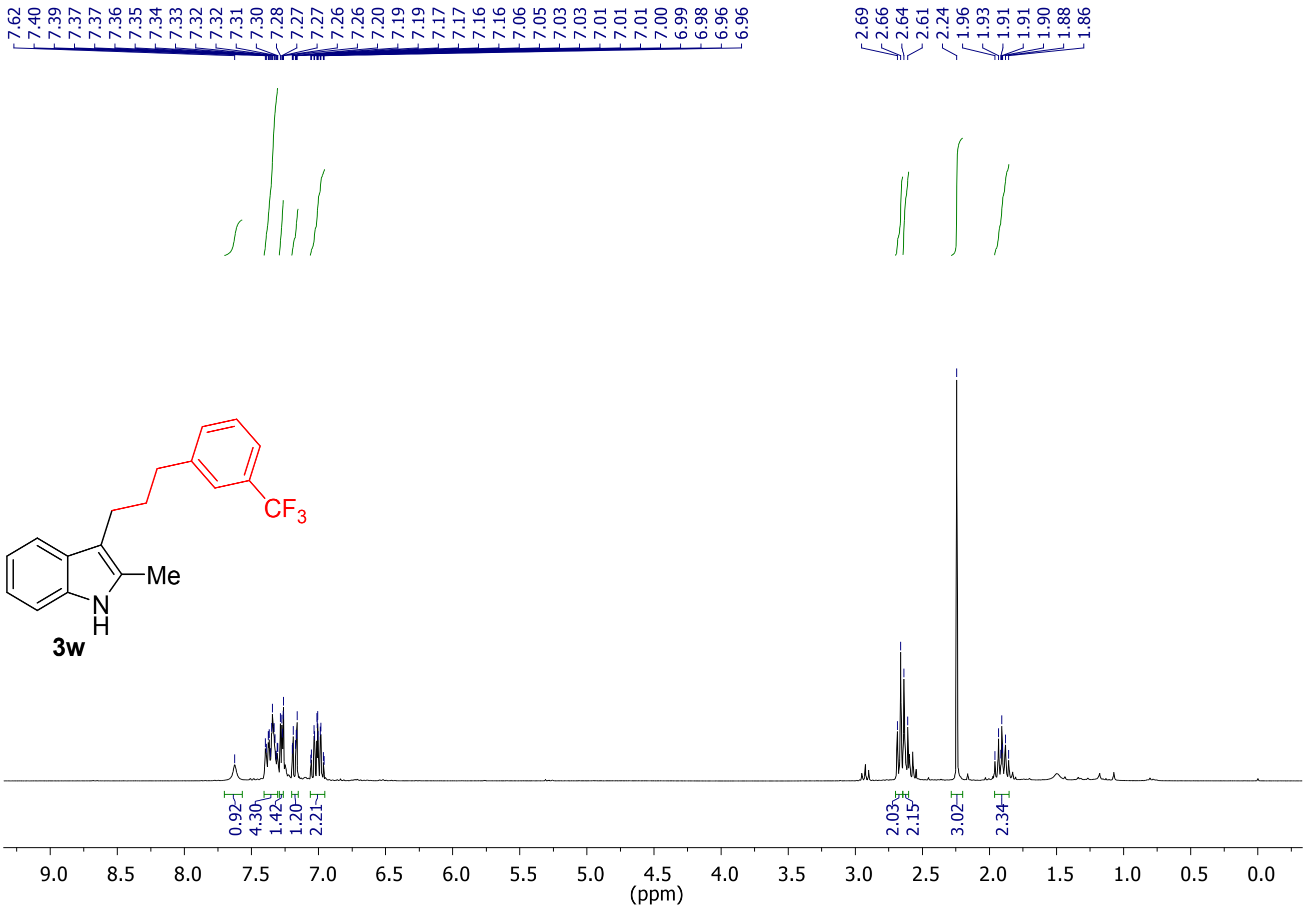

^{13}C NMR

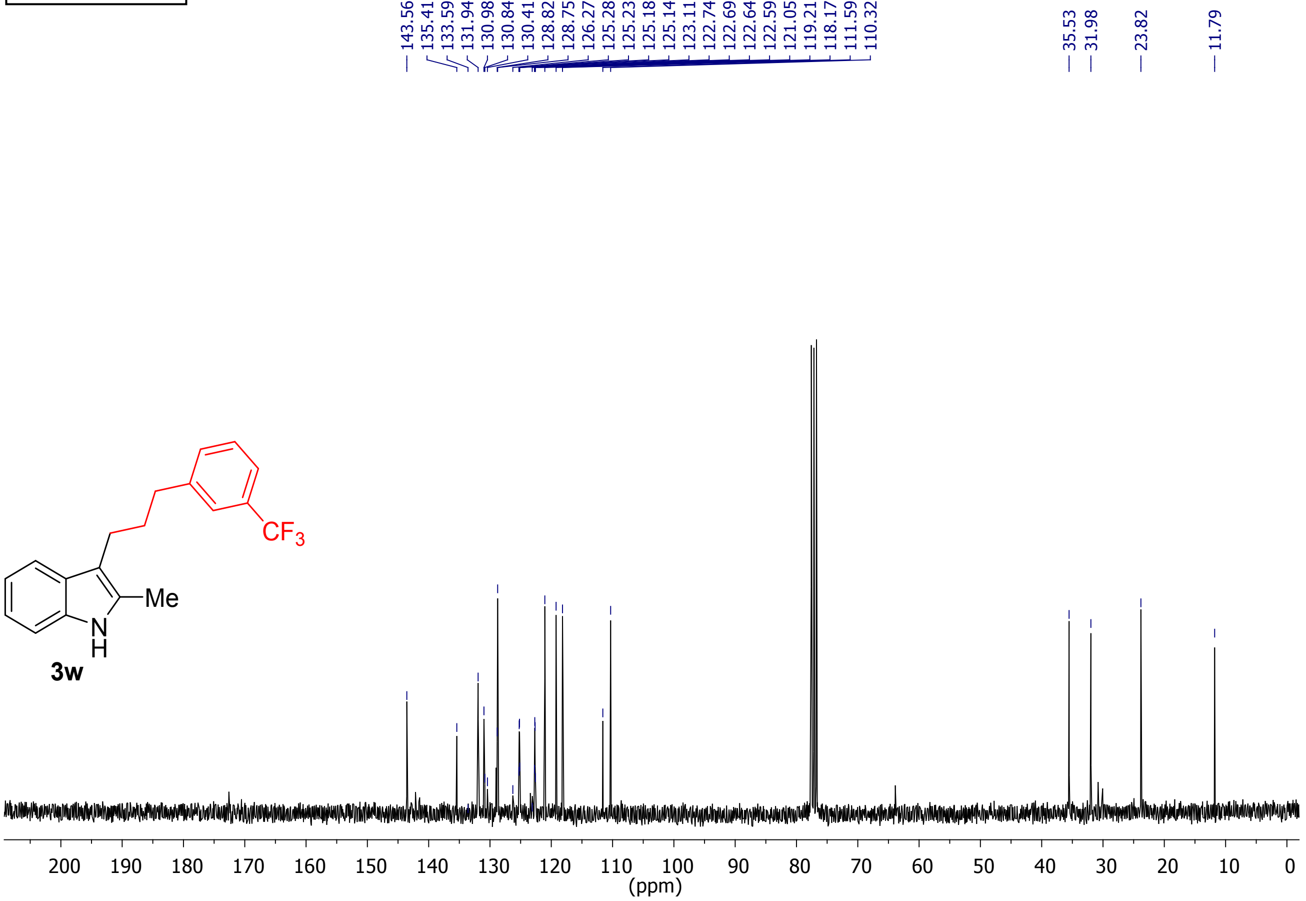

^{19}F NMR

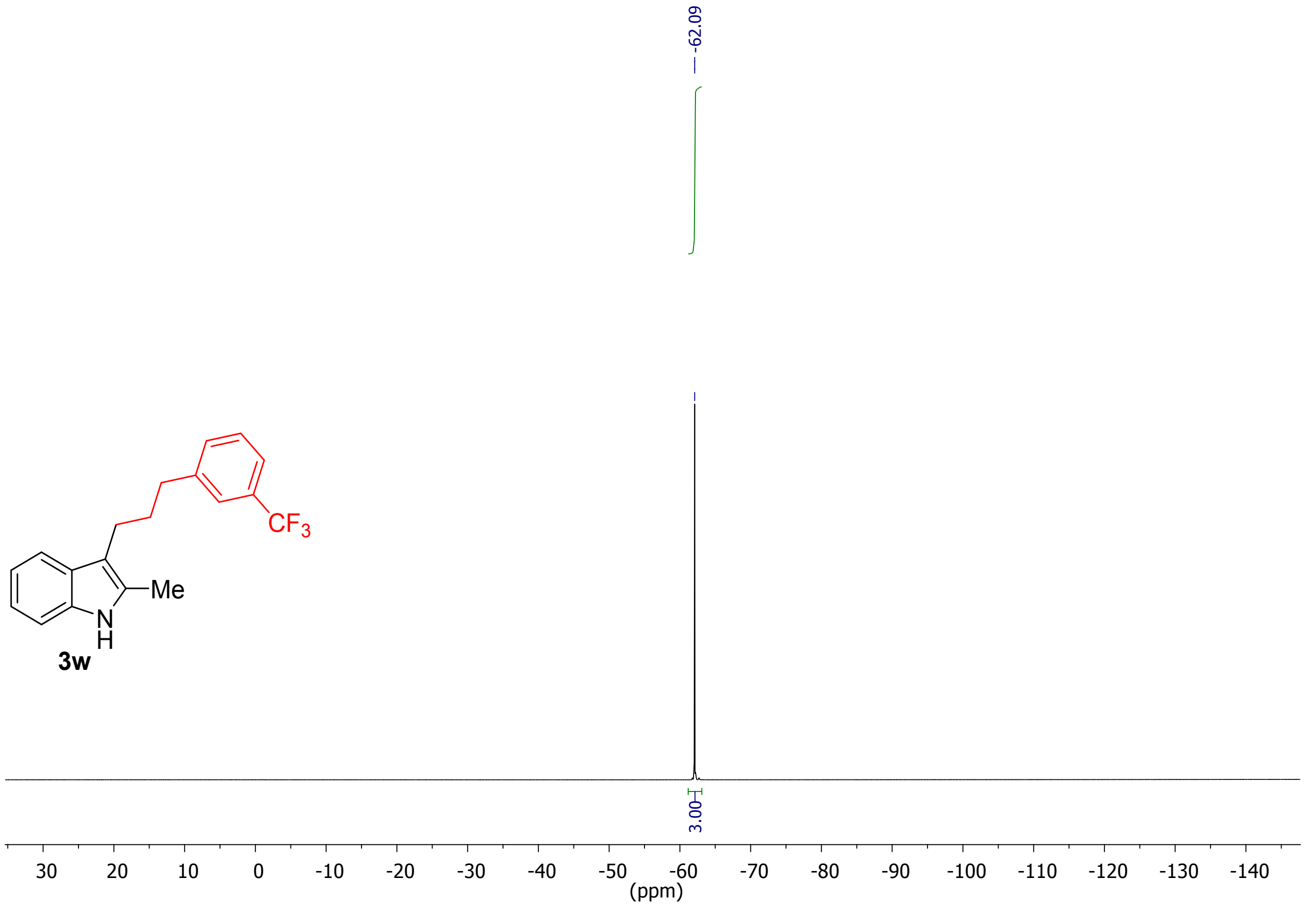

-62.09
CF3
Me
N
H
3w
3.00
30 20 10 0 -10 -20 -30 -40 -50 -60 -70 -80 -90 -100 -110 -120 -130 -140
(ppm)

^{1}H NMR

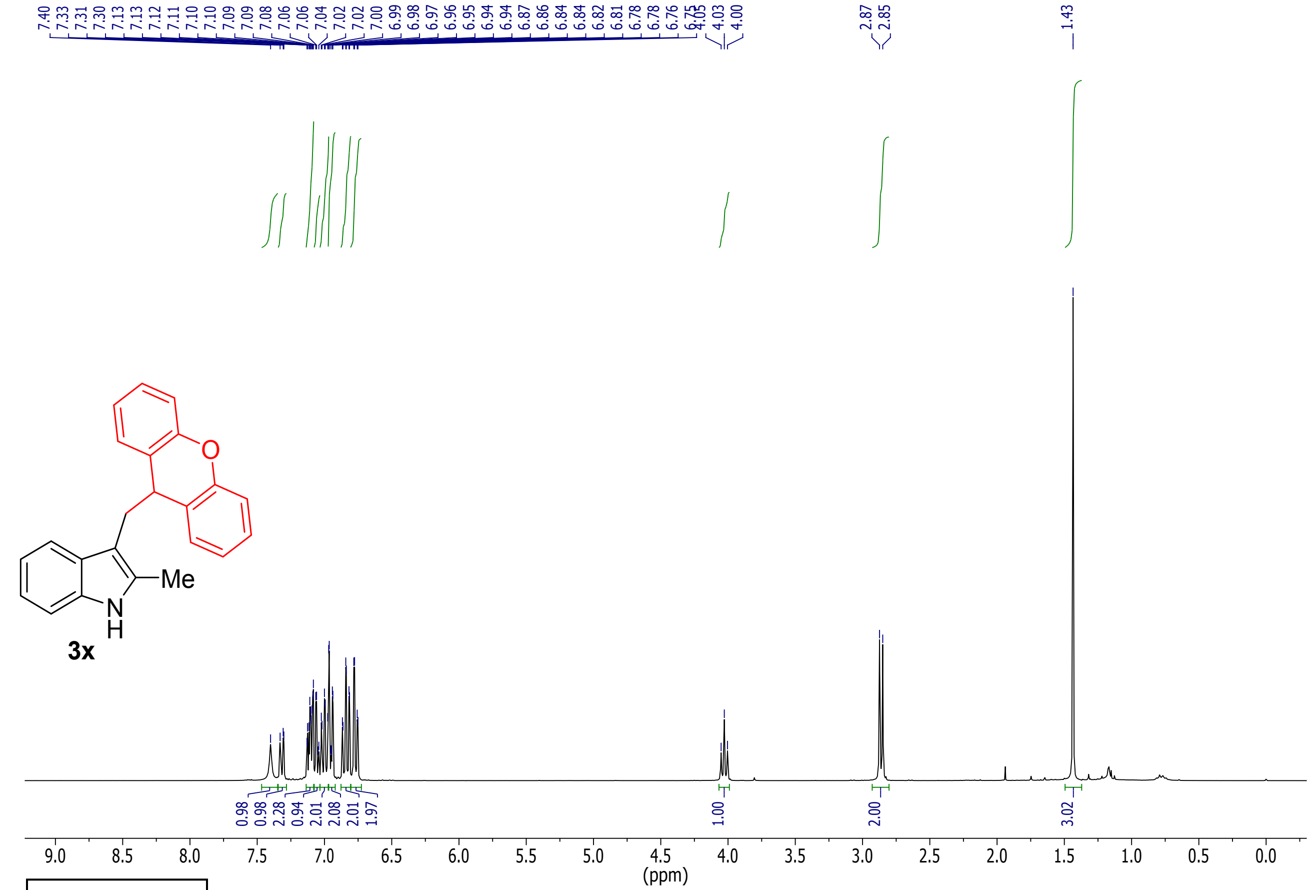

^{13}C NMR

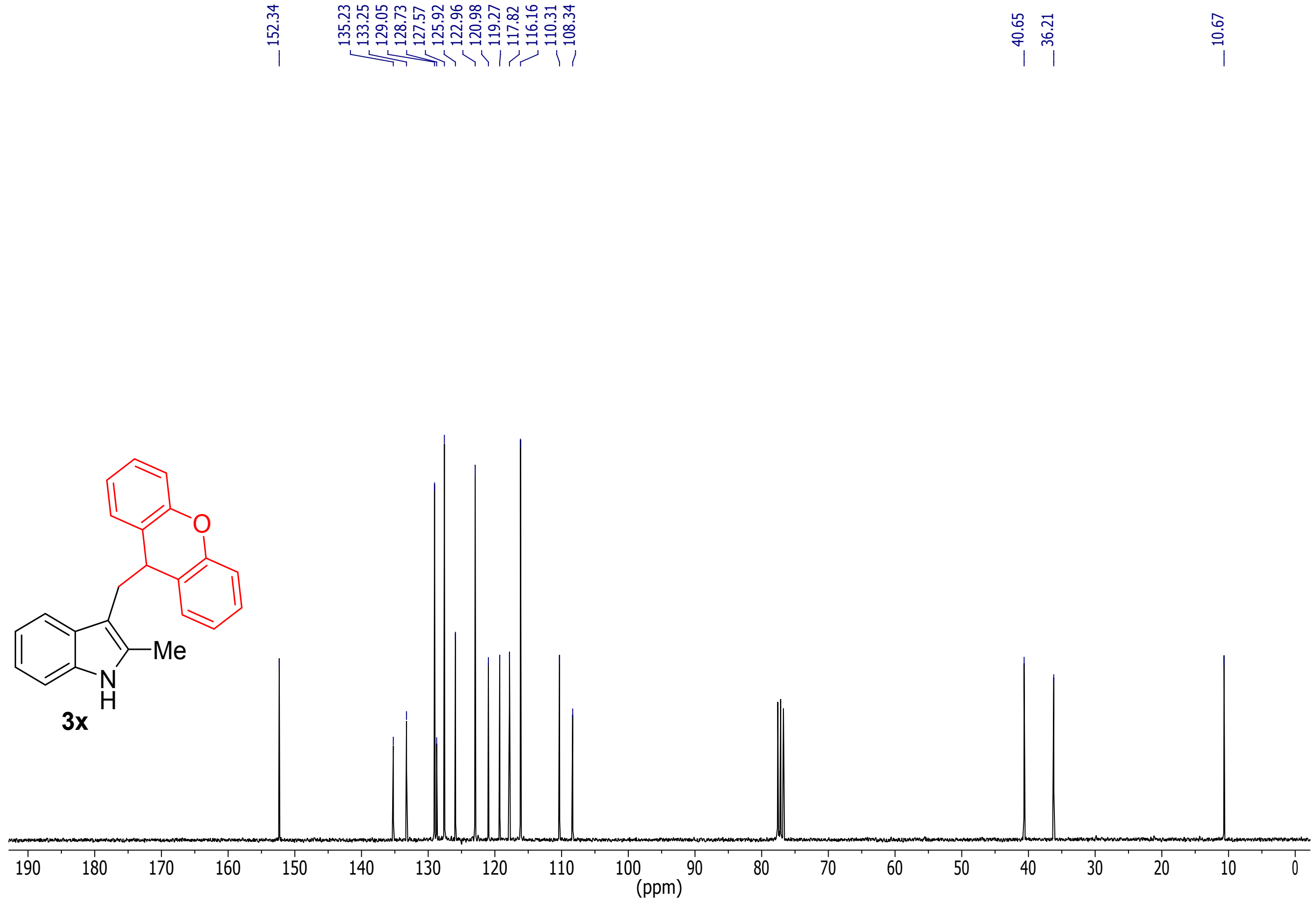

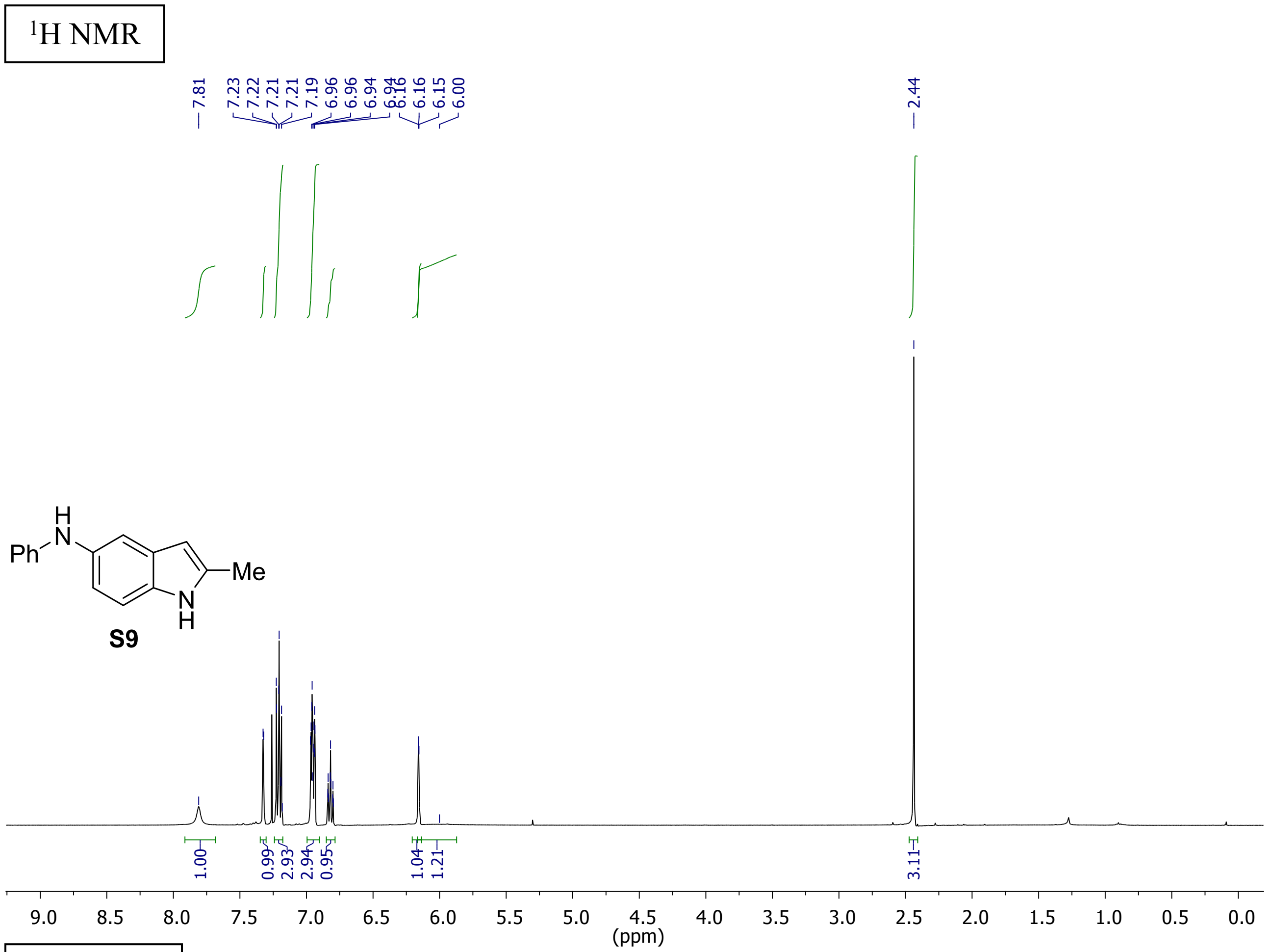

1H NMR
7.81
7.23
7.22
7.21
7.21
7.19
6.96
6.96
6.94
6.94
6.16
6.16
6.15
6.00
2.44
Ph
H
N
Me
N
H
S9
1.00
0.99
2.93
2.94
0.95
1.04
1.21
3.11
9.0 8.5 8.0 7.5 7.0 6.5 6.0 5.5 5.0 4.5 4.0 3.5 3.0 2.5 2.0 1.5 1.0 0.5 0.0
(ppm)

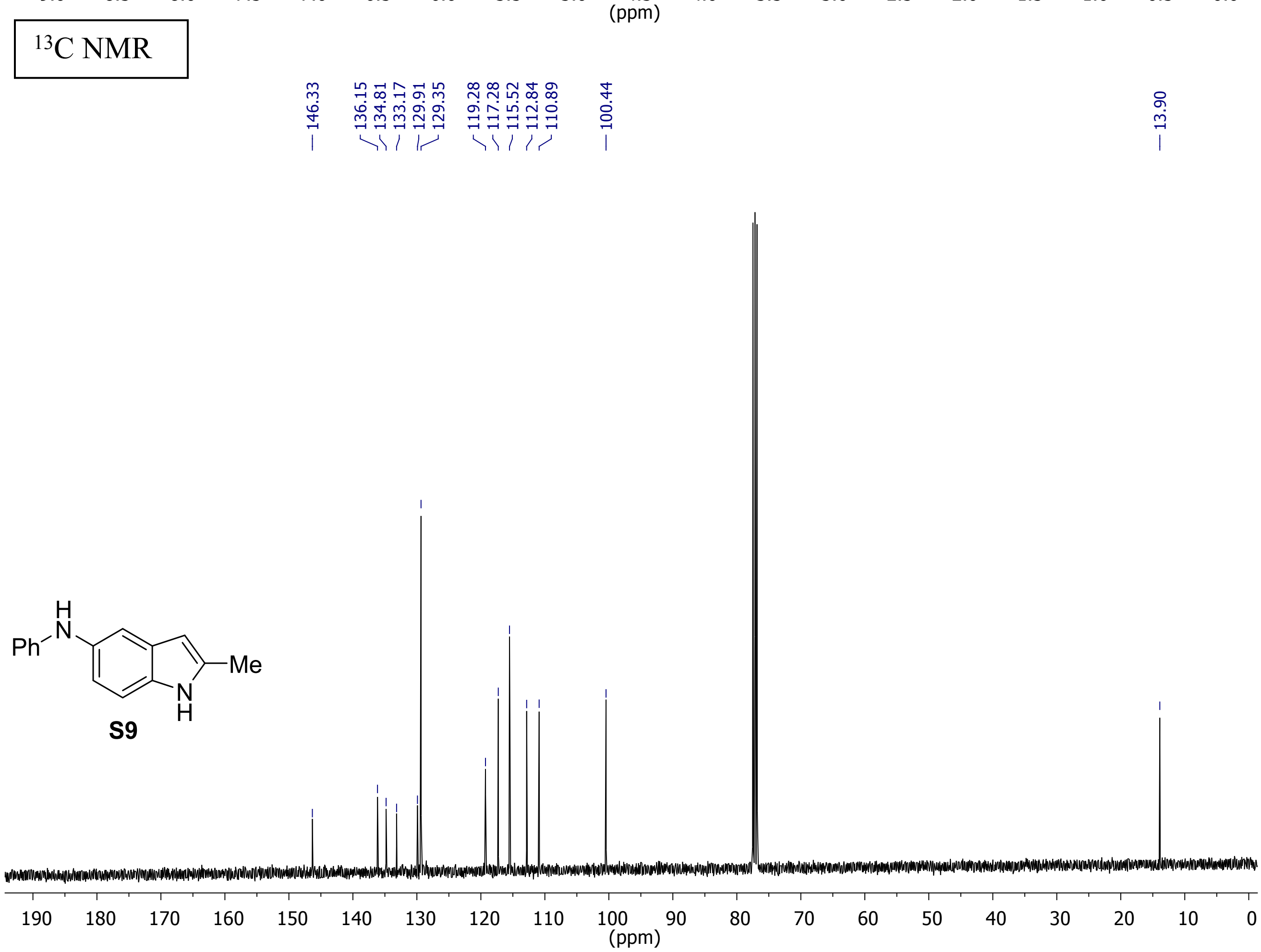

13C NMR
146.33
136.15
134.81
133.17
129.91
129.35
119.28
117.28
115.52
112.84
110.89
100.44
13.90
Ph
H
N
Me
N
H
S9
190 180 170 160 150 140 130 120 110 100 90 80 70 60 50 40 30 20 10 0
(ppm)

^{1}H NMR

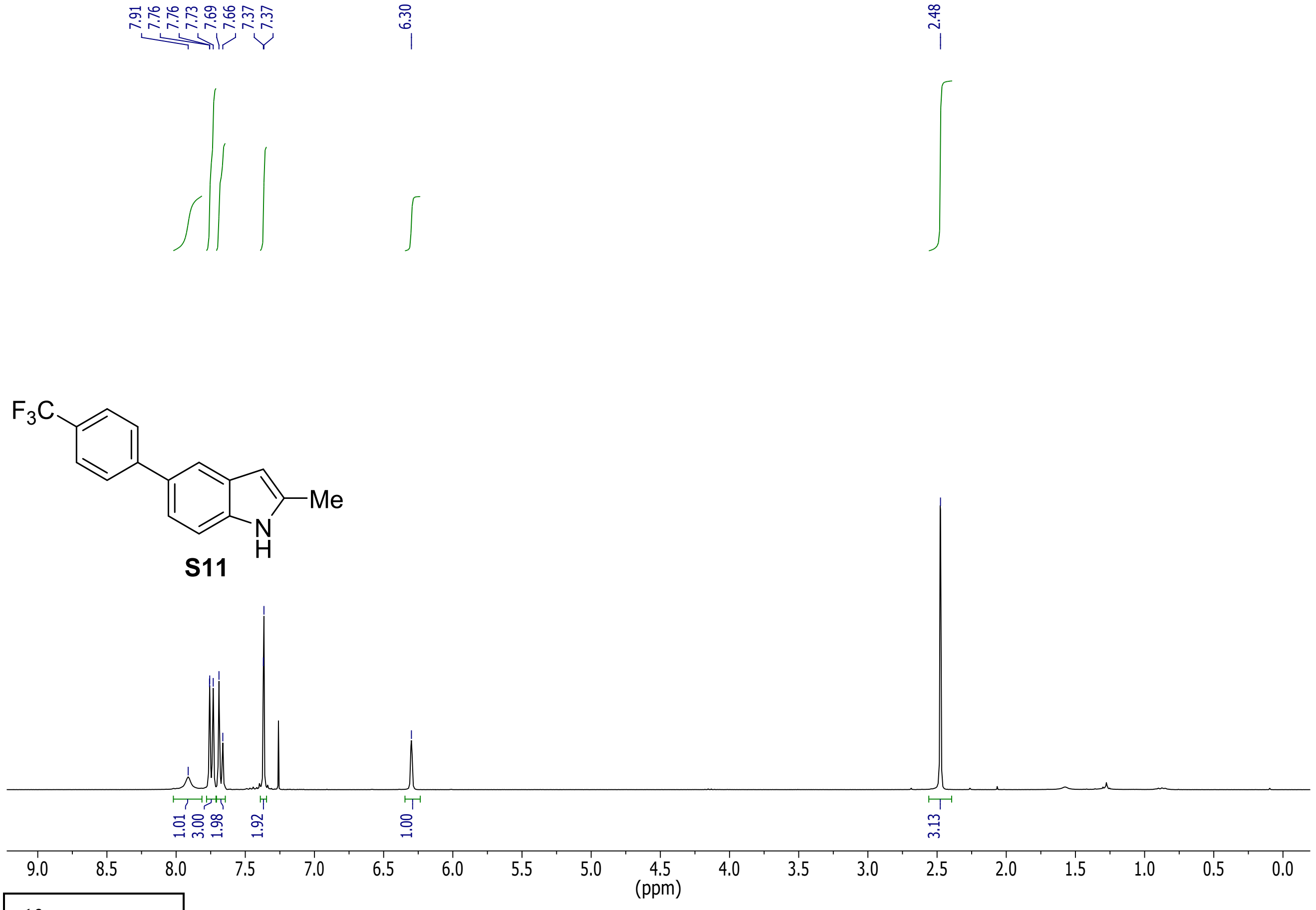

^{13}C NMR

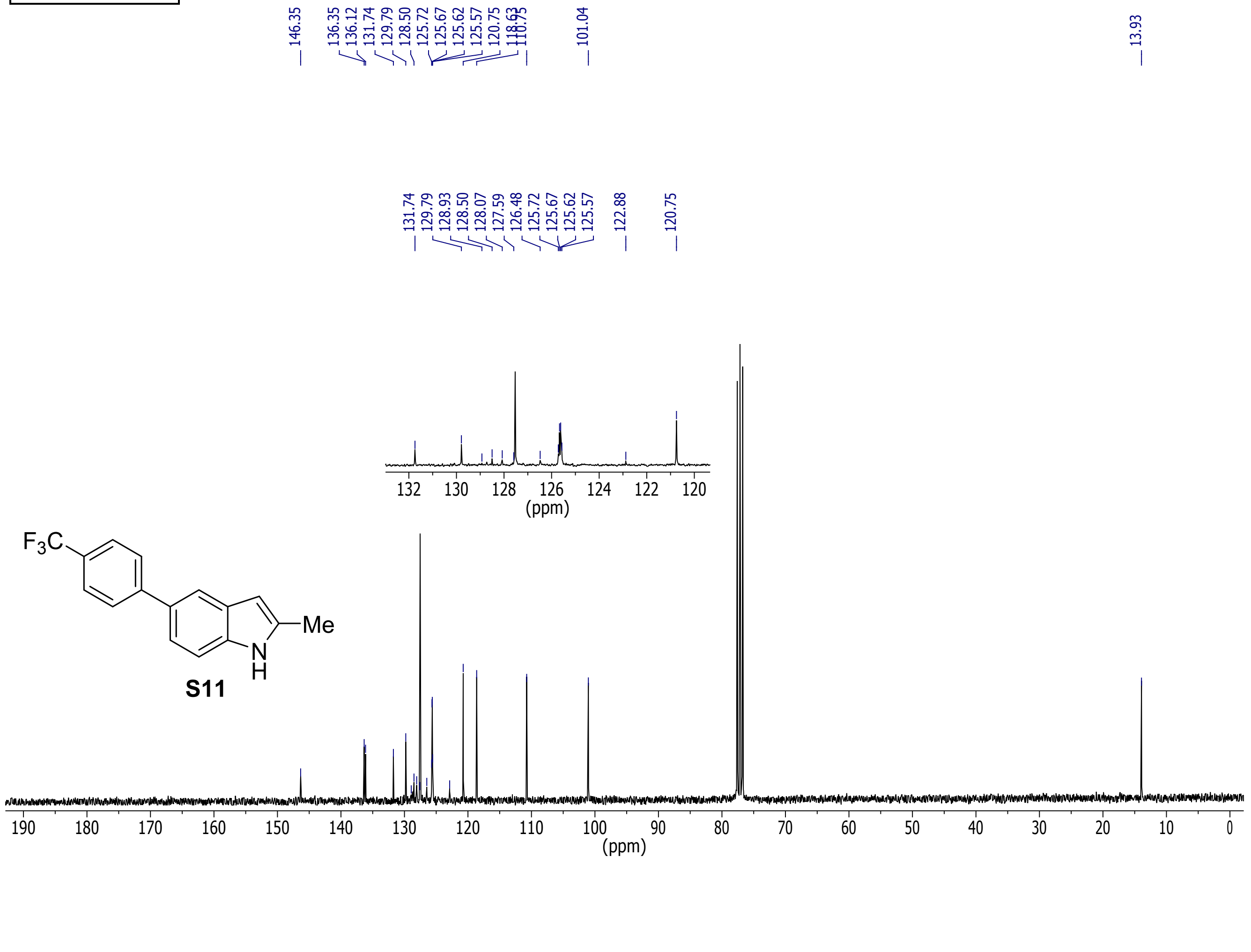

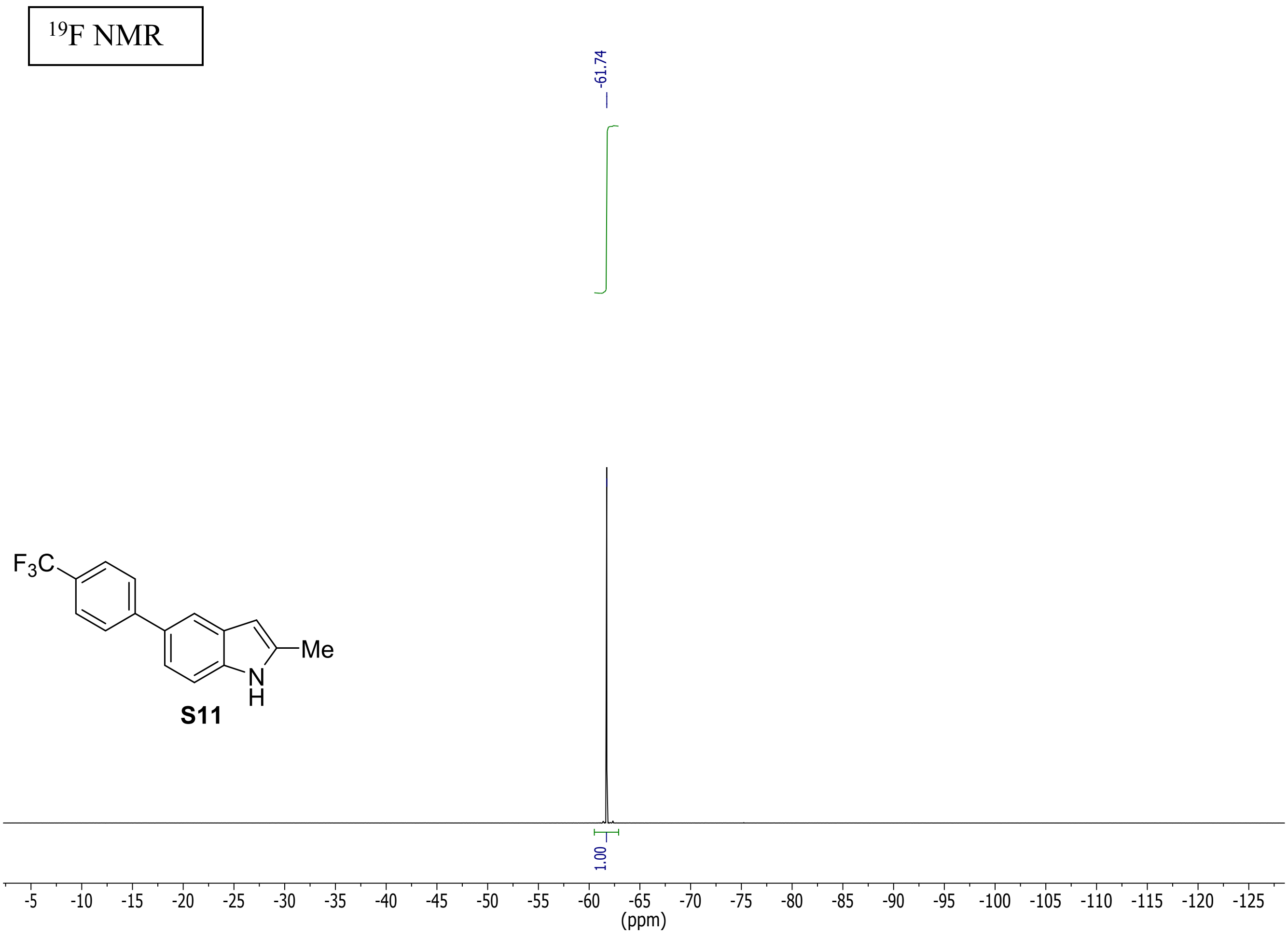

^{19}F NMR
-61.74
1.00
-5 -10 -15 -20 -25 -30 -35 -40 -45 -50 -55 -60 -65 -70 -75 -80 -85 -90 -95 -100 -105 -110 -115 -120 -125
(ppm)
F3C
Me
N
H
S11

^{1}H NMR

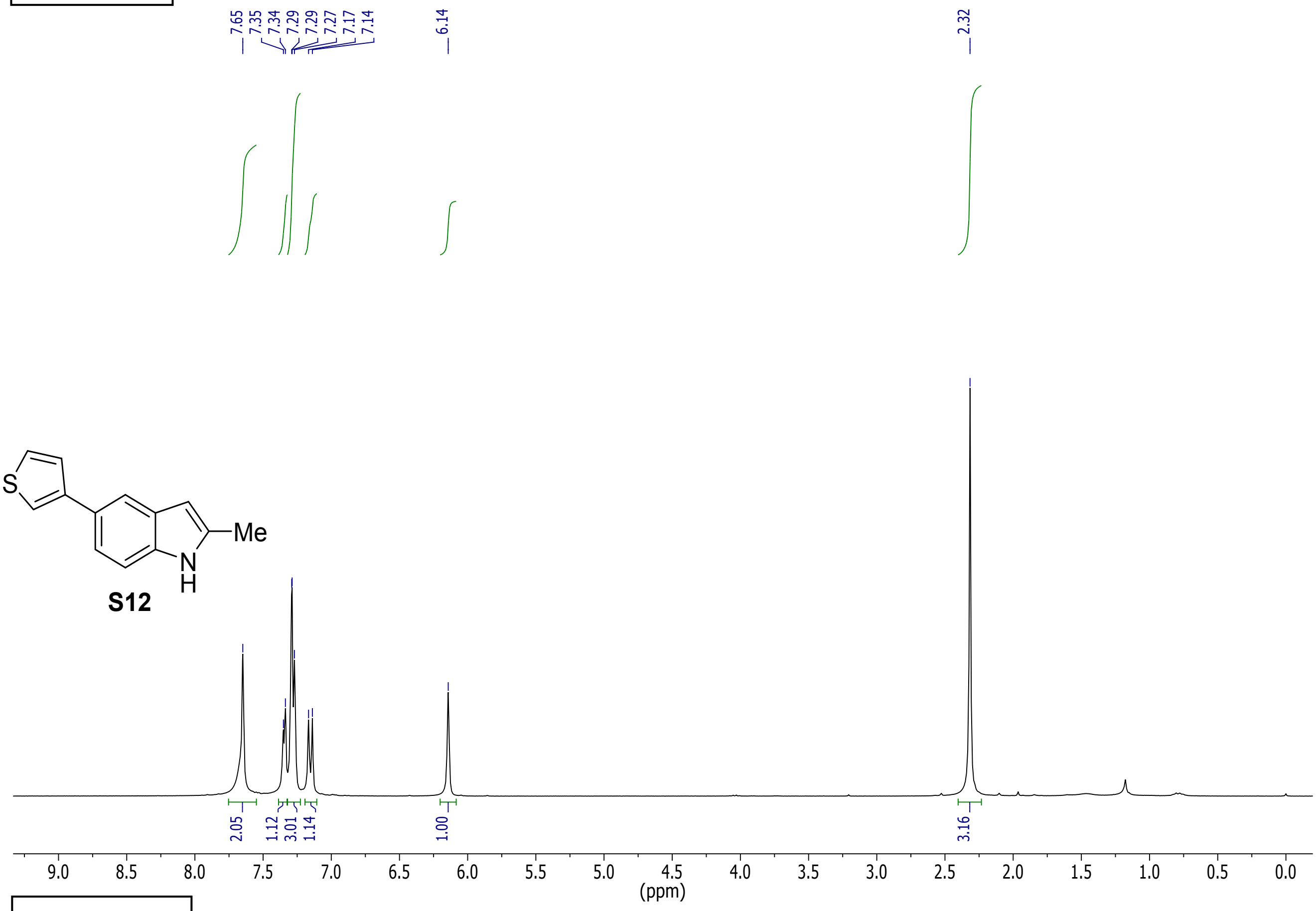

^{13}C NMR

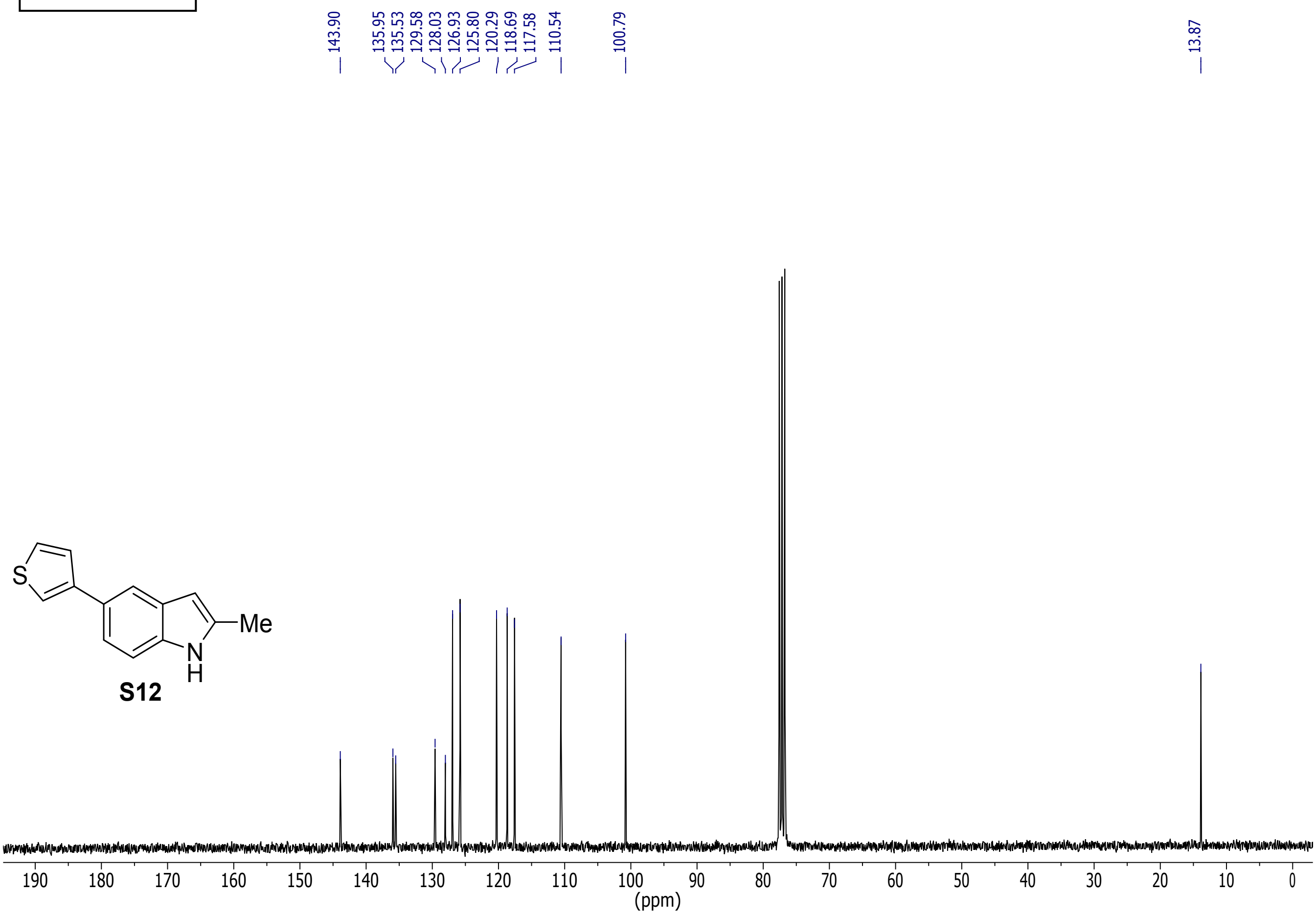

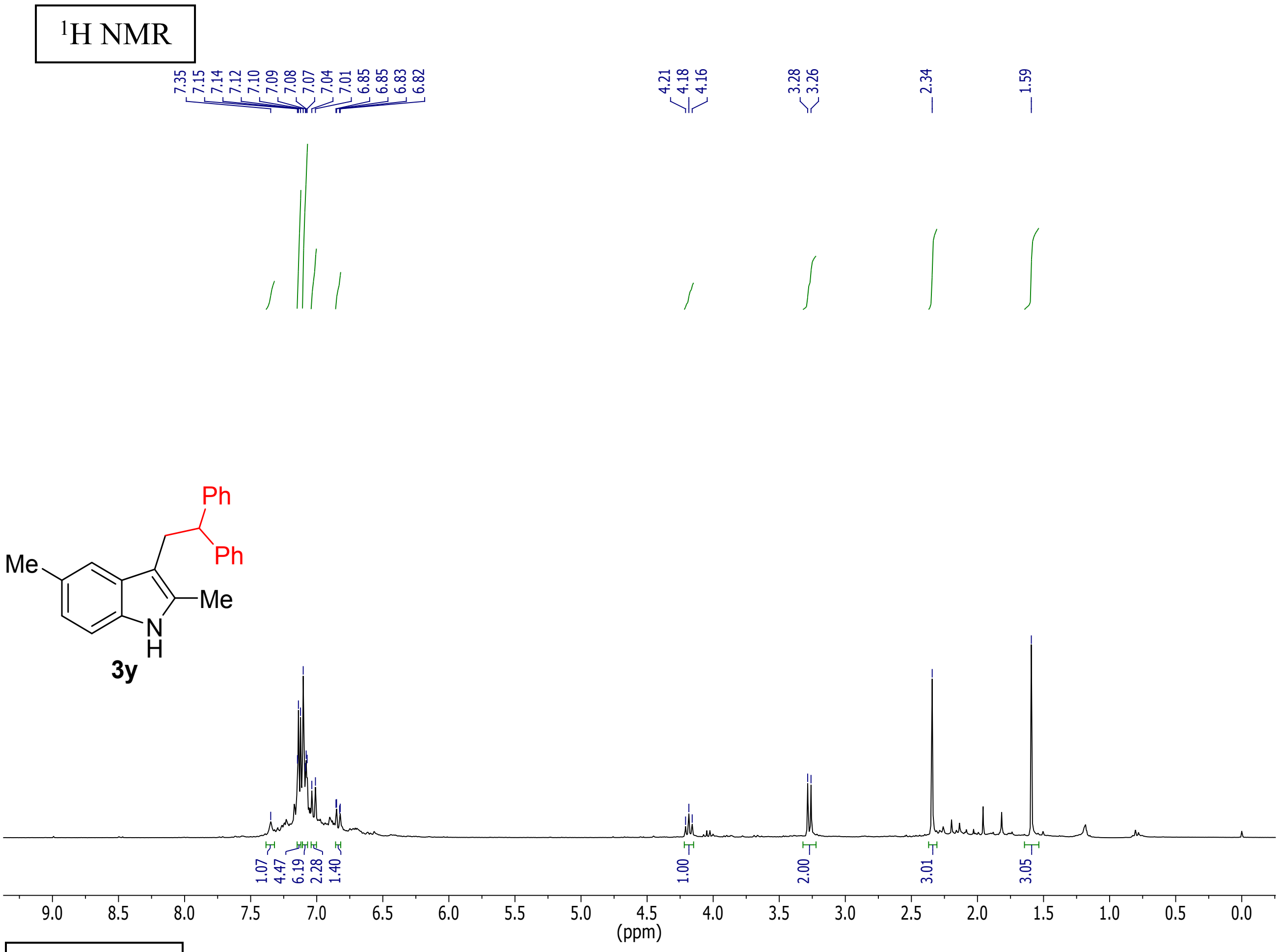
¹H NMR
7.35 7.15 7.14 7.12 7.10 7.09 7.08 7.07 7.04 7.01 6.85 6.85 6.83 6.82
4.21 4.18 4.16
3.28 3.26
2.34
1.59
Ph
Ph
Me
Me
N
H
3y
1.07 4.47 6.19 2.28 1.40
1.00
2.00
3.01
3.05
9.0 8.5 8.0 7.5 7.0 6.5 6.0 5.5 5.0 4.5 4.0 3.5 3.0 2.5 2.0 1.5 1.0 0.5 0.0
(ppm)

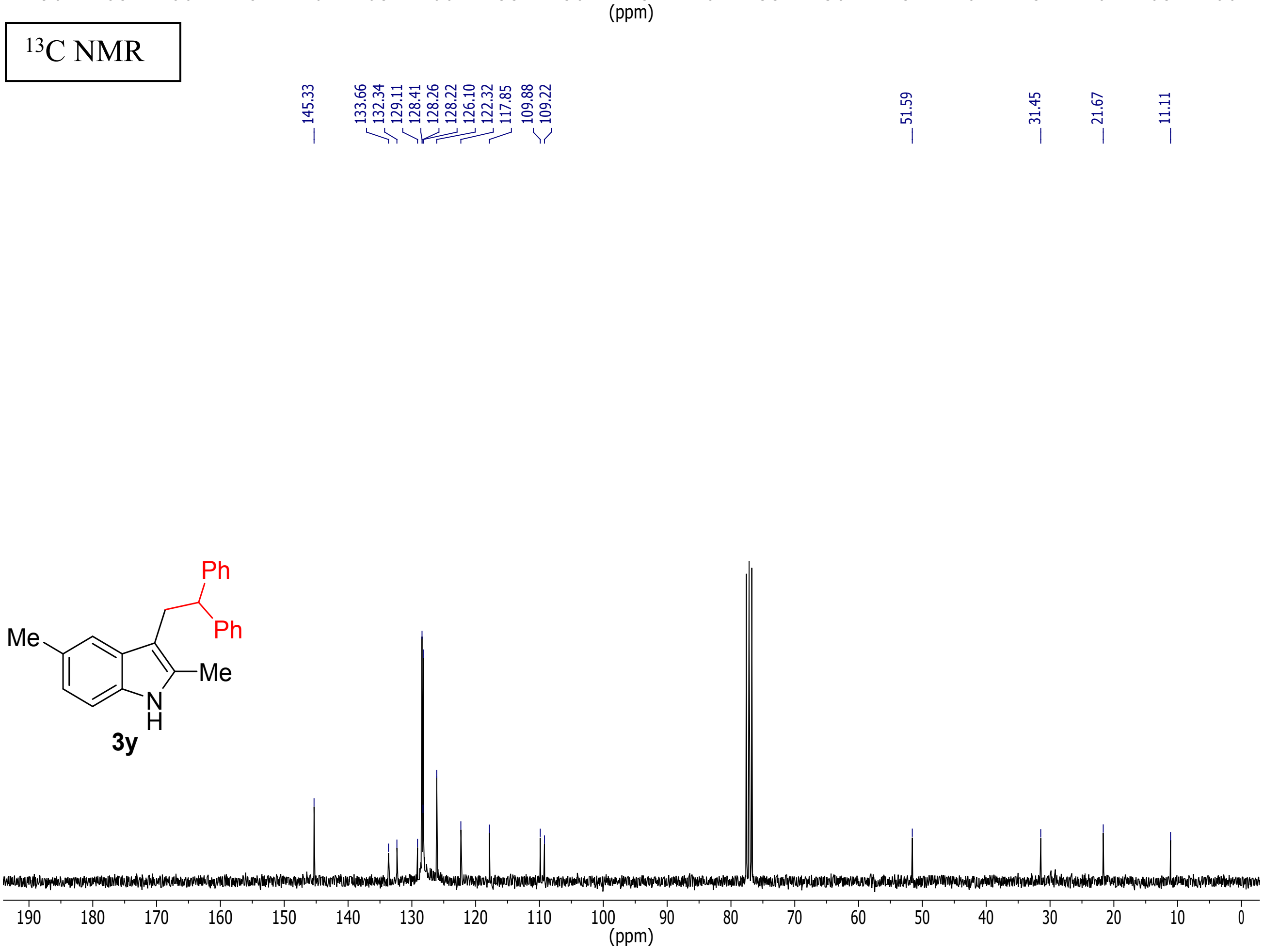
¹³C NMR
145.33
133.66 132.34 129.11 128.41 128.26 128.22 126.10 122.32 117.85 109.88 109.22
51.59
31.45
21.67
11.11
Ph
Ph
Me
Me
N
H
3y
190 180 170 160 150 140 130 120 110 100 90 80 70 60 50 40 30 20 10 0
(ppm)

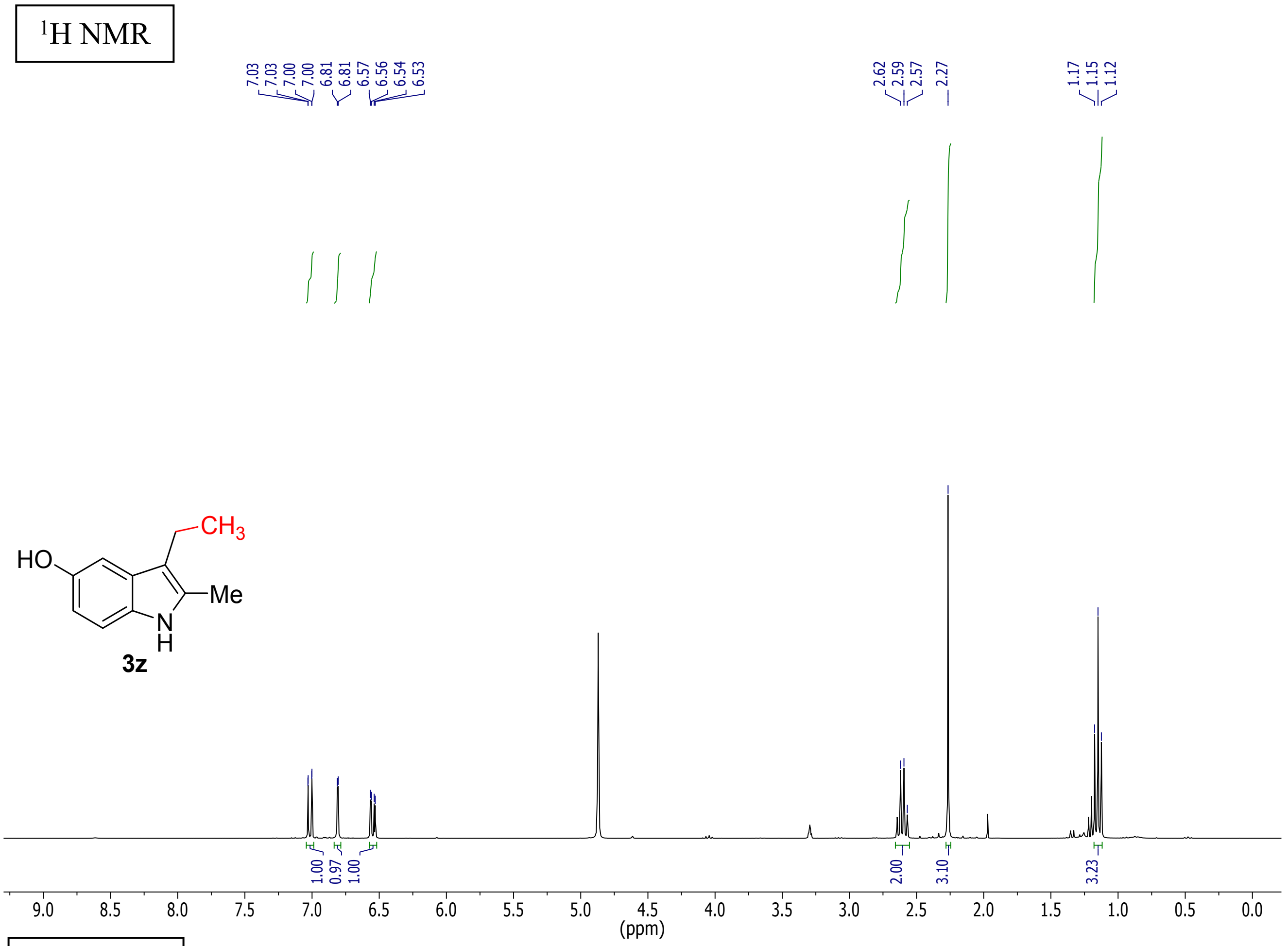

¹H NMR
7.03 7.03 7.00 7.00 6.81 6.81 6.57 6.56 6.54 6.53
2.62 2.59 2.57 2.27
1.17 1.15 1.12
HO
CH3
Me
N
H
3z
1.00 0.97 1.00
2.00 3.10
3.23
9.0 8.5 8.0 7.5 7.0 6.5 6.0 5.5 5.0 4.5 4.0 3.5 3.0 2.5 2.0 1.5 1.0 0.5 0.0
(ppm)

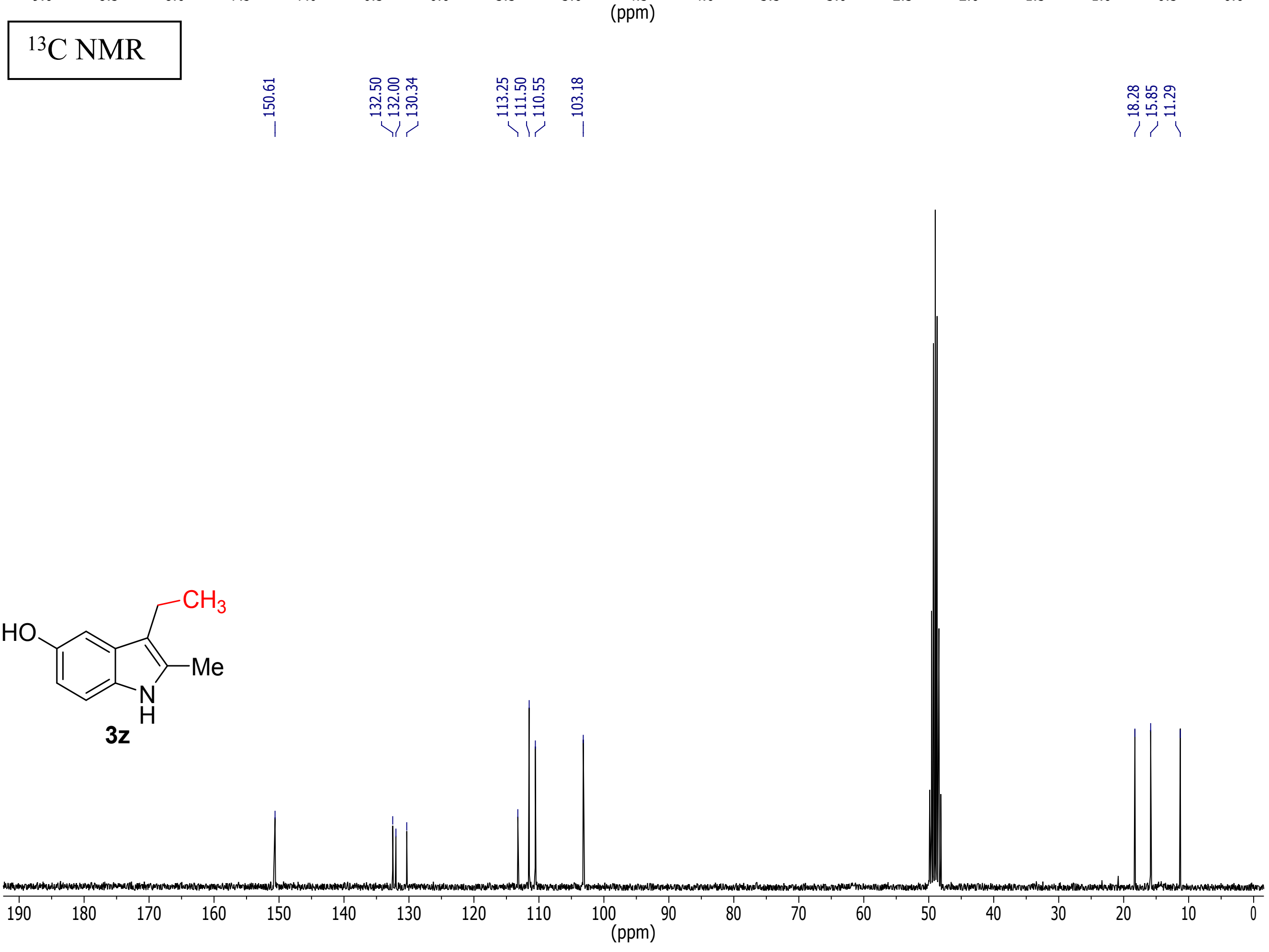

¹³C NMR
150.61
132.50 132.00 130.34
113.25 111.50 110.55
103.18
18.28 15.85 11.29
HO
CH3
Me
N
H
3z
190 180 170 160 150 140 130 120 110 100 90 80 70 60 50 40 30 20 10 0
(ppm)

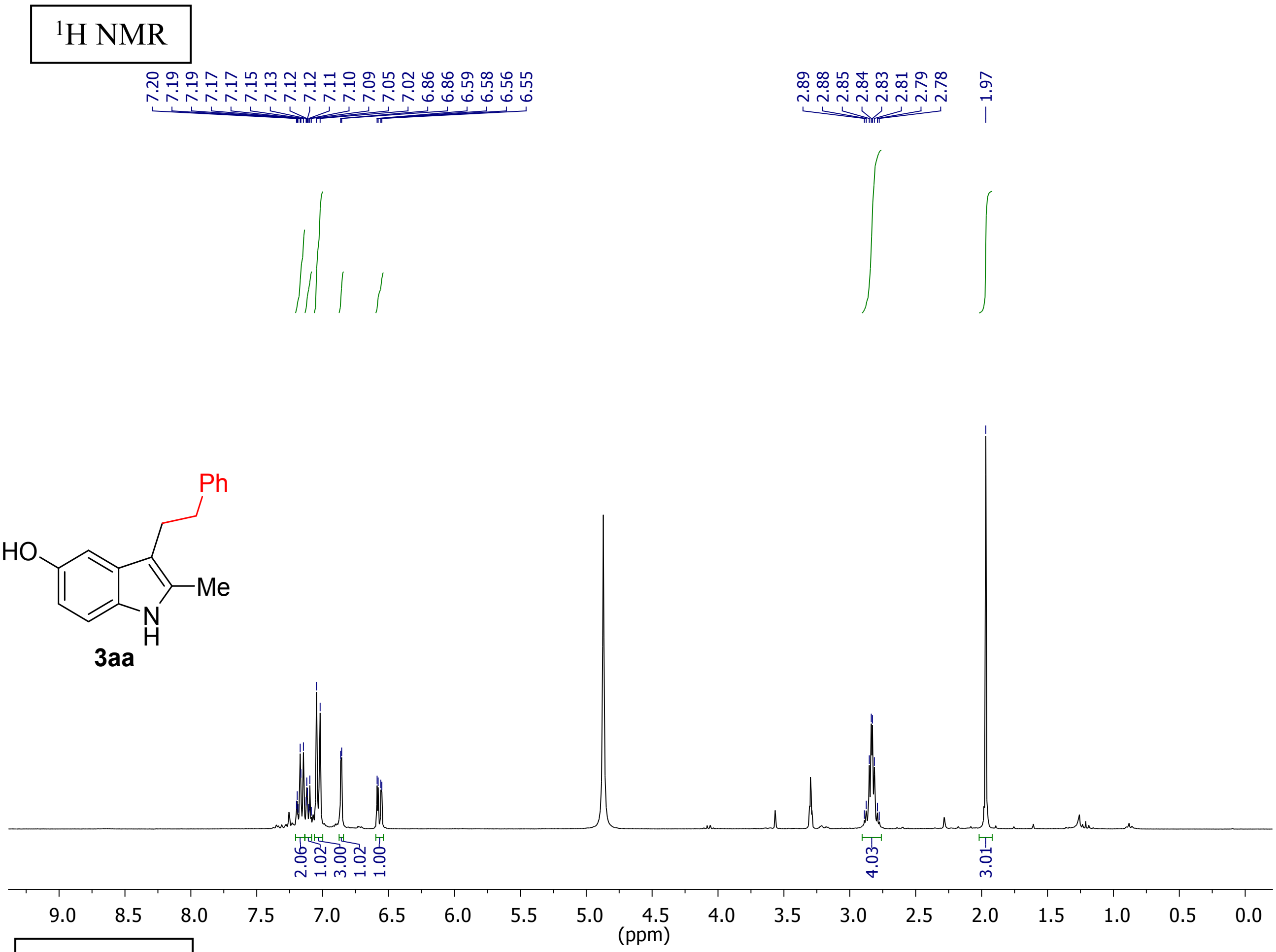

1H NMR
7.20 7.19 7.19 7.17 7.17 7.15 7.13 7.12 7.12 7.11 7.10 7.09 7.05 7.02 6.86 6.86 6.59 6.58 6.56 6.55
2.89 2.88 2.85 2.84 2.83 2.81 2.79 2.78
1.97
Ph
HO
Me
N
H
3aa
2.06 1.02 3.00 1.02 1.00
4.03
3.01
9.0 8.5 8.0 7.5 7.0 6.5 6.0 5.5 5.0 4.5 4.0 3.5 3.0 2.5 2.0 1.5 1.0 0.5 0.0
(ppm)

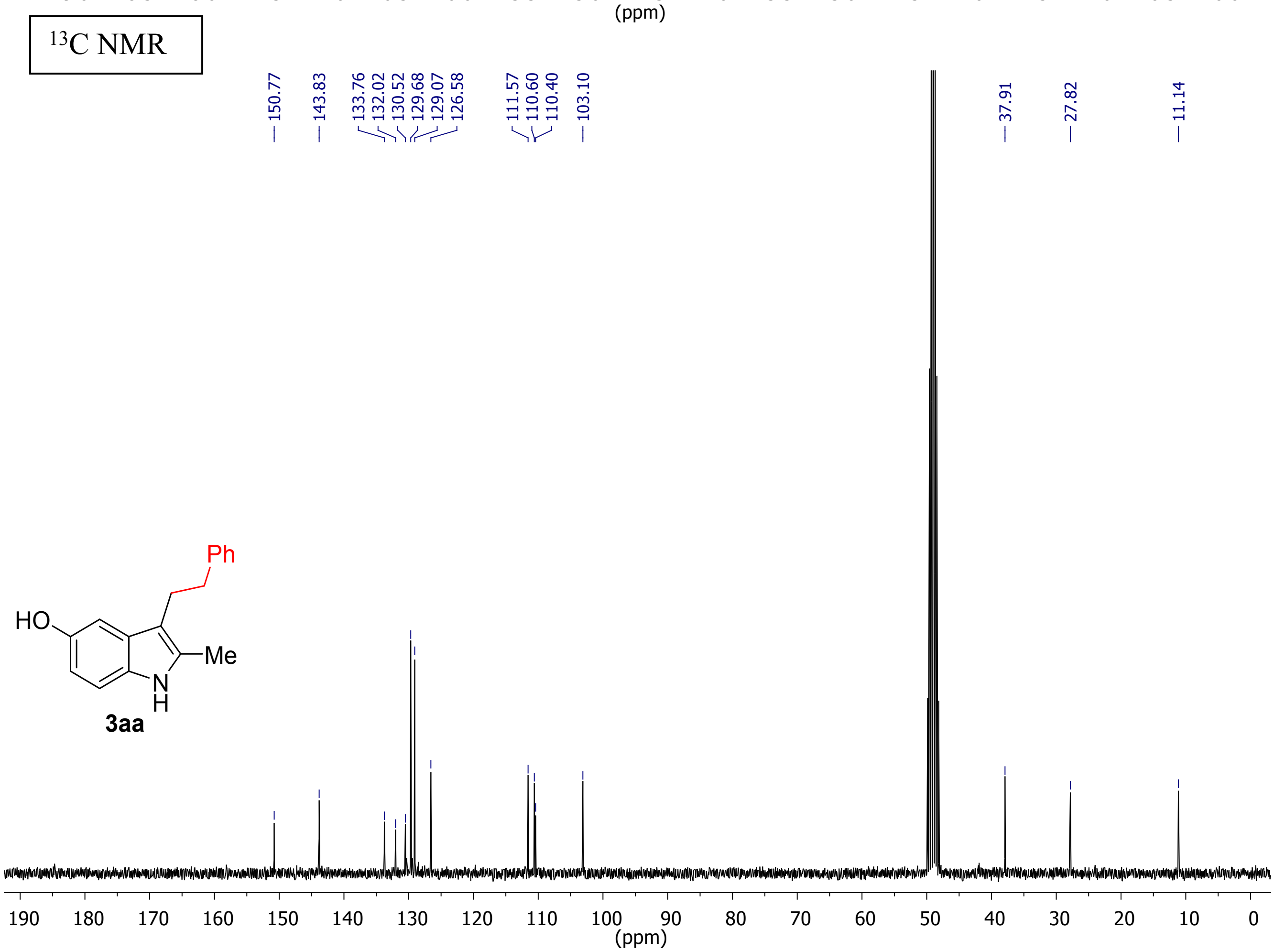

13C NMR
150.77
143.83
133.76 132.02 130.52 129.68 129.07 126.58
111.57 110.60 110.40
103.10
37.91
27.82
11.14
Ph
HO
Me
N
H
3aa
190 180 170 160 150 140 130 120 110 100 90 80 70 60 50 40 30 20 10 0
(ppm)

^{1}H NMR

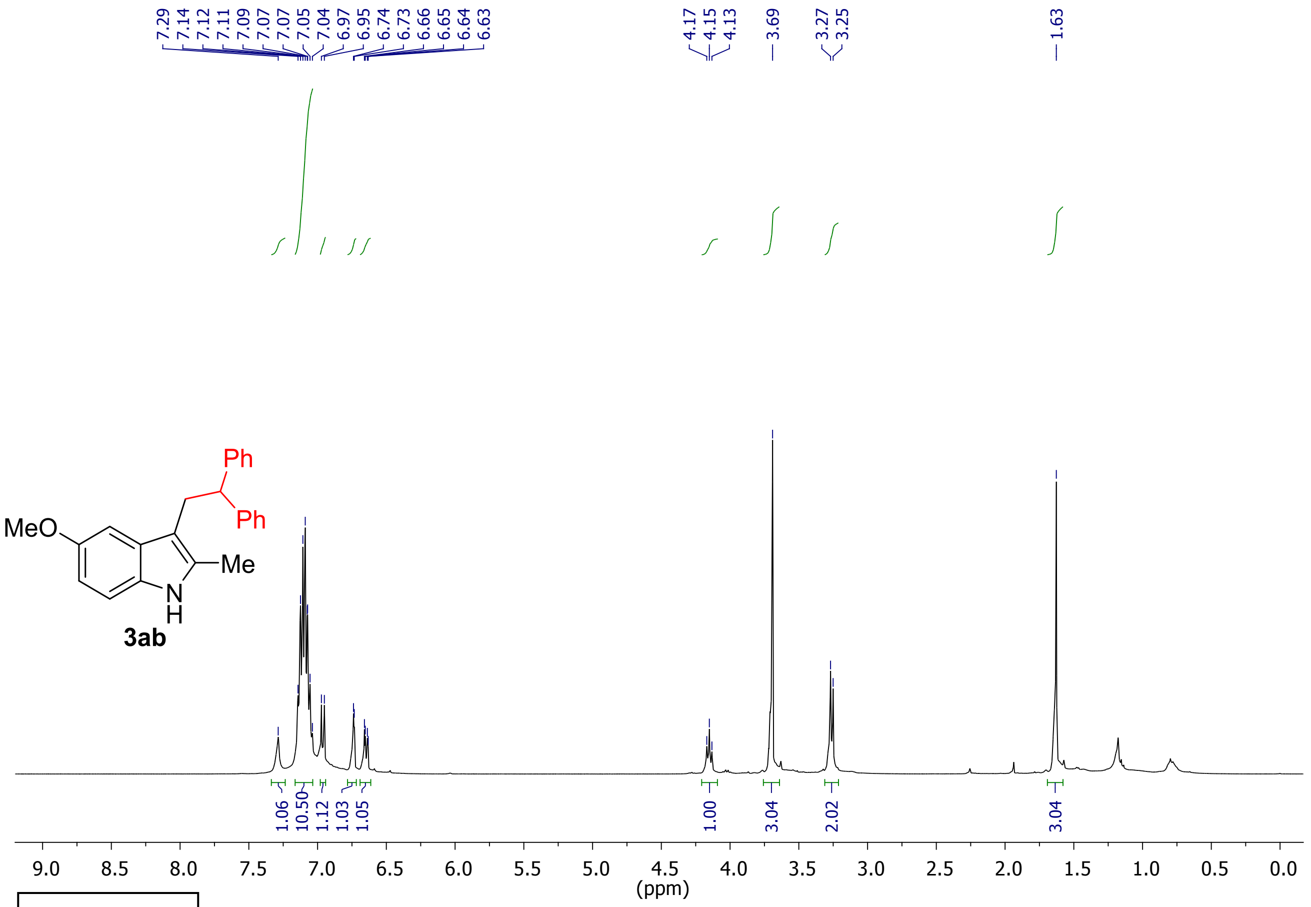

^{13}C NMR

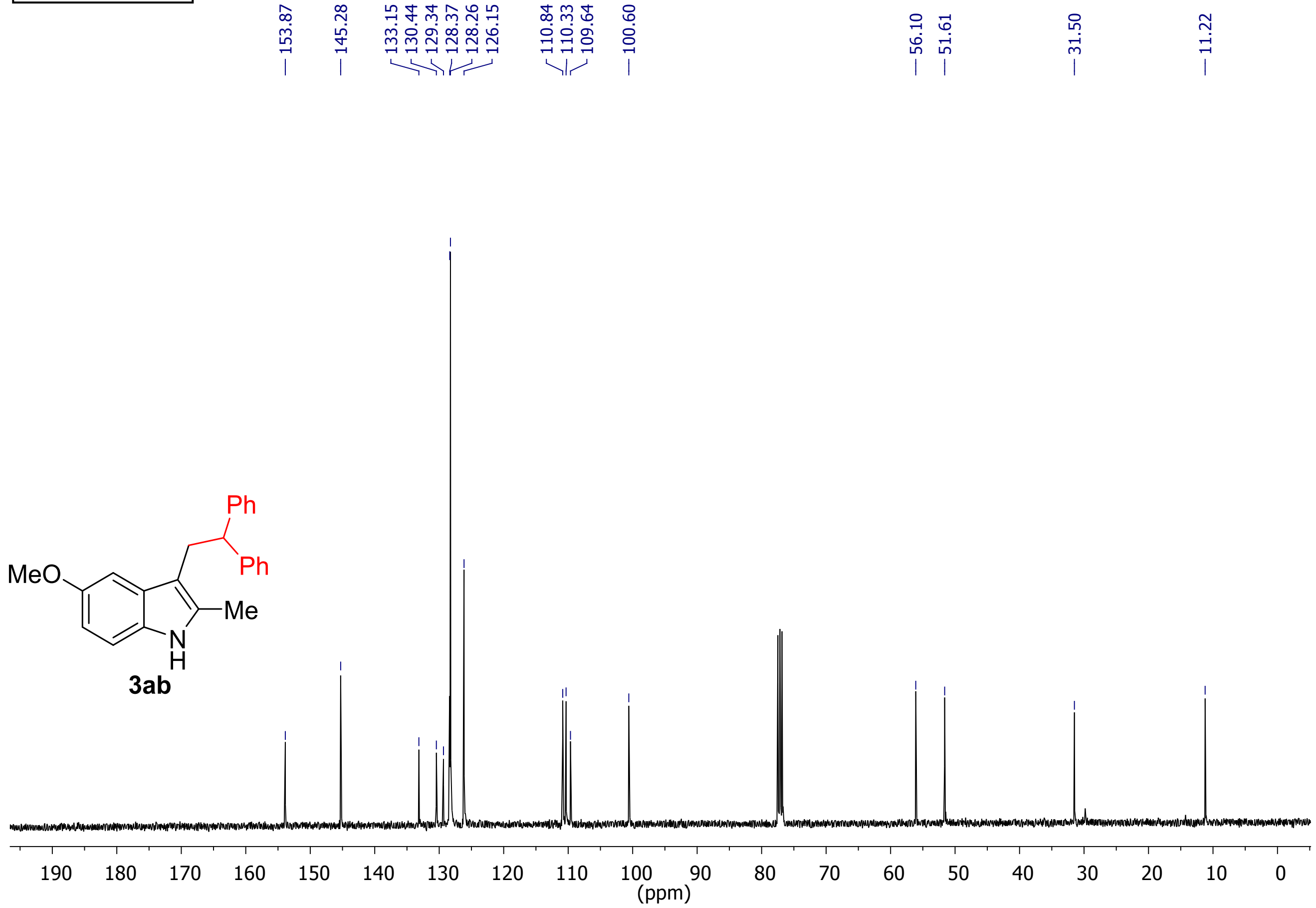

^{1}H NMR

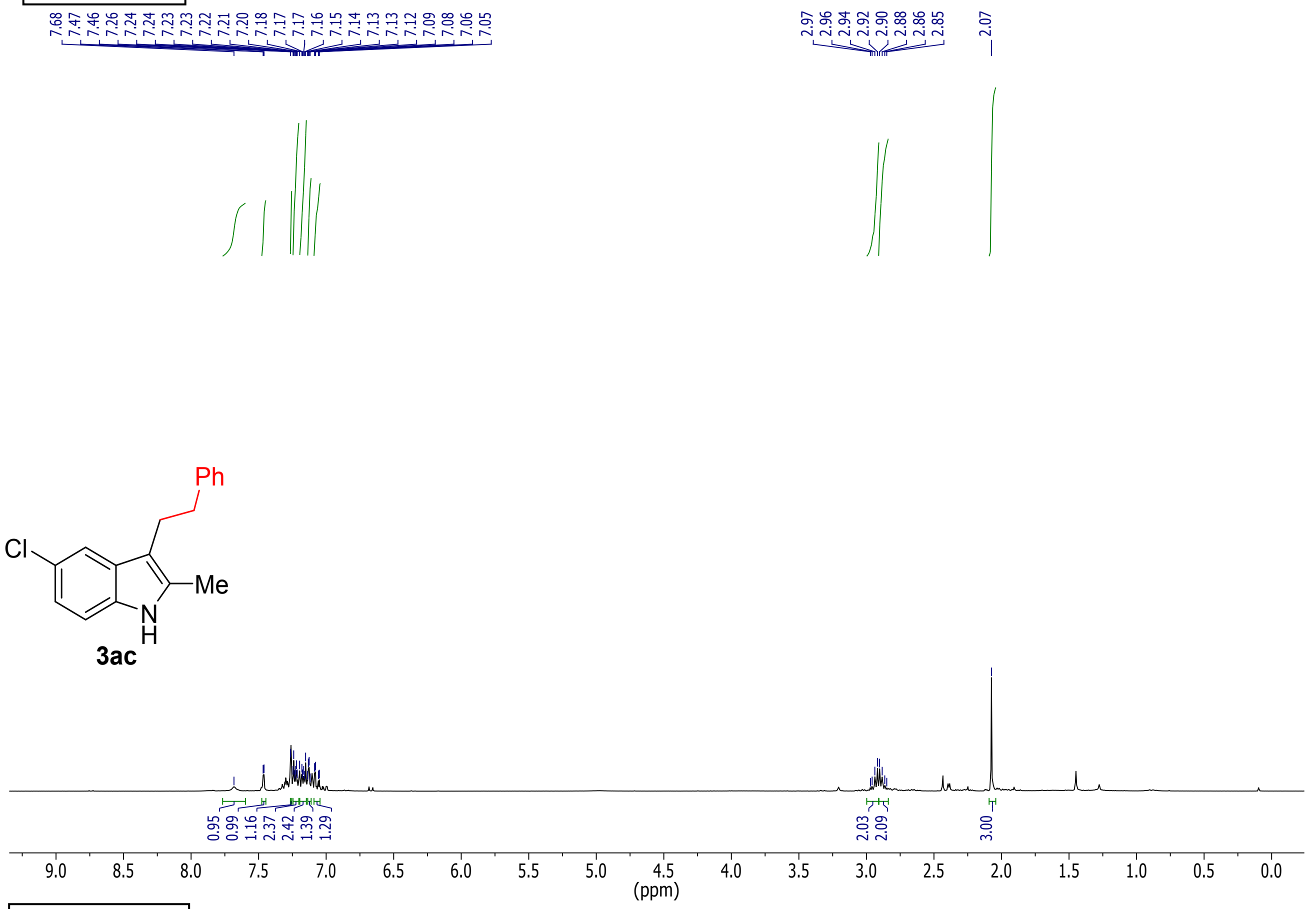

^{13}C NMR

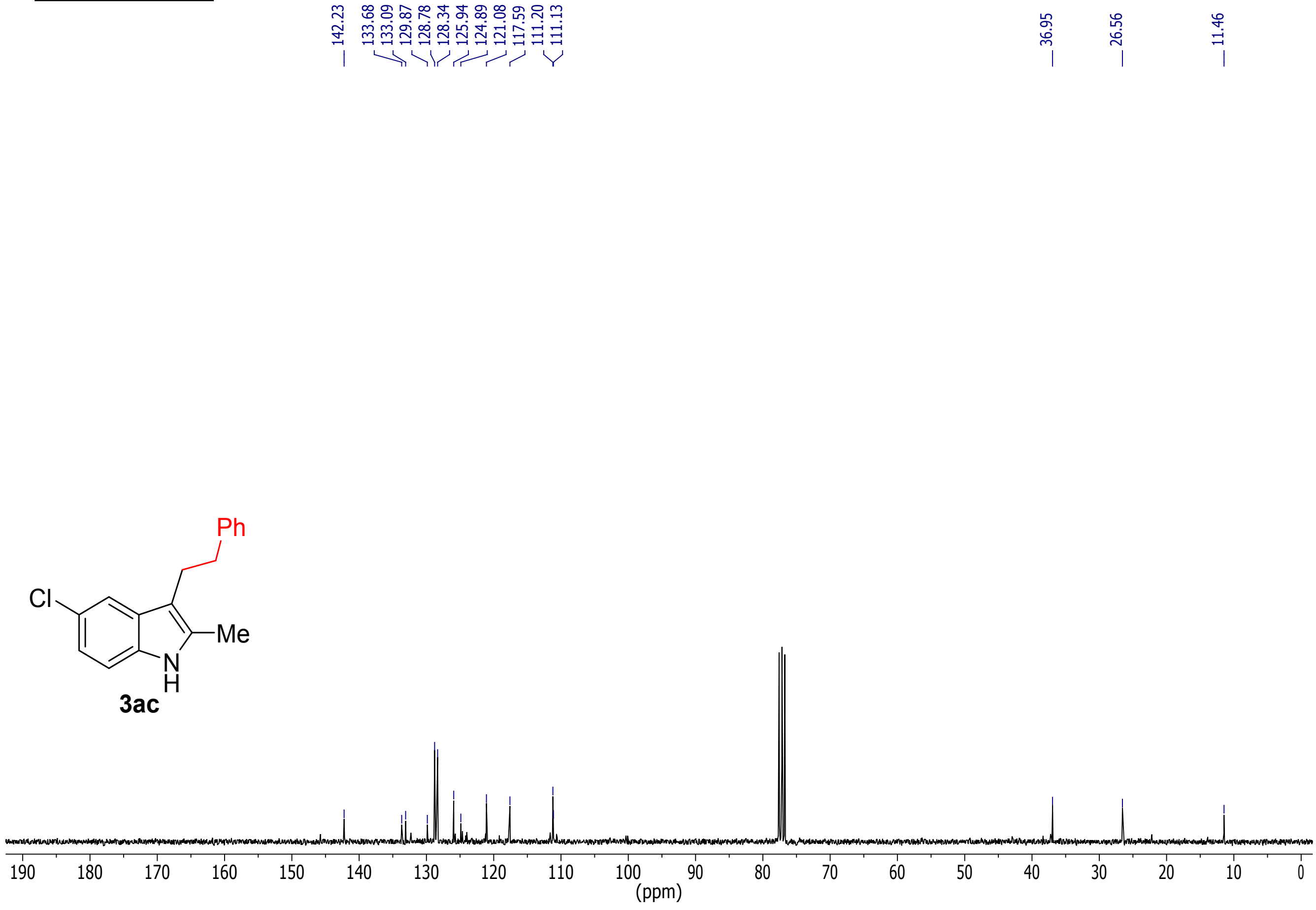

^{1}H NMR

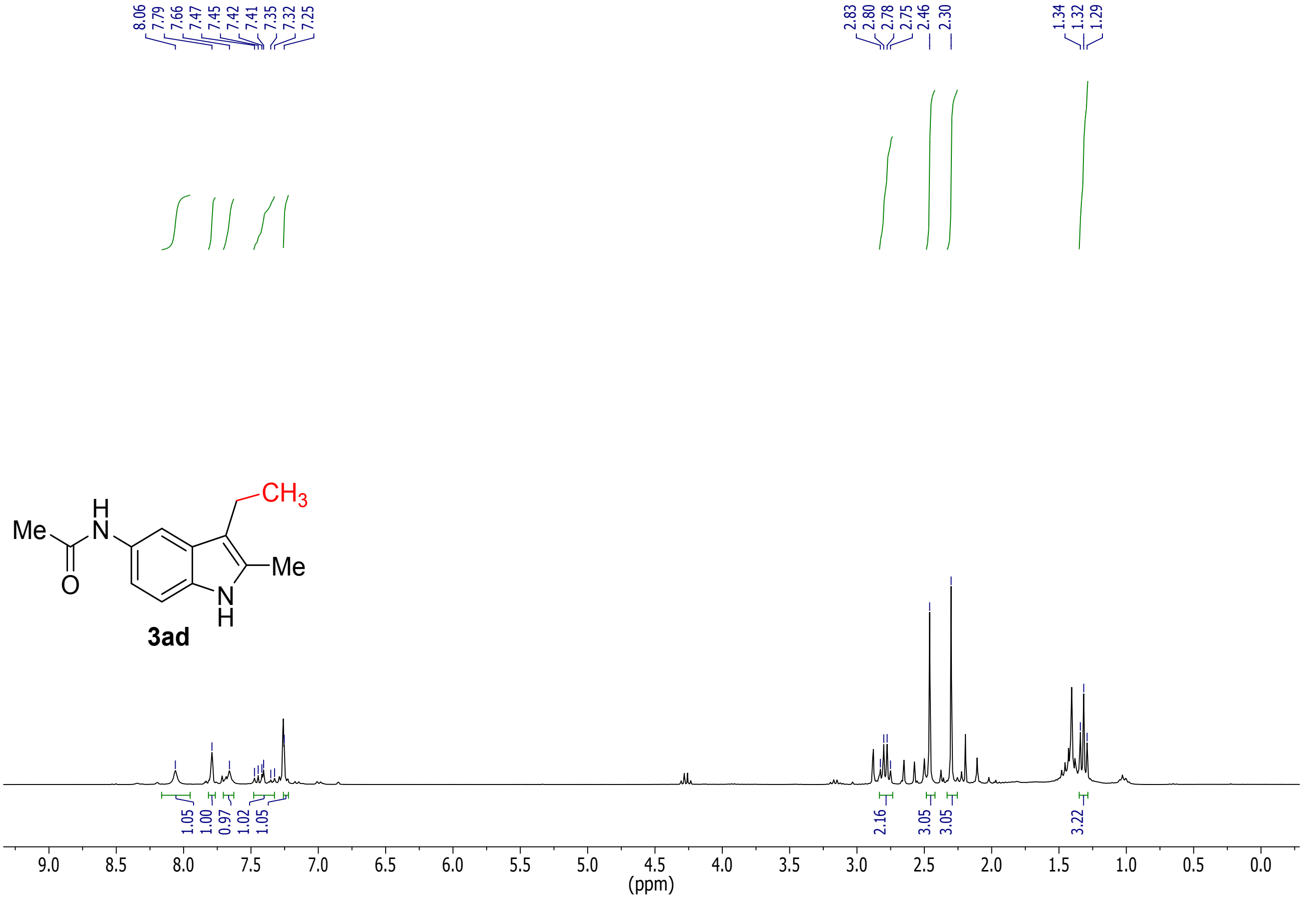

^{13}C NMR

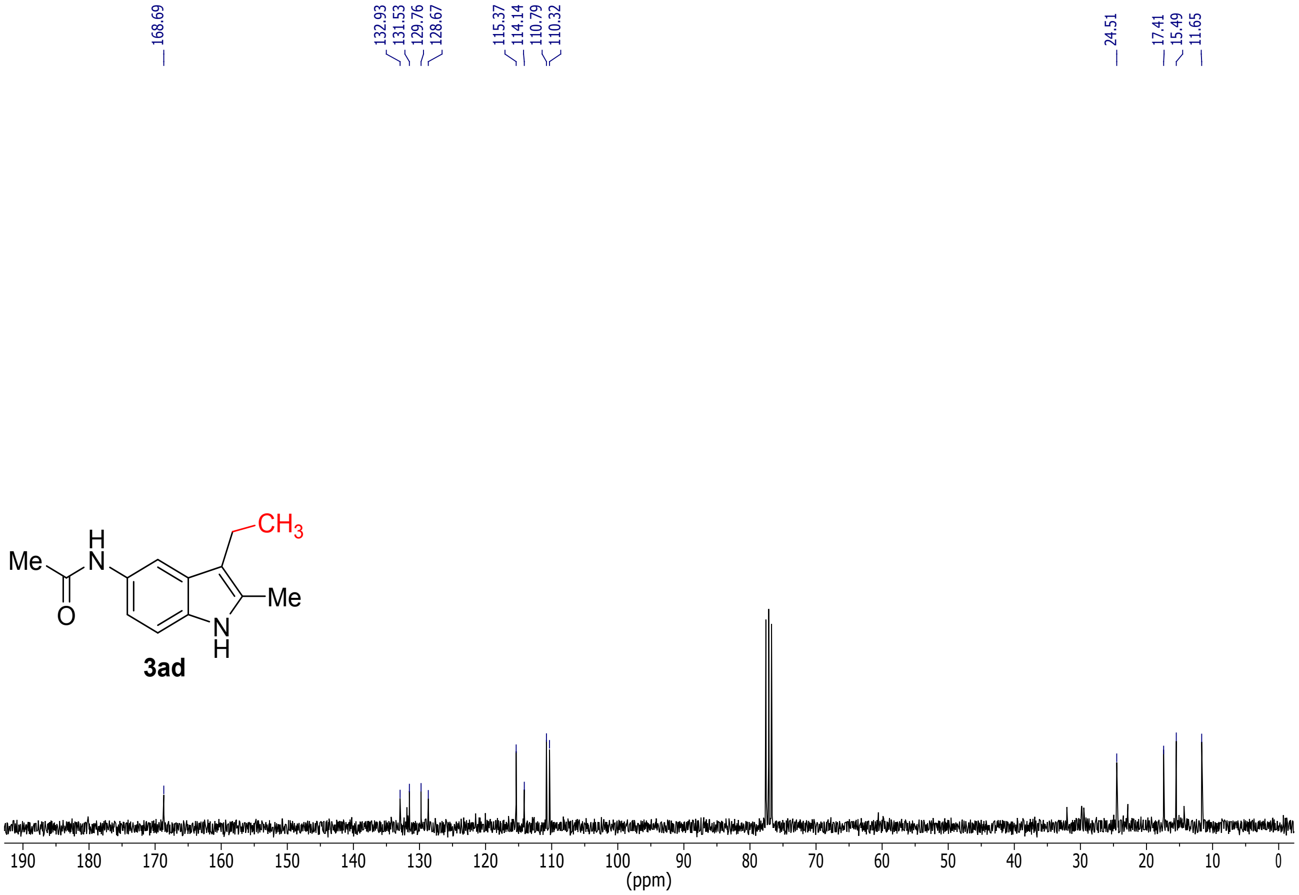

^{1}H NMR

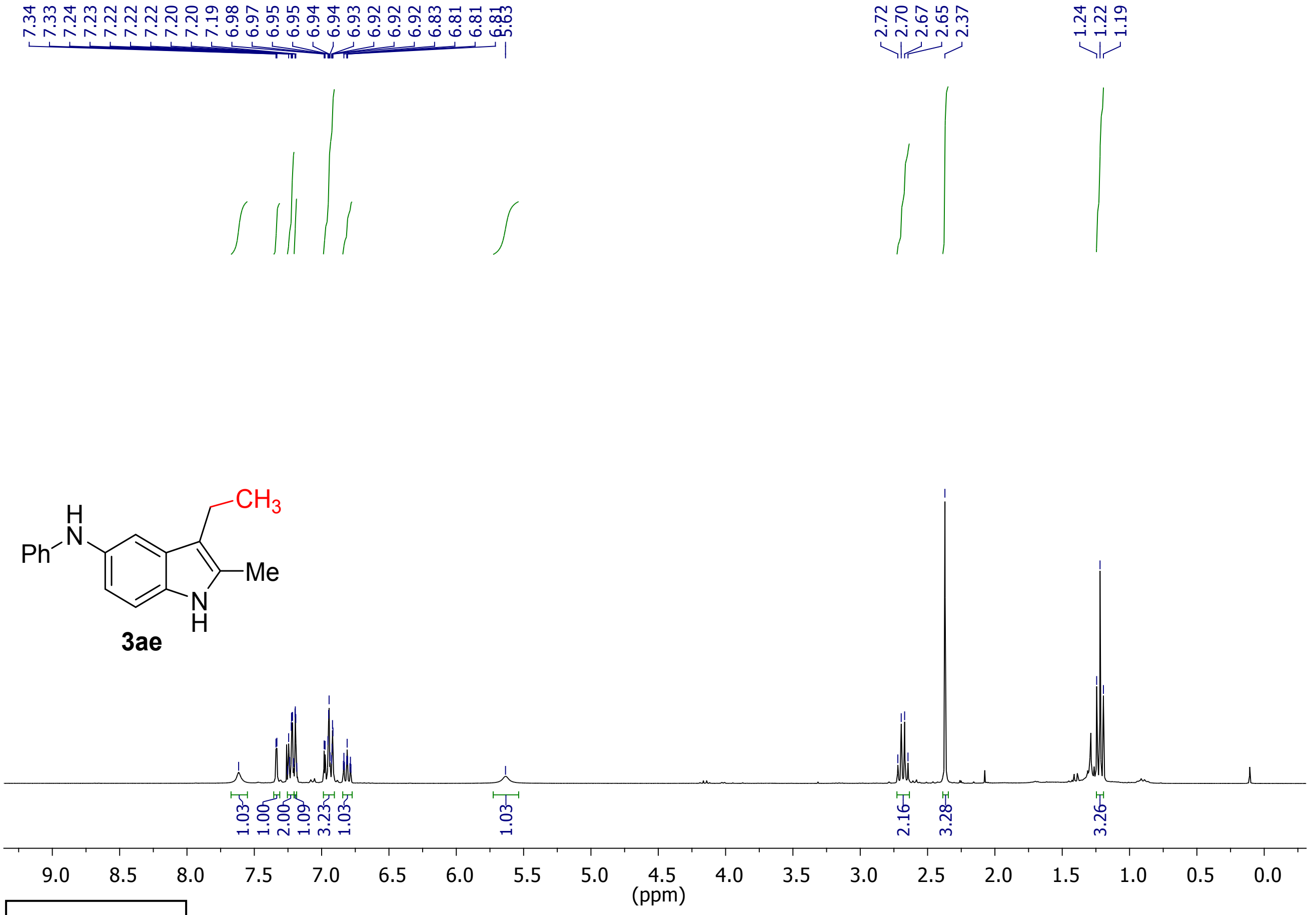

^{13}C NMR

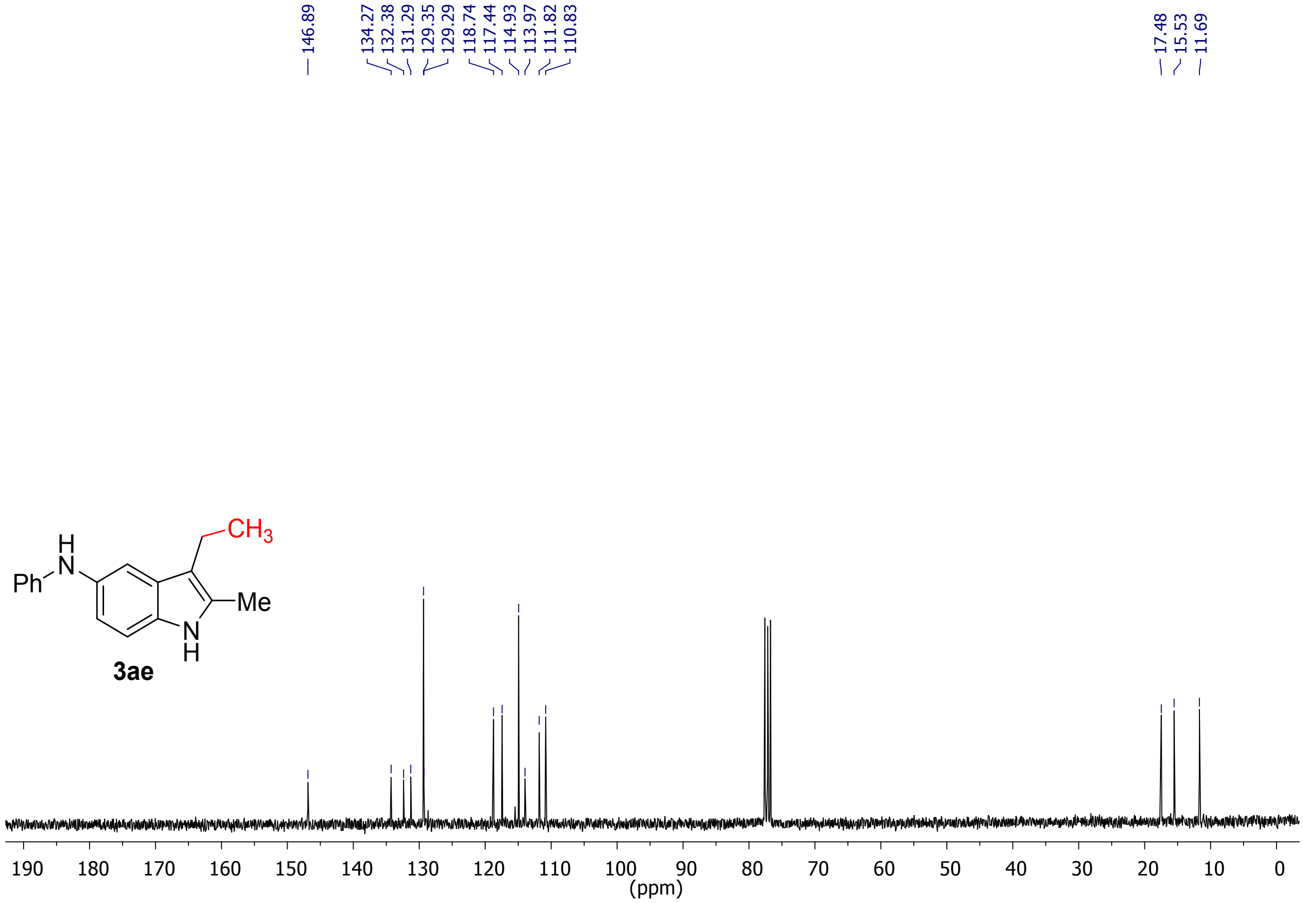

^{1}H NMR

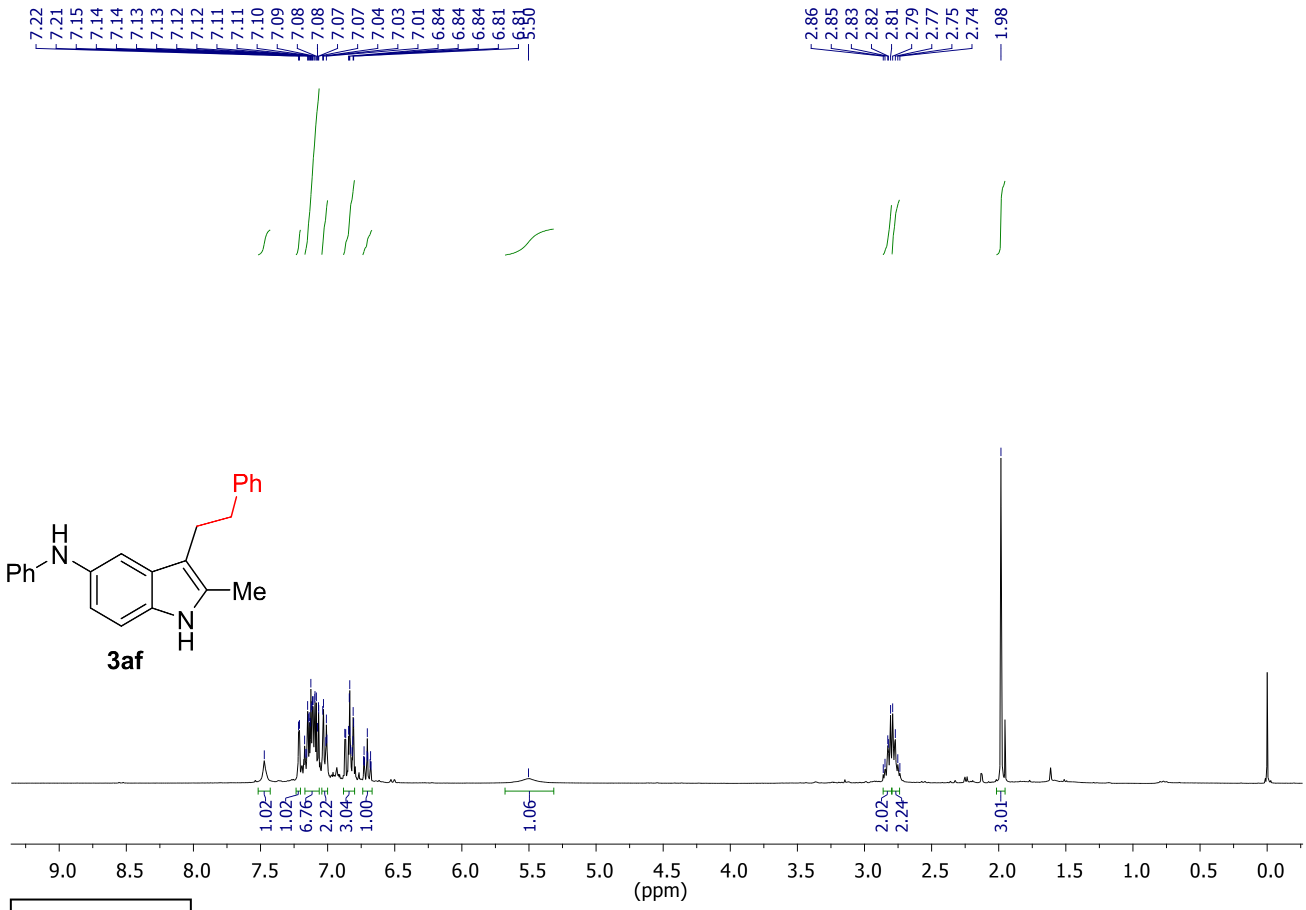

^{13}C NMR

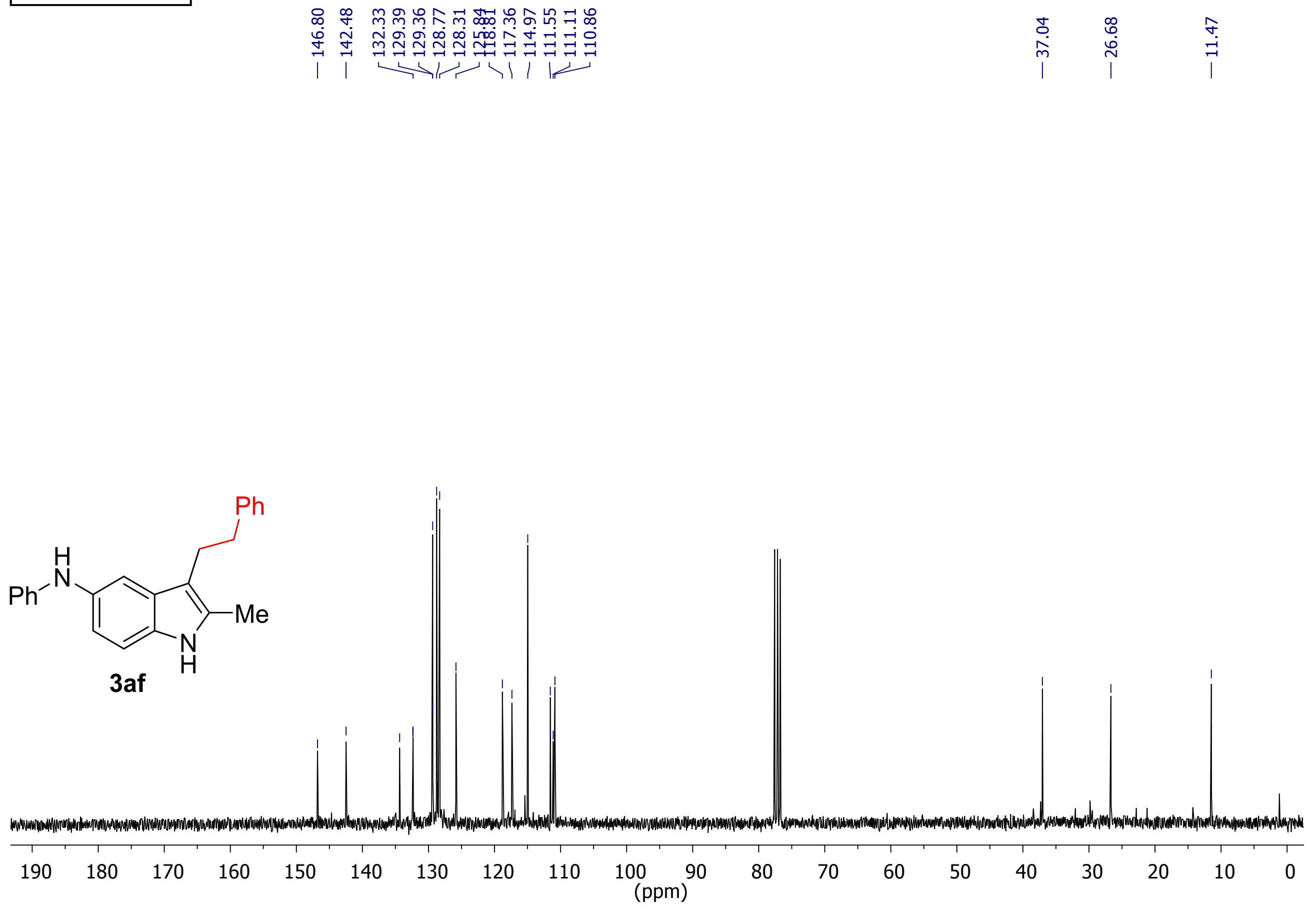

^{1}H NMR

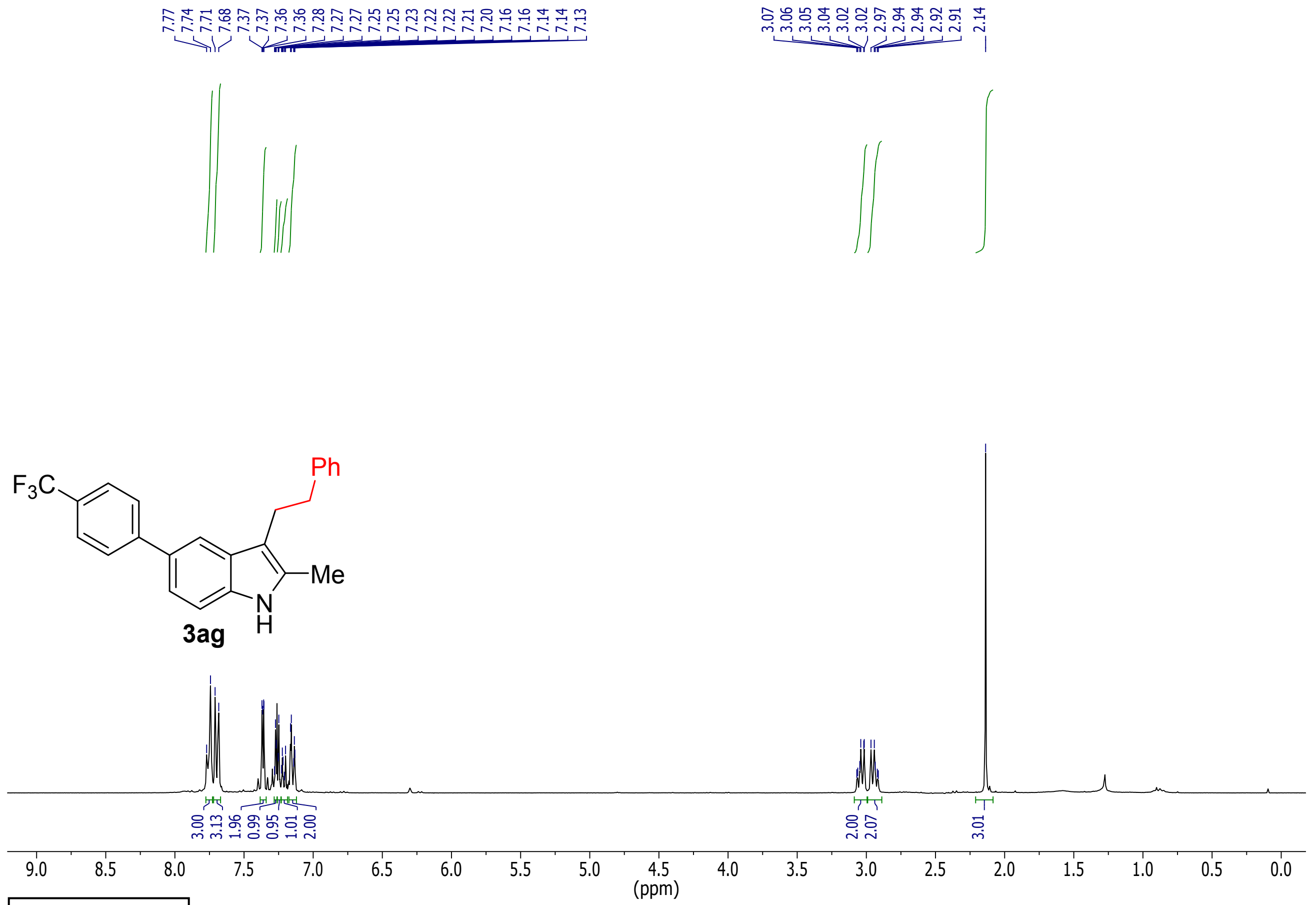

^{13}C NMR

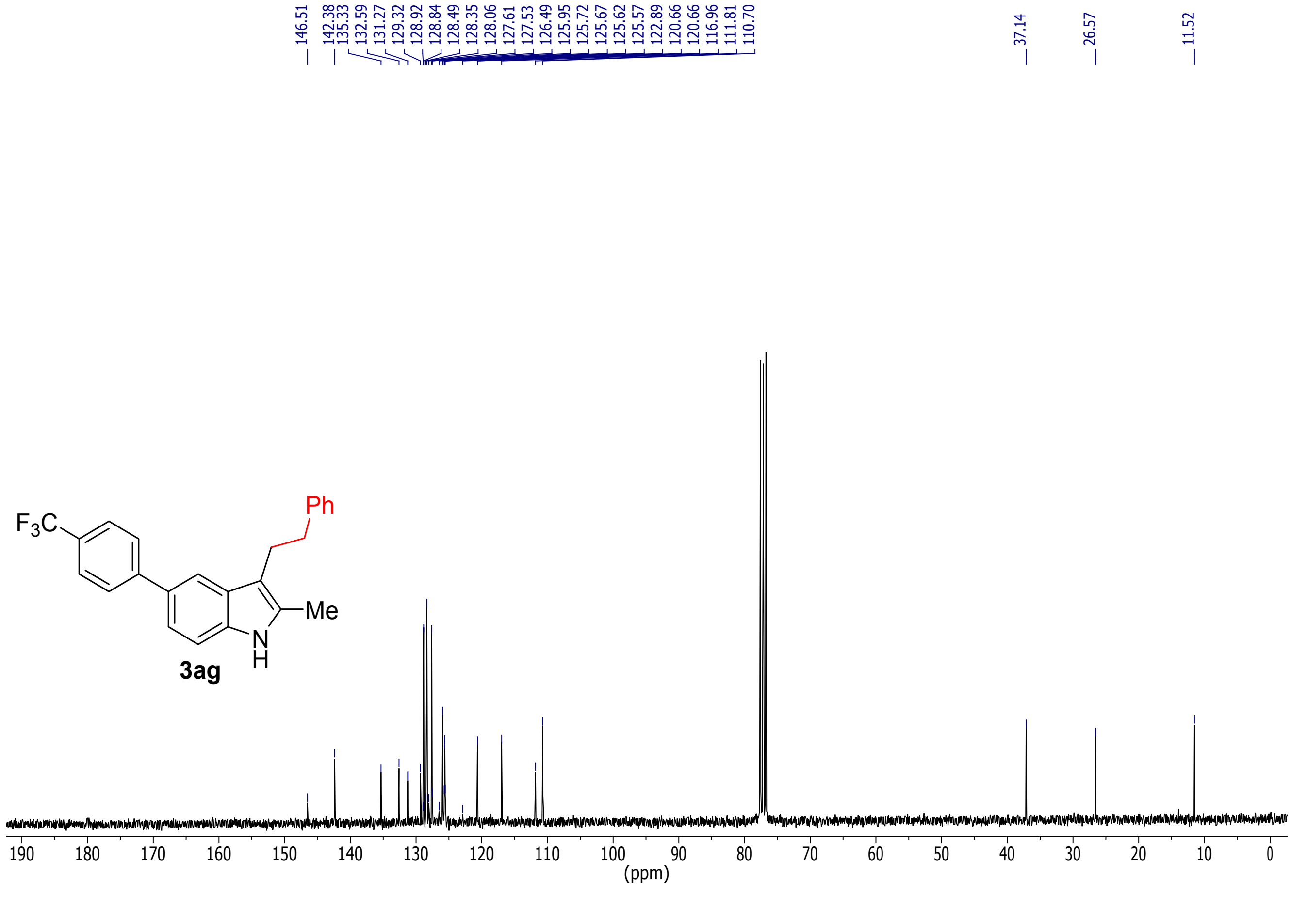

^{19}F NMR

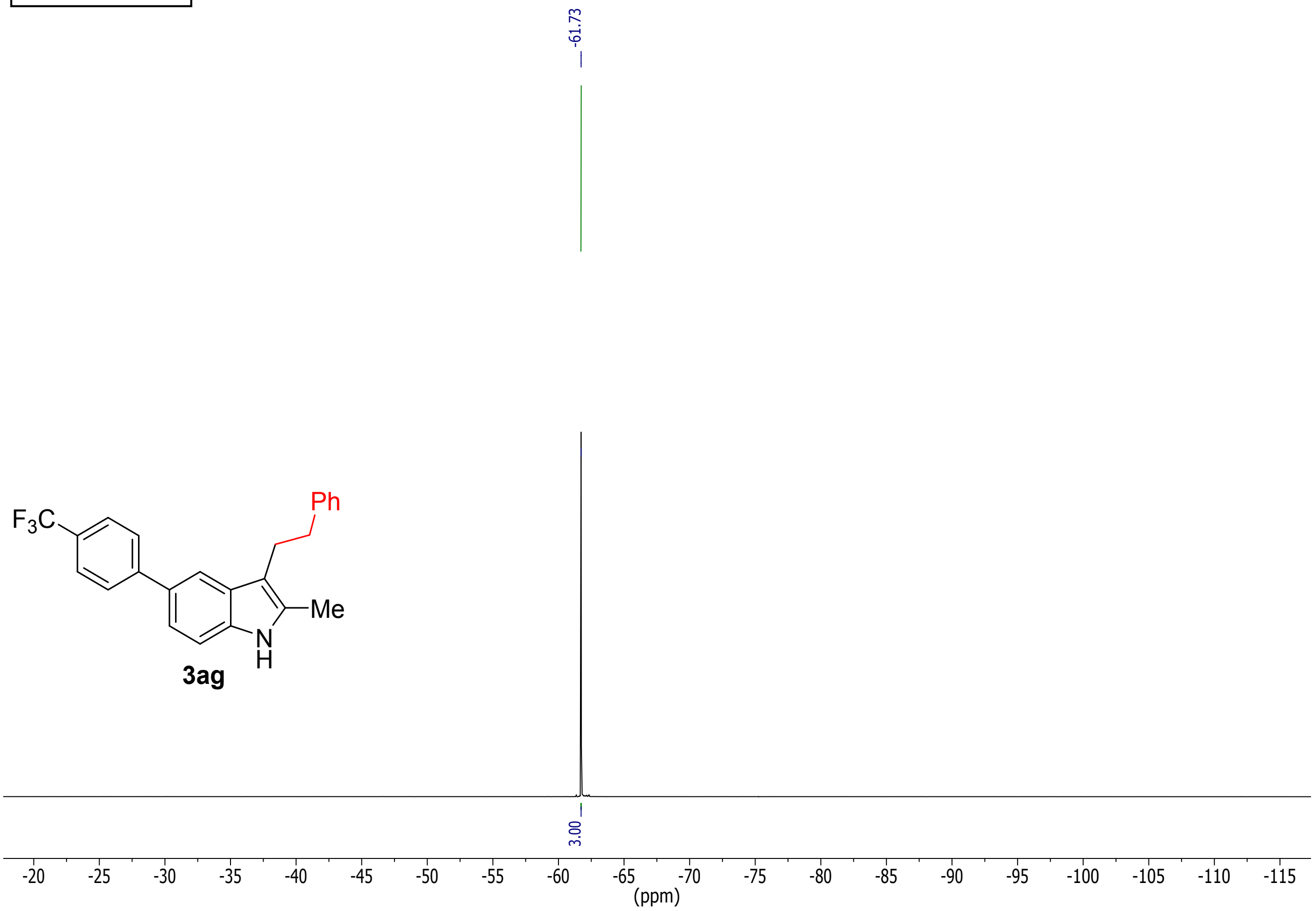

-61.73
F3C
Ph
Me
N
H
3ag
3.00
-20
-25
-30
-35
-40
-45
-50
-55
-60
-65
-70
-75
-80
-85
-90
-95
-100
-105
-110
-115
(ppm)

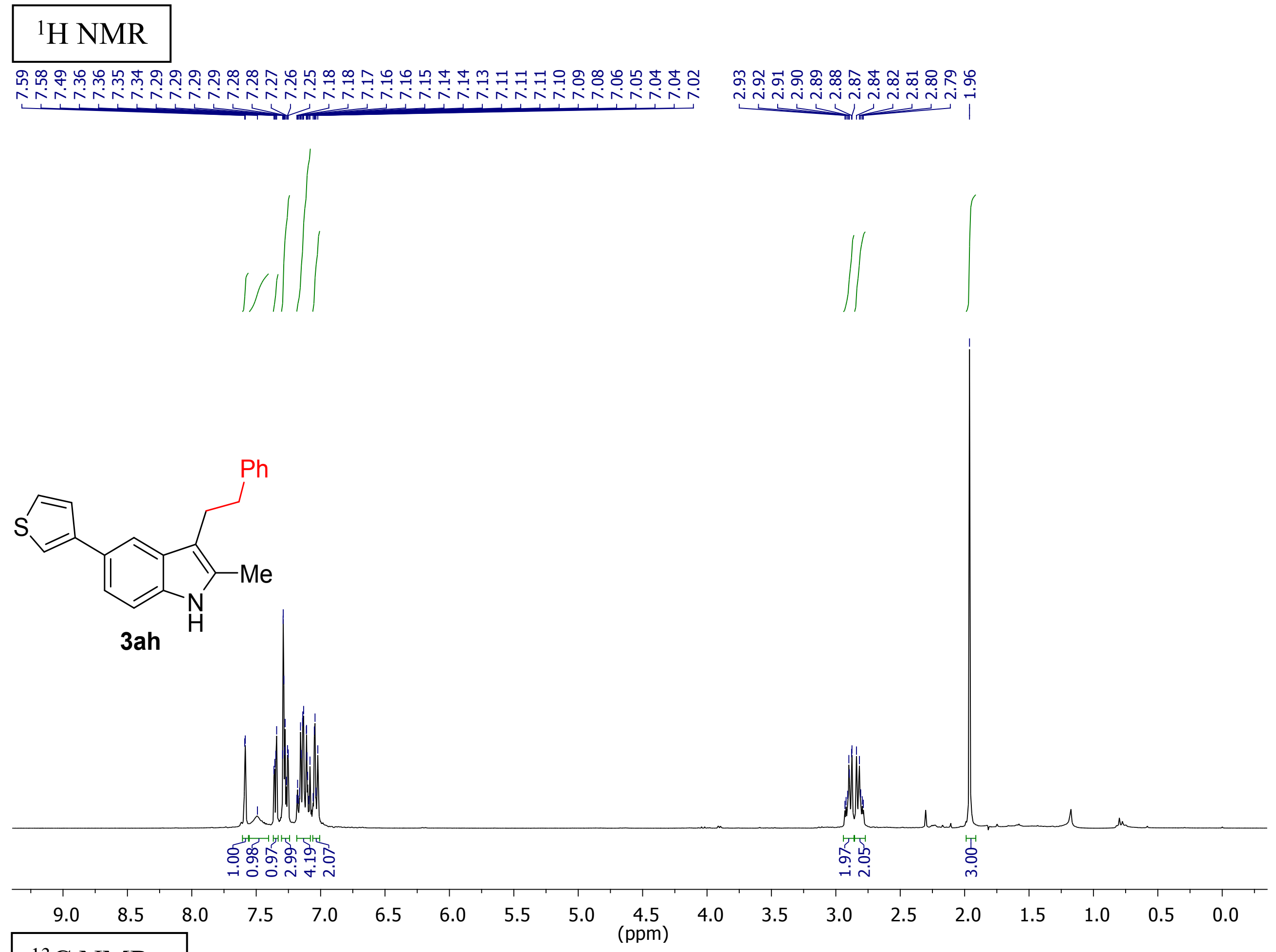

^{1}H NMR
7.59 7.58 7.49 7.36 7.36 7.35 7.34 7.29 7.29 7.29 7.29 7.28 7.28 7.27 7.26 7.25 7.18 7.18 7.17 7.16 7.16 7.15 7.14 7.14 7.13 7.11 7.11 7.11 7.10 7.09 7.08 7.06 7.05 7.04 7.04 7.02
2.93 2.92 2.91 2.90 2.89 2.88 2.87 2.84 2.82 2.81 2.80 2.79
1.96
Ph
S
Me
N
H
3ah
1.00 0.98 0.97 2.99 4.19 2.07
1.97 2.05
3.00
9.0 8.5 8.0 7.5 7.0 6.5 6.0 5.5 5.0 4.5 4.0 3.5 3.0 2.5 2.0 1.5 1.0 0.5 0.0
(ppm)

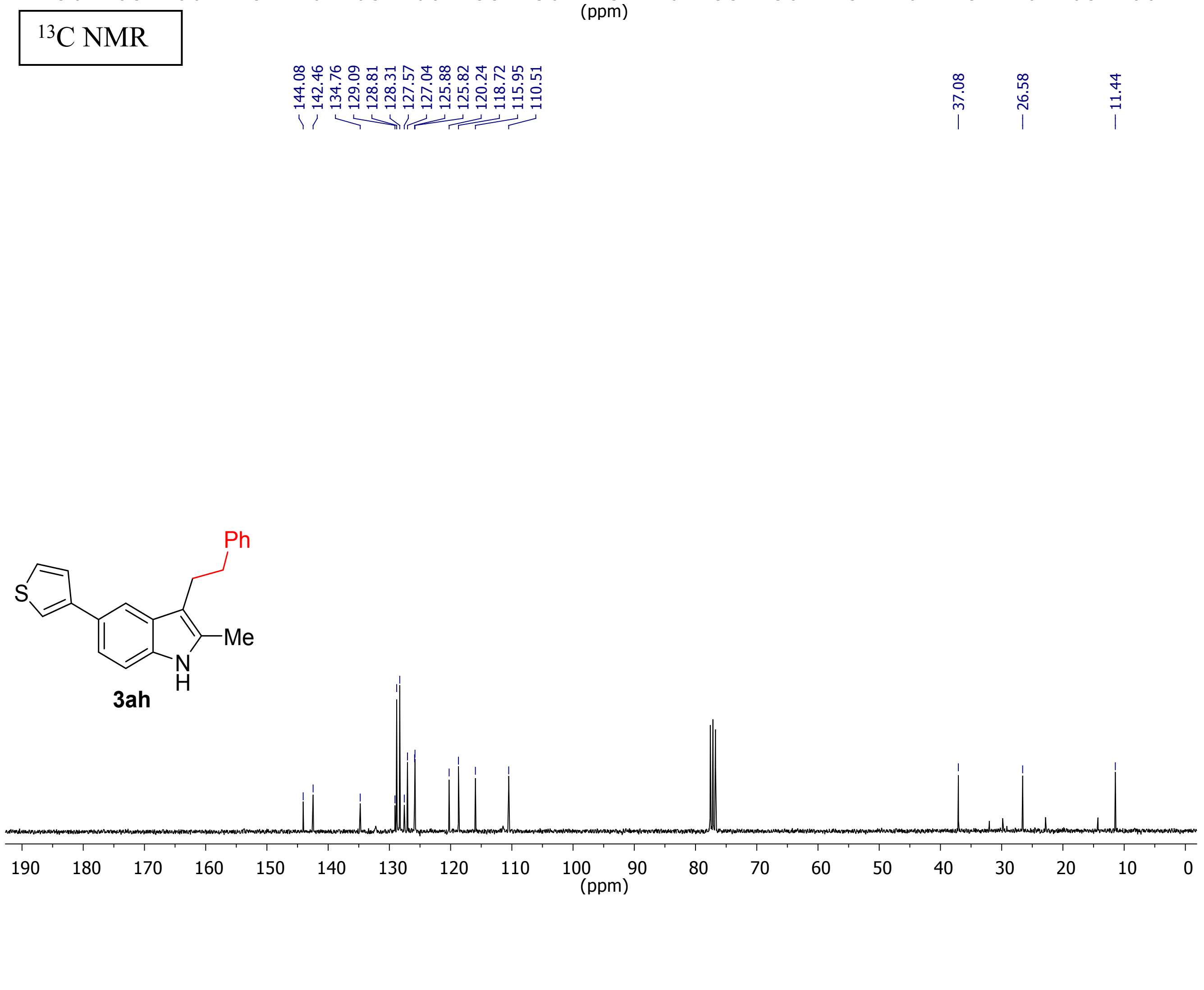

^{13}C NMR
144.08 142.46 134.76 129.09 128.81 128.31 127.57 127.04 125.88 125.82 120.24 118.72 115.95 110.51
37.08
26.58
11.44
Ph
S
Me
N
H
3ah
190 180 170 160 150 140 130 120 110 100 90 80 70 60 50 40 30 20 10 0
(ppm)

¹H NMR

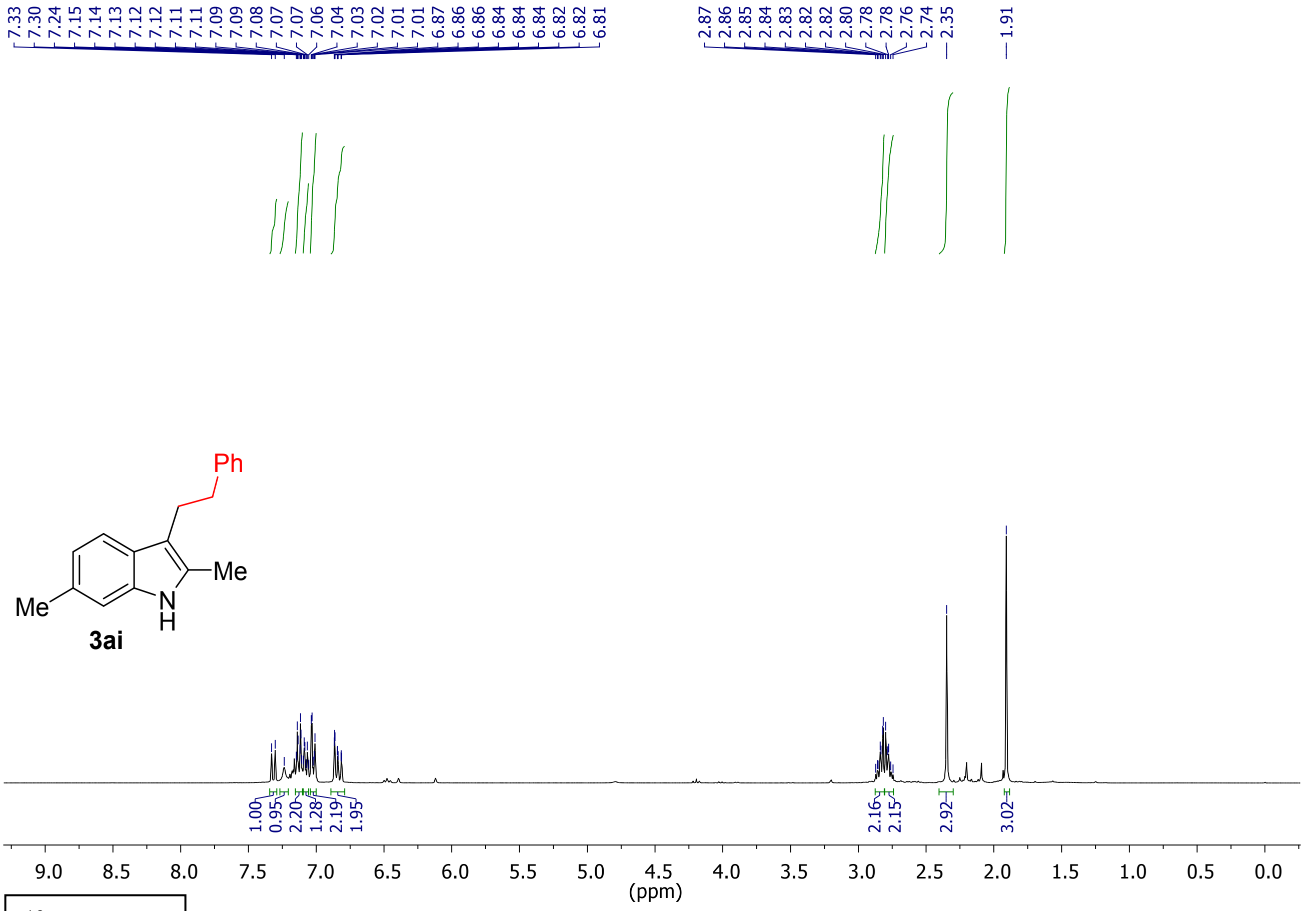

¹³C NMR

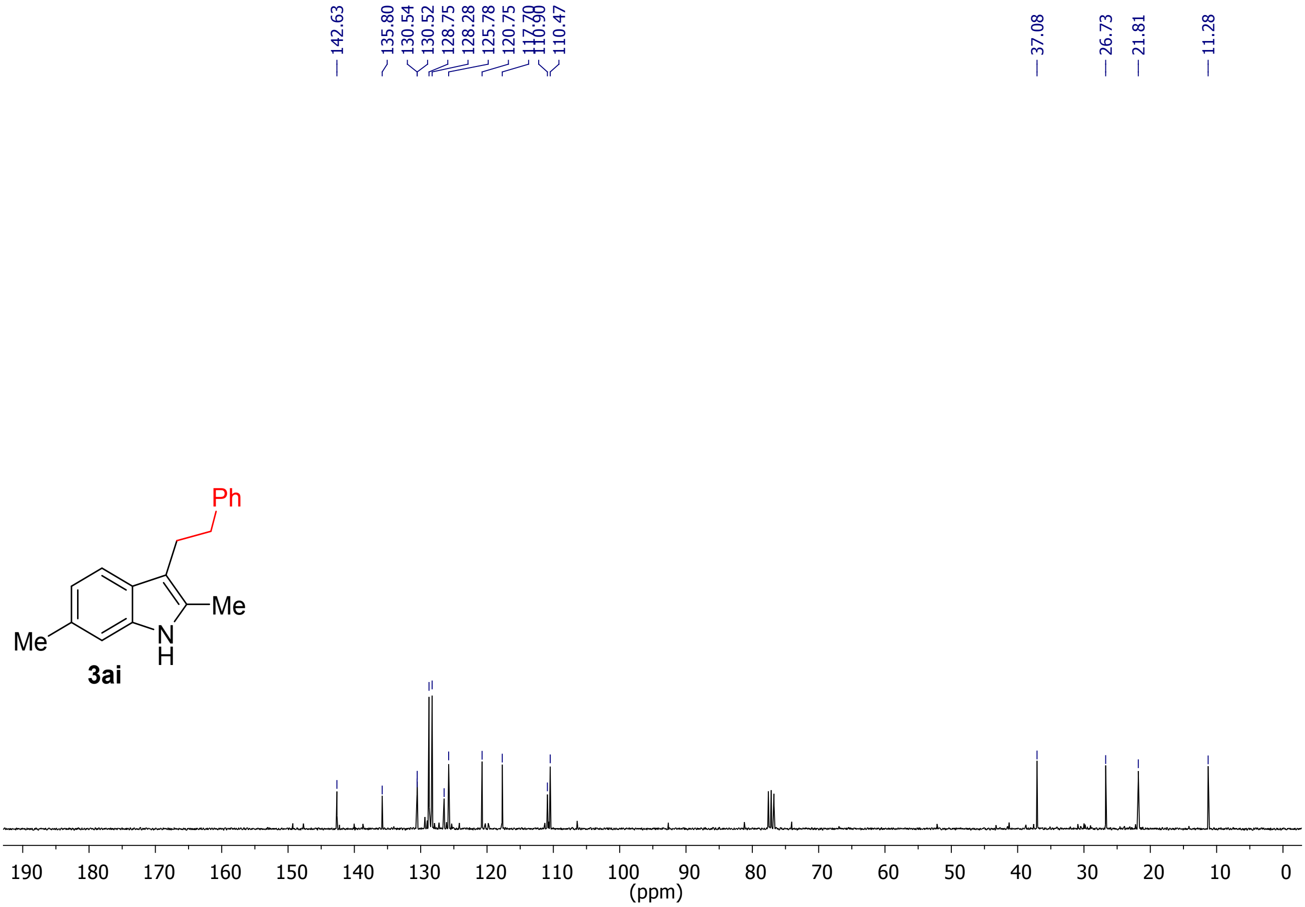

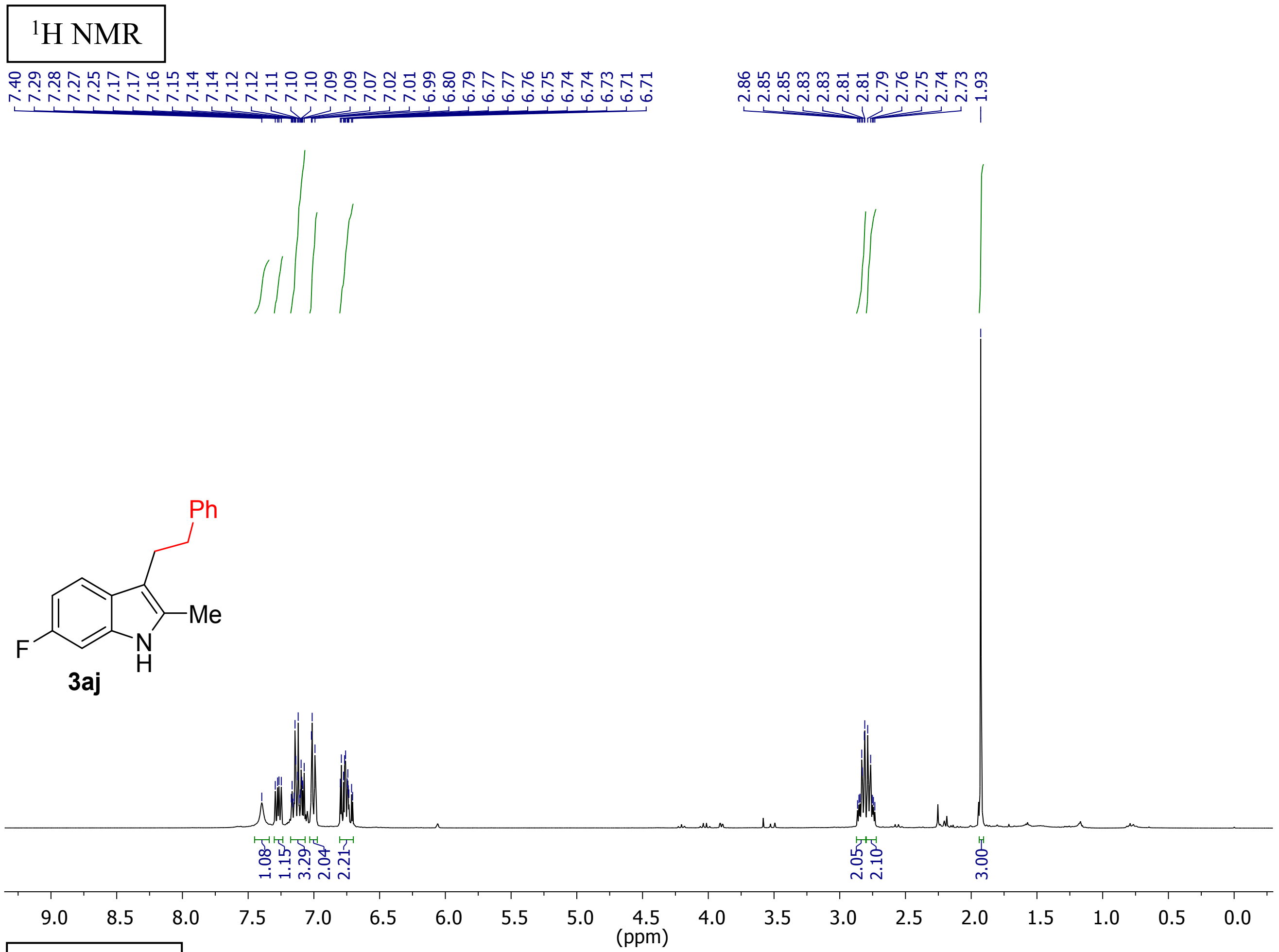

1H NMR
7.40 7.29 7.28 7.27 7.25 7.17 7.17 7.16 7.15 7.14 7.14 7.12 7.12 7.11 7.10 7.10 7.09 7.09 7.07 7.02 7.01 6.99 6.80 6.79 6.77 6.77 6.76 6.75 6.74 6.74 6.73 6.71 6.71
2.86 2.85 2.85 2.83 2.83 2.81 2.81 2.79 2.76 2.75 2.74 2.73 1.93
Ph
Me
F
N
H
3aj
1.08 1.15 3.29 2.04 2.21 2.05 2.10 3.00
9.0 8.5 8.0 7.5 7.0 6.5 6.0 5.5 5.0 4.5 4.0 3.5 3.0 2.5 2.0 1.5 1.0 0.5 0.0
(ppm)

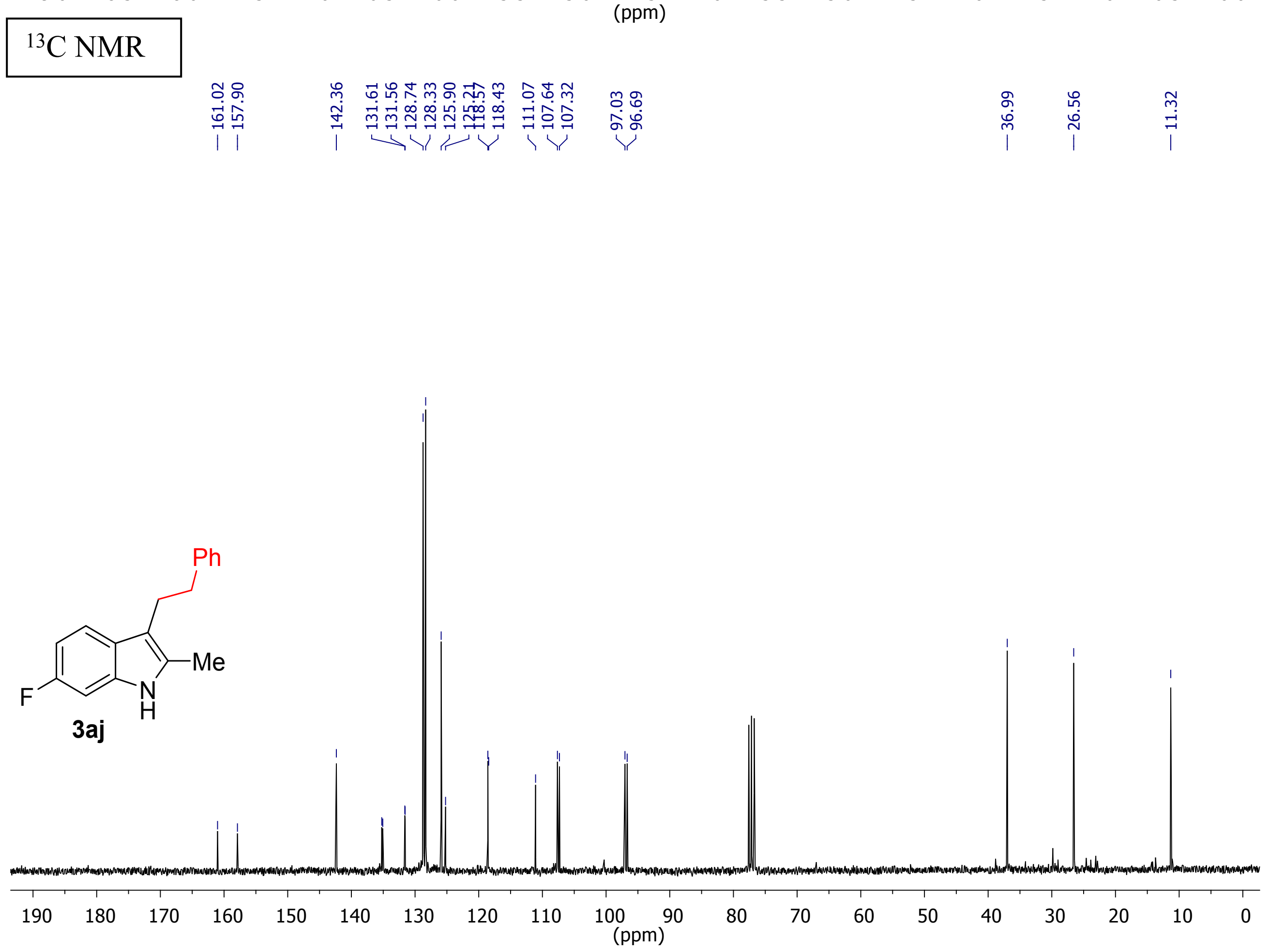

13C NMR
161.02 157.90 142.36 131.61 131.56 128.74 128.33 125.90 125.21 118.57 118.43 111.07 107.64 107.32 97.03 96.69 36.99 26.56 11.32
Ph
Me
F
N
H
3aj
190 180 170 160 150 140 130 120 110 100 90 80 70 60 50 40 30 20 10 0
(ppm)

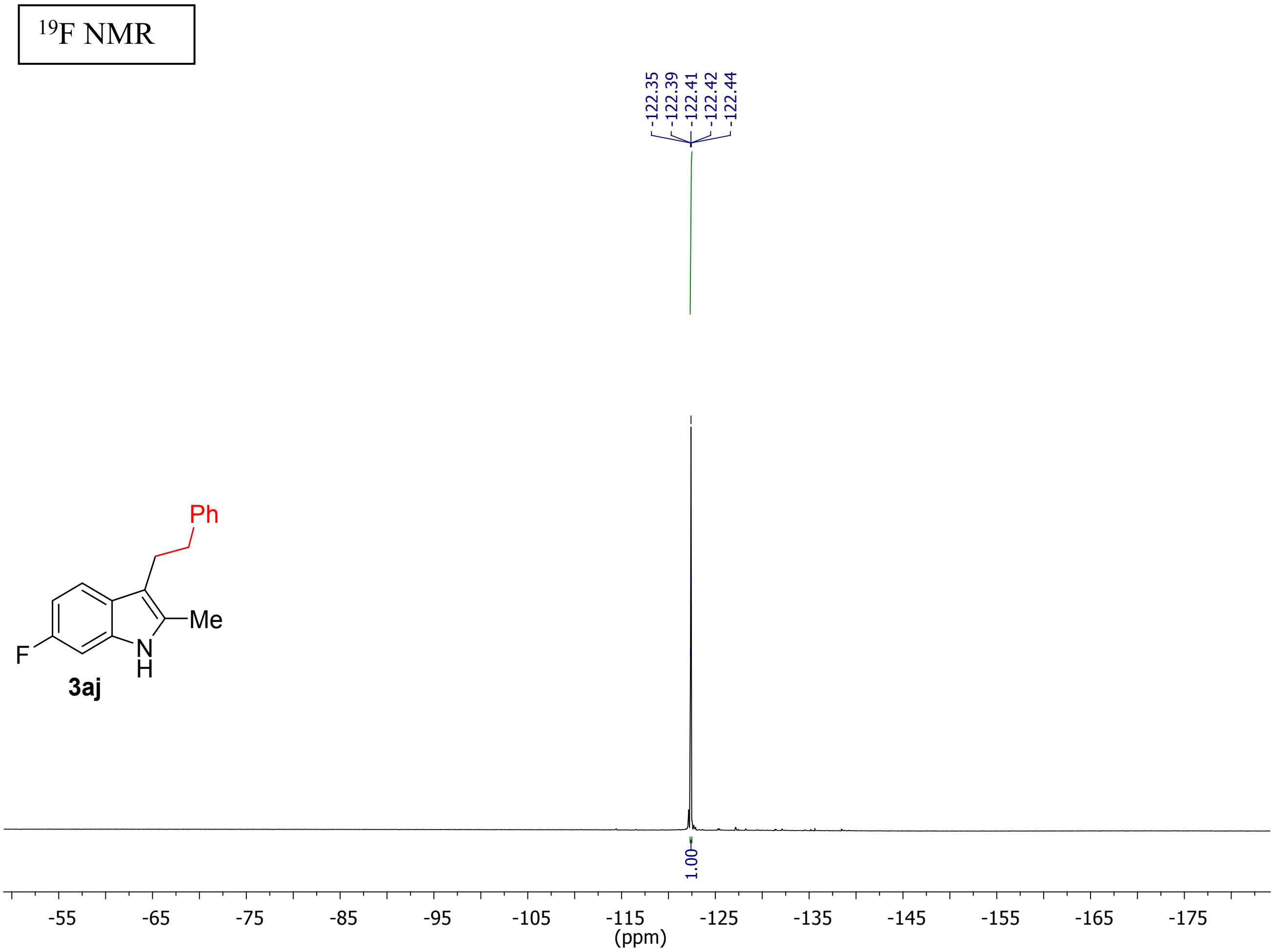

19F NMR
-122.35
-122.39
-122.41
-122.42
-122.44
Ph
Me
F
N
H
3aj
1.00
-55
-65
-75
-85
-95
-105
-115
-125
-135
-145
-155
-165
-175
(ppm)

^{1}H NMR

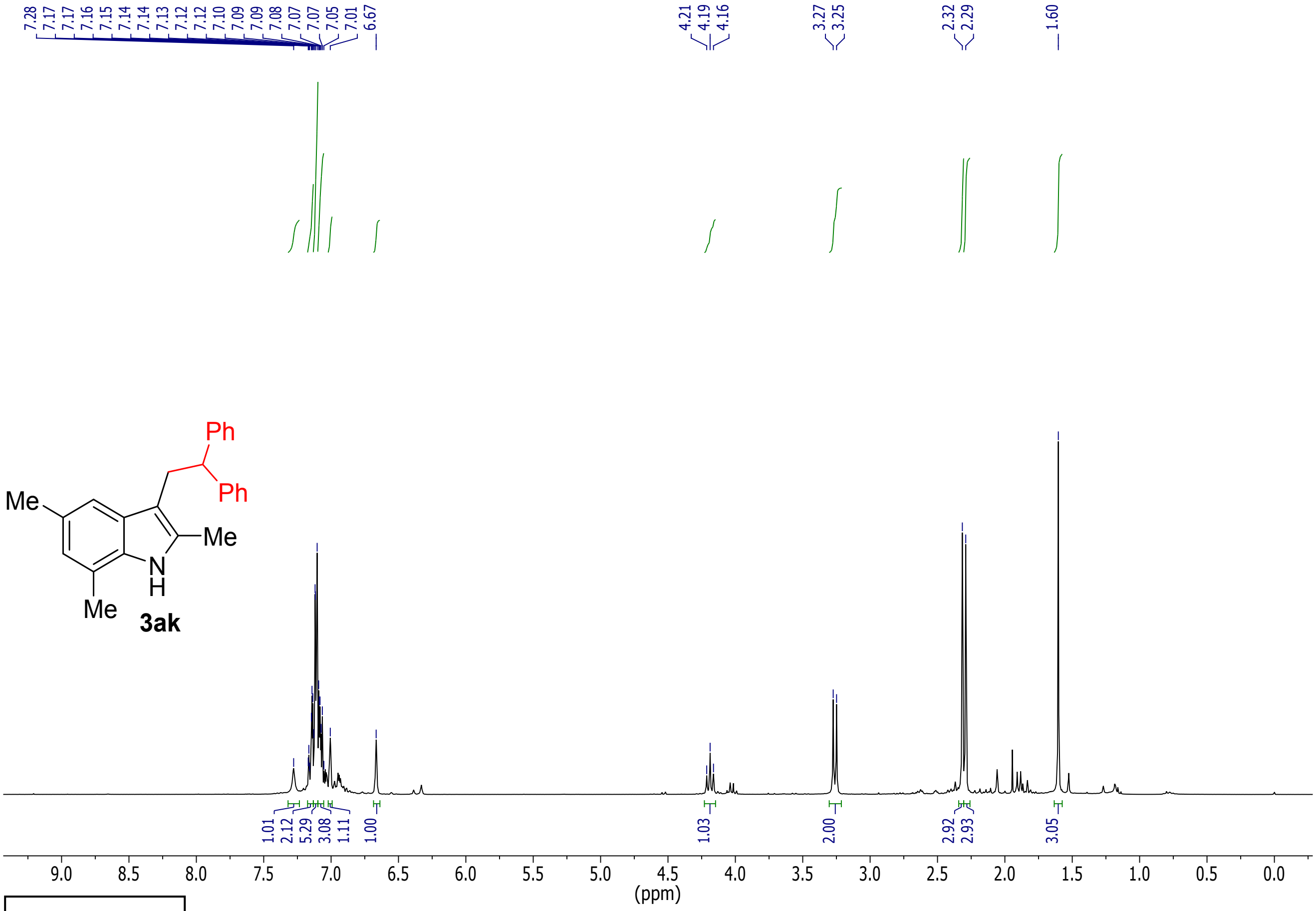

^{13}C NMR

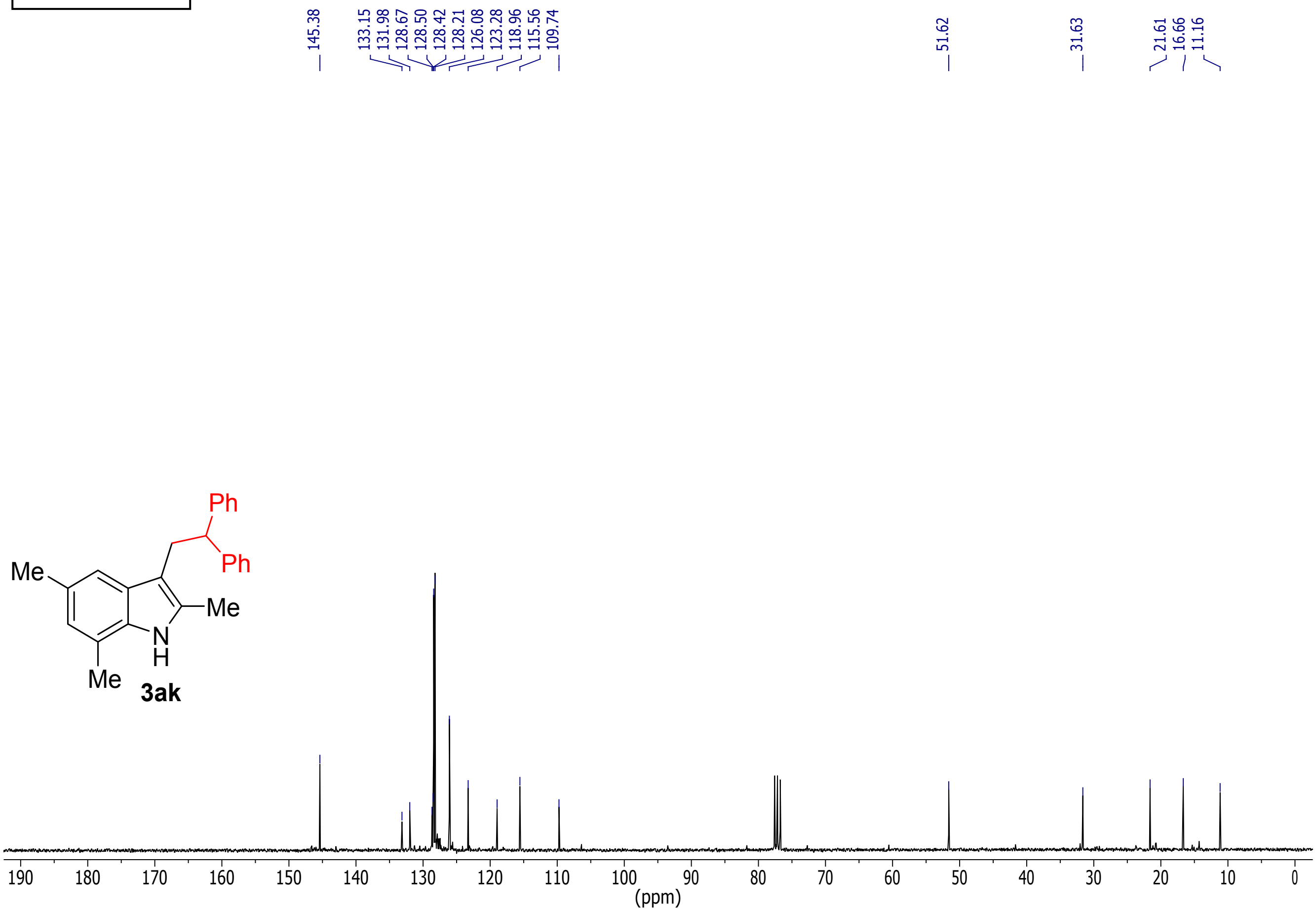

^{1}H NMR

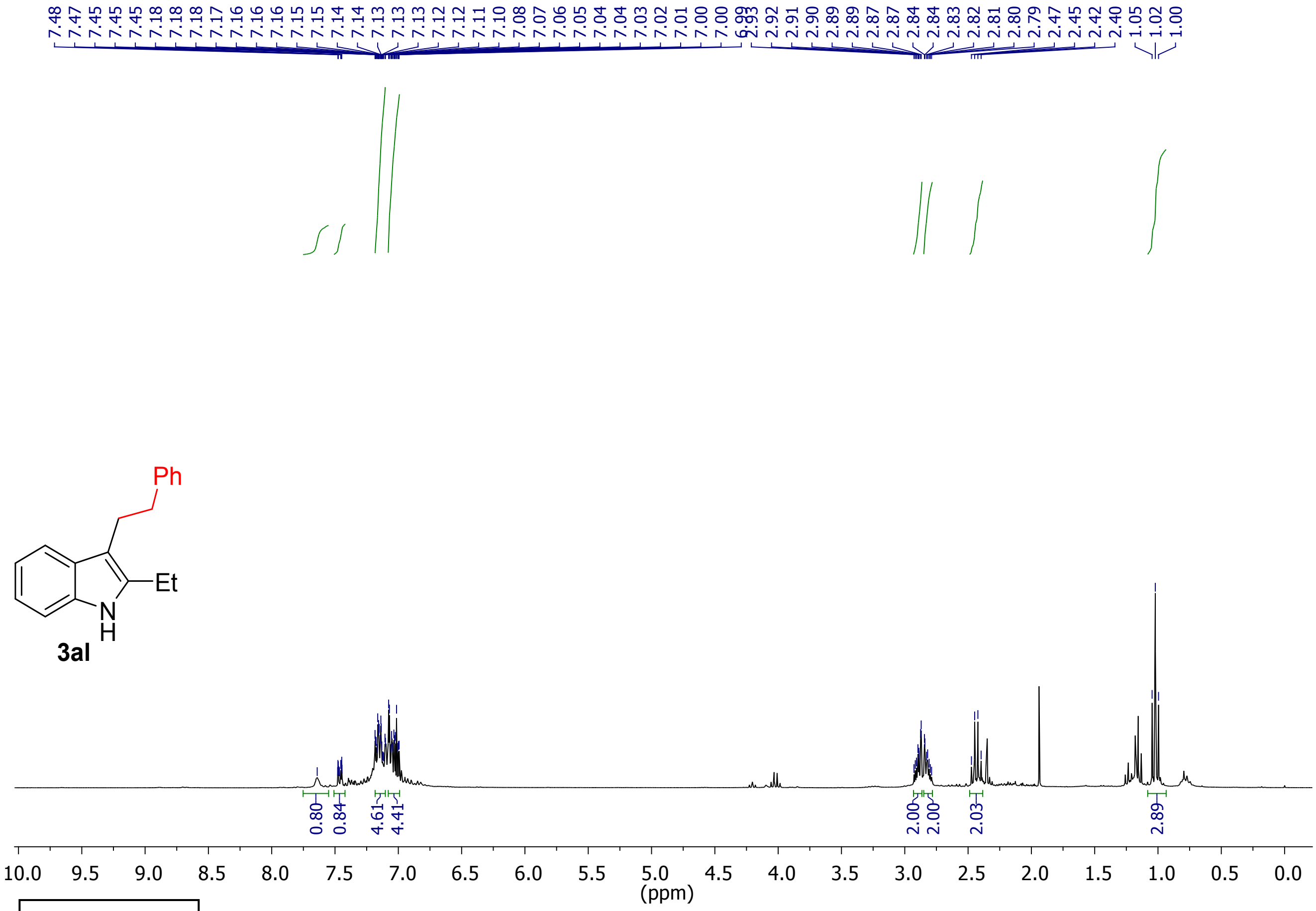

^{13}C NMR

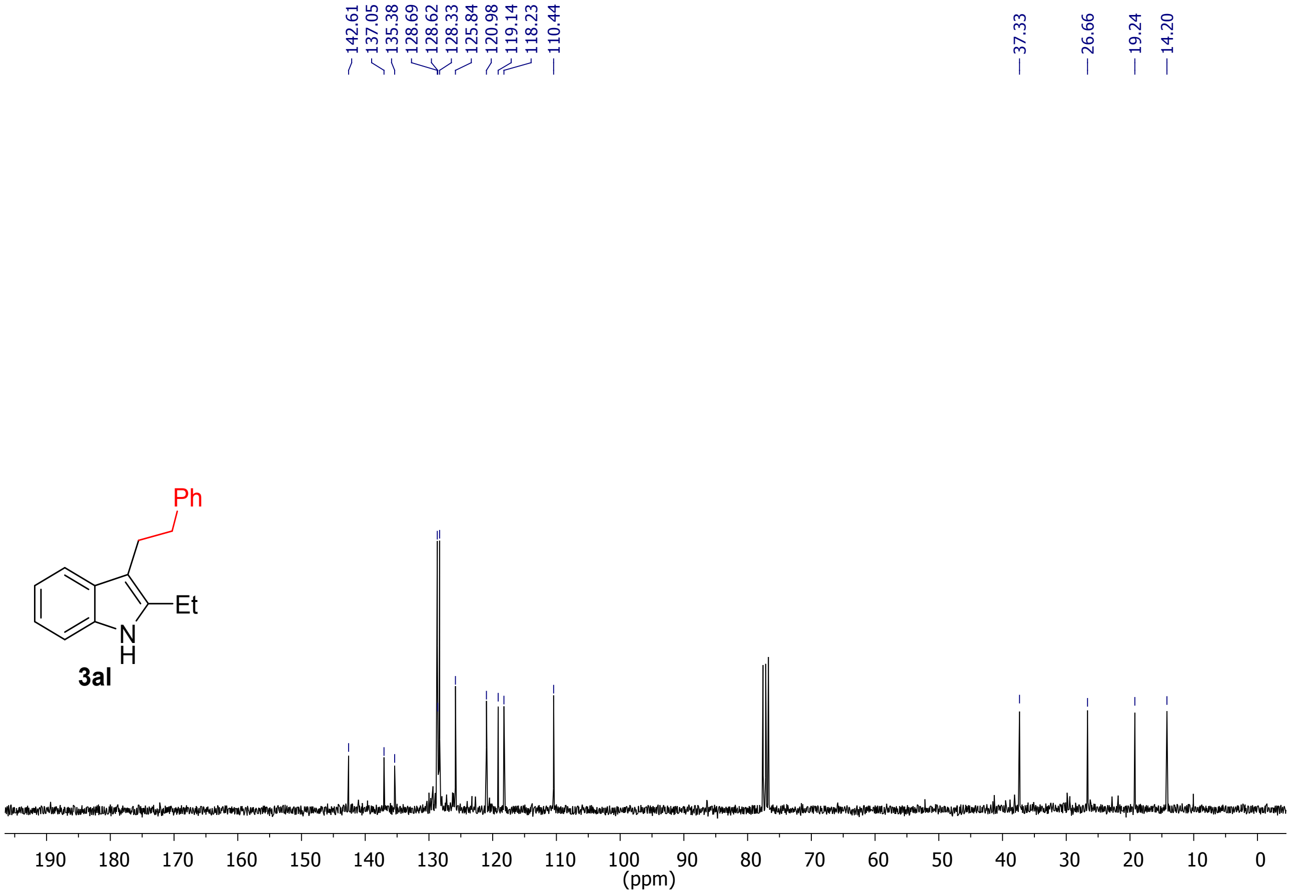

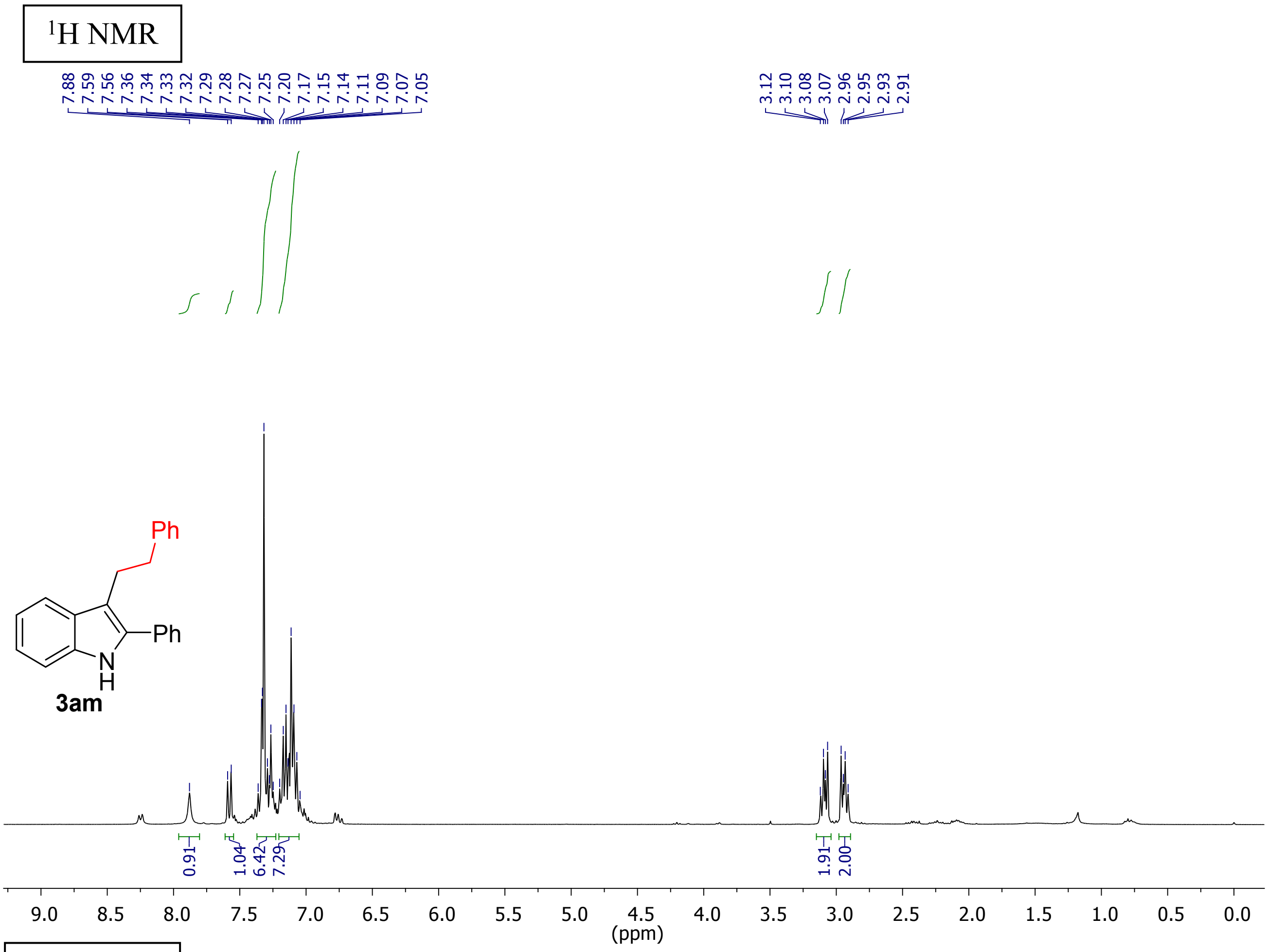
1H NMR
7.88 7.59 7.56 7.36 7.34 7.33 7.32 7.29 7.28 7.27 7.25 7.20 7.17 7.15 7.14 7.11 7.09 7.07 7.05
3.12 3.10 3.08 3.07 2.96 2.95 2.93 2.91
0.91 1.04 6.42 7.29 1.91 2.00
Ph
Ph
N
H
3am
9.0 8.5 8.0 7.5 7.0 6.5 6.0 5.5 5.0 4.5 4.0 3.5 3.0 2.5 2.0 1.5 1.0 0.5 0.0
(ppm)

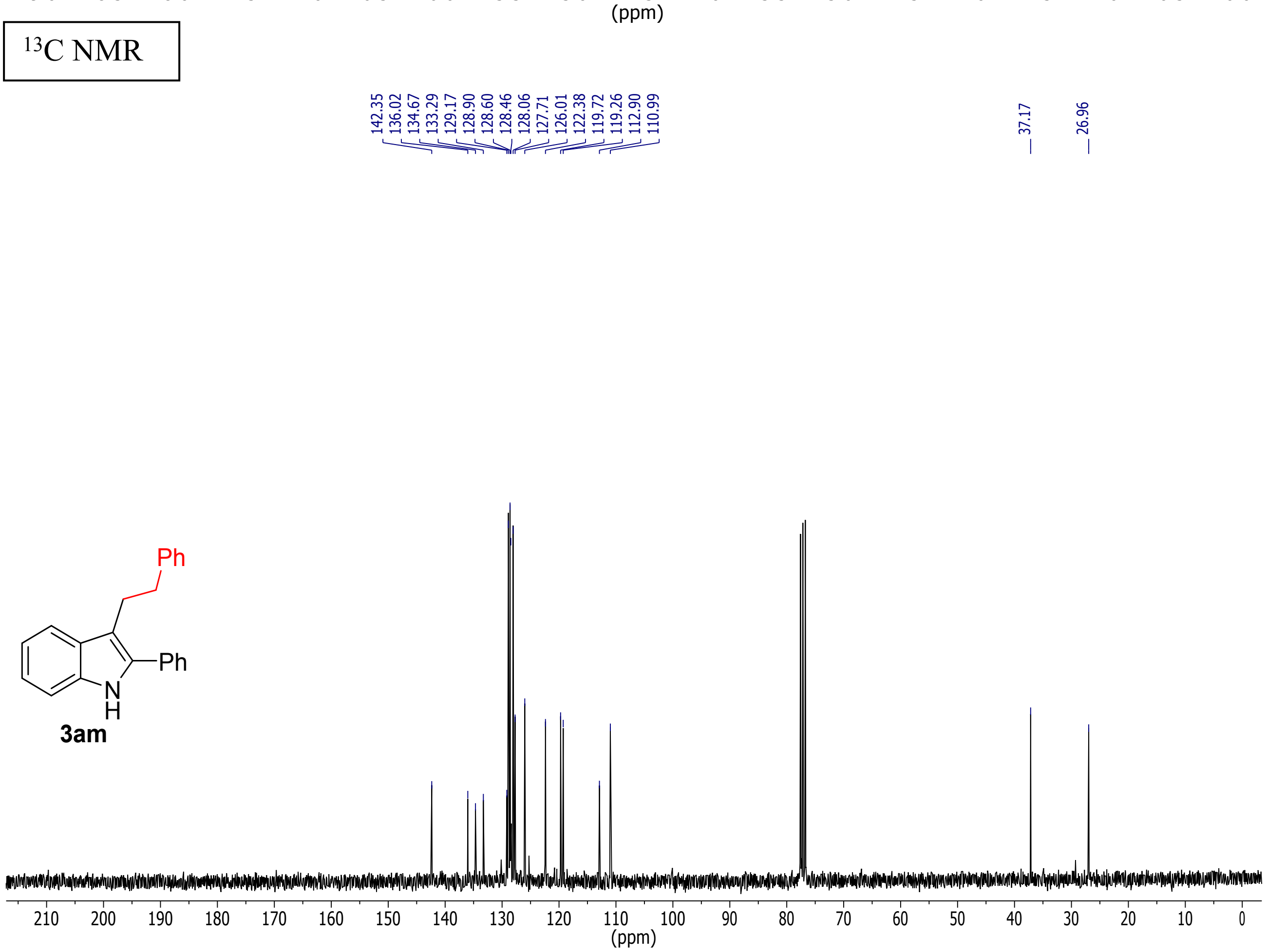
13C NMR
142.35 136.02 134.67 133.29 129.17 128.90 128.60 128.46 128.06 127.71 126.01 122.38 119.72 119.26 112.90 110.99
37.17
26.96
Ph
Ph
N
H
3am
210 200 190 180 170 160 150 140 130 120 110 100 90 80 70 60 50 40 30 20 10 0
(ppm)

^{1}H NMR

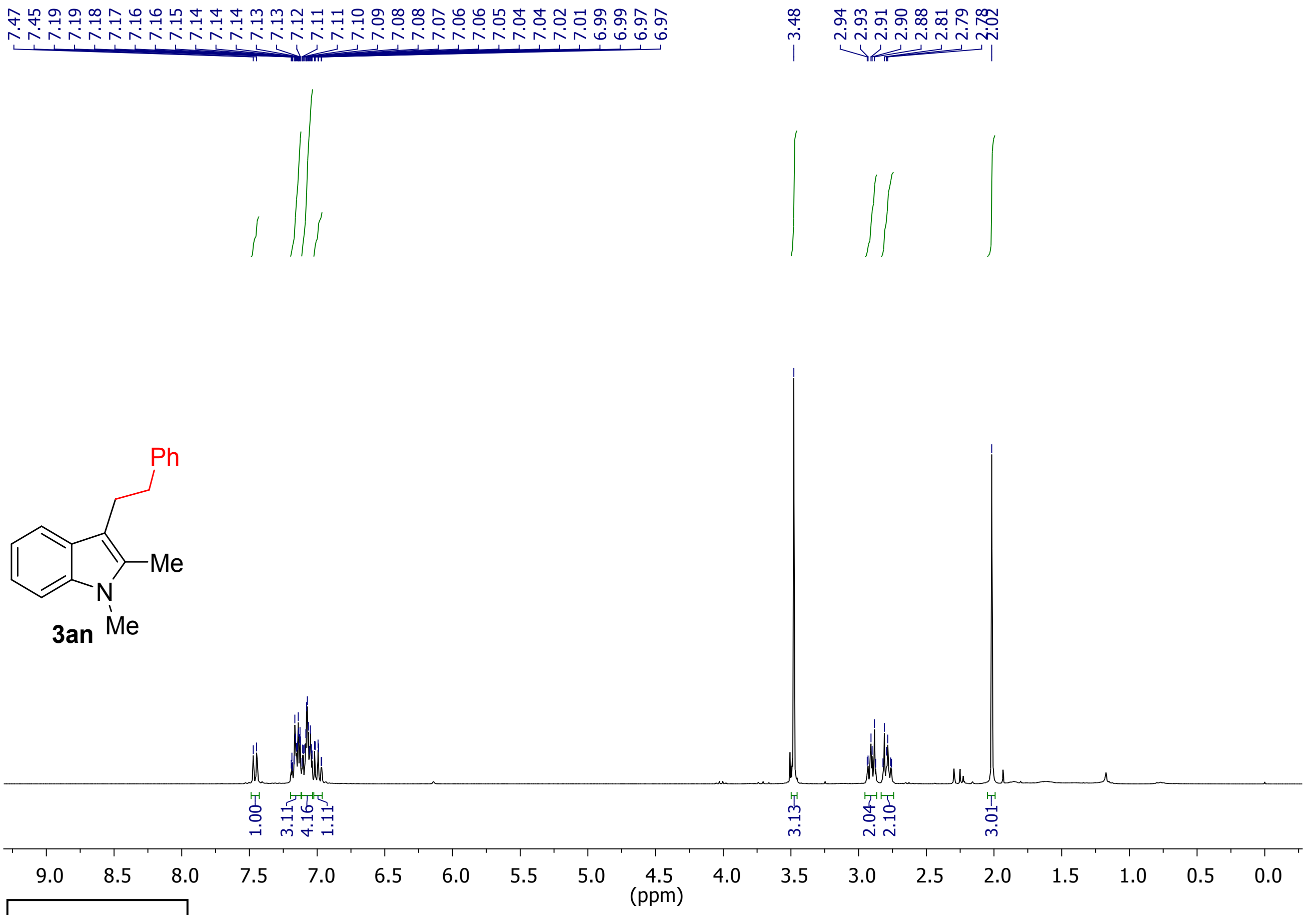

^{13}C NMR

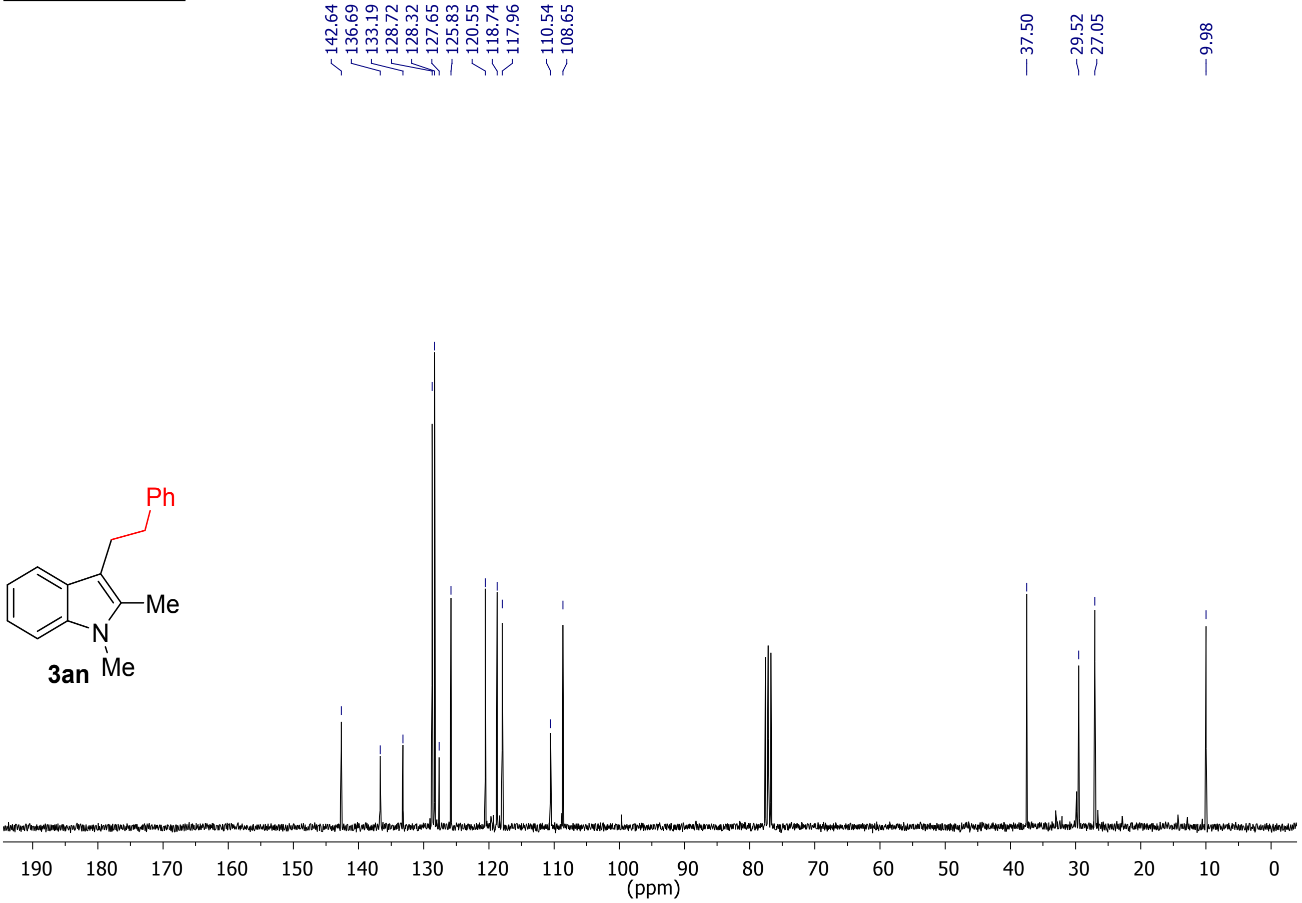

^{1}H NMR

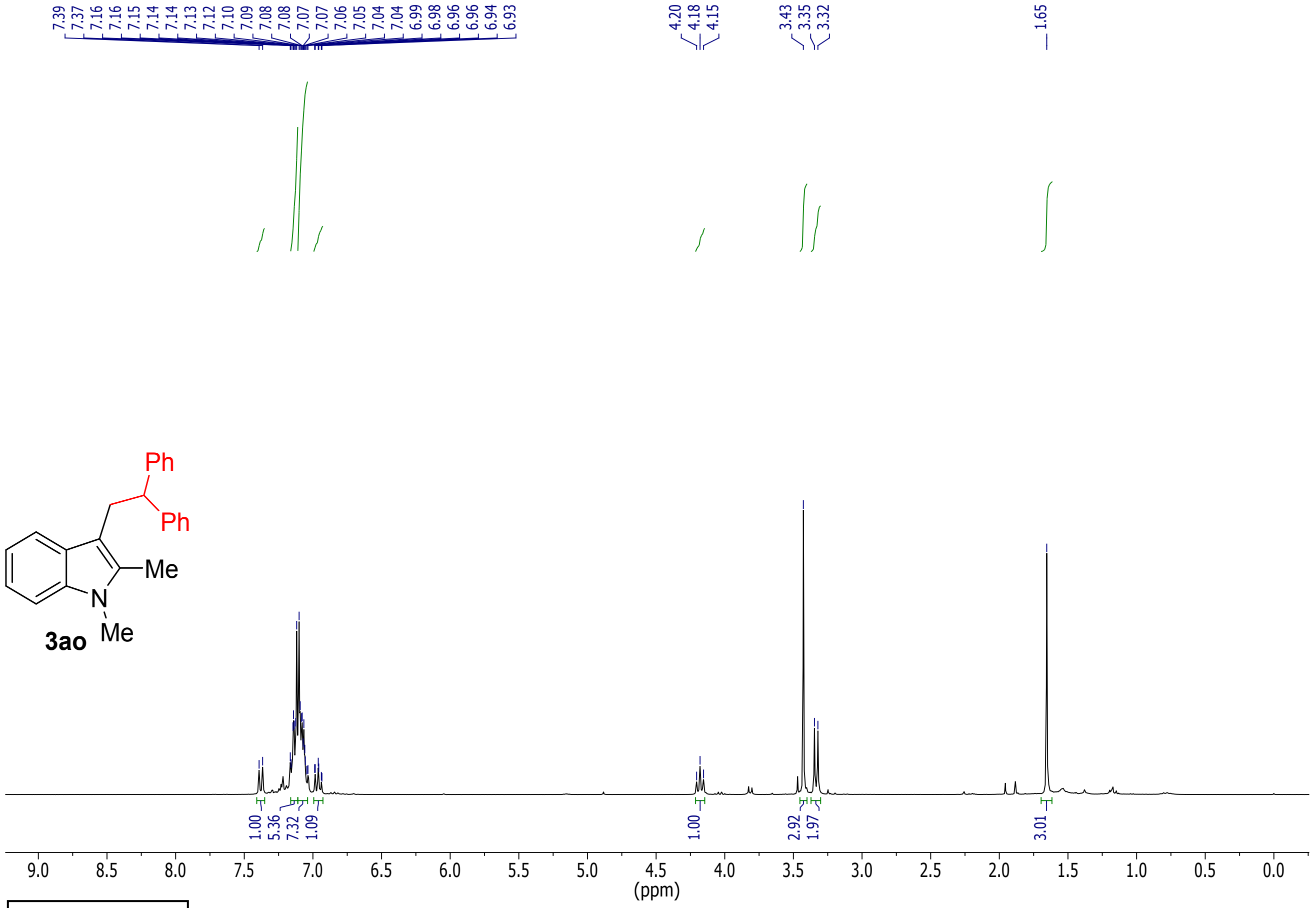

^{13}C NMR

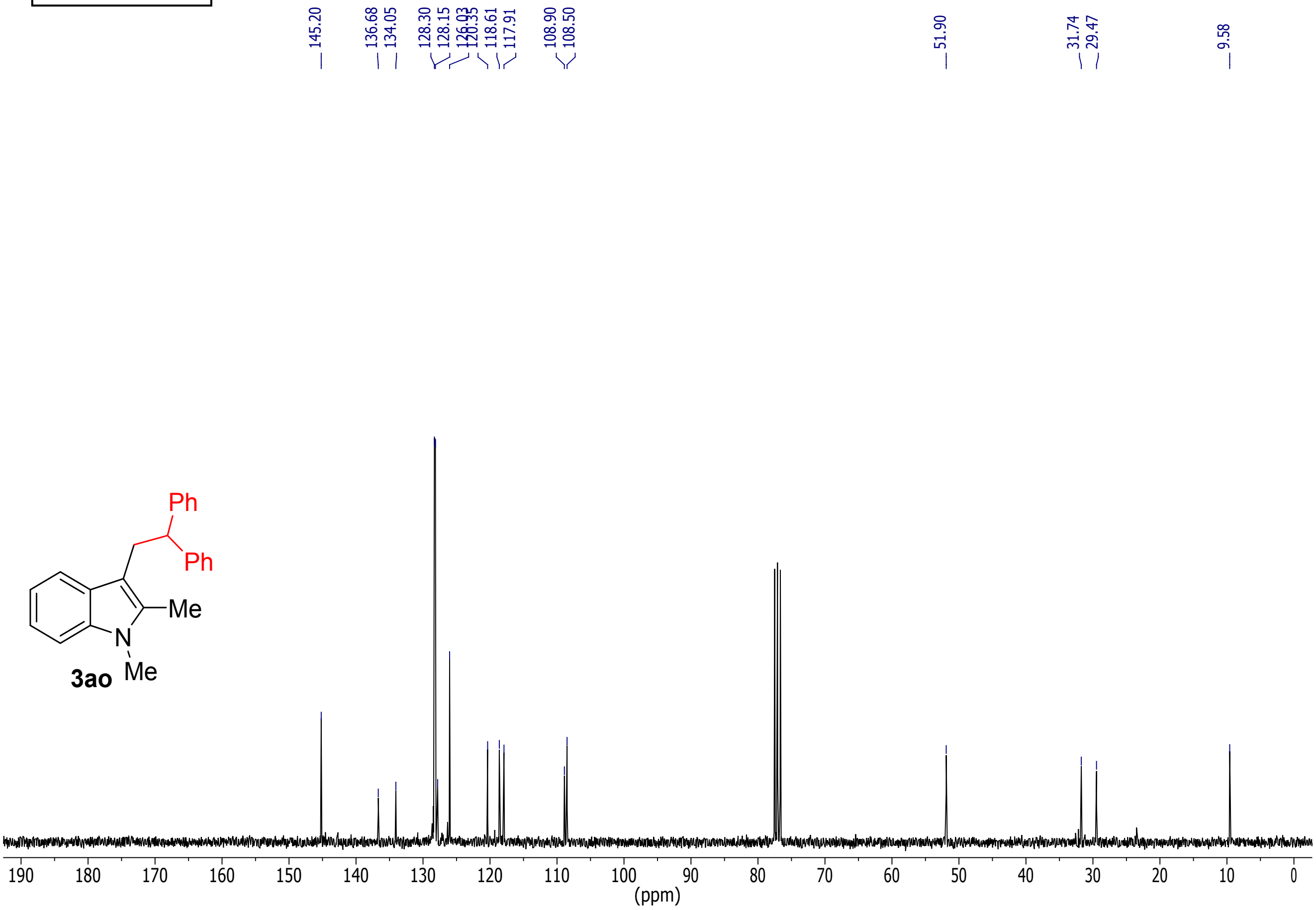

^{1}H NMR

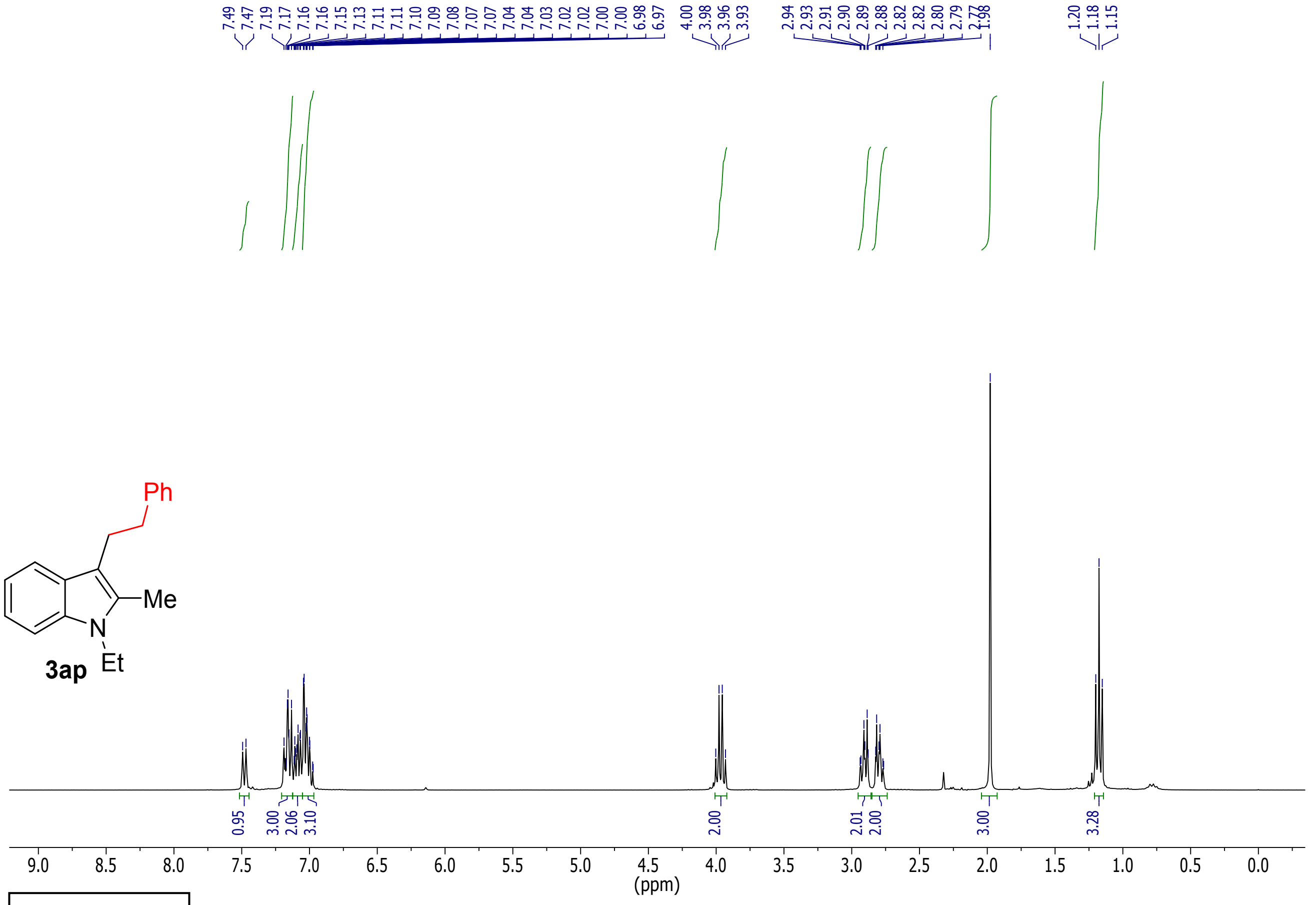

^{13}C NMR

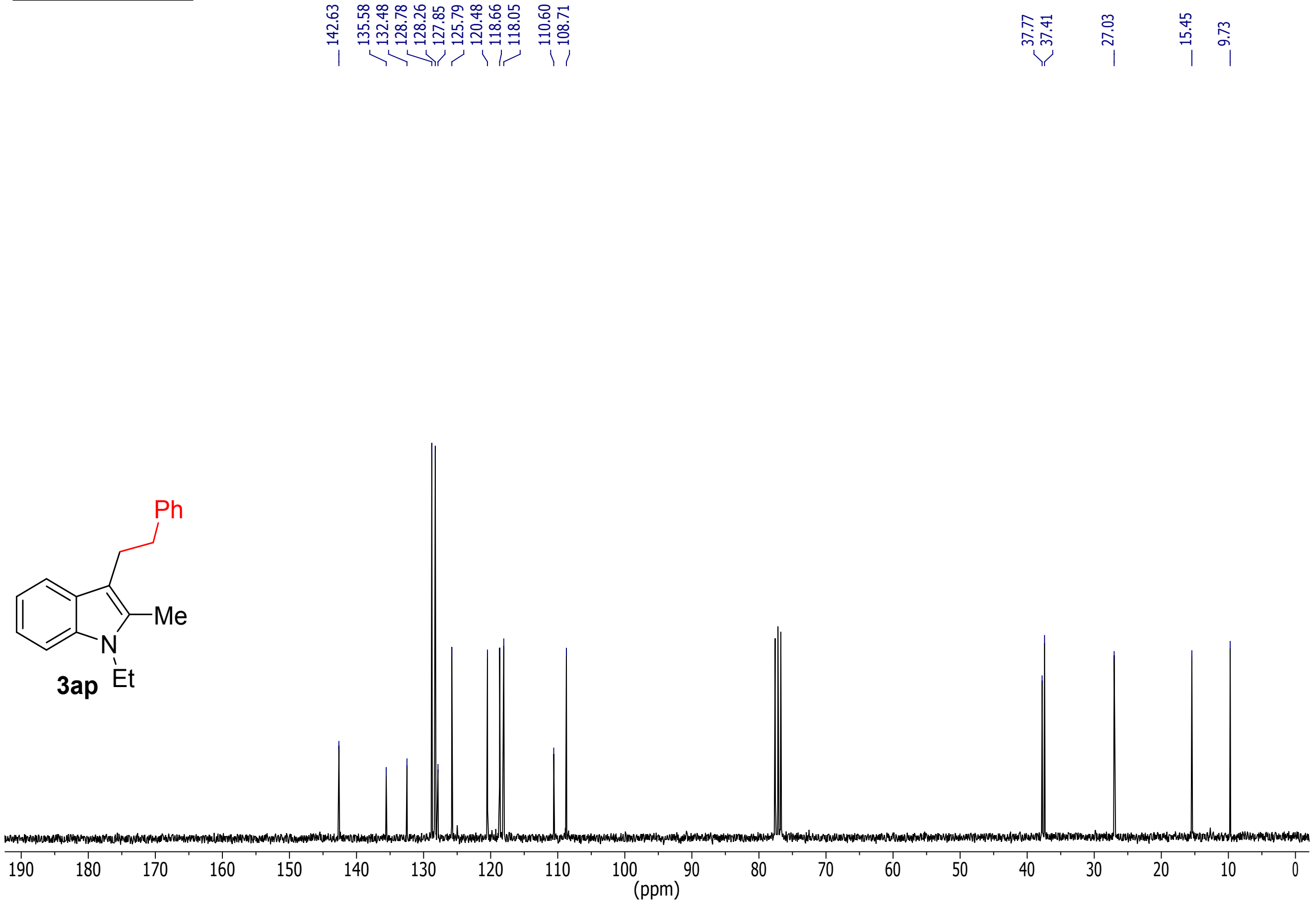

^{1}H NMR

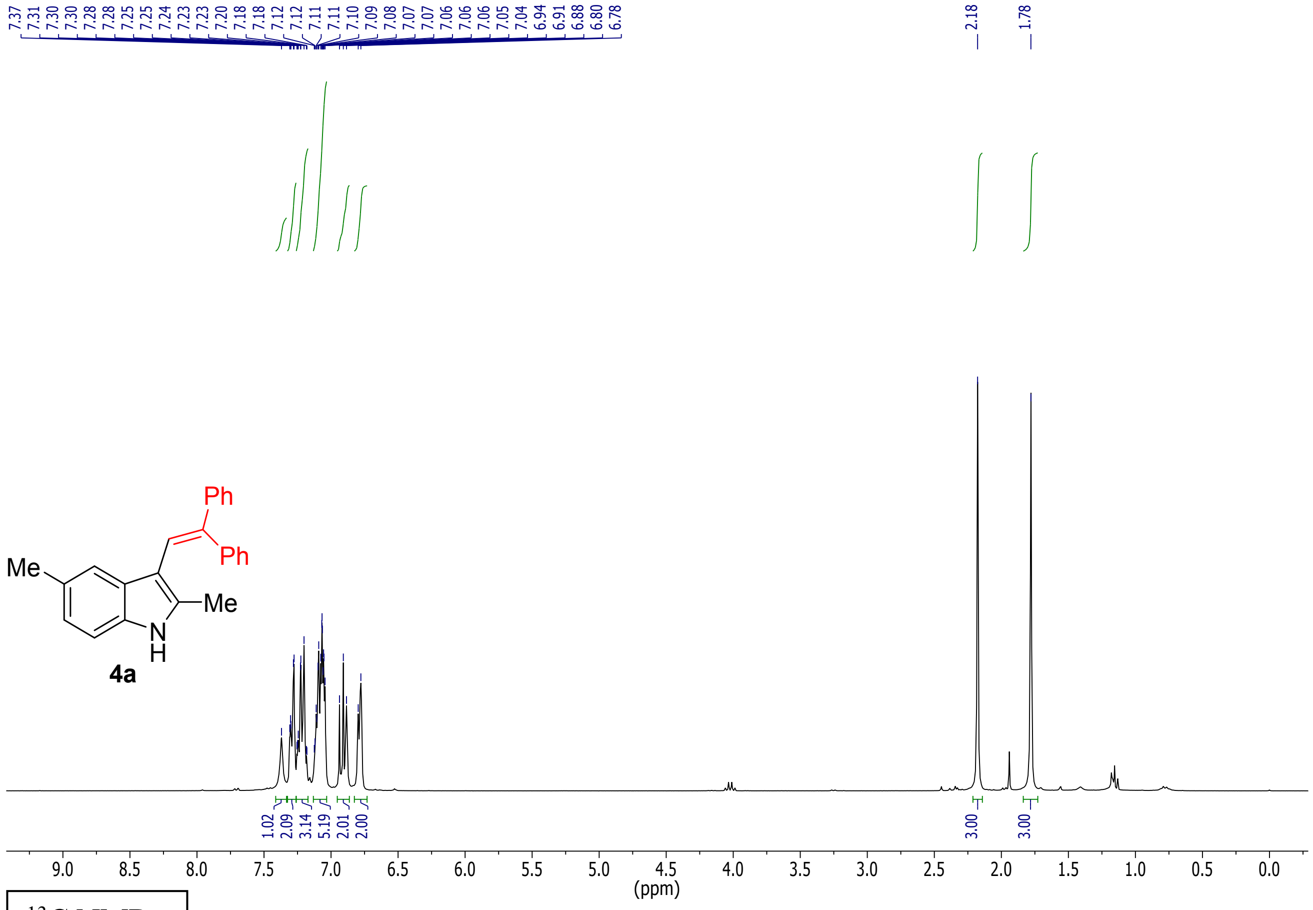

^{13}C NMR

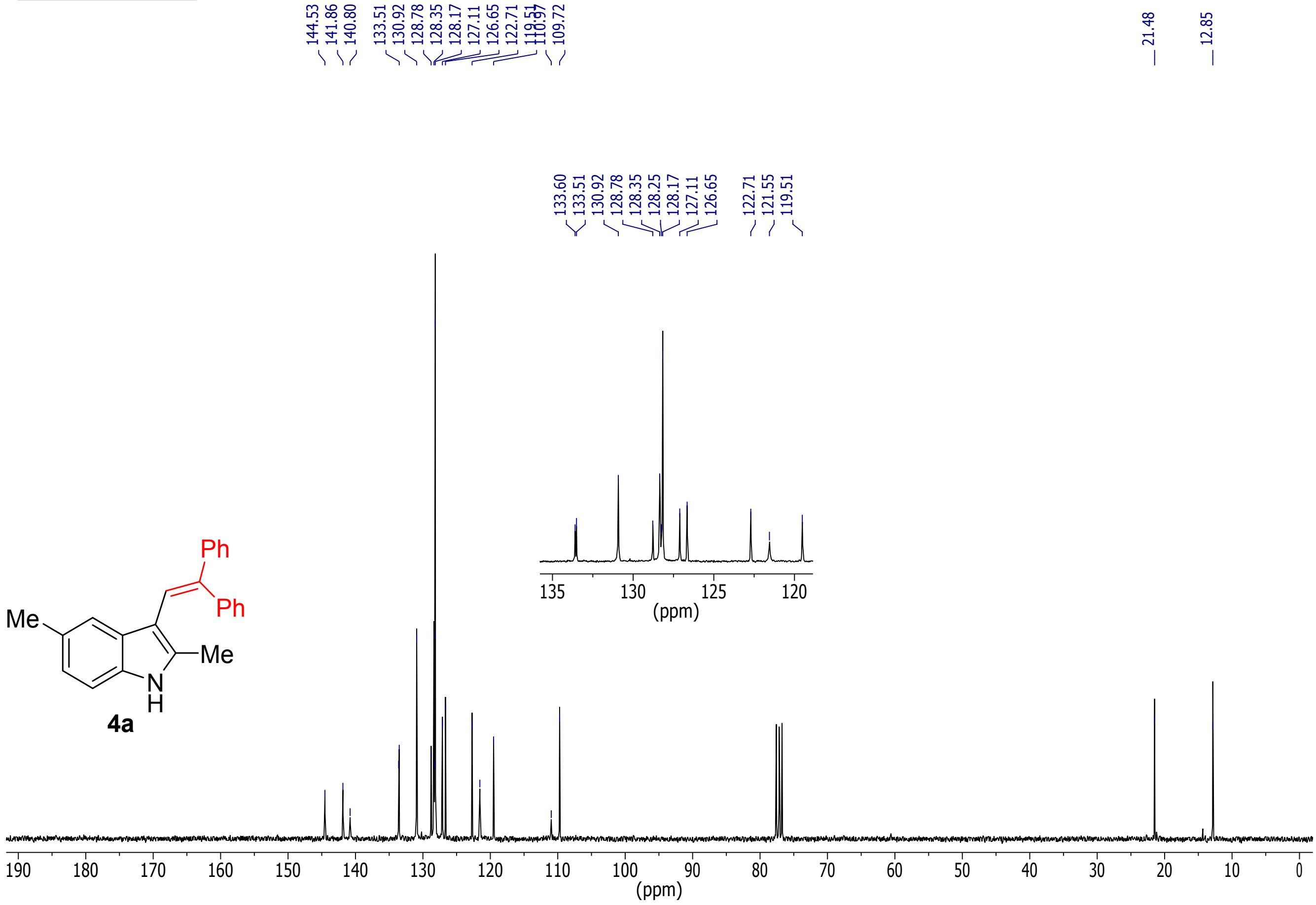

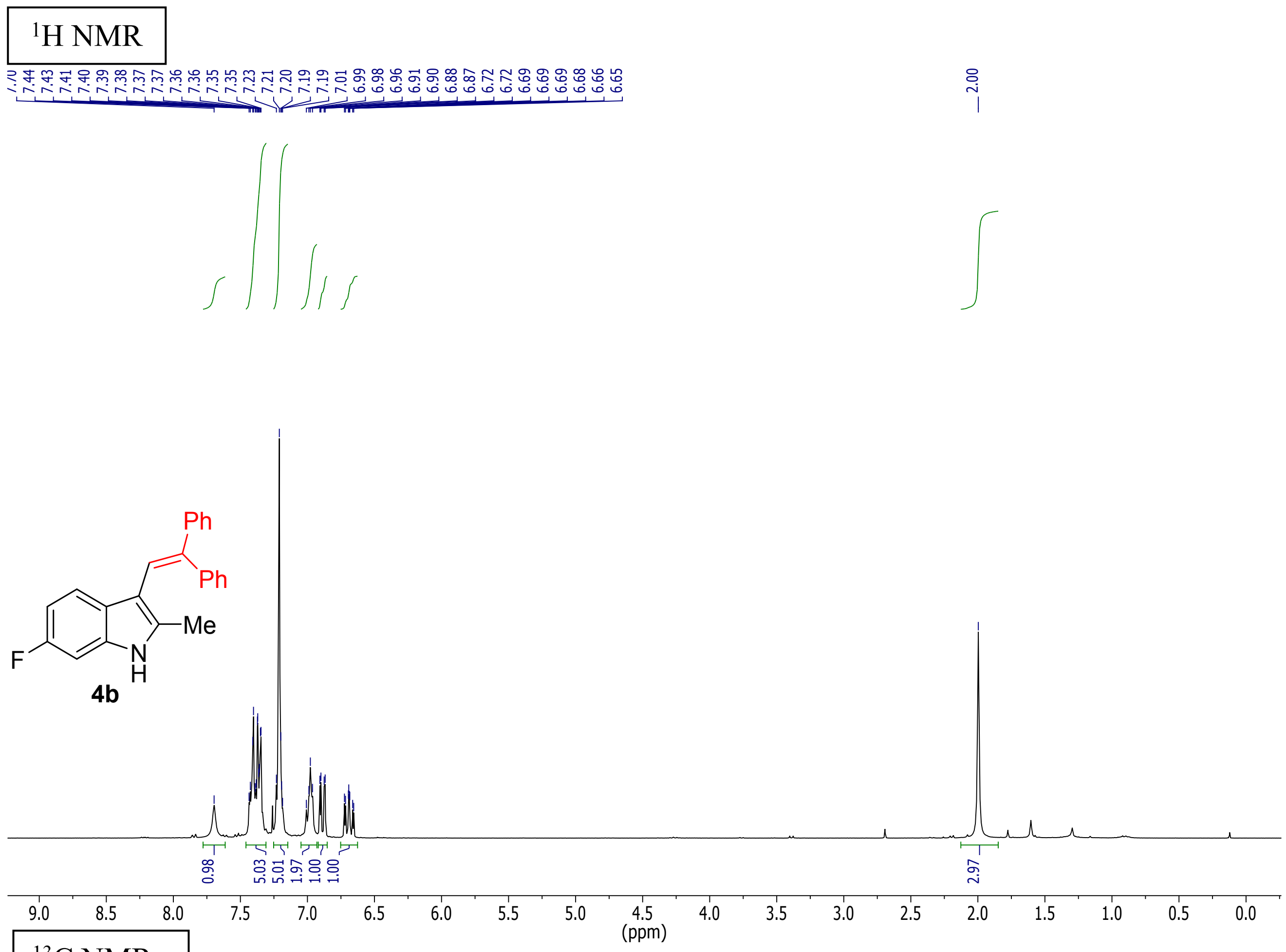

1H NMR
7.70 7.44 7.43 7.41 7.40 7.39 7.38 7.37 7.37 7.36 7.36 7.35 7.35 7.23 7.21 7.20 7.19 7.19 7.01 6.99 6.98 6.96 6.91 6.90 6.88 6.87 6.72 6.72 6.69 6.69 6.69 6.68 6.66 6.65
2.00
Ph
Ph
Me
F
N
H
4b
0.98 5.03 5.01 1.97 1.00 1.00
2.97
9.0 8.5 8.0 7.5 7.0 6.5 6.0 5.5 5.0 4.5 4.0 3.5 3.0 2.5 2.0 1.5 1.0 0.5 0.0
(ppm)

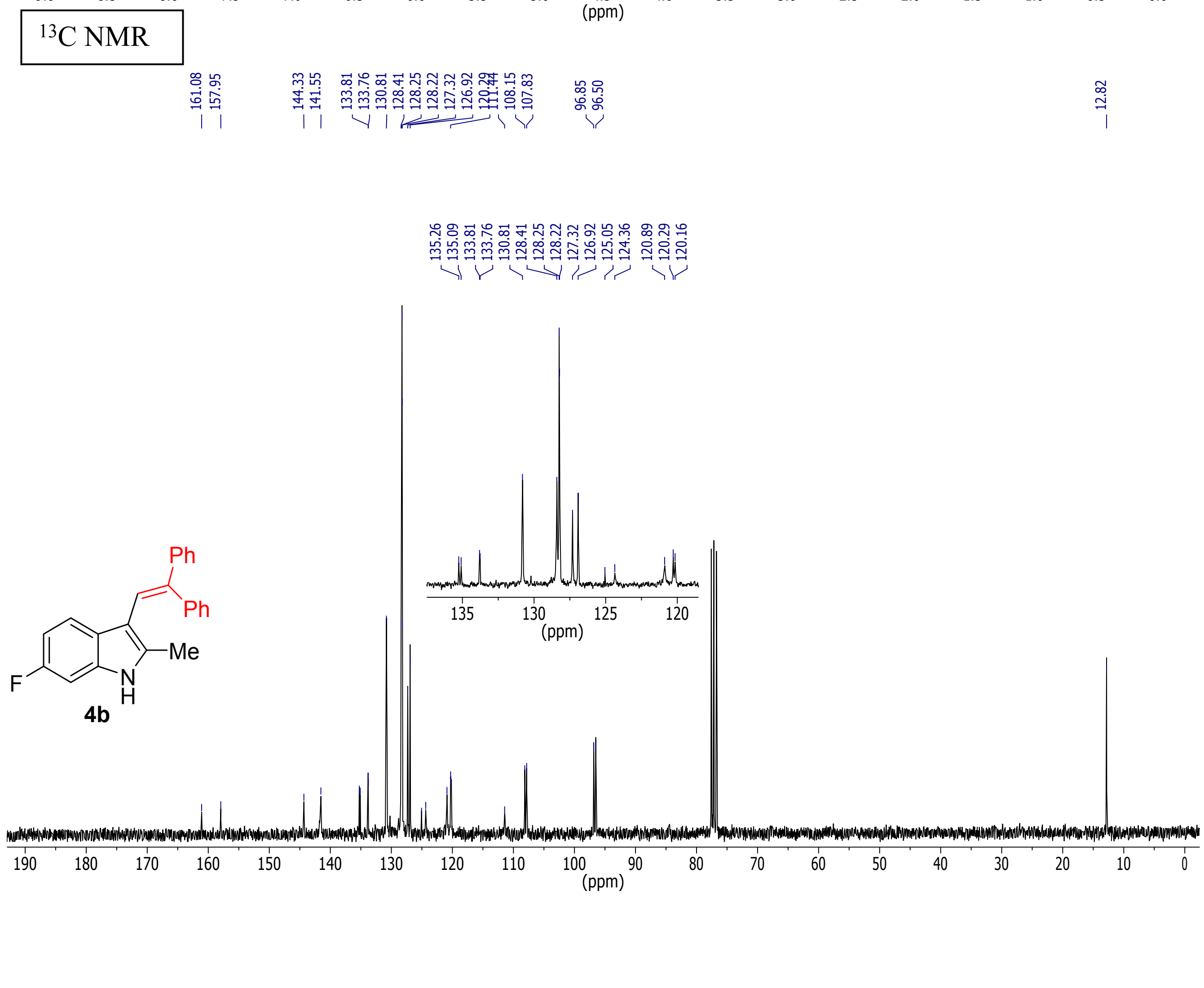

13C NMR
161.08 157.95
144.33 141.55
133.81 133.76 130.81 128.41 128.25 128.22 127.32 126.92 120.29 111.44 108.15 107.83
96.85 96.50
12.82
135.26 135.09 133.81 133.76 130.81 128.41 128.25 128.22 127.32 126.92 125.05 124.36 120.89 120.29 120.16
135 130 125 120
(ppm)
Ph
Ph
Me
F
N
H
4b
190 180 170 160 150 140 130 120 110 100 90 80 70 60 50 40 30 20 10 0
(ppm)

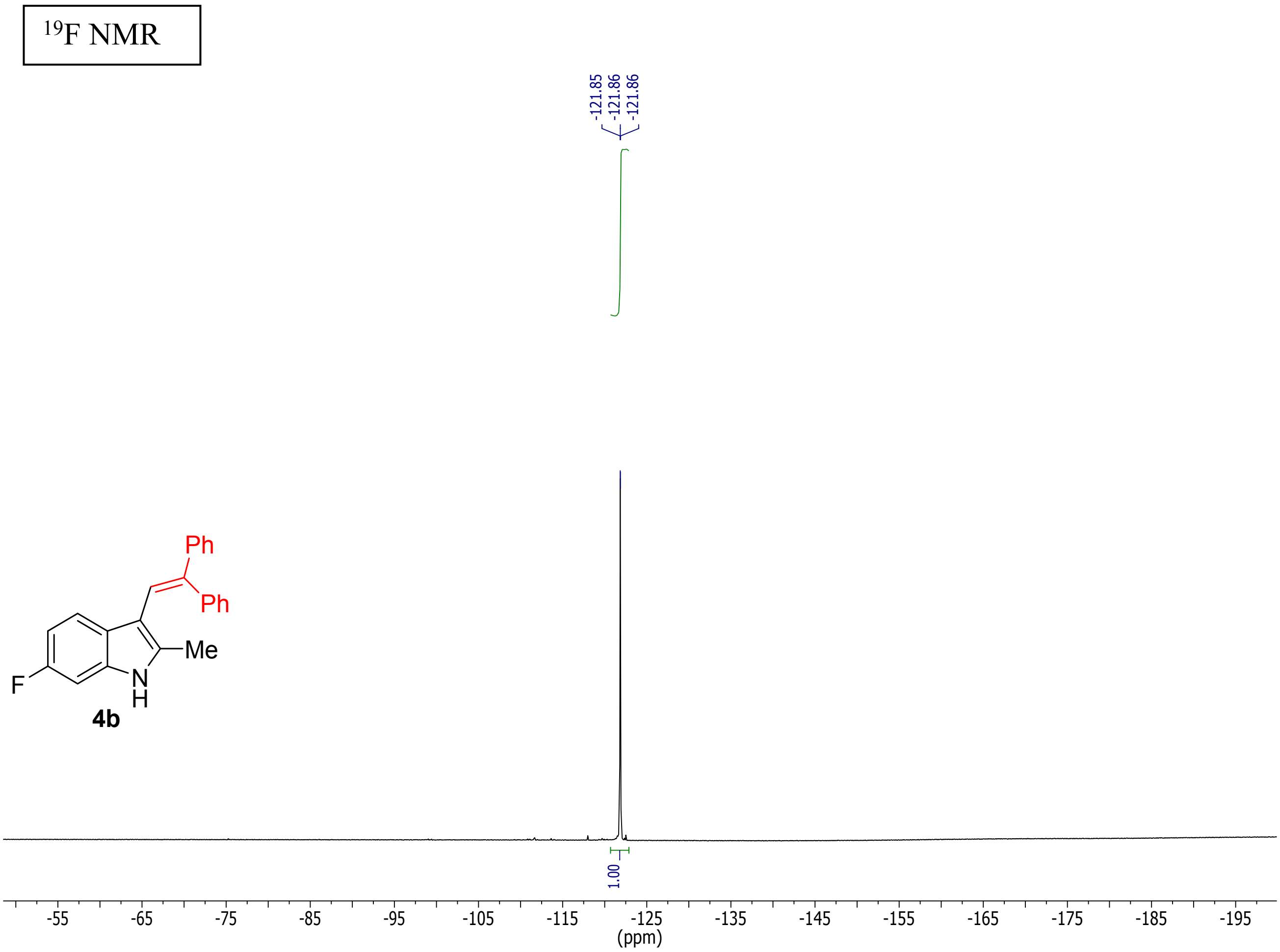

19F NMR
-121.85
-121.86
-121.86
Ph
Ph
Me
F
N
H
4b
1.00
-55
-65
-75
-85
-95
-105
-115
-125
-135
-145
-155
-165
-175
-185
-195
(ppm)

^{1}H NMR

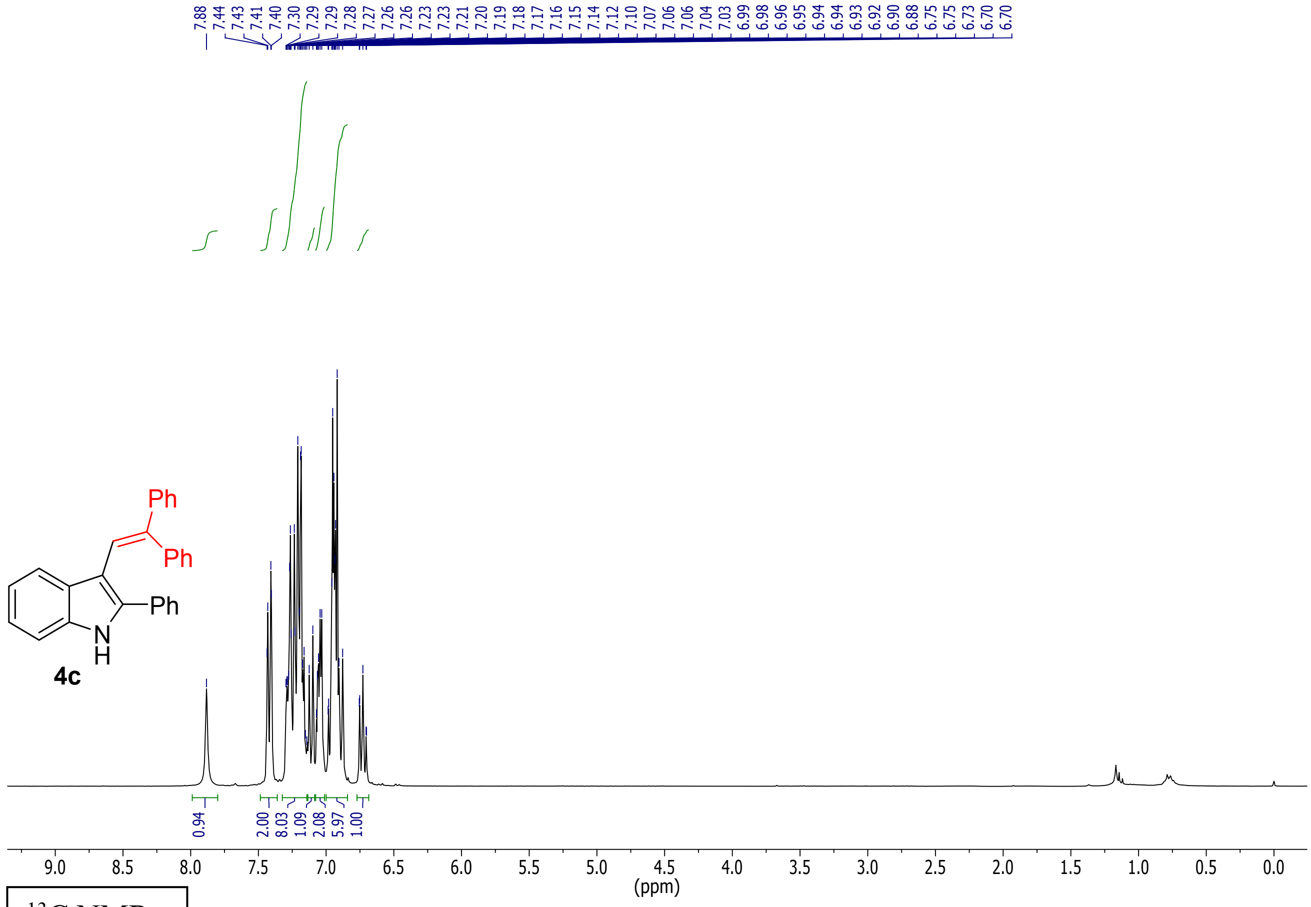

^{13}C NMR

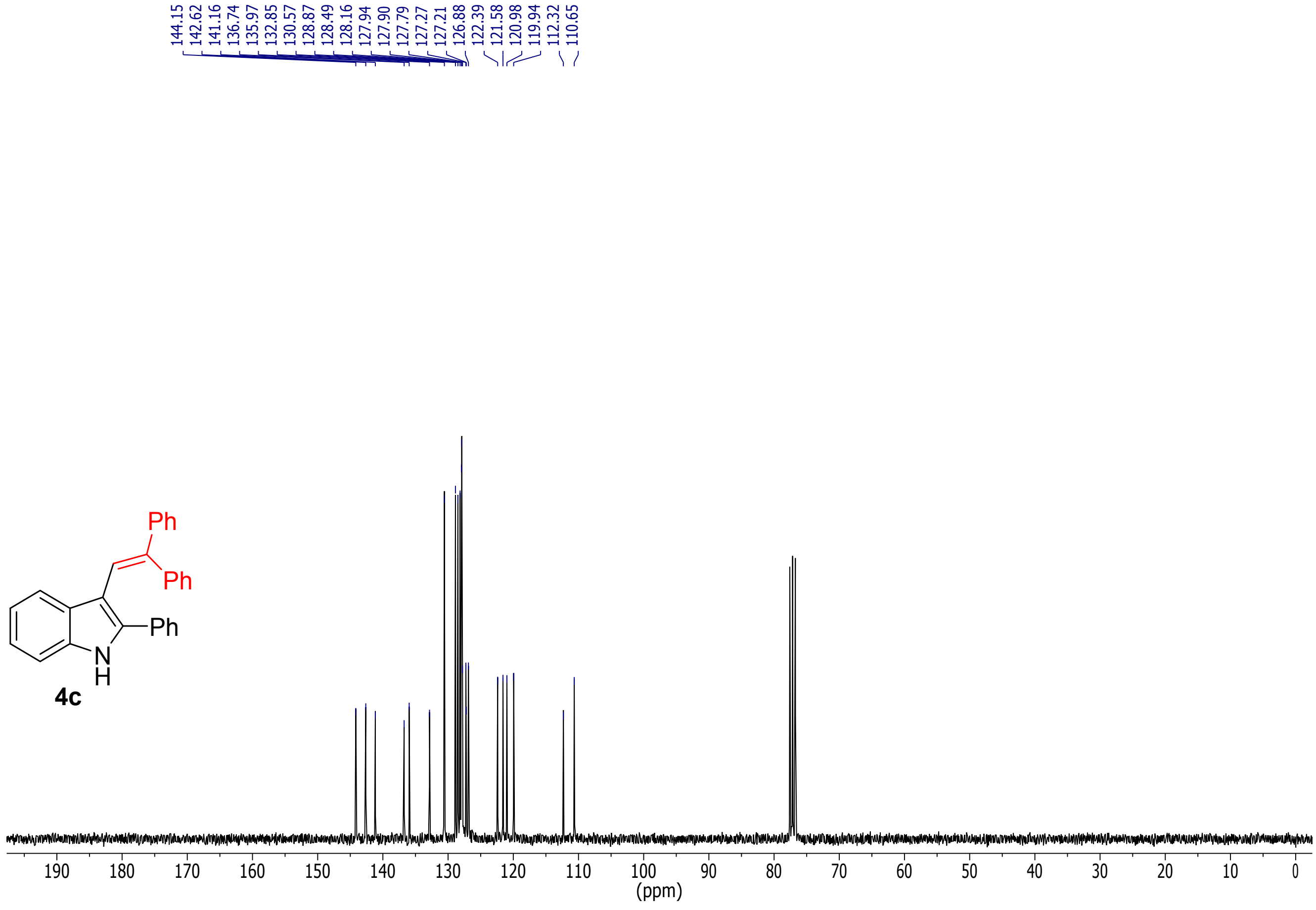

^{1}H NMR

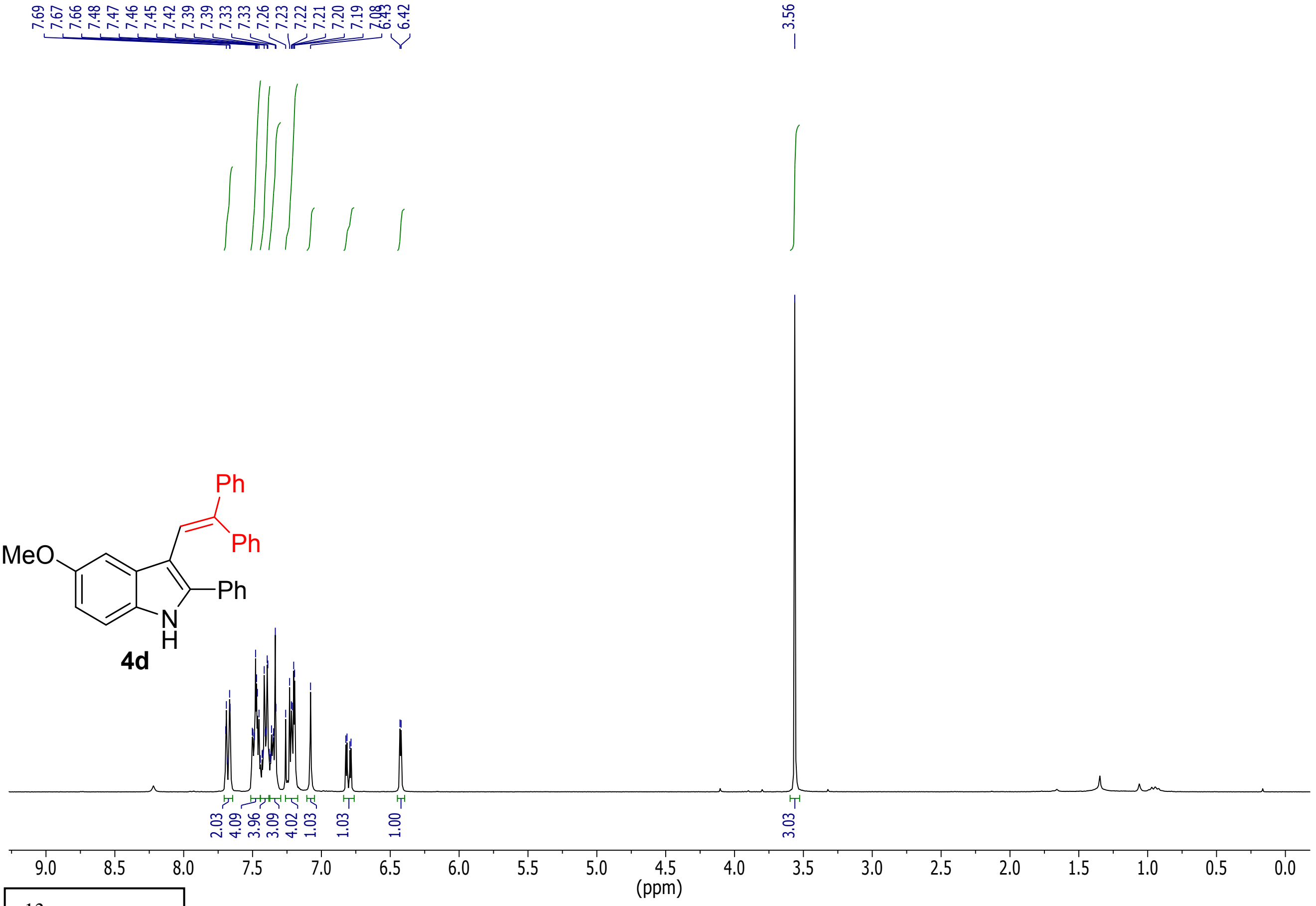

^{13}C NMR

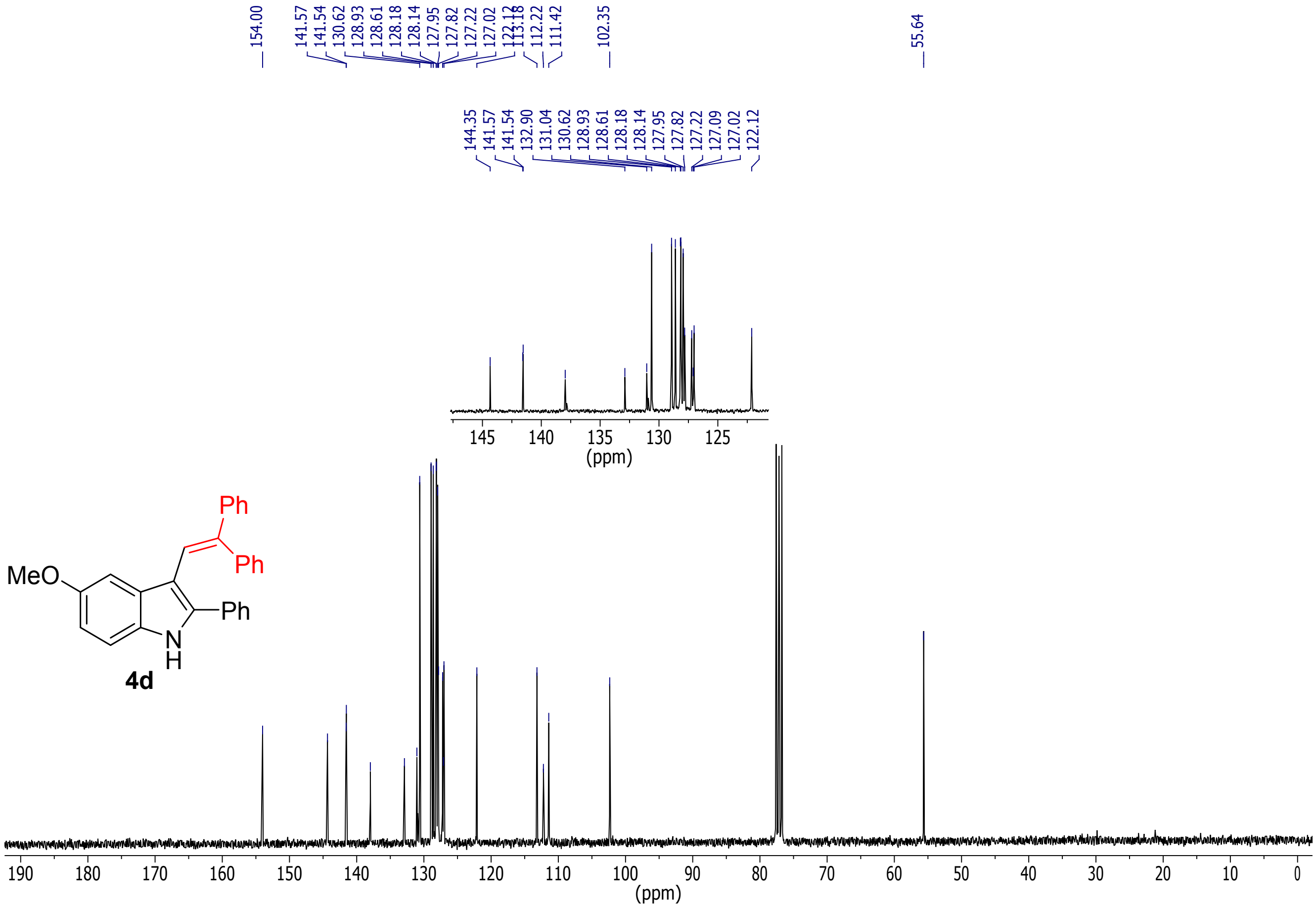

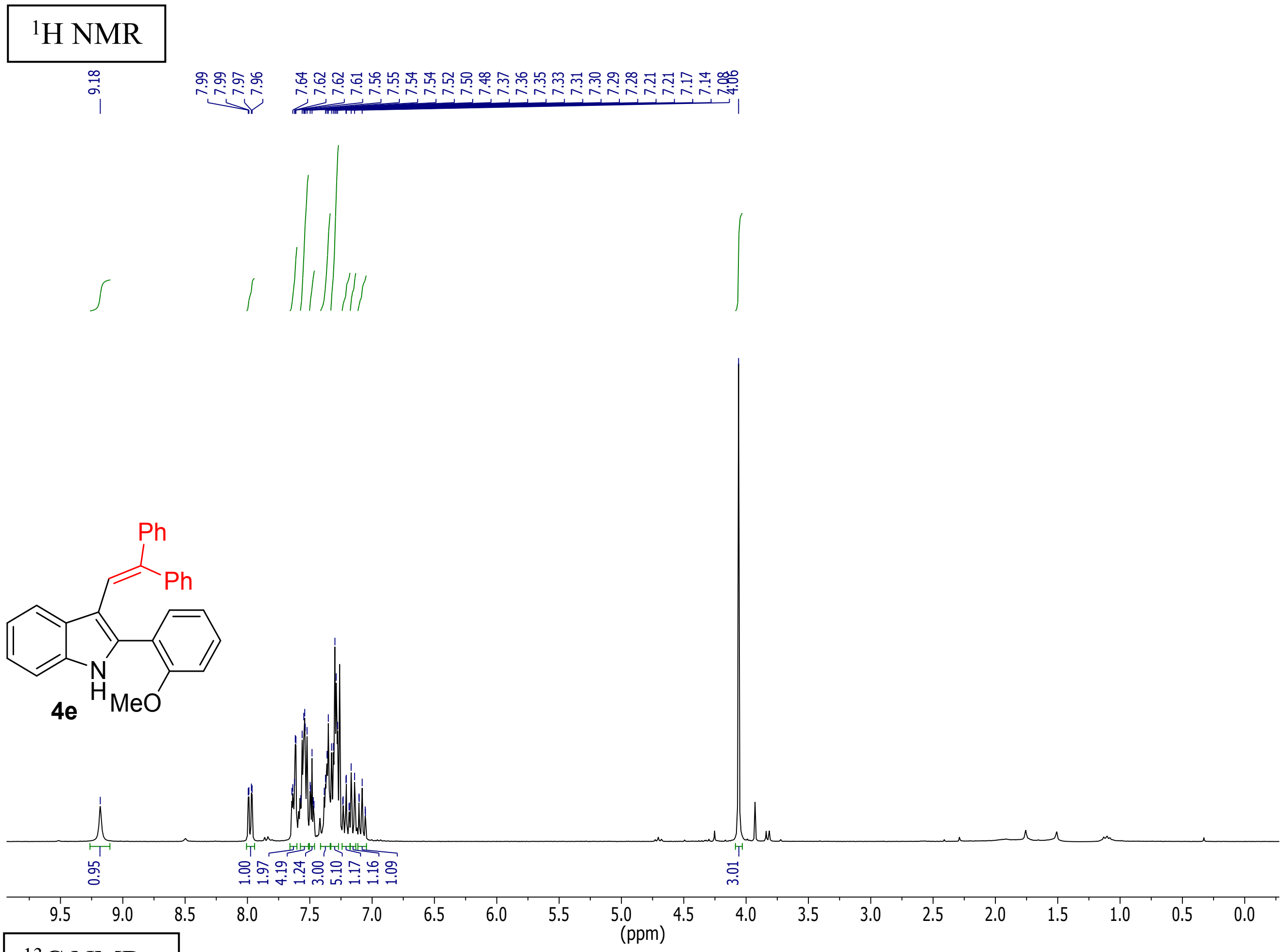

1H NMR
9.18
7.99
7.99
7.97
7.96
7.64
7.62
7.62
7.61
7.56
7.55
7.54
7.54
7.52
7.50
7.48
7.37
7.36
7.35
7.33
7.31
7.30
7.29
7.28
7.21
7.21
7.17
7.14
7.08
4.06
Ph
Ph
N
H
MeO
4e
0.95
1.00
1.97
4.19
1.24
3.00
5.10
1.17
1.16
1.09
3.01
(ppm)

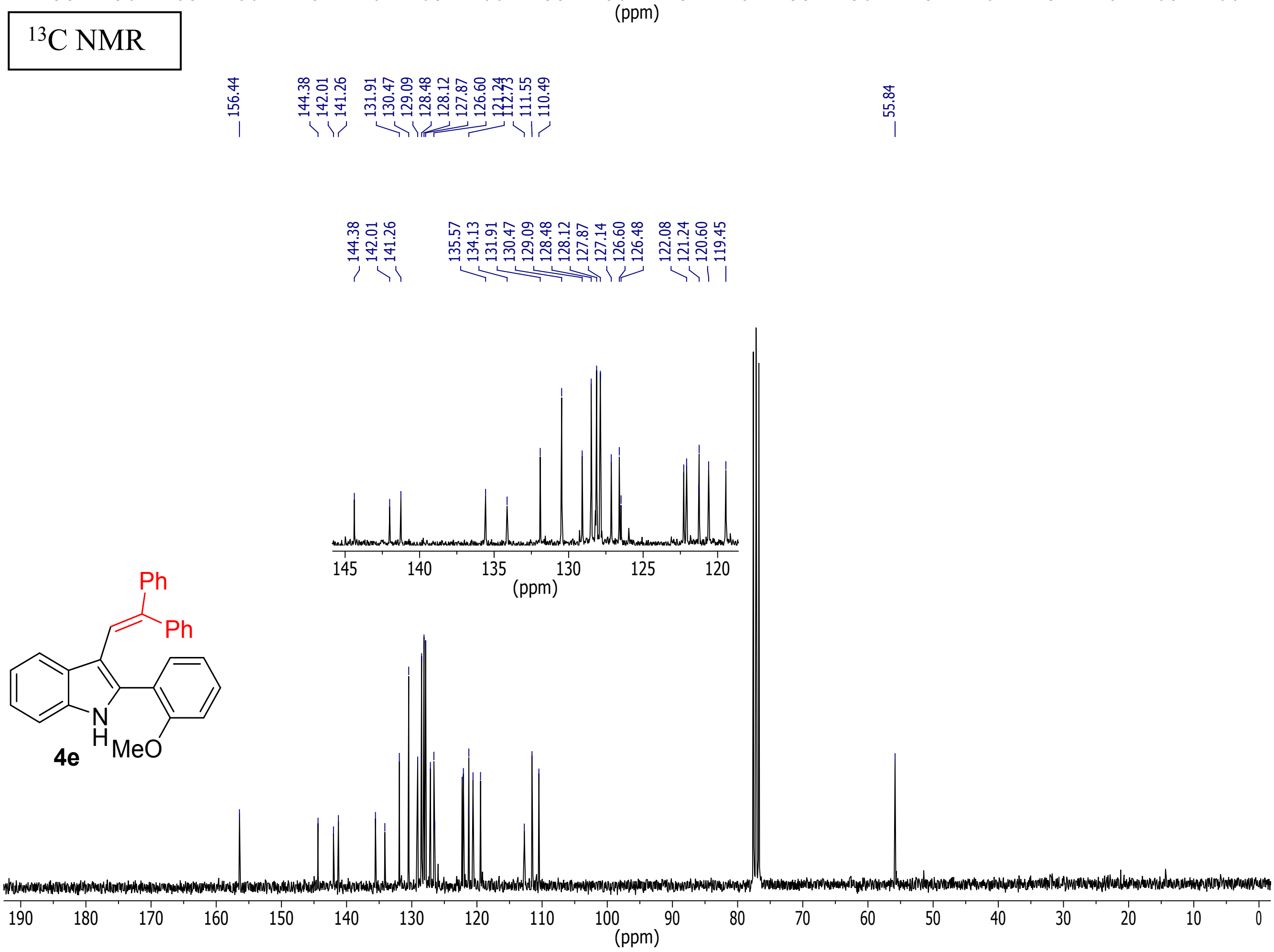

13C NMR
156.44
144.38
142.01
141.26
131.91
130.47
129.09
128.48
128.12
127.87
126.60
121.24
112.73
111.55
110.49
55.84
144.38
142.01
141.26
135.57
134.13
131.91
130.47
129.09
128.48
128.12
127.87
127.14
126.60
126.48
122.08
121.24
120.60
119.45
(ppm)
Ph
Ph
N
H
MeO
4e
(ppm)

^{1}H NMR

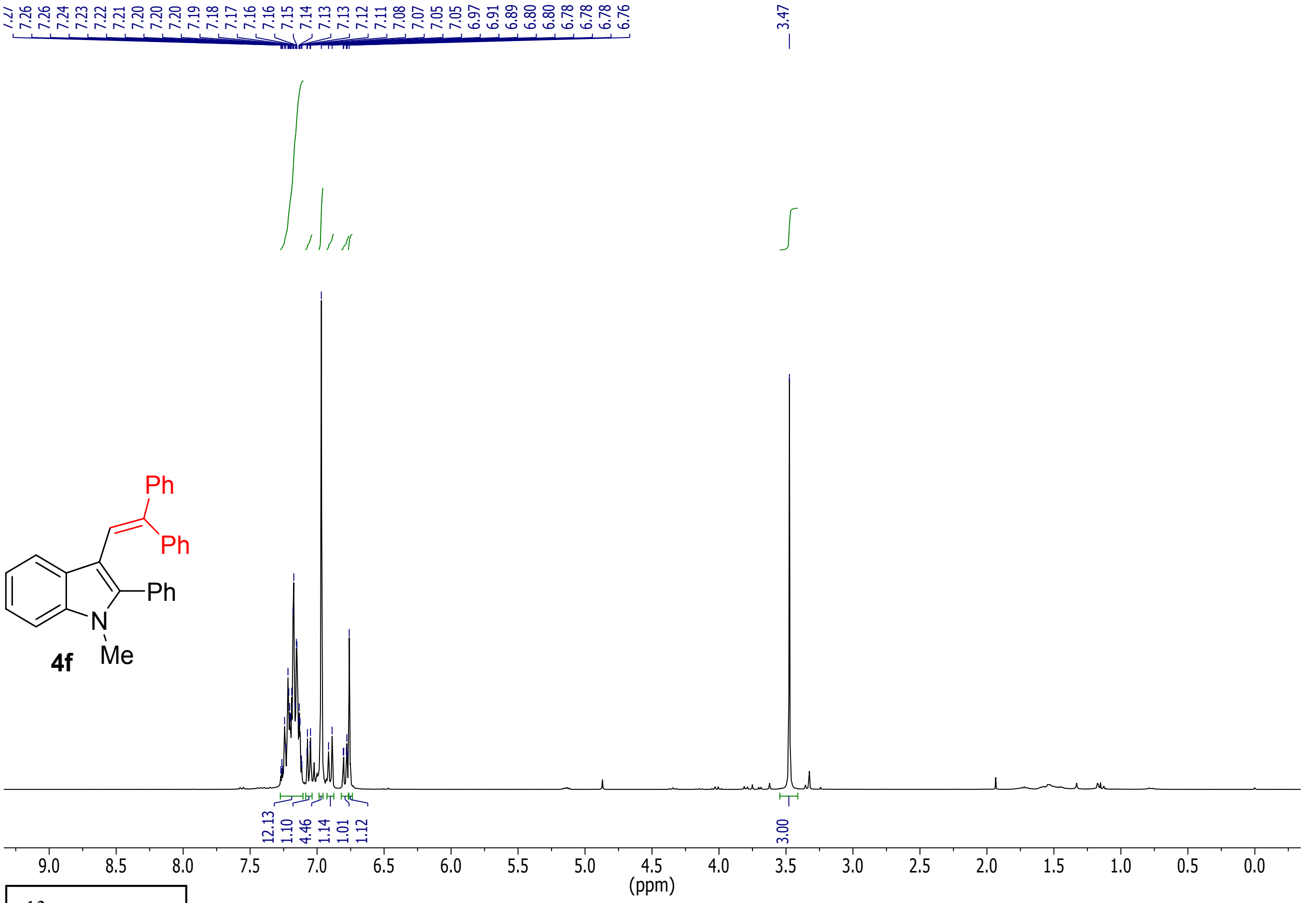

^{13}C NMR

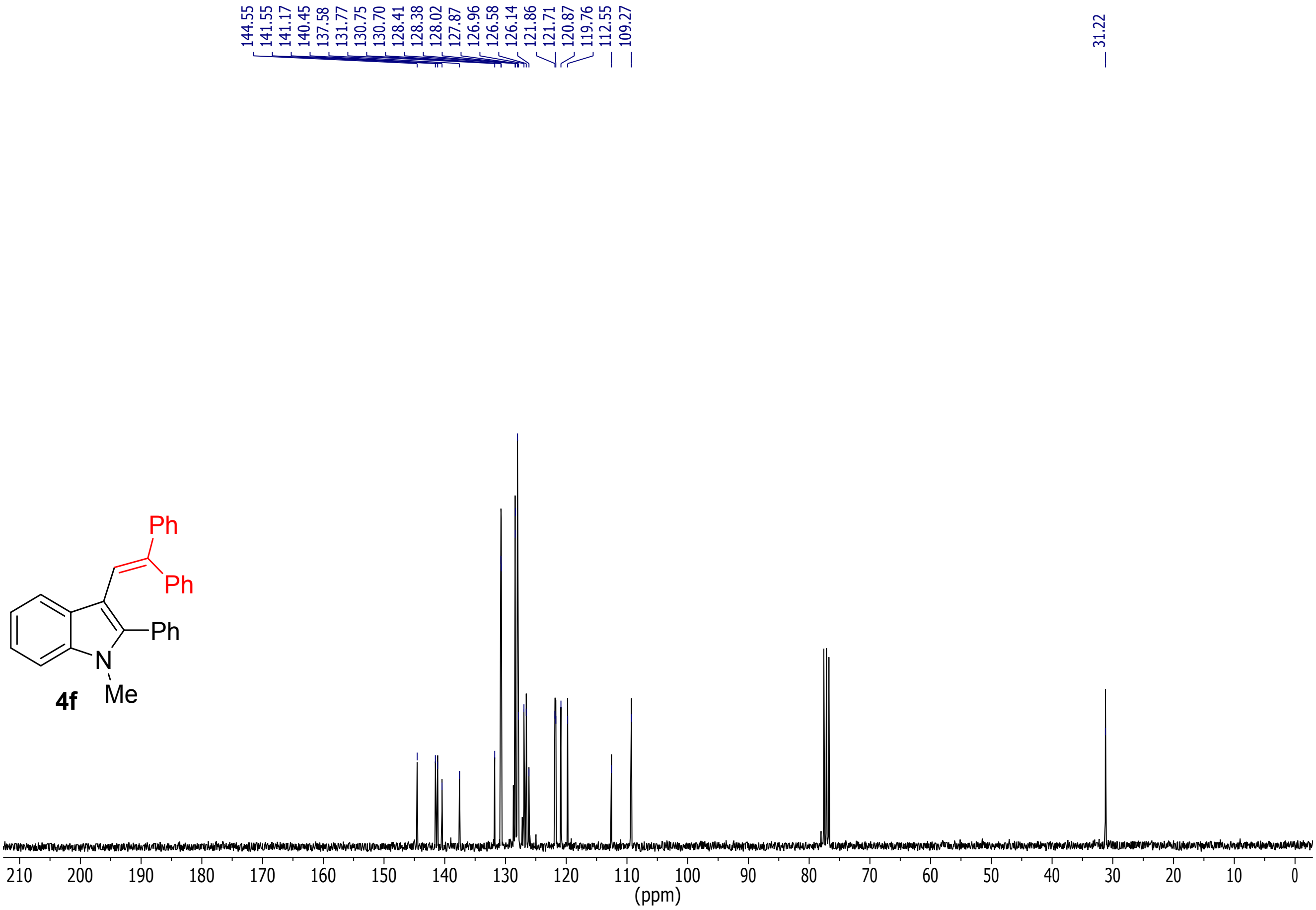

^{1}H NMR

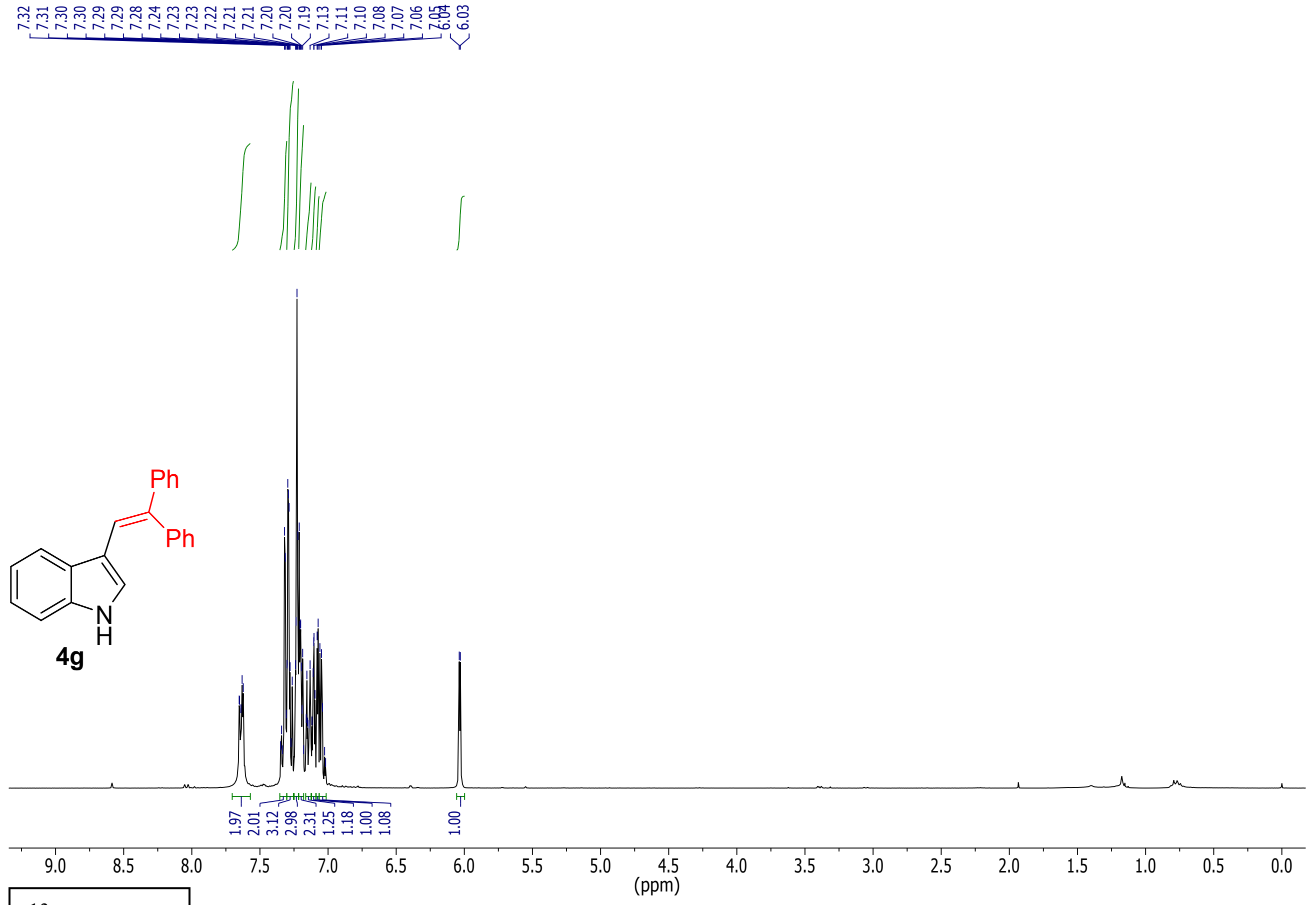

^{13}C NMR

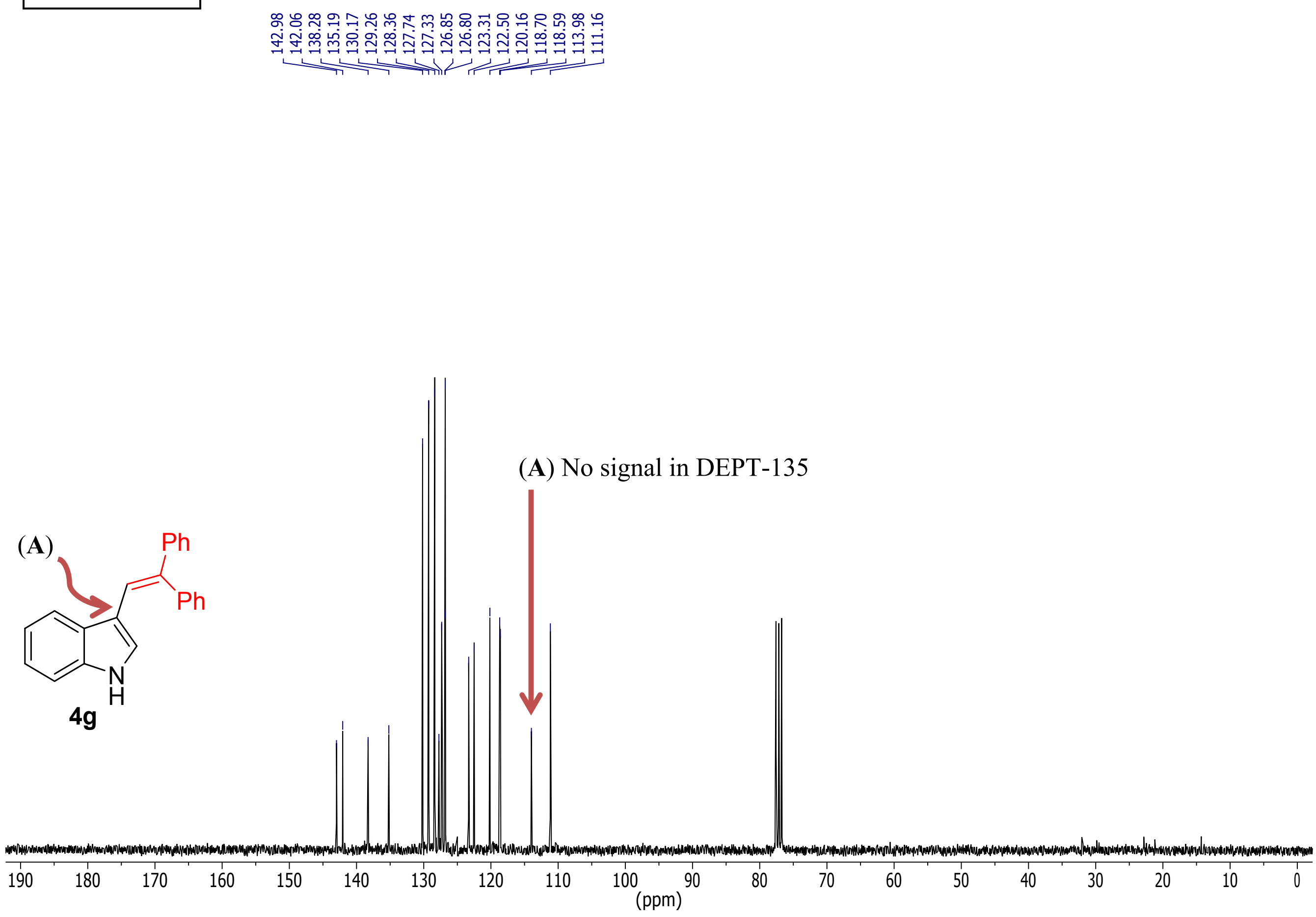

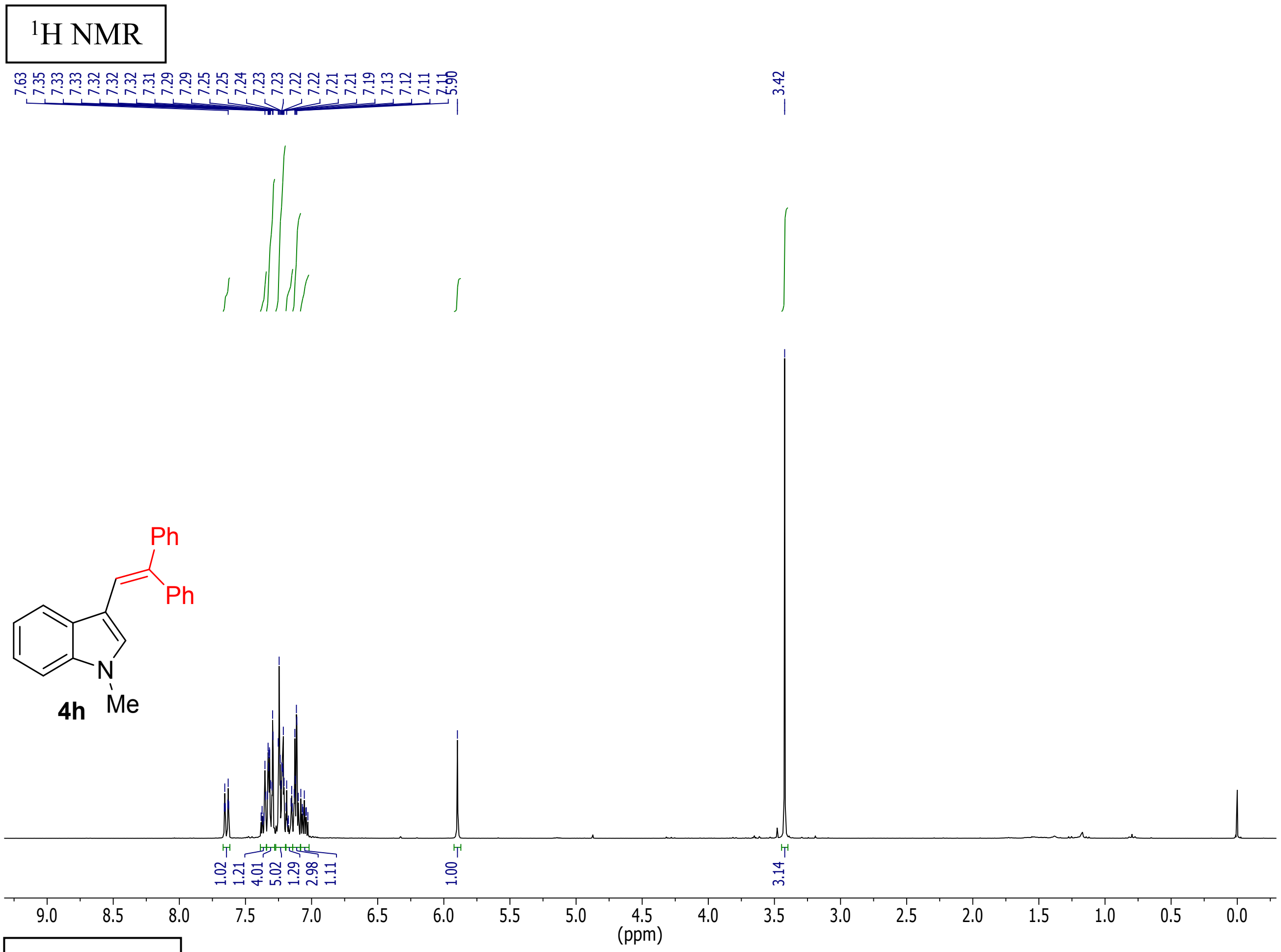

1H NMR
7.63
7.35
7.33
7.33
7.32
7.32
7.32
7.31
7.29
7.29
7.25
7.25
7.24
7.23
7.23
7.22
7.22
7.21
7.21
7.19
7.13
7.12
7.11
7.11
5.90
3.42
Ph
Ph
N
Me
4h
1.02
1.21
4.01
5.02
1.29
2.98
1.11
1.00
3.14
(ppm)

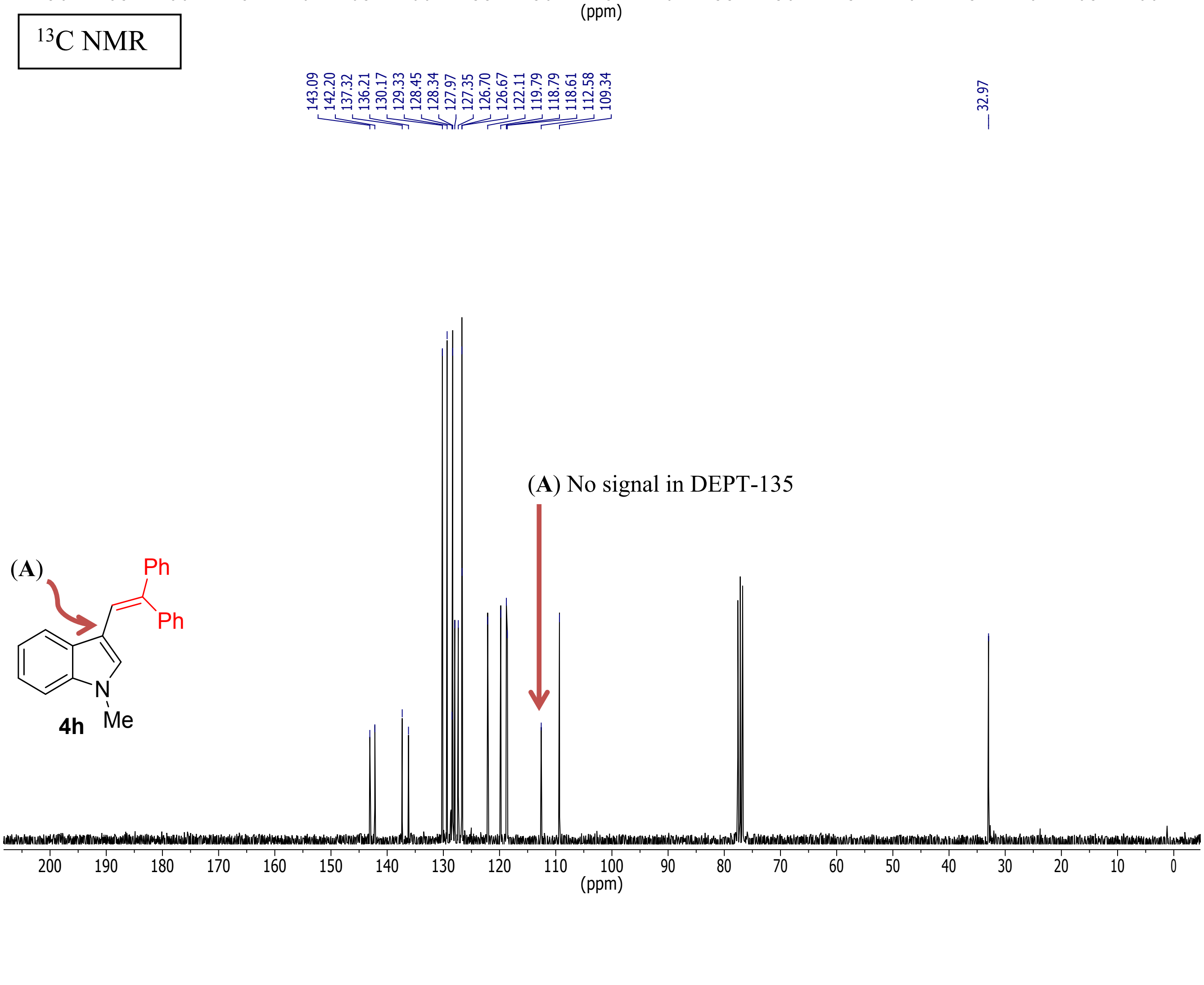

13C NMR
143.09
142.20
137.32
136.21
130.17
129.33
128.45
128.34
127.97
127.35
126.70
126.67
122.11
119.79
118.79
118.61
112.58
109.34
32.97
(A) No signal in DEPT-135
(A)
Ph
Ph
N
Me
4h
(ppm)

^{1}H NMR

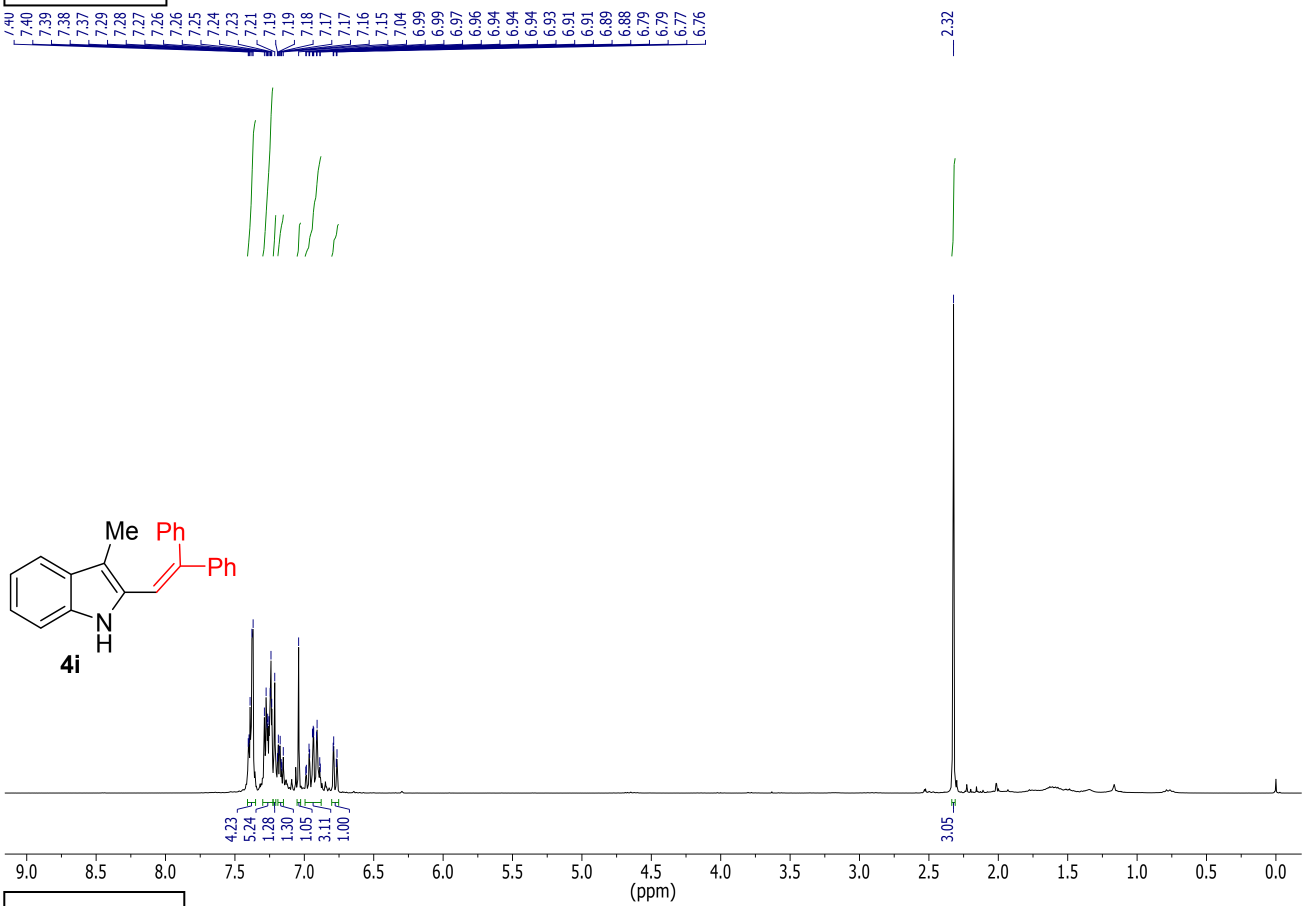

^{13}C NMR

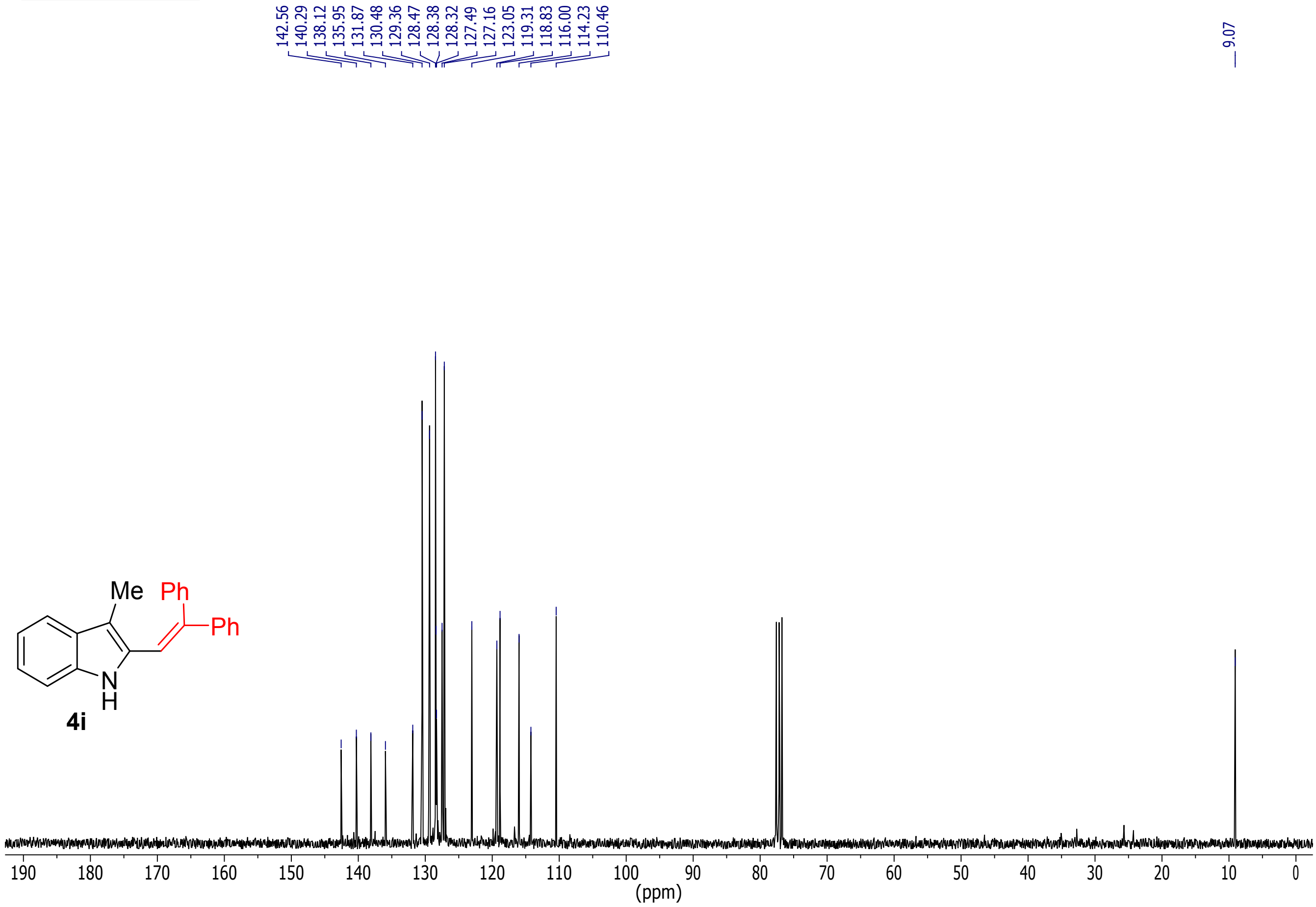

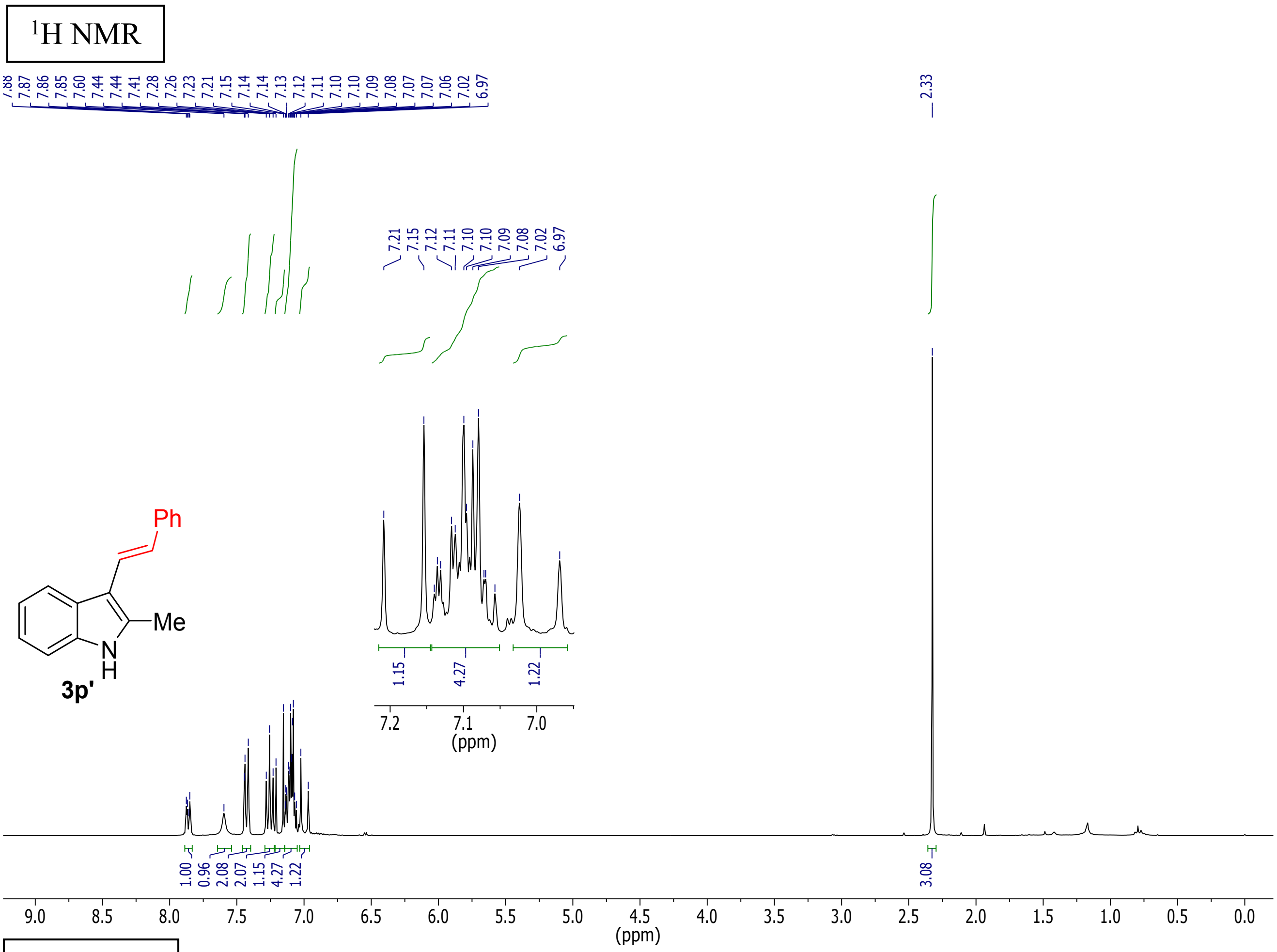

1H NMR
7.88
7.87
7.86
7.85
7.60
7.44
7.44
7.41
7.28
7.26
7.23
7.21
7.15
7.14
7.14
7.13
7.12
7.11
7.10
7.10
7.09
7.08
7.07
7.07
7.06
7.02
6.97
2.33
Ph
Me
N
H
3p'
1.00
0.96
2.08
2.07
1.15
4.27
1.22
3.08
1.15
4.27
1.22
7.2
7.1
7.0
(ppm)
9.0
8.5
8.0
7.5
7.0
6.5
6.0
5.5
5.0
4.5
4.0
3.5
3.0
2.5
2.0
1.5
1.0
0.5
0.0
(ppm)

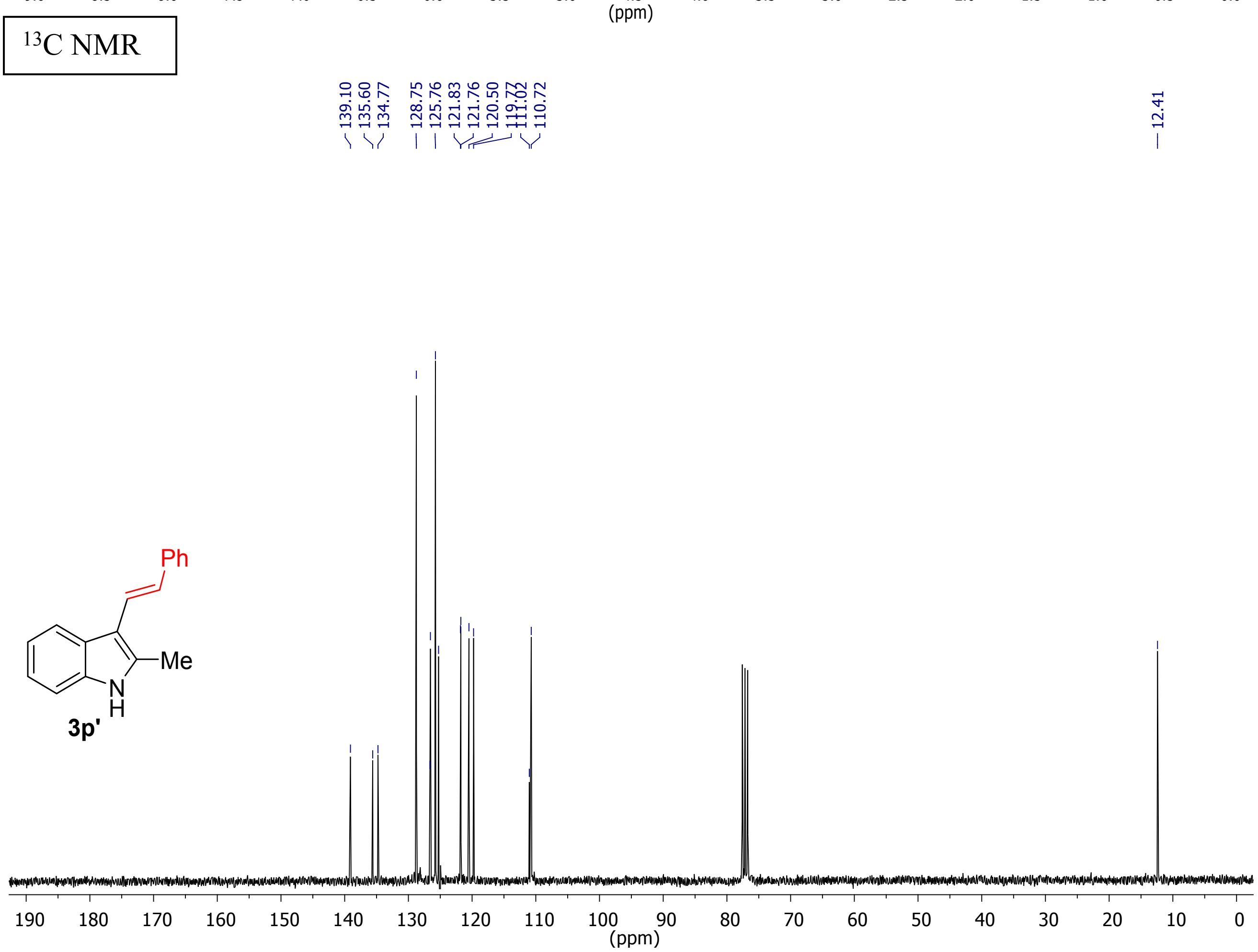

13C NMR
139.10
135.60
134.77
128.75
125.76
121.83
121.76
120.50
119.72
111.02
110.72
12.41
Ph
Me
N
H
3p'
190
180
170
160
150
140
130
120
110
100
90
80
70
60
50
40
30
20
10
0
(ppm)
